# Effect of long and short half-life PDE5 inhibitors on HbA1c levels: a systematic review and meta-analysis

**DOI:** 10.1016/j.eclinm.2024.103035

**Published:** 2024-12-31

**Authors:** Joseph Kim, Rui Zhao, Lawrence Richard Kleinberg, Kitai Kim

**Affiliations:** a Department of Biophysics, Johns Hopkins University, 3400 N Charles Street, Baltimore, MD, 21218, USA; b Department of Biochemistry and Molecular Genetics, University of Alabama Birmingham Heersink School of Medicine, Room 714, 1825 University Blvd., Birmingham, AL, 35294-2182, USA; c Department of Radiation Oncology and Molecular Radiation Sciences, Johns Hopkins University, Johns Hopkins Sidney Kimmel Comprehensive Cancer Center, 401 N. Broadway, Baltimore, MD, 21231, USA; d Human Stem Cell and Genome Engineering Center, University of California Los Angeles David Geffen School of Medicine, UCLA - CHS 36 - 125/133/143 650 Charles E. Young Dr. South, Los Angeles, CA, 90095, USA; e Department of Biological Chemistry, University of California Los Angeles David Geffen School of Medicine, UCLA - CHS 36 - 125/133/143 650 Charles E. Young Dr. South, Los Angeles, CA, 90095, USA; f Virginia University of Integrative Medicine, 1980 Gallows Road, Vienna, VA, 22182, USA

**Keywords:** PDE5 inhibitors, Tadalafil, Sildenafil, HbA1c, Meta-analysis

## Abstract

**Background:**

Phosphodiesterase 5 (PDE5) inhibitors, owing to their mechanism of action, have been gaining recognition as a potential case of drug repurposing and combination therapy for diabetes treatment. We aimed to examine the effect of long and short half-life PDE5 inhibitors have on Haemoglobin A1c (HbA1c) levels.

**Methods:**

A systematic review and meta-analysis was conducted of randomised controlled trials (RCTs) in people with elevated HbA1c (>6%) to assess mean difference in HbA1c levels from baseline versus controls after any PDE5 inhibitor intervention of ≥4 weeks, excluding multiple interventions. Cochrane CENTRAL, PMC Medline, ClinicalTrials.gov, and WHO ICTRP were searched without language restrictions up to September 30, 2024. Summary data from published data were extracted. PRISMA and Cochrane guidelines used to extract and assess data using a random-effects meta-analysis. This study is registered with the Research Registry, reviewregistry1733.

**Findings:**

Among 1096 studies identified, in analysis of 13 studies with 1083 baseline patients, long half-life PDE5 inhibitors (tadalafil, PF-00489791) had decreases in HbA1c while short half-life PDE5 inhibitors (sildenafil, avanafil) had no change. Five (38.5%) studies had a low risk of bias, and eight (61.5%) had some concerns. Long half-life inhibitors had significant mean decrease of −0.40% ([−0.66, −0.14], p = 0.002, I^2^ = 82%, 7.70% baseline HbA1c). Short half-life inhibitors had insignificant mean difference of +0.08% ([−0.16, 0.33], p = 0.51, I^2^ = 40%, 7.73% baseline HbA1c). In ≥8-week trials with participants with type 2 diabetes (T2D) and mean HbA1c ≥ 6.5%, long half-life inhibitors had significant mean decrease of −0.50% ([−0.83, −0.17], I^2^ = 88%, p = 0.003); short half-life inhibitors had significant mean increase of +0.36% ([0.03, 0.68], I^2^ = 3%, p = 0.03).

**Interpretation:**

At the well-controlled HbA1c of the participants, previous literature shows current diabetes treatments have similar HbA1c decreases, so the HbA1c mean difference of long half-life PDE5 inhibitors may indeed be clinically relevant. This suggests future investigation into PDE5 inhibitors as part of combination therapy or as therapy for high HbA1c individuals is needed, especially because of variable risk of biases, homogeneity, and sample sizes in our study.

**Funding:**

None.

**Research in context**

**Evidence before this study**

PubMed was first searched on June 20, 2023 without date or language restrictions using the search term “PDE5 inhibitor AND HbA1c”. We identified a meta-analysis published in 2016, which reported no statistically significant effects of Phosphodiesterase 5 (PDE5) inhibitors on Haemoglobin A1c (HbA1c). However, this meta-analysis only included sildenafil, a PDE5 inhibitor with a half-life that may be too short to show significant effects, thus highlighting the need for further investigation.

**Added value of this study**

In adults with elevated HbA1c levels, this study reveals that long half-life PDE5 inhibitors (tadalafil, PF-00489791) had a statistically significant decrease (p = 0.002) in HbA1c, with mean difference similar to those of other diabetes medications in previous literature given the baseline HbA1c and thus may be clinically relevant. Comparatively, short half-life PDE5 inhibitors (sildenafil, avanafil) had no decrease in HbA1c or a statistically significant increase (p = 0.03) in HbA1c when considering a subset most representative of type 2 diabetes, thereby confirming the conclusion from earlier meta-analyses.

**Implications of all the available evidence**

Long half-life PDE5 inhibitors may represent promising candidates for drug repurposing for diabetes management, especially because they are readily available, increasingly affordable, and have limited major side effects. Because PDE5 inhibitors may have a unique mechanism, they may be promising in combination therapy. More clinical trials are necessary to optimise their pharmacokinetics, particularly in populations with high baseline HbA1c levels. Furthermore, the potential impact of PDE5 inhibitors should be considered in future clinical trials among patients with diabetes; the assumption that PDE5 inhibitor usage is trivial should be investigated as they are frequently prescribed.

## Introduction

Diabetes is a pervasive global health concern, with its prevalence steadily rising worldwide. Among the numerous challenges posed by diabetes, the management of Haemoglobin A1c (HbA1c) levels stands as a paramount priority within the realm of public health. HbA1c levels reflect average glycaemia and is regarded as the gold standard for assessing glycaemic control, having strong correlation with diabetes complications.^1^ Elevated HbA1c levels are not only associated with an augmented risk of mortality, particularly in terms of cardiovascular health,^2^ but also intricately associated with microvascular complications in diabetic individuals. One significant facet of diabetes is the development of cardiac dysfunction, where vascular endothelial dysfunction plays a pivotal role.

Drug repurposing has gained traction as an efficient method for identifying new therapeutic uses for existing medications, bypassing some of the early safety testing required for new drugs.^3^ Moreover, combination therapy is becoming recognised as a more effective way to control diabetes compared to monotherapy, as seen in meta-analysis.^4^ Phosphodiesterase-5 (PDE5) inhibitors, commonly used for erectile dysfunction and pulmonary arterial hypertension, are emerging as potential candidates for repurposing in diabetes management due to their favourable safety profile and potential cardiovascular benefits.

Recent clinical evidence suggest that PDE5 inhibitors may improve glycaemic control by enhancing endothelial function, which could lead to improved glucose transport and insulin sensitivity.^5^, ^6^, ^7^ These inhibitors also reduce inflammation, oxidative stress, and improve nitric oxide production, factors commonly impaired in type 2 diabetes.^7^

Several mechanistic studies suggest that PDE5 inhibitors have the potential to reduce HbA1c levels in diabetic patients and may be considered as a viable treatment option. A potential mechanism by which PDE5 inhibitors improve diabetes-related symptoms is through enhancement of insulin production and sensitivity. PDE5 inhibitors act by increasing cyclic guanosine monophosphate (cGMP),^8^ which in turn stimulates cyclic adenosine monophosphate (cAMP) and the phosphoinositide 3-kinase/protein kinase B (PI3K/AKT) pathway, which simulates glucose uptake without the use of an insulin intermediary (Supplementary Fig. S2a).^9^

Moreover, medications acting upon intermediates in the pathway have favourable effects on HbA1c (Supplementary Fig. S2b). Indeed, praliciguat, a soluble guanylyl cyclase (sGC) stimulator, has been shown to decrease HbA1c levels. Alpelisib and cisplatin, which are PI3K inhibitors, have been seen to increase HbA1c.^10^ The existing antihyperglycemic agent classes of sulfonylureas and meglitinides act by increasing calcium (Ca^2+^) concentration, albeit often investigated in the context of beta cells.^11^

This pathway is not the primary target of many first-line therapies, including medications such as metformin,^12^ suggesting the potential effectiveness of combination therapy.^4^ Furthermore, PDE5 inhibitors are considered safe because they are already commonly prescribed with few side effects.^13^

PDE5 inhibitors with longer half-lives, such as tadalafil, are commonly preferred over others by patients^14^ and can offer improved medication adherence and persistence.^15^ Addressing kinetics, longer half-lives can allow more stable glucose control, especially if half-life is so short that the medication is significantly metabolised during sleep.

Despite these promising indications, no comprehensive meta-analysis has yet examined the overall impact of different half-lives of PDE5 inhibitors on HbA1c levels in individuals with diabetes. A 2016 meta-analysis evaluated the effects of PDE5 inhibitors but found no significant results, as only data on sildenafil was available. Since then, several key clinical trials involving other PDE5 inhibitors have been conducted and published.^16^ However, a recent spate of clinical trials performed since 2022 involving a broader range of PDE5 inhibitors, alongside greater availability of search data, posits a reinvestigation.

In this study, we seek to address this crucial gap in the literature by conducting a systematic review and meta-analysis to evaluate the effects of various PDE5 inhibitor medication on HbA1c levels. Through a comprehensive synthesis of existing clinical trials, we aim to provide a deeper understanding of PDE5 inhibitors' potential in improving glycaemic control among individuals with diabetes, shedding light on its implications for both clinical practice and future research endeavours. We particularly compare the effect on HbA1c by the long half-life PDE5 inhibitors tadalafil and PF-00489791's (half-life: 15 h,^17^ 17.5 h^18^) to the effect on HbA1c by the short half-life PDE5 inhibitors sildenafil and avanafil (half-life: 4 h^18^).

## Methods

### Search strategy and selection criteria

A systematic review with meta-analysis was conducted. The study adhered to Cochrane and PRISMA guidelines.^19^^,^^20^

We included only randomised controlled trials (RCTs). Other trial design types, such as case-control studies, observational studies, or case reports were excluded. We included studies performed with human participants with mean baseline HbA1c of at least 6% (to only include those with elevated HbA1c levels but not exclude those diagnosed with type 2 diabetes via other accepted methods such as fasting glucose). RCTs were included regardless of erectile dysfunction or pulmonary arterial hypertension status, gender or sex, or location.

We included studies with an intervention of any dosage of chronic PDE5 inhibitor treatment for at least 4 weeks that included a control/placebo. We excluded studies and participant populations for which a different intervention was adjusted as a variable in combination therapy with PDE5 inhibitor.

We chose to allow trials as short as 4 weeks because this would be sufficient to meaningfully reflect a drop in HbA1c, even if underestimated. Indeed, Michaelis–Menten models of HbA1c predict that a medication should have 34.2% of its maximal effect by 30 days, 56.6% by 60 days, and 71.4% by 90 days, so the mean difference at 30 days is approximately half that at 90 days (Supplementary Fig. S4). Subgroup analysis on longer trial lengths is performed to avoid underestimation of results.

**Fig. 1:**
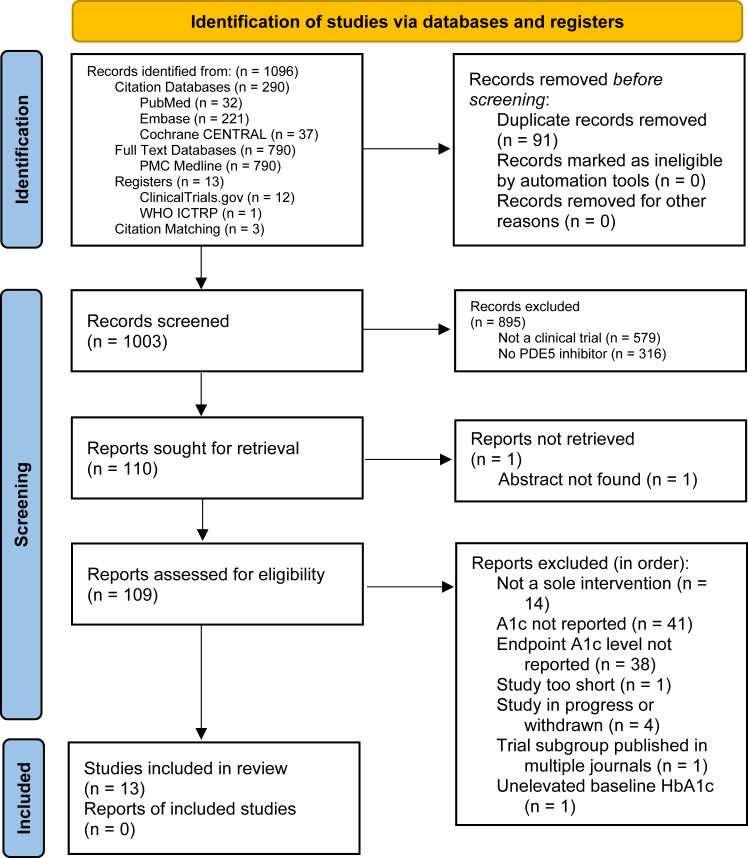
**PRISMA flowchart**. Due to the nature of haemoglobin A1c (HbA1c) concentration reporting, the full text was analysed where possible. Abbreviations: World Health Organization International Clinical Trials Registry Platform (WHO ICTRP), Cochrane Central Register of Controlled Trials (Cochrane CENTRAL), PubMed Central (PMC).

In total, 6 databases and registries were searched (Fig. 1). The search terms used are provided (Supplementary Fig. S5). The databases of citations and abstracts searched from inception to September 28, 2024 were MEDLINE (PubMed), Elsevier Embase, and the Cochrane Central Register of Controlled Trials (CENTRAL). The full-text databases searched from inception to September 28, 2024 was PubMed Central (PMC). The following trial registries searched from inception to September 28, 2024 were ClinicalTrials.gov by the United States National Library of Medicine and the World Health Organization International Clinical Trials Registry Platform (WHO ICTRP).

Citation matching was also performed where relevant. All search methods were completed without restriction on language. Where necessary, articles were translated using Google Translate (Mountain View, CA) or DeepL Translator (Cologne, Germany). Summary estimates were retrieved.

All databases were independently searched by two review authors (both JK and KK) and any disagreements were resolved through discussion. If necessary, a third person would have been involved for resolution. Due to the routine nature of HbA1c level reporting, many studies did not report HbA1c data in abstracts or article metadata, so all studies were examined and analysed by their full text where possible. All data were extracted and analysed by both JK and KK until agreement. Two authors (JK and KK) independently extracted and analysed data used the Risk of Bias 2.0 tool.

The protocol was unpublished.

### Data analysis

Data was extracted from article text, tables, figures, or Supplementary Data where applicable. Microsoft Office 365 (Redmond, Washington, USA) and Python 3.12 (Wilmington, Delaware, USA) were used to assist with data extraction and importation to Google Sheets. The Google Docs Editors suite (Mountain View, California, USA) was utilised to process and tabulate results. Data was extracted to Google Sheets and verified for agreement before transfer into RevMan 5.4.1 and to RevMan Web (London, UK).

Due to the routine nature of HbA1c level reporting, many studies did not report HbA1c data in abstracts or article metadata, so all studies were examined and analysed by their full text where possible. After search results were imported into Google Sheets, duplicates were automatically removed if any of the following ID numbers (as reported by searched databases) were duplicated: Embase PUI; PubMed ID; PMC ID; Cochrane CENTRAL ID; ClinicalTrials.gov ID; CINAHL ID; WHO ICTRP ID; DOI.

The primary outcome measured was HbA1c. No restrictions on measurement technique were placed; both NGSP and IFCC measurements were included. HbA1c must have been measured and reported in both experimental and control groups.

Secondary analysis was performed on homoeostatic model assessment—insulin resistance (HOMA-IR), body mass index (BMI), fasting glucose, 2 h post prandial glucose, fasting insulin, cholesterol, triglycerides, high-density lipoprotein (HDL), and low-density lipoprotein (LDL). Due to protocol design, data for all secondary analysis was only collected from studies also reporting HbA1c. Outcome data was collected from endpoint data or from change-from-baseline data and baseline data, where applicable.

Adverse event assessments were not performed due to well-established adverse event data. Race or ethnicity data was not collected due to lack of data granularity.

We used a random-effects model to partially account for potential heterogeneity in factors such as medication dosage, medication intervals, or population differences. Risk of bias analysis for quality of data was completed using the Risk of Bias 2.0 (RoB 2.0) tool (Supplementary Figs. S36–S50). The appropriate RoB 2.0 tool was utilised according to the study design, specifically for parallel trials and crossover trials.

To test the robustness of the results found, several sensitivity analyses, including one excluding studies with high risk of bias, were performed. Analysis of the impact of choosing to report data in NGSP units of %: results reported in IFCC units of mmol/mol were generated, although the conversion is linear and would only result in differences due to rounding error (Supplementary Figs. S6–S24c and d). Analysis of the impact of choosing a subgroup of studies lasting at least 8 weeks and with only participants with type 2 diabetes as well as having a mean baseline HbA1c of at least 6.5%: results under subgroups with any combination of these three criteria were generated (Supplementary Figs. S6–S13). Analysis of the impact of choosing to include all participants regardless of dosage: results under only including participant arms receiving the lower/higher dosage were generated (Supplementary Figs. S14 and S15). Analysis of the impact of choosing to only include data from the longer time period: results under only including data from the shorter time period were generated (Supplementary Fig. S16). Analysis of the impact of choosing to group trials by half-life of PDE5 inhibitors: results under grouping trials by the medication administered were generated (Supplementary Fig. S17). Analysis of the impact of choosing to include all trials regardless of medication scheduling: results under only including trials with consistent, time-based intervention administration were generated (Supplementary Fig. S18). Analysis of the impact of choosing to include all studies regardless of overall risk of bias: results under grouping trials by assessed risk of bias were generated (Supplementary Fig. S19). Analysis of the impact of choosing a random-effects model: results under a fixed-effects model were generated (Supplementary Figs. S19 and S20). Analysis of the impact of allowing data estimation: results under subgroups allowing only studies reporting data in methods not needing data estimation (such as by using change-from-baseline statistics or by using median and quartile measures) were generated (Supplementary Fig. S22). Analysis of the impact of subjective outliers: results under excluding subjective outliers were generated (Supplementary Figs. S23 and S24). Analysis of the impact of grouping PDE5 inhibitors by half-life: results under combining all data were generated (Supplementary Fig. S25).

For main analysis, data parameters retrieved were number of patients, specific PDE5 inhibitor used in intervention, period of treatment, diabetes status, and outcome data. For risk of bias analysis, all baseline statistics were also extracted. For study characterisation, the year range of recruitment, trial design, blinding type, and country of recruitment were also extracted.

Data normalisation was performed in accordance with Cochrane guidelines or by using normal distribution statistics where applicable.

The stratification of studies on the bases of administration of short and long half-life inhibitors depended on the potential requirement for medication administration frequency to exceed once daily, as based on half-life. For the four PDE5 inhibitors approved by the Food and Drug Administration (FDA) in the United States, this would place avanafil (4 h), sildenafil (4 h), and vardenafil (4 h) as a short half-life PDE5 inhibitor and tadalafil (17.5 h) as a long half-life inhibitor.^18^ For other available PDE5 inhibitors not approved by the FDA but could be available especially in other sovereign states, this would place lodenafil (2 h) and mirodenafil (2.5 h) as a short half-life PDE5 inhibitor and udenafil (12 h) as a long half-life PDE5 inhibitor. Other PDE5 inhibitors which may be found during search would be classified according to similar guidelines.

Because HbA1c concentrations reflect the average blood sugar level over the past 2–3 months^21^ and is most relevant to the treatment of type 2 diabetes, and because of other methods to diagnose type 2 diabetes (such as through fasting plasma glucose), subgroup analysis was done, only including trials which lasted at least 2 month and exclusively recruited participants with type 2 diabetes as well as having a mean baseline HbA1c of at least 6.5%. The main analysis including all trials is still relevant, however, as only trials with elevated HbA1c levels were included, and a short duration likely has the effect of underestimating any effect on HbA1c.

As per Cochrane guidelines, visual inspection of forest plots as well as Chi^2^ and more importantly I^2^ statistics were used to assess heterogeneity.

Statistical analysis, confidence analysis, heterogeneity analysis, forest plot generation, sensitivity analysis, and funnel plot (reporting bias) analysis was done in RevMan 5.4.1 and in RevMan Web (London, UK). RevMan forest plots were enhanced using Adobe Illustrator (San Jose, California, USA).

The meta-analysis is registered on Research Registry (reviewregistry1733) and is available online (https://www.researchregistry.com/browse-the-registry#registryofsystematicreviewsmeta-analyses/registryofsystematicreviewsmeta-analysesdetails/65519717f8cb970026361b5f/).

Differences between protocol and review can be found in the Supplementary extended, structured methods (Supplementary Fig. S3).

### Role of the funding source

There was no funding source for this study. JK and KK have access to and verify the underlying study data. JK and KK were responsible for the decision to submit the manuscript.

## Results

We identified 1096 records (Fig. 1), out of which 13 studies met the inclusion criteria (baseline n = 1083) (Table 1).^22^, ^23^, ^24^, ^25^, ^26^, ^27^, ^28^, ^29^, ^30^, ^31^, ^32^, ^33^, ^34^ No studies meeting inclusion criteria were excluded. Clinical trials were conducted in a wide diversity of locations. 8 were performed in the European Region, 2 were performed in the Eastern Mediterranean Region, 1 was performed in the Western Pacific Region, 1 was performed in the Region of the Americas, and 1 spanned multiple regions.

**Table 1:** Study characteristics.

	Date range (recruitment)	Intervention	Duration	Trial type	Trial design	Blinding	Country of recruitment	n (baseline)	n (control)
Hegazy et al. (2024)^22^	2022–2023	Tadalafil	6 months	RCT	Parallel	Open label	Egypt	60	30
Fryk et al. (2023)^23^	2016–2019	Tadalafil	6 weeks	RCT	Crossover	Double blind	Sweden	18	18
Pofi et al. (2022)^24^	2014–2018	Tadalafil	20 weeks	RCT	Parallel	Double blind	Italy	122	61
Lee et al. (2022)^25^	2017–2018	Tadalafil	6 months	RCT	Parallel	Double blind	Republic of Korea	68	23
Derosa et al. (2022)^26^	Not reported	Avanafil	3 months	RCT	Parallel	Double blind	Italy	61	30
Liu et al. (2016)^27^	2011–2014	Sildenafil	12 weeks	RCT	Parallel	Double blind	The Netherlands	52	26
Scheele et al. (2016)^28^	Not reported	PF-00489791	12 weeks	RCT	Parallel	Double blind	Multiple	248	61
Kirilmaz et al. (2015)^29^	2010–2011	Sildenafil	3 months	RCT	Parallel	Not reported	Türkiye	83	41
Khazaal et al. (2014)^30^	2014	Tadalafil	8 weeks	RCT	Parallel	Double blind	Iraq	40	20
Giannetta et al. (2012)^31^	2008–2009	Sildenafil	12 weeks	RCT	Parallel	Double blind	Italy	59	29
Morano et al. (2007)^32^	2002–2003	Sildenafil	12 weeks	RCT	Parallel	Double blind	Italy	16	8
Grover-Páez et al. (2007)^33^	Not reported	Sildenafil	30 days	RCT	Parallel	Double blind	Mexico	40	20
Sáenz de Tejada et al. (2002)^34^	1999–2000	Tadalafil	12 weeks	RCT	Parallel	Double blind	Spain	216	71

The study characteristics of the 13 included trials are tabulated.

Out of 7 studies with long half-life PDE5 inhibitors (tadalafil, PF-00489791), there were 742 baseline patients and the mean baseline HbA1c was 7.70% (60.7 mmol/mol). Out of 6 studies with short half-life PDE5 inhibitors (sildenafil, avanafil), there were 311 baseline patients and the mean baseline HbA1c was 7.73% (61.0 mmol/mol). Out of all studies, the mean baseline HbA1c was 7.71% (60.8 mmol/mol) (Supplementary Fig. S1).

**Fig. 2:**
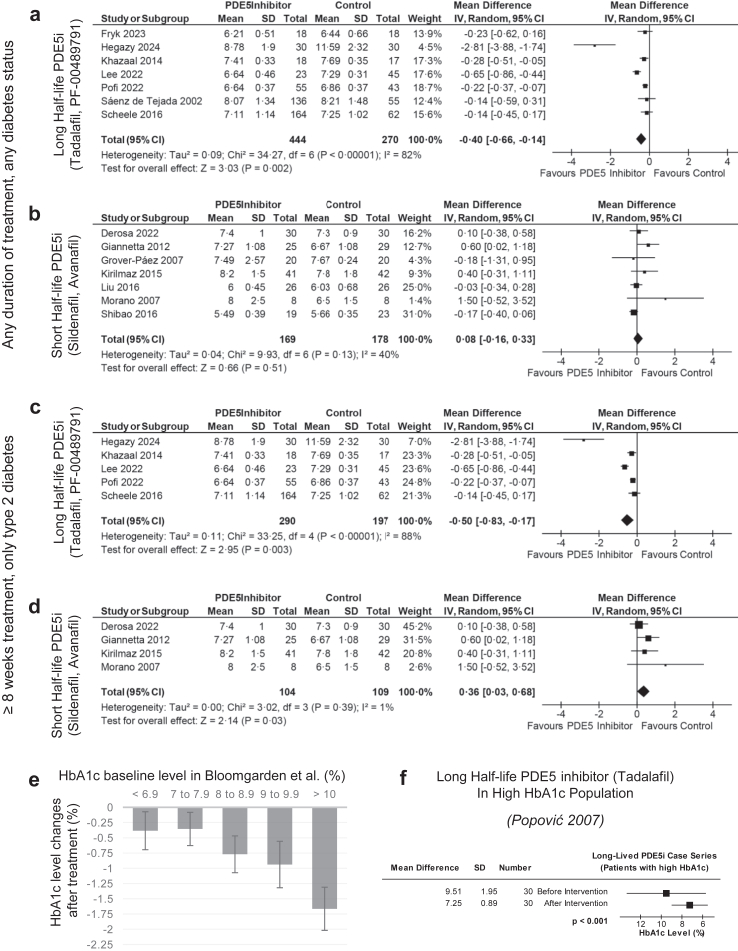
**Forest plot comparing and contextualising post-intervention HbA1c levels in the intervention group to that in the control group**. Haemoglobin A1c (HbA1c) data is reported in National Glycohemoglobin Standardization Program (NGSP) units (%). Analysis was done using a continuous inverse variance data type. Phosphodiesterase 5 (PDE5) inhibitors were investigated. **(a)** Long half-life PDE5 inhibitors (PDE5i) (tadalafil) have a statistically significantly lower HbA1c after treatment when compared to that of control. **(b)** Short half-life PDE5 inhibitors (sildenafil, avanafil) have an insignificantly lower HbA1c after treatment when compared to that of control. **(c)** Among studies with a duration of at least 8 weeks, long half-life PDE5 inhibitors (tadalafil) have a statistically significantly lower HbA1c after treatment when compared to that of control. **(d)** Among studies with a duration of at least 8 weeks, short half-life PDE5 inhibitors (sildenafil, avanafil) have an insignificantly lower HbA1c after treatment when compared to that of control. **(e)** Impact of Baseline HbA1c Levels on HbA1c Reduction with Oral Anti-Hyperglycaemic Medications. The figure illustrates the relationship between baseline HbA1c levels and the observed reduction in HbA1c achieved with oral anti-hyperglycaemic medications. Irrespective of drug class, the baseline glycaemic control significantly influences the overall magnitude of efficacy. Error bars represent standard deviations. **(f)** The only available long half-life PDE5 inhibitor study done in high HbA1c population near 10% shows far more significant drug effect as would be expected.

13 studies compared PDE5 inhibitor (PDE5i) treatment to control treatment (Fig. 2a and b). In 7 studies, mostly conducted among a population with already well controlled diabetes, long half-life PDE5 inhibitors (tadalafil, PF-00489791) demonstrated a statistically significant reduction in HbA1c levels compared to control with a mean difference of −0.40% (−4.9 mmol/mol, 95% confidence interval (CI) [−0.66%, −0.14%], 95% CI [−7.2 mmol/mol, −1.5 mmol/mol], p = 0.002, baseline HbA1c 7.70% [60.7 mmol/mol]) (Fig. 2a). Heterogeneity was considerably high with Chi^2^ p < 0.00001 and I^2^ = 83%, potentially due to the effect of baseline HbA1c levels on HbA1c level reduction. Considering this heterogeneity, sensitivity analysis shows that removing the outlier still retains statistical significance of the results. This HbA1c decrease is comparable to commercially available diabetes medications in populations, which have a −0.35% reduction with a similarly already low baseline HbA1c of 7–7.9%.^35^

Comparatively, the 6 studies with short half-life PDE5 inhibitors (sildenafil, avanafil) had an insignificant change in HbA1c levels with a mean difference of +0.18% (+2.0 mmol/mol, 95% CI [−0.09%, 0.45%], 95% CI [−1.0 mmol/mol, 4.9 mmol/mol], p = 0.19, baseline HbA1c 7.73% [59.2 mmol/mol]) (Fig. 2b). Heterogeneity was low with Chi^2^ p = 0.29 and I^2^ = 19%, suggesting that this insignificance is not due to uncertainty from heterogeneity.

As HbA1c concentrations reflect the average blood sugar level over the past 2–3 months^21^ and is most relevant to the treatment of type 2 diabetes and because of other methods to diagnose type 2 diabetes (such as through fasting plasma glucose), subgroup analysis was conducted, analysing 9 RCTs that were conducted for more than 8 weeks in only patients with type 2 diabetes as well as having a mean baseline HbA1c of at least 6.5% in order to assess outcomes with a higher quality dataset (Fig. 2c and d). Mostly conducted among a population with already well controlled diabetes, long half-life PDE5 inhibitors (tadalafil, PF-00489791) exhibited a statistically significant reduction in HbA1c with a mean difference of −0.50% (5.4 mmol/mol, 95% CI [−0.83%, −0.17%], 95% CI [−9.1 mmol/mol, −1.8 mmol/mol], p = 0.003) (Fig. 2c). As with the overall analysis, heterogeneity was considerably high with Chi^2^ p < 0.00001 and I^2^ = 88%, potentially due to the effect of baseline HbA1c levels on HbA1c level reduction. As investigated in sensitivity analysis, statistical significance is retained even if the outlier is removed.

However, short half-life PDE5 inhibitors (sildenafil, avanafil) exhibited a statistically significant increase in HbA1c with a mean difference of +0.36% (+3.9 mmol/mol, 95% CI [0.03%, 0.68%], 95% CI [0.3 mmol/mol, 7.5 mmol/mol], p = 0.03) (Fig. 2d). While it is surprising that this subgroup increases HbA1c (rather than decreasing HbA1c as in the case of long half-life PDE5 inhibitors), the p-value of 0.03 is close to the threshold of 0.05 and more trials to increase statistical power should be conducted. Mirroring overall analysis, heterogeneity was low with Chi^2^ p = 0.39 and I^2^ = 1%, suggesting that this is not due to uncertainty from heterogeneity.

Thus, the indirect mean difference between long half-life PDE5 inhibitors (e.g., tadalafil and PF-00489791) and short half-life PDE5 inhibitors (e.g., sildenafil and avanafil) stands at 0.58% (6.4 mmol/mol) in overall analysis and 0.86% (9.3 mmol/mol) in subgroup analysis, though this relationship would best be directly tested with RCTs. These results suggest long half-life PDE5 inhibitors as a potential drug choice. This disparity can be deemed clinically significant among patients with diabetes having an average baseline HbA1c of 7% (53 mmol/mol) levels under single drug treatment^35^ and will be investigated further.

The only available tadalafil RCT with high baseline HbA1c levels is Hegazy 2024 which shows far more significant drug effect at a baseline of near 10%, having a statistically significant HbA1c decrease with a mean difference of −2.81% (+30.7 mmol/mol, 95% CI [−3.88%, −1.74%], 95% CI [−42.5 mmol/mol, −19.0 mmol/mol], p = 0.03). We also found that the only available tadalafil non-RCT with high baseline A1c levels was a case series^36^ also having baseline A1c levels near 10%, with a mean difference of −2.26% (−24.7 mmol/mol, 95% CI [−3.03%, −1.49%], 95% CI [−33.1 mmol/mol, −16.3 mmol/mol], p < 0.00001) (Fig. 2f). Both of these trails align with expected effect of current diabetes medication (Fig. 2e).^35^ Therefore, this outcome underscores the importance for the clinical research community to explore the drug effects of long half-life PDE5 inhibitors in patients with higher HbA1c levels. Furthermore, with a mechanism that differs from many front-line diabetes medication, long half-life PDE5 inhibitors are a potential candidate for combination therapy.^37^ Hence, these results advocate for the inclusion of long half-life PDE5 inhibitors in patients with high HbA1c levels to optimise their conditions through combination therapy and a multi-faceted approach for maximal reduction of HbA1c levels.^38^

Secondary analysis of the effect of PDE5 inhibitors on various other related secondary parameters associated with type 2 diabetes among trials measuring HbA1c was conducted. The parameters tested were: HOMA-IR (Supplementary Fig. S26), BMI (Supplementary Fig. S27), Fasting glucose (Supplementary Fig. S28), 2 h post prandial glucose (Supplementary Fig. S29), Fasting insulin (Supplementary Fig. S30), Cholesterol (Supplementary Fig. S31), Triglycerides (Supplementary Fig. S32), HDL (Supplementary Fig. S33), LDL (Supplementary Fig. S34).

No secondary analysis reached statistical significance, apart from the 2 h post prandial glucose analysis. This analysis only included one trial—Hegazy 2024. All long half-life PDE5 inhibitor trials had mean differences in clinically beneficial directions (such as reducing HOMA-IR, reducing fasting glucose, and increasing HDL), with Hegazy 2024 showing stronger magnitudes than other trials. Meanwhile, short half-life PDE5 inhibitor trials had mean differences in mostly random directions.

Among both primary and secondary outcomes, significant heterogeneity can be seen. However, when considering baseline HbA1c, clinical trials with high baseline HbA1c levels (i.e., Hegazy 2024) visually are the most heterogenous compared to the other clinical trials with well-controlled HbA1c levels. Indeed, sensitivity analysis confirms that removing these outliers very significantly reduces heterogeneity. Therefore, this heterogeneity suggests that HbA1c is an important confounding factor to control for.

Risk of bias analysis shows that the trials range from a low risk of bias to some concerns (Supplementary Fig. S26). 3 long and 2 short half-life PDE5 inhibitor trials had low risk of bias. 4 long and 4 short half-life PDE5 inhibitor trials had some concerns of bias. Concerns arose mainly due to the absence of an analysis plan or protocol made available before clinical trial completion (Supplementary Figs. S26–S40). The number of studies was too low to accurately assess reporting bias (Supplementary Fig. S35).

The specific choices made for analysis methods does not significantly affect the results, as seen in sensitivity analysis. All sensitivity analyses resulted in statistically significant HbA1c decrease in long half-life PDE5 inhibitors and a statistically insignificant HbA1c change or a statistically significant HbA1c increase, as also seen in the overall and subgroup analysis. In all sensitivity analyses apart from three (which are described in further detail), all alternative analysis techniques did not result in statistically significant subgroup differences.

The choice to include trials with a duration of at least 8 weeks and with patients having type 2 diabetes as well as having a mean baseline HbA1c of at least 6.5% as secondary analysis does not significantly affect the results, and taking any combination of these three criteria maintains statistical significance (Supplementary Figs. S6–S13). Several studies included multiple dosages of PDE5 inhibitor or measured endpoint characteristics at multiple points in time. The choice to combine doses or only include the longer time period does not significantly affect the results and only causes minute changes (Supplementary Figs. S14–S16). Moreover, other possible choices for analysis, such as choosing to analyse trials by the specific pharmacological intervention or choosing to analyse trials with time-based medication administration, do not significantly affect the results (Supplementary Figs. S17 and S18). Segmenting studies by risk of bias shows that both trials with low risk of and some concerns of bias maintain the status quo (Supplementary Fig. S19). However, this is the only sensitivity analysis to show a statistically significant subgroup difference in long half-life PDE5 inhibitors, with p = 0.04. This is likely due to the outlier of Hegazy 2024; moreover, as 22 different sensitivity analyses were performed, we should expect to have significant p-values by chance, which is supported by the fact that the p-value is very close to the threshold of 0.05. Additionally, changing the assumption of random effects to one of fixed effects does not affect the statistical significance of the results in any of the figures (Supplementary Figs. S20 and S21). Finally, the choice to estimate data does not affect statistical significance of any results (Supplementary Fig. S22).

Outliers have a strong effect on the results. If subjective outliers are excluded, long half-life PDE5 inhibitors still have a statistically significant decrease in HbA1c, although not as pronounced (Supplementary Figs. S23 and S24). Furthermore, short half-life PDE5 inhibitors have a statistically insignificant effect on HbA1c levels, even in the subgroup analysis. As expected, excluding outliers leads to statistically significant subgroup differences between the outlier and the rest of the data.

In sensitivity analysis investigating the choice to split trials by half-life shows that combining all trials causes statistical significance to be lost (Supplementary Fig. S25). However, as expected, this shows a very statistically significant subgroup difference between the half-life, with p = 0.002, suggesting that half-life is a reasonable distinction to make.

## Discussion

We investigate an intriguing case of drug repurposing of PDE5 inhibitors (PDE5i), traditionally used for treating erectile dysfunction and pulmonary arterial hypertension, for the novel purpose of lowering HbA1c associated with type 2 diabetes. We compare long half-life PDE5 inhibitors to short half-life PDE5 inhibitors on the basis of increasing efficacy and medication compliance. In synthesising data from 13 clinical trials and 1083 baseline patients, our findings suggest a highly statistically significant reduction in HbA1c levels after long half-life PDE5 inhibitors (tadalafil, PF-00489791) compared to that after control (p = 0.002); meanwhile, short half-life PDE5 inhibitors (sildenafil, avanafil) show no significant difference in overall analysis and shows weakly statistically significant increase in HbA1c levels in subgroup analysis (p = 0.03). At the baseline HbA1c levels seen, long half-life PDE5 inhibitors show comparable HbA1c reduction to the five major classes of oral anti-hyperglycaemic agents.^35^ This result synthesises new results atop a previous meta-analysis that also found no significant difference in HbA1c after short half-life PDE5 inhibitor treatment, but was unable to investigate long half-life PDE5 inhibitor treatment.^16^

Certainly, the difference in effectiveness of diabetes medication among various baseline levels of HbA1c has been reported.^35^ A previous meta-regression analysis of 61 clinical trials evaluating the efficacy of the five major classes of oral diabetic medication demonstrated a strong direct correlation between baseline HbA1c level and the decrease in HbA1c induced (R^2^ = 0.18, F = 21.20, p < 0.0001) and can be compared to the results of mean difference (Fig. 2e).^35^ The average HbA1c baseline of the participants of the RCTs with an intervention of long half-life PDE5 inhibitor is 7.48% (58.3 mmol/mol) and showed an HbA1c mean difference of −0.32%, which is highly comparable to the drug effects of current diabetic medication that show a simple average of −0.35% (−3.5 mmol/mol) HbA1c mean difference at baseline of 7%–7.9% (Fig. 2e).^35^ Thus, the magnitude of HbA1c mean difference may be small because of the low baseline HbA1c approaching nondiabetic levels of 6.5% (47.5 mmol/mol).^1^

There are limitations on the results of this statistical analysis. HbA1c was used in many studies to exclude patients with uncontrolled diabetes, which may have minimised the observed effects (Fig. 2a–d); however, when considering studies with participants with high HbA1c, much greater effects were observed (Fig. 2f). Further meta-analyses may benefit from meta-regression on baseline HbA1c once more data representing high baseline HbA1c levels is available to better determine clinical and practical significance. Additionally, some trials were shorter than the 2–3-month period necessary for HbA1c changes, potentially biasing the results toward the null hypothesis. Future clinical trials should extend beyond three months to properly evaluate the effects on HbA1c. Finally, secondary analyses were limited by lack of data and study design. Future trials may benefit measuring other outcomes associated with diabetes, such as HOMA-IR.

There is a growing interest in exploring diverse drug classes that utilise different mechanisms to lower HbA1c, particularly in combination therapies.^37^ The distinct mechanisms of PDE5 inhibitors suggest they could be a promising addition to existing diabetes treatments, especially for patients with high baseline HbA1c levels.^38^ Further investigation into long half-life PDE5 inhibitors in patients with high HbA1c levels could yield valuable insights for combination therapy.

Meta-analysis has found that 66.3% of patients with type 2 diabetes have erectile dysfunction.^39^ Given this overlap, PDE5 inhibitors could serve a dual purpose in these patients. Although high medication costs have been a barrier to PDE5 adherence,^14^ the availability of low-cost options, such as those from CostPlus Drug Company or GoodRx, may address this concern.^40^

Unlike erectile dysfunction, which can be managed with on-demand treatment, diabetes requires continuous therapy. Therefore, further research is warranted to optimise the pharmacokinetics of long half-life PDE5 inhibitors (e.g., dose, frequency, extended-release formulations) for HbA1c reduction.^18^

While we present a case of drug repurposing for PDE5 inhibitors, this is suggestive of future consideration of half-life in other cases of drug repurposing. The quintessential use for PDE5 inhibitors for erectile dysfunction is, in itself, a case of drug repurposing of research on medications for coronary heart disease.^41^ Accordingly, the alternative use of PDE5 inhibitors is also being explored for other purposes. For instance, studies suggest that PDE5 inhibitors may support a reduced risk of Alzheimer's disease.^42^ However, the drug effects on cognitive impairment are highly variable, possibly because these studies do not consider the effect of half-life, as seen in our results for diabetes control. Addressing simple half-life considerations in clinical trial design may help resolve discrepancies in our research findings even if it may lead to the uncovering of completely independent mechanisms. Regardless, the research community investigating alternative disease treatments with PDE5 inhibitors should take these factors into consideration.

Lastly, our findings suggest that future clinical trials on diabetes should account for PDE5 inhibitor use. In 79/93 (85%) of the trials we reviewed which would have otherwise been included in analysis, HbA1c was not measured as an endpoint, likely due to the assumption that PDE5 inhibitors have no effect on HbA1c (Supplementary Table S1). Our results challenge this assumption and indicate that HbA1c should be measured in all relevant trials, especially since endpoints like erectile dysfunction could be affected by diabetes severity and almost 1/4 of all patients with diabetes are prescribed PDE5 inhibitors.^8^

## Contributors

Conceptualization: JK.

Data curation: JK and KK.

Formal analysis: JK, RZ, LRK, and KK.

Funding acquisition: N/A.

Investigation: JK and KK.

Methodology: JK, RZ, and KK.

Project administration: RZ and KK.

Resources: KK.

Software: JK and KK.

Supervision: RZ and KK.

Validation: JK and KK.

Visualization: JK and KK.

Writing – original draft: JK.

Writing – review & editing: JK, RZ, LRK, and KK.

JK and KK have access to and verify the underlying study data. JK and KK are the guarantors of this work and, as such, have full access to all the data in the study and take responsibility for the integrity of the data and the accuracy of the data analysis.

JK and KK were responsible for the decision to submit the manuscript.

## Data sharing statement

All datasets generated during and/or analysed in the current study are available from the corresponding author upon reasonable request immediately following publication with no end date. Researchers who provide a sound proposal and will conduct analysis that achieves an approved proposal should be directed to KNKim@mednet.ucla.edu.

## Declaration of interests

The authors have no conflict of interest to disclose.
