# Supplementary material for: Effect of long and short half-life PDE5 inhibitors on HbA1c levels: a systematic review and meta-analysis
Source: eClinicalMedicine. 2024 Dec 31;80:103035. doi: 10.1016/j.eclinm.2024.103035 (PMC11751502; doi:10.1016/j.eclinm.2024.103035)
Supplement: Supplementary Figures and Tables [file mmc1.pdf]

Table of Contents

Supplementary Fig. 1: Graphical Abstract 3

Supplementary Fig. 2: Potential mechanism of PDE5 inhibitors 4

Supplementary Fig. 3: Structured Methods 5

Supplementary Fig. 4: Michaelis-Menten model of HbA1c change over time 13

Supplementary Fig. 5: Search Terms 14

Supplementary Fig. 6: Sensitivity analysis of meta-analysis containing all trials 15

Supplementary Fig. 7: Sensitivity analysis of meta-analysis containing trials with only participants with type 2 diabetes 17

Supplementary Fig. 8: Sensitivity analysis of meta-analysis containing only trials with a treatment period of at least 8 weeks 19

Supplementary Fig. 9: Sensitivity analysis of meta-analysis containing only trials with only participants with type 2 diabetes and with a treatment period of at least 8 weeks 21

Supplementary Fig. 10: Sensitivity analysis of meta-analysis containing only trials with participants with a mean baseline HbA1c of at least 6.5% 23

Supplementary Fig. 11: Sensitivity analysis of meta-analysis containing only trials with participants with type 2 diabetes and a mean baseline HbA1c of at least 6.5% 25

Supplementary Fig. 12: Sensitivity analysis of meta-analysis containing only trials with participants with a mean baseline HbA1c of at least 6.5% and with a treatment period of at least 8 weeks 27

Supplementary Fig. 13: Sensitivity analysis of meta-analysis containing only trials with only participants with type 2 diabetes and a mean baseline HbA1c of at least 6.5% and with a treatment period of at least 8 weeks 29

Supplementary Fig. 14: Sensitivity analysis of meta-analysis containing all trials, using lowest dosage data 31

Supplementary Fig. 15: Sensitivity analysis of meta-analysis containing all trials, using highest dosage data 33

Supplementary Fig. 16: Sensitivity analysis of meta-analysis containing all trials, using shorter time period data 35

Supplementary Fig. 17: Sensitivity analysis of meta-analysis grouped by intervention 37

Supplementary Fig. 18: Sensitivity analysis of meta-analysis containing only trials with consistent, time-based intervention administration 39

Supplementary Fig. 19: Sensitivity analysis of meta-analysis grouped by risk of bias 41

Supplementary Fig. 20: Sensitivity analysis of meta-analysis performed using a fixed-effects model 43

Supplementary Fig. 21: Sensitivity analysis of meta-analysis subgroup analysis performed using a fixed-effects model 45

Supplementary Fig. 22: Sensitivity analysis of meta-analysis containing only trials not requiring data estimation 47

Supplementary Fig. 23: Sensitivity analysis of meta-analysis excluding subjective outliers 49

Supplementary Fig. 24: Sensitivity analysis of meta-analysis subgroup analysis excluding subjective outliers 51

Supplementary Fig. 25: Sensitivity analysis of meta-analysis grouping PDE5 inhibitors by half-life 53

Supplementary Fig. 26: Secondary analysis on HOMA-IR 55

Supplementary Fig. 27: Secondary analysis on BMI 56

Supplementary Fig. 28: Secondary analysis on fasting glucose 57

Supplementary Fig. 29: Secondary analysis on 2 hour post prandial glucose 58

Supplementary Fig. 30: Secondary analysis on fasting insulin..59
Supplementary Fig. 31: Secondary analysis on cholesterol...60
Supplementary Fig. 32: Secondary analysis on triglycerides 61
Supplementary Fig. 33: Secondary analysis on HDL 62
Supplementary Fig. 34: Secondary analysis on LDL 63
Supplementary Fig. 35: Funnel plots..64
Supplementary Fig. 36: Risk of Bias Analysis Overview 67
Supplementary Fig. 37: Risk of Bias Baseline Characteristics Analysis...68
Supplementary Fig. 38: Risk of Bias Analysis (Hegazy 2024) 72
Supplementary Fig. 39: Risk of Bias Analysis (Fryk 2023) 81
Supplementary Fig. 40: Risk of Bias Analysis (Pofi 2022)..90
Supplementary Fig. 41: Risk of Bias Analysis (Lee 2022)...98
Supplementary Fig. 42: Risk of Bias Analysis (Derosa 2022) 106
Supplementary Fig. 43: Risk of Bias Analysis (Liu 2016) 114
Supplementary Fig. 44: Risk of Bias Analysis (Scheele 2016) 122
Supplementary Fig. 45: Risk of Bias Analysis (Kirilmaz 2015) 130
Supplementary Fig. 46: Risk of Bias Analysis (Khazaal 2014)..138
Supplementary Fig. 47: Risk of Bias Analysis (Giannetta 2012) 146
Supplementary Fig. 48: Risk of Bias Analysis (Morano 2007) 154
Supplementary Fig. 49: Risk of Bias Analysis (Grover-Páez 2007) 162
Supplementary Fig. 50: Risk of Bias Analysis (Sáenz de Tejada 2002)...170
Supplementary Table 1: List of Study Search Results...178
Supplementary References 179

Supplementary Fig. 1: Graphical Abstract

A visual representation of the abstract is provided.

Drug Repurposing for Diabetes: Decreasing HbA1c Levels with Long Half-life PDE5 Inhibitors for Potential Combination Therapy

Short Half-Life PDE5i

6 RCTs

n = 311

7·73% A1c

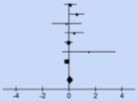

A1c Mean Difference:
+0·08% [-0·16, 0·33]

No significant difference

All PDE5 Inhibitors

13 RCTs, n = 1083

Alternative A1c lowering mechanism

Current Treatment

7·0-7·9% A1c

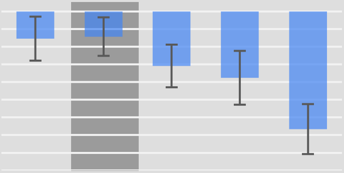

A1c Decrease:
-0·32%

Combination Therapy

Drug A + Drug B > Drug A, Drug B alone

Long Half-Life PDE5i

7 RCTs

n = 742

7·70% A1c

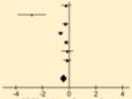

A1c Mean Difference:
-0·40% [-0·66, -0·14]

Comparable to standard treatment

Supplementary Fig. 2: Potential mechanism of PDE5 inhibitors

A potential mechanism for the action of Phosphodiesterase 5 (PDE5) inhibitors based on known interactions is provided. Rectangles represent gene products (such as protein), squrounds represent chemical compounds and other molecules, and unboxed labels represent general outcomes. A single pointed arrow represents stimulation, a single flat arrow represents inhibition, a double pointed arrow represents conversion, and a double arrow through a label represents transportation. (a) An in-depth representation of the mechanism is given, underscoring other pathway targets that also show appropriate changes in haemoglobin A1c (HbA1c) as well as the cause of glucose uptake. (b) A simplified representation representing major targets of the pathway, highlighting the bypassing of an insulin intermediate for decreasing HbA1c. Abbreviations: Phosphodiesterase 5 (PDE5), haemoglobin A1c (HbA1c), Phosphodiesterase 3 / Phosphodiesterase 4 / Phosphodiesterase 10 (PDE3/4/10), cyclic guanosine monophosphate (cGMP), 3',5'-cyclic adenosine monophosphate (cAMP), 5'-cyclic adenosine monophosphate (5’ cAMP), phosphoinositide 3-kinase / protein kinase B (PI3K/AKT), soluble guanylyl cyclase (sGC), calcium ions (Ca2+), glucose transporter type 4 (GLUT4), cyclic nucleotide gated channel alpha 1 / cyclic nucleotide gated channel beta 1 (CNG), deoxyribonucleic acid (DNA).

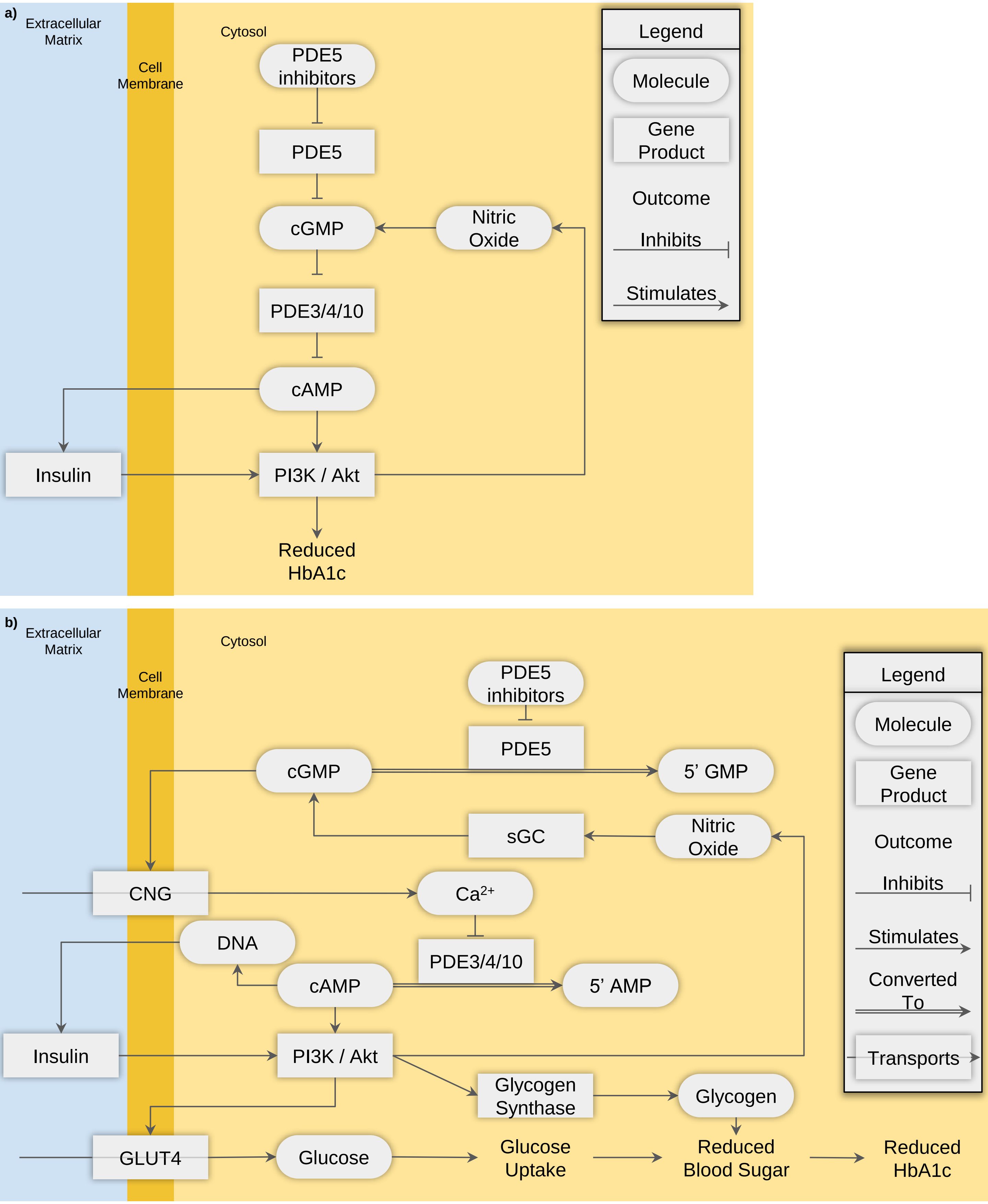
a)
Extracellular Matrix
Cell Membrane
Cytosol
PDE5 inhibitors
PDE5
cGMP
Nitric Oxide
PDE3/4/10
cAMP
Insulin
PI3K / Akt
Reduced HbA1c
Legend
Molecule
Gene Product
Outcome
Inhibits
Stimulates
b)
Extracellular Matrix
Cell Membrane
Cytosol
PDE5 inhibitors
PDE5
cGMP
5' GMP
sGC
Nitric Oxide
CNG
Ca2+
DNA
PDE3/4/10
cAMP
5' AMP
Insulin
PI3K / Akt
Glycogen Synthase
Glycogen
GLUT4
Glucose
Glucose Uptake
Reduced Blood Sugar
Reduced HbA1c
Legend
Molecule
Gene Product
Outcome
Inhibits
Stimulates
Converted To
Transports

Supplementary Fig. 3: Structured Methods

A systematic review with meta-analysis was conducted. The study adhered to Cochrane and PRISMA guidelines.[1,2] Statistical analysis, confidence analysis, heterogeneity analysis, forest plot generation, sensitivity analysis, and funnel plot (reporting bias) analysis was done in RevMan 5.4.1 and in RevMan Web (London, UK). RevMan forest plots were enhanced using Adobe Illustrator (San Jose, California, USA). The protocol was unpublished. The meta-analysis is registered on Research Registry (reviewregistry1733) and is available online (https://www.researchregistry.com/browse-the-registry#registryofsystematicreviewsmeta-analyses/registryofsystematicreviewsmeta-analysesdetails/65519717f8cb970026361b5f/).

Types of studies

We included only randomized controlled trials (RCTs). Other trial design types, such as case-control studies, observational studies, or case reports were excluded.

Types of participants

We included studies performed with participants:

- who are human
- with mean baseline HbA1c of at least 6%
    - to only include those with elevated HbA1c levels but not exclude those diagnosed with type 2 diabetes via other accepted methods such as fasting glucose
- regardless of erectile dysfunction or pulmonary arterial hypertension status
- regardless of gender or sex
- regardless of location

Types of interventions

We included studies with the following interventions:

- Any dosage of chronic PDE5 inhibitor treatment
- Treatment for at least 4 weeks
- Control / placebo

We excluded studies and participant populations for which a different intervention was adjusted as a variable in combination therapy with PDE5 inhibitor.

We chose to allow trials as short as 4 weeks because this would be sufficient to meaningfully reflect a drop in HbA1c, even if underestimated. Indeed, Michaelis-Menten models of HbA1c predict that a medication should have 34.2% of its maximal effect by 30 days, 56.6% by 60 days, and 71.4% by 90 days, so the mean difference at 30 days

is approximately half that at 90 days (**Supplementary Fig. 3**). Subgroup analysis on longer trial lengths is performed to avoid underestimation of results.

Types of outcome measures

Primary outcomes:

- Haemoglobin A1c (HbA1c)
    - No restrictions on measurement technique were placed; both NGSP and IFCC measurements were included
    - Must be measured and reported in both experimental and control groups

Secondary analysis:

- Homeostatic model assessment – insulin resistance (HOMA-IR)
- Body mass index (BMI)
- Fasting glucose
- 2 hour post prandial glucose
- Fasting insulin
- Cholesterol
- Triglycerides
- High-density lipoprotein (HDL)
- Low-density lipoprotein (LDL)
    - Due to protocol design, data for all secondary analysis was only collected from studies also reporting HbA1c.

Adverse event assessments were not performed due to well-established adverse event data.

Outcome data was collected from the following timepoints, where applicable:

- Endpoint data
- Change-from-baseline data and baseline data

Search methods for identification of studies

In total, 6 databases and registries were searched (**Figure 2**). The search terms used are provided (**Supplementary Fig. 4**).

The following databases of citations and abstracts were searched from inception to September 28, 2024:

- MEDLINE (PubMed)
- Elsevier Embase
- Cochrane Central Register of Controlled Trials (Cochrane CENTRAL)

The following full-text databases were searched from inception to September 28, 2024:

- PMC (PubMed Central) Medline

The following trial registries were search from inception to September 28, 2024:

- ClinicalTrials.gov by the United States National Library of Medicine
- World Health Organization International Clinical Trials Registry Platform (WHO ICTRP)

Citation matching was also performed where relevant. All search methods were completed without restriction on language. Where necessary, articles were translated using Google Translate (Mountain View, CA) or DeepL Translator (Cologne, Germany). Grey literature sources were not identified, and study authors were not contacted.

Selection of studies

All databases were independently searched by two review authors (both JK and KK) and any disagreements were resolved through discussion. If necessary, a third person would have been involved for resolution. Due to the routine nature of HbA1c level reporting, many studies did not report HbA1c data in abstracts or article metadata, so all studies were examined and analysed by their full text where possible. After search results were imported into Google Sheets, duplicates were automatically removed if any of the following ID numbers (as reported by searched databases) were duplicated:

- Embase identification number (PUI)
- PubMed ID (PMID)
- PubMed Central ID (PMC ID)
- Cochrane CENTRAL ID
- ClinicalTrials.gov ID
- Cumulative Index to Nursing and Allied Health Literature ID (CINAHL ID)
- WHO ICTRP ID
- Digital Object Identifier (DOI)

Data extraction and management

All data were extracted and analysed by both JK and KK until agreement. Summary estimates were retrieved. For main analysis, data parameters retrieved were number of patients, specific PDE5 inhibitor used in intervention, period of treatment, diabetes status, and outcome data. For risk of bias analysis, all baseline statistics were also extracted. For study characterization, the year range of recruitment, trial design, blinding type, and country of recruitment were also extracted. Data was extracted from article text, tables, figures, or supplementary data where applicable. Microsoft Office 365 (Redmond, Washington, USA) and Python 3.12 (Wilmington, Delaware, USA) were used to assist with data extraction and importation to Google Sheets. The Google Docs Editors suite (Mountain View, California, USA) was utilized to process and tabulate results. Data was extracted to Google Sheets and verified for agreement before transfer into RevMan 5.4.1 and to RevMan Web (London, UK).

Assessment of risk of bias in included studies

Risk of bias was independently assessed by both JK and KK, with cases of discordance resolved through discussion. Risk of bias analysis for quality of data was completed using the Risk of Bias 2.0 (RoB 2.0) tool (**Supplementary Fig. 35-49**). The appropriate RoB 2.0 tool was utilized according to the study design, specifically for parallel trials and crossover trials.

Following the RoB 2.0 tool, the following 5 domains were assessed using three measures (low risk, some concerns, or high risk of bias) to assess the effect of assignment to intervention (the 'intention-to-treat' effect) using the appropriate tool for the study design:

- Domain 1: Risk of bias arising from the randomization process
- Domain 2: Risk of bias due to deviations from the intended interventions
- Domain 3: Risk of bias due to missing outcome data
- Domain 4: Risk of bias in measurement of the outcome
- Domain 5: Risk of bias in selection of the reported result

Measures of treatment effect

We calculated mean differences (MDs) and 95% confidence intervals (CIs). A continuous inverse variance data type was used comparing intervention to control.

Unit of analysis issues

In our analysis, the individual participant was the unit of measurement.

Studies with multiple treatment groups

Where multiple dosages or multiple time periods were present, doses were combined and the longer time period were chosen. This is to avoid double-counting participants. The impact of these choices are investigated in the sensitivity analysis (**Supplementary Fig. 5-24**).

Dealing with missing data

Data normalization was performed in accordance with Cochrane guidelines or by using normal distribution statistics where applicable. Due to prevalent omission of standard deviations of change-from-baseline, all change-of-baseline statistics were converted to post-intervention statistics. Under Cochrane guidelines, medians and quartiles were converted to mean and standard deviation estimates using normal distributions. Where applicable, mean and standard deviation data were estimated from percentile measures and from baseline standard deviation. The impact of allowing data estimation is investigated in sensitivity analysis (**Supplementary Fig. 21**). Correlation imputation of change-from-baseline standard deviations was not used. IFCC units of mmol/mol were converted to the clinically familiar NGSP units of % using well-established formulae.[3] Therefore, all data represents absolute percentage points, not relative percentages.

Statistical heterogeneity

As per Cochrane guidelines, visual inspection of forest plots as well as Chi^2 and more importantly I^2 statistics were used to assess heterogeneity, with significance level of 0·1 for Chi^2 (accounting for low Chi^2 power in small sample sizes) and the following interpretations for I^2:

- 0% to 40%: might not be important
- 30% to 60%: may represent moderate heterogeneity
- 50% to 90%: may represent substantial heterogeneity
- 75% to 100%: considerable heterogeneity

Assessment of reporting biases

We planned to assess reporting bias using funnel plots. However, we had an insufficient number of studies (less than 10) for each treatment classification, so funnel plot analysis is not included. Nevertheless, funnel plots were generated for reference for future meta-analyses **(Supplementary Fig. 34)**.

Data synthesis

We used a random-effects model to partially account for potential heterogeneity in factors such as medication dosage, medication intervals, or population differences.

The stratification of studies on the bases of administration of short and long half-life inhibitors depended on the potential requirement for medication administration frequency to exceed once daily, as based on half-life. For the four PDE5 inhibitors approved by the FDA in the United States, this would place avanafil (4 hr), sildenafil (4 hr), and vardenafil (4 hr) as a short half-life PDE5 inhibitor and tadalafil (17·5 hr) as a long half-life inhibitor.[4] For other available PDE5 inhibitors not approved by the FDA but could be available especially in other sovereign states, this would place lodenafil (2 hr) and mirodenafil (2·5 hr) as a short half-life PDE5 inhibitor and udenafil (12 hr) as a long half-life PDE5 inhibitor. Other PDE5 inhibitors which may be found during search would be classified according to similar guidelines.

Subgroup analysis and investigation of heterogeneity

Because HbA1c concentrations reflect the average blood sugar level over the past 2-3 months[5] and is most relevant to the treatment of type 2 diabetes, and because of other methods to diagnose type 2 diabetes (such as through fasting plasma glucose), subgroup analysis was done, only including trials which lasted at least 2 month and exclusively recruited type 2 diabetes participants as well as having a mean baseline HbA1c of at least 6.5%. The main analysis including all trials is still relevant, however, as only trials with elevated HbA1c levels were included, and a short duration likely has the effect of underestimating any effect on HbA1c.

Sensitivity analysis

In order to test the robustness of the results found, the following sensitivity analyses were performed:

- Impact of choosing to report data in NGSP units of %: results reported in IFCC units of mmol/mol were generated, although the conversion is linear and would only result in differences due to rounding error (**Supplementary Fig. 5-23 c,d**)
- Impact of choosing a subgroup of studies lasting at least 8 weeks and with only participants with type 2 diabetes as well as having a mean baseline HbA1c of at least 6.5%: results under subgroups with any combination of these three criteria were generated (**Supplementary Fig. 5-12**)
- Impact of choosing to include all participants regardless of dosage: results under only including participant arms receiving the lower/higher dosage were generated (**Supplementary Fig. 13-14**)
- Impact of choosing to only include data from the longer time period: results under only including data from the shorter time period were generated (**Supplementary Fig. 15**)
- Impact of choosing to group trials by half-life of PDE5 inhibitors: results under grouping trials by the medication administered were generated (**Supplementary Fig. 16**)
- Impact of choosing to include all trials regardless of medication scheduling: results under only including trials with consistent, time-based intervention administration were generated (**Supplementary Fig. 17**)
- Impact of choosing to include all studies regardless of overall risk of bias: results under grouping trials by assessed risk of bias were generated (**Supplementary Fig. 18**)
- Impact of choosing a random-effects model: results under a fixed-effects model were generated (**Supplementary Fig. 18-19**)
- Impact of allowing data estimation: results under subgroups allowing only studies reporting data in methods not needing data estimation (such as by using change-from-baseline statistics or by using median and quartile measures) were generated (**Supplementary Fig. 20**)

- Impact of subjective outliers: results under excluding subjective outliers were generated (**Supplementary Fig. 21-22**)
- Impact of grouping PDE5 inhibitors by half-life: results under combining all data were generated (**Supplementary Fig. 23**)

Differences between protocol and review

Types of interventions: we originally planned to allow any duration of treatment to be counted. However, during data collection, we did not anticipate a trial which fit all criteria but ran treatment for an extremely short 12 hours, which is an unreasonable timeframe to expect a change in HbA1c. We put a minimum duration of treatment to avoid including errant trials like this. Moreover, we originally planned to only include trials testing tadalafil. To increase generalizability of analysis, we changed this to include all PDE5 inhibitors.

Types of outcome measures: we originally planned to only conduct the included primary analysis. To increase clinical significance of results, we also performed the included secondary analysis.

Search methods for identification of studies: we originally planned to search only PubMed and PMC Medline. To increase quality of data, we increased the databases searched to also include Embase, Cochrane CENTRAL, ClinicalTrials.gov, and the WHO ICTRP.

Assessment of risk of bias in included studies: GRADE analysis was initially planned, but was not feasible due to the limited number of trials.

Data synthesis: we originally planned to use a fixed-effects model. Accounting for significant heterogeneity, we changed this to a random-effects model to help account for the uncertainty potentially caused by baseline HbA1c differences.

Sensitivity analysis: we originally did not plan to run sensitivity analysis on subjective outliers. After seeing the magnitude of some outliers, we included the relevant sensitivity analysis. We also originally planned to run sensitivity analysis on the type of diabetes. Due to absence of such results, this sensitivity analysis could not be run. We also originally planned to run sensitivity analysis on sex and/or gender, but were unable to because of lack of data.

Summary of findings and assessment of the certainty of the evidence

Two authors (JK and KK) independently extracted and analysed data used the Risk of Bias 2.0 tool to assess the certainty of the evidence for the following outcome:

- HbA1c

Role of the funding source
There was no funding source for this study.

Supplementary Fig. 4: Michaelis-Menten model of HbA1c change over time

A Michaelis-Menten model of HbA1c change over time was produced to investigate the predicted effect of a medication, where a person with a steady HbA1c of 8% takes a medication that immediately drops their glucose to a level such that, as time goes to infinity, they would eventually have an HbA1c of 7%.[5] Parameters used, as experimentally determined, were g=141 mg/dL, K = 0.000533 dL/mg A1c_0 (%), $A1c_0$ = 8%, k_{age} = 0.0129 day^{-1}, k_{gly} = 0.00000728 dL mg^{-1} day^{-1}.

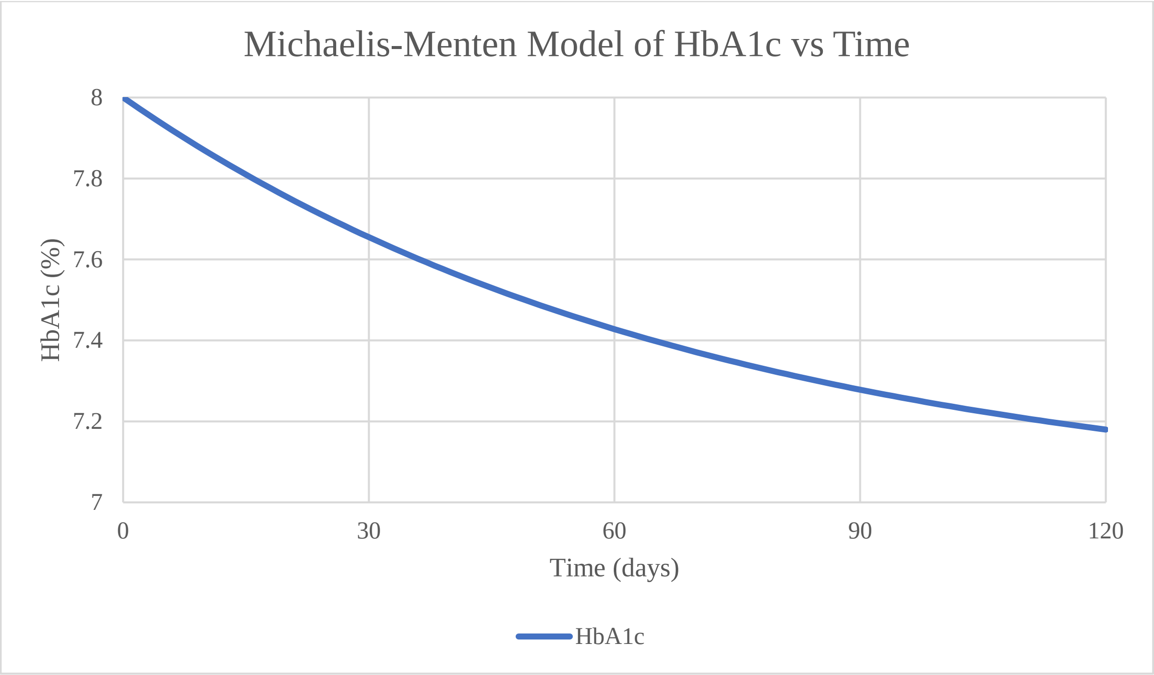

.

Supplementary Fig. 5: Search Terms

The following search term was used based on MeSH terms, pharmacological chemoinformatics, and terminology capturing:

(("PDE5 inhibitor") OR ("PDE-5 Inhibitor") OR ("PDE 5 Inhibitor") OR ("PDE5 Inhibitors") OR ("PDE-5 Inhibitors") OR ("PDE 5 Inhibitors") OR ("Phosphodiesterase 5 Inhibitors [Pharmacological Action]") OR ("Aildenafil") OR ("methisosildenafil") OR ("mirodenafil") OR ("Sildenafil") OR ("NCX-911") OR ("NCX 911") OR ("NCX911") OR ("Revatio") OR ("UK 92480-10") OR ("UK 92480 10") OR ("UK 9248010") OR ("UK-92,480-10") OR ("UK 92,480 10") OR ("Homosildenafil") OR ("methyl-sildenafil") OR ("Hydroxyhomosildenafil") OR ("Lodenafil") OR ("Viagra") OR ("Acetildenafil") OR ("hongdenafil") OR ("Desmethylsildenafil") OR ("T 1032") OR ("T-1032") OR ("Tadalafil") OR ("IC351") OR ("IC-351") OR ("IC 351") OR ("Cialis") OR ("Udenafil") OR ("Zidena") OR ("DA 8159") OR ("DA-8159") OR ("Vardenafil") OR ("Levitra")) AND (("Glycated Hemoglobin") OR ("Hemoglobin, Glycated") OR ("Hemoglobin, Glycosylated") OR ("Glycosylated Hemoglobin") OR ("Glycated Hemoglobins") OR ("Hemoglobins, Glycated") OR ("Glycohemoglobin") OR ("Glycohemoglobins") OR ("Glycated Hemoglobin A1c") OR ("Hemoglobin A1c, Glycated") OR ("Glycosylated Hemoglobin A1c") OR ("Hemoglobin A1c, Glycosylated") OR ("Hb A1a-2") OR ("Hemoglobin, Glycated A1a-2") OR ("A1a-2 Hemoglobin, Glycated") OR ("Glycated A1a-2 Hemoglobin") OR ("Hemoglobin, Glycated A1a 2") OR ("Glycated Hemoglobin A") OR ("Hemoglobin A, Glycated") OR ("Hb A1a+b") OR ("Hb A1c") OR ("HbA1") OR ("Glycosylated Hemoglobin A") OR ("Hemoglobin A, Glycosylated") OR ("Hb A1") OR ("Glycohemoglobin A") OR ("Hemoglobin A(1)") OR ("Hemoglobin, Glycosylated A1a-1") OR ("A1a-1 Hemoglobin, Glycosylated") OR ("Glycosylated A1a-1 Hemoglobin") OR ("Hemoglobin, Glycosylated A1a 1") OR ("Hb A1a-1") OR ("Hemoglobin, Glycated A1b") OR ("A1b Hemoglobin, Glycated") OR ("Glycated A1b Hemoglobin") OR ("Hb A1b") OR ("Hemoglobin, Glycosylated A1b") OR ("A1b Hemoglobin, Glycosylated") OR ("Glycosylated A1b Hemoglobin") OR ("Fructated Hemoglobins") OR ("Hemoglobins, Fructated") OR ("HbA1c"))

Supplementary Fig. 6: Sensitivity analysis of meta-analysis containing all trials

Sensitivity analysis was performed on a potential decision to include all trials, as well as on a potential decision to represent data in IFCC units (mmol/mol) or NGSP units (%). The following conditions were used for each meta-analysis: a) long half-life PDE5 inhibitors using NGSP units; b) short half-life PDE5 inhibitors using NGSP units; c) long half-life PDE5 inhibitors using IFCC units; d) short half-life PDE5 inhibitors using IFCC units.

a)

	PDE5Inhibitor			Control				Mean Difference
**Study or Subgroup**	**Mean**	**SD**	**Total**	**Mean**	**SD**	**Total**	**Weight**	**IV, Random, 95% CI**
Fryk 2023	6·21	0·51	18	6·44	0·66	18	13·9%	-0·23 [-0·62, 0·16]
Hegazy 2024	8·78	1·9	30	11·59	2·32	30	4·5%	-2·81 [-3·88, -1·74]
Khazaal 2014	7·41	0·33	18	7·69	0·35	17	17·3%	-0·28 [-0·51, -0·05]
Lee 2022	6·64	0·46	23	7·29	0·31	45	17·6%	-0·65 [-0·86, -0·44]
Pofi 2022	6·64	0·37	55	6·86	0·37	43	18·7%	-0·22 [-0·37, -0·07]
Sáenz de Tejada 2002	8·07	1·34	136	8·21	1·48	55	12·4%	-0·14 [-0·59, 0·31]
Scheele 2016	7·11	1·14	164	7·25	1·02	62	15·6%	-0·14 [-0·45, 0·17]
**Total (95% CI)**			**444**			**270**	**100·0%**	**-0·40 [-0·66, -0·14]**

Heterogeneity: Tau² = 0·09; Chi² = 34·27, df = 6 (P < 0·00001); I² = 82%
Test for overall effect: Z = 3·03 (P = 0·002)

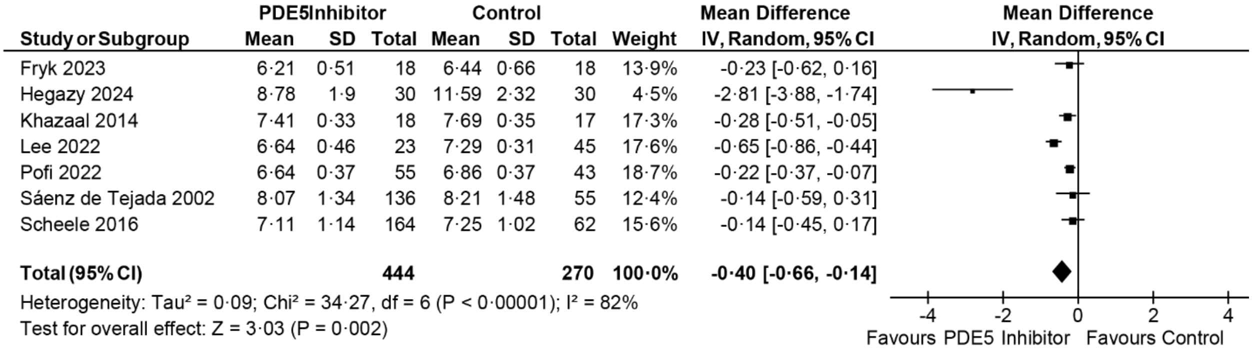

b)

	PDE5Inhibitor			Control				Mean Difference
**Study or Subgroup**	**Mean**	**SD**	**Total**	**Mean**	**SD**	**Total**	**Weight**	**IV, Random, 95% CI**
Derosa 2022	7·4	1	30	7·3	0·9	30	23·1%	0·10 [-0·38, 0·58]
Giannetta 2012	7·27	1·08	25	6·67	1·08	29	17·4%	0·60 [0·02, 1·18]
Grover-Páez 2007	7·49	2·57	20	7·67	0·24	20	5·3%	-0·18 [-1·31, 0·95]
Kirilmaz 2015	8·2	1·5	41	7·8	1·8	42	12·3%	0·40 [-0·31, 1·11]
Liu 2016	6	0·45	26	6·03	0·68	26	40·1%	-0·03 [-0·34, 0·28]
Morano 2007	8	2·5	8	6·5	1·5	8	1·7%	1·50 [-0·52, 3·52]
**Total (95% CI)**			**150**			**155**	**100·0%**	**0·18 [-0·09, 0·45]**

Heterogeneity: Tau² = 0·02; Chi² = 6·15, df = 5 (P = 0·29); I² = 19%
Test for overall effect: Z = 1·32 (P = 0·19)

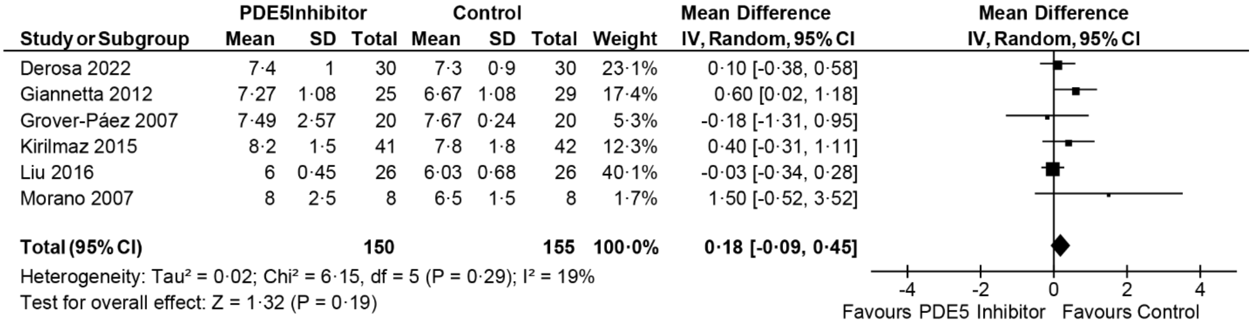

c)

	PDE5Inhibitor			Control				Mean Difference
**Study or Subgroup**	**Mean**	**SD**	**Total**	**Mean**	**SD**	**Total**	**Weight**	**IV, Random, 95% CI**
Fryk 2023	44·4	5·5	18	46·9	7·2	18	13·9%	-2·50 [-6·69, 1·69]
Hegazy 2024	72·5	20·8	30	103·2	25·4	30	4·6%	-30·70 [-42·45, -18·95]
Khazaal 2014	57·5	3·6	18	60·5	3·9	17	17·2%	-3·00 [-5·49, -0·51]
Lee 2022	49·1	5	23	56·2	3·4	45	17·6%	-7·10 [-9·37, -4·83]
Pofi 2022	49·1	4	55	51·4	4	43	18·7%	-2·30 [-3·90, -0·70]
Sáenz de Tejada 2002	64·7	14·7	136	66·3	16·1	55	12·5%	-1·60 [-6·52, 3·32]
Scheele 2016	54·2	12·5	164	55·7	11·1	62	15·6%	-1·50 [-4·86, 1·86]
**Total (95% CI)**			**444**			**270**	**100·0%**	**-4·38 [-7·23, -1·52]**

Heterogeneity: Tau² = 10·72; Chi² = 34·71, df = 6 (P < 0·00001); I² = 83%
Test for overall effect: Z = 3·00 (P = 0·003)

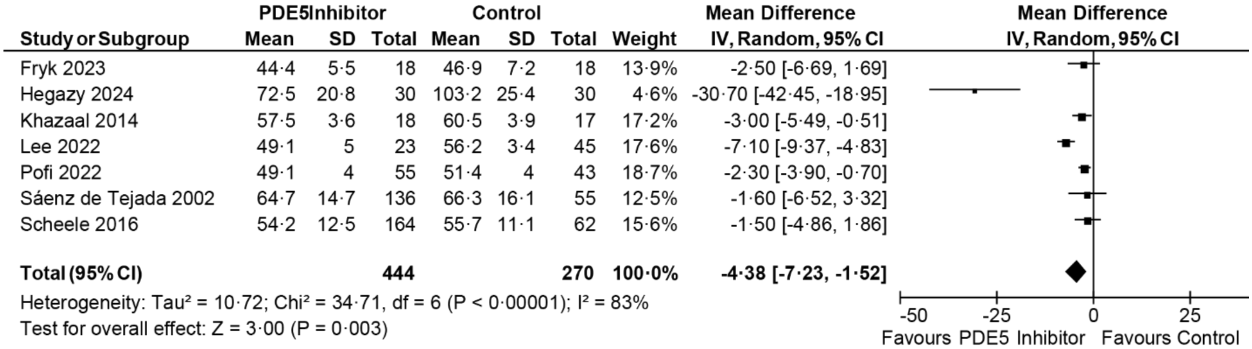

d)

Study or Subgroup	PDE5 Inhibitor Mean	SD	Total	Control Mean	SD	Total	Weight	Mean Difference IV, Random, 95% CI
Derosa 2022	57·4	10·9	30	56·3	9·8	30	23·1%	1·10 [-4·15, 6·35]
Giannetta 2012	56	11·8	25	49·4	11·8	29	17·4%	6·60 [0·29, 12·91]
Grover-Páez 2007	58·4	28·1	20	60·4	2·6	20	5·3%	-2·00 [-14·37, 10·37]
Kirilmaz 2015	66·1	16·4	41	61·8	19·7	42	12·3%	4·30 [-3·49, 12·09]
Liu 2016	42·1	4·9	26	42·4	7·4	26	40·2%	-0·30 [-3·71, 3·11]
Morano 2007	63·9	27·3	8	47·5	16·4	8	1·7%	16·40 [-5·67, 38·47]
**Total (95% CI)**			**150**			**155**	**100·0%**	**1·99 [-0·95, 4·93]**

Heterogeneity: Tau² = 2·57; Chi² = 6·15, df = 5 (P = 0·29); I² = 19%

Test for overall effect: Z = 1·32 (P = 0·19)

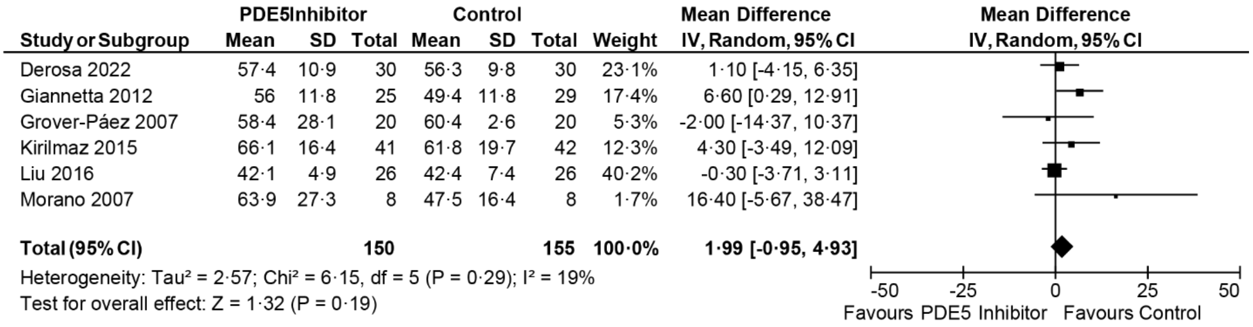

Supplementary Fig. 7: Sensitivity analysis of meta-analysis containing trials with only participants with type 2 diabetes

Sensitivity analysis was performed on a potential decision to include trials with only participants with type 2 diabetes, as well as on a potential decision to represent data in IFCC units (mmol/mol) or NGSP units (%). The following conditions were used for each meta-analysis: a) long half-life PDE5 inhibitors using NGSP units; b) short half-life PDE5 inhibitors using NGSP units; c) long half-life PDE5 inhibitors using IFCC units; d) short half-life PDE5 inhibitors using IFCC units.

a)

	PDE5Inhibitor			Control				Mean Difference
**Study or Subgroup**	**Mean**	**SD**	**Total**	**Mean**	**SD**	**Total**	**Weight**	**IV, Random, 95% CI**
**2·1·1 Trials With Only Patients Having Type 2 Diabetes**								
Fryk 2023	6·21	0·51	18	6·44	0·66	18	13·9%	-0·23 [-0·62, 0·16]
Hegazy 2024	8·78	1·9	30	11·59	2·32	30	4·5%	-2·81 [-3·88, -1·74]
Khazaal 2014	7·41	0·33	18	7·69	0·35	17	17·3%	-0·28 [-0·51, -0·05]
Lee 2022	6·64	0·46	23	7·29	0·31	45	17·6%	-0·65 [-0·86, -0·44]
Pofi 2022	6·64	0·37	55	6·86	0·37	43	18·7%	-0·22 [-0·37, -0·07]
Scheele 2016	7·11	1·14	164	7·25	1·02	62	15·6%	-0·14 [-0·45, 0·17]
**Subtotal (95% CI)**			**308**			**215**	**87·6%**	**-0·45 [-0·73, -0·16]**
Heterogeneity: Tau² = 0·10; Chi² = 33·57, df = 5 (P < 0·00001); I² = 85%								
Test for overall effect: Z = 3·04 (P = 0·002)								
**2·1·2 Other Trials**								
Sáenz de Tejada 2002	8·07	1·34	136	8·21	1·48	55	12·4%	-0·14 [-0·59, 0·31]
**Subtotal (95% CI)**			**136**			**55**	**12·4%**	**-0·14 [-0·59, 0·31]**
Heterogeneity: Not applicable								
Test for overall effect: Z = 0·61 (P = 0·54)								
**Total (95% CI)**			**444**			**270**	**100·0%**	**-0·40 [-0·66, -0·14]**
Heterogeneity: Tau² = 0·09; Chi² = 34·27, df = 6 (P < 0·00001); I² = 82%								
Test for overall effect: Z = 3·03 (P = 0·002)								
Test for subgroup differences: Chi² = 1·26, df = 1 (P = 0·26), I² = 20·5%								

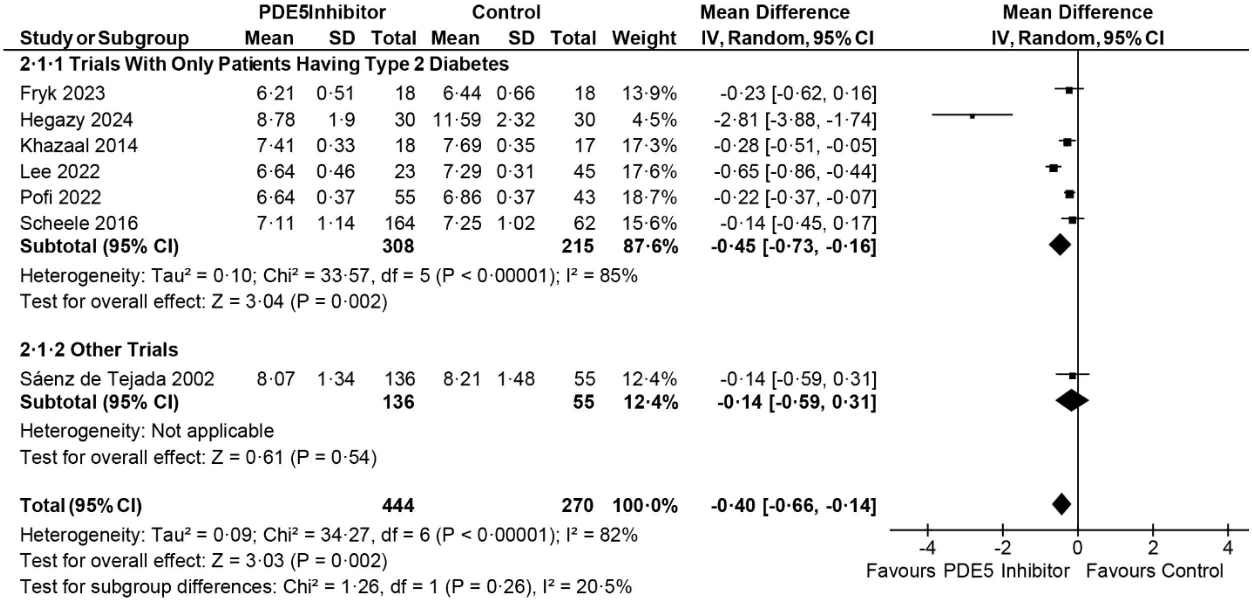

b)

	PDE5Inhibitor			Control				Mean Difference
**Study or Subgroup**	**Mean**	**SD**	**Total**	**Mean**	**SD**	**Total**	**Weight**	**IV, Random, 95% CI**
**2·2·1 Trials Meeting Subgroup Criteria**								
Derosa 2022	7·4	1	30	7·3	0·9	30	23·1%	0·10 [-0·38, 0·58]
Giannetta 2012	7·27	1·08	25	6·67	1·08	29	17·4%	0·60 [0·02, 1·18]
Grover-Páez 2007	7·49	2·57	20	7·67	0·24	20	5·3%	-0·18 [-1·31, 0·95]
Kirilmaz 2015	8·2	1·5	41	7·8	1·8	42	12·3%	0·40 [-0·31, 1·11]
Morano 2007	8	2·5	8	6·5	1·5	8	1·7%	1·50 [-0·52, 3·52]
**Subtotal (95% CI)**			**124**			**129**	**59·9%**	**0·31 [0·00, 0·63]**
Heterogeneity: Tau² = 0·00; Chi² = 3·81, df = 4 (P = 0·43); I² = 0%								
Test for overall effect: Z = 1·98 (P = 0·05)								
**2·2·2 Other Trials**								
Liu 2016	6	0·45	26	6·03	0·68	26	40·1%	-0·03 [-0·34, 0·28]
**Subtotal (95% CI)**			**26**			**26**	**40·1%**	**-0·03 [-0·34, 0·28]**
Heterogeneity: Not applicable								
Test for overall effect: Z = 0·19 (P = 0·85)								
**Total (95% CI)**			**150**			**155**	**100·0%**	**0·18 [-0·09, 0·45]**
Heterogeneity: Tau² = 0·02; Chi² = 6·15, df = 5 (P = 0·29); I² = 19%								
Test for overall effect: Z = 1·32 (P = 0·19)								
Test for subgroup differences: Chi² = 2·34, df = 1 (P = 0·13), I² = 57·2%								

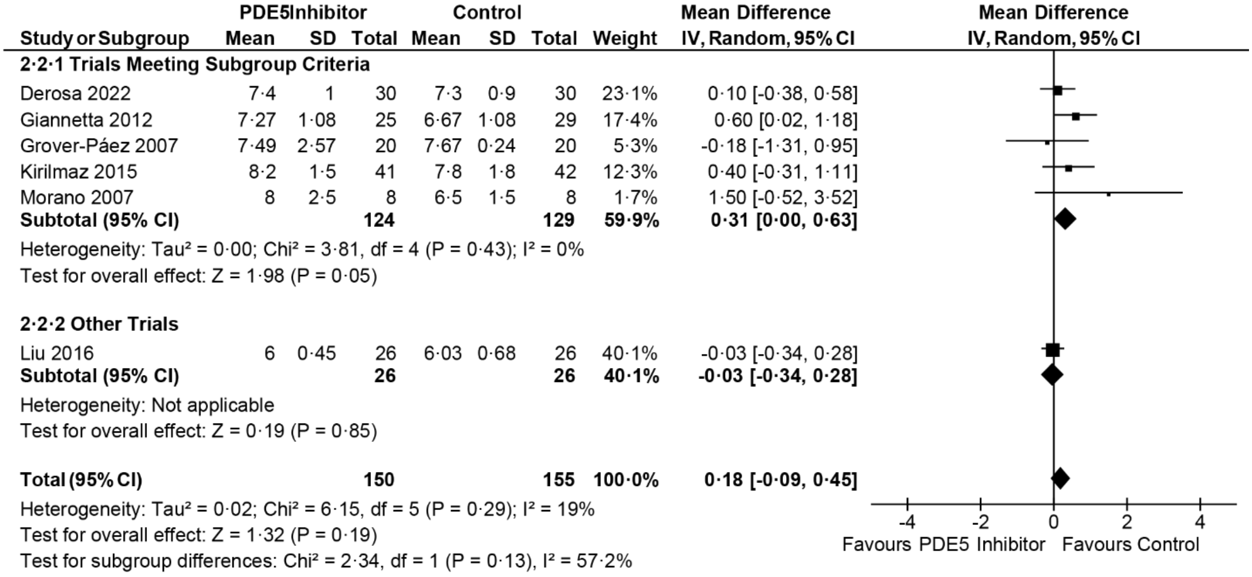

c)

Study or Subgroup	PDE5Inhibitor Mean	SD	Total	Control Mean	SD	Total	Weight	Mean Difference IV, Random, 95% CI
**2·3·1 Trials Meeting Subgroup Criteria**								
Fryk 2023	44·4	5·5	18	46·9	7·2	18	13·9%	-2·50 [-6·69, 1·69]
Hegazy 2024	72·5	20·8	30	103·2	25·4	30	4·6%	-30·70 [-42·45, -18·95]
Khazaal 2014	57·5	3·6	18	60·5	3·9	17	17·2%	-3·00 [-5·49, -0·51]
Lee 2022	49·1	5	23	56·2	3·4	45	17·6%	-7·10 [-9·37, -4·83]
Pofi 2022	49·1	4	55	51·4	4	43	18·7%	-2·30 [-3·90, -0·70]
Scheele 2016	54·2	12·5	164	55·7	11·1	62	15·6%	-1·50 [-4·86, 1·86]
**Subtotal (95% CI)**			**308**			**215**	**87·5%**	**-4·84 [-8·01, -1·68]**
Heterogeneity: Tau² = 11·72; Chi² = 34·09, df = 5 (P < 0·00001); I² = 85%								
Test for overall effect: Z = 3·00 (P = 0·003)								
**2·3·2 Other Trials**								
Sáenz de Tejada 2002	64·7	14·7	136	66·3	16·1	55	12·5%	-1·60 [-6·52, 3·32]
**Subtotal (95% CI)**			**136**			**55**	**12·5%**	**-1·60 [-6·52, 3·32]**
Heterogeneity: Not applicable								
Test for overall effect: Z = 0·64 (P = 0·52)								
**Total (95% CI)**			**444**			**270**	**100·0%**	**-4·38 [-7·23, -1·52]**
Heterogeneity: Tau² = 10·72; Chi² = 34·71, df = 6 (P < 0·00001); I² = 83%								
Test for overall effect: Z = 3·00 (P = 0·003)								
Test for subgroup differences: Chi² = 1·18, df = 1 (P = 0·28), I² = 15·2%								

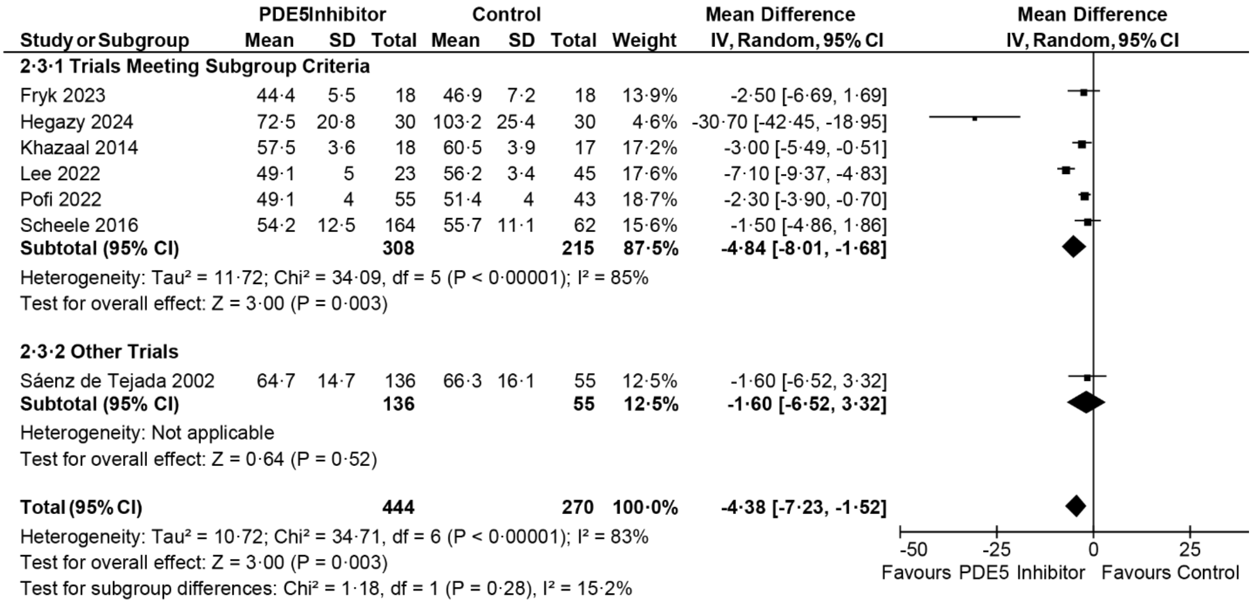

d)

Study or Subgroup	PDE5Inhibitor Mean	SD	Total	Control Mean	SD	Total	Weight	Mean Difference IV, Random, 95% CI
**2·4·1 Trials Meeting Subgroup Criteria**								
Derosa 2022	57·4	10·9	30	56·3	9·8	30	23·1%	1·10 [-4·15, 6·35]
Giannetta 2012	56	11·8	25	49·4	11·8	29	17·4%	6·60 [0·29, 12·91]
Grover-Páez 2007	58·4	28·1	20	60·4	2·6	20	5·3%	-2·00 [-14·37, 10·37]
Kirilmaz 2015	66·1	16·4	41	61·8	19·7	42	12·3%	4·30 [-3·49, 12·09]
Morano 2007	63·9	27·3	8	47·5	16·4	8	1·7%	16·40 [-5·67, 38·47]
**Subtotal (95% CI)**			**124**			**129**	**59·8%**	**3·43 [0·03, 6·83]**
Heterogeneity: Tau² = 0·00; Chi² = 3·84, df = 4 (P = 0·43); I² = 0%								
Test for overall effect: Z = 1·98 (P = 0·05)								
**2·4·2 Other Trials**								
Liu 2016	42·1	4·9	26	42·4	7·4	26	40·2%	-0·30 [-3·71, 3·11]
**Subtotal (95% CI)**			**26**			**26**	**40·2%**	**-0·30 [-3·71, 3·11]**
Heterogeneity: Not applicable								
Test for overall effect: Z = 0·17 (P = 0·86)								
**Total (95% CI)**			**150**			**155**	**100·0%**	**1·99 [-0·95, 4·93]**
Heterogeneity: Tau² = 2·57; Chi² = 6·15, df = 5 (P = 0·29); I² = 19%								
Test for overall effect: Z = 1·32 (P = 0·19)								
Test for subgroup differences: Chi² = 2·31, df = 1 (P = 0·13), I² = 56·7%								

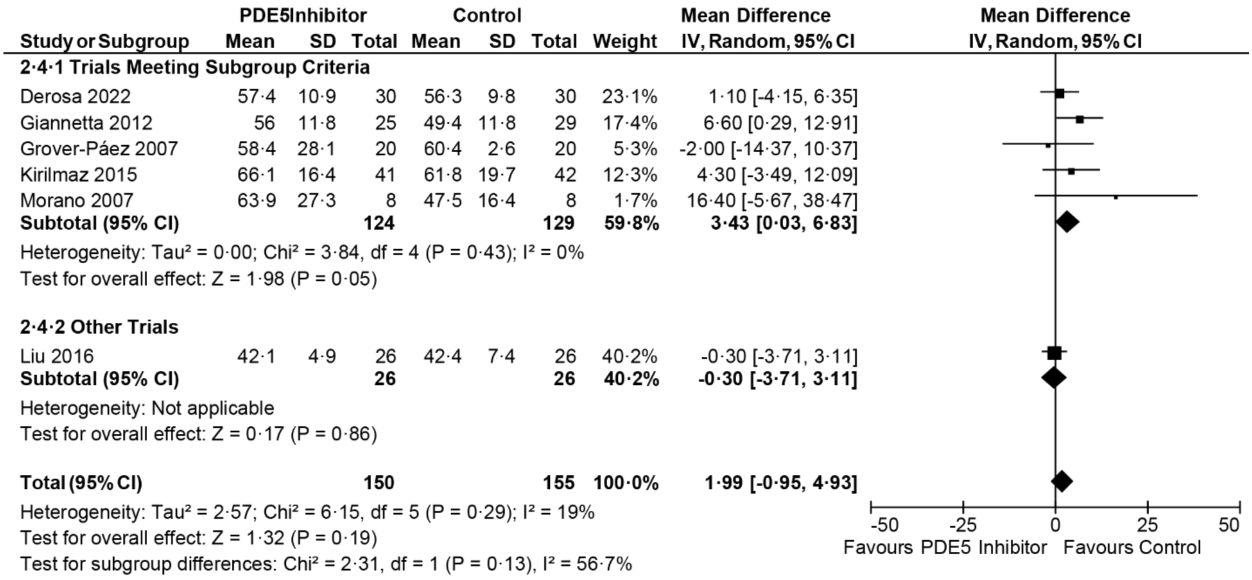

Supplementary Fig. 8: Sensitivity analysis of meta-analysis containing only trials with a treatment period of at least 8 weeks

Sensitivity analysis was performed on a potential decision to only include trials with a treatment period of at least 8 weeks, as well as on a potential decision to represent data in IFCC units (mmol/mol) or NGSP units (%). The following conditions were used for each meta-analysis: a) long half-life PDE5 inhibitors using NGSP units; b) short half-life PDE5 inhibitors using NGSP units; c) long half-life PDE5 inhibitors using IFCC units; d) short half-life PDE5 inhibitors using IFCC units.

a)

	PDE5Inhibitor			Control				Mean Difference
**Study or Subgroup**	**Mean**	**SD**	**Total**	**Mean**	**SD**	**Total**	**Weight**	**IV, Random, 95% CI**
**3·1·1 Trials Meeting Subgroup Criteria**								
Hegazy 2024	8·78	1·9	30	11·59	2·32	30	4·5%	-2·81 [-3·88, -1·74]
Khazaal 2014	7·41	0·33	18	7·69	0·35	17	17·3%	-0·28 [-0·51, -0·05]
Lee 2022	6·64	0·46	23	7·29	0·31	45	17·6%	-0·65 [-0·86, -0·44]
Pofi 2022	6·64	0·37	55	6·86	0·37	43	18·7%	-0·22 [-0·37, -0·07]
Sáenz de Tejada 2002	8·07	1·34	136	8·21	1·48	55	12·4%	-0·14 [-0·59, 0·31]
Scheele 2016	7·11	1·14	164	7·25	1·02	62	15·6%	-0·14 [-0·45, 0·17]
**Subtotal (95% CI)**			**426**			**252**	**86·1%**	**-0·44 [-0·74, -0·14]**
Heterogeneity: Tau² = 0·10; Chi² = 34·01, df = 5 (P < 0·00001); I² = 85%								
Test for overall effect: Z = 2·92 (P = 0·004)								
**3·1·2 Other Trials**								
Fryk 2023	6·21	0·51	18	6·44	0·66	18	13·9%	-0·23 [-0·62, 0·16]
**Subtotal (95% CI)**			**18**			**18**	**13·9%**	**-0·23 [-0·62, 0·16]**
Heterogeneity: Not applicable								
Test for overall effect: Z = 1·17 (P = 0·24)								
**Total (95% CI)**			**444**			**270**	**100·0%**	**-0·40 [-0·66, -0·14]**
Heterogeneity: Tau² = 0·09; Chi² = 34·27, df = 6 (P < 0·00001); I² = 82%								
Test for overall effect: Z = 3·03 (P = 0·002)								
Test for subgroup differences: Chi² = 0·72, df = 1 (P = 0·40), I² = 0%								

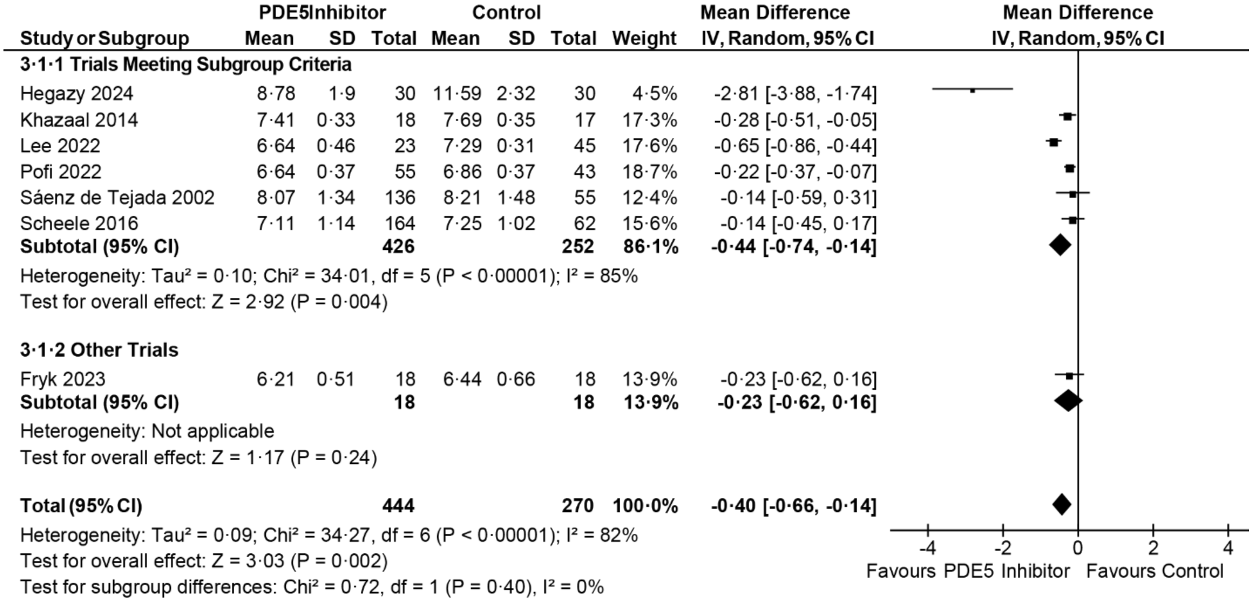

b)

	PDE5Inhibitor			Control				Mean Difference
**Study or Subgroup**	**Mean**	**SD**	**Total**	**Mean**	**SD**	**Total**	**Weight**	**IV, Random, 95% CI**
**3·2·1 Trials Meeting Subgroup Criteria**								
Derosa 2022	7·4	1	30	7·3	0·9	30	23·1%	0·10 [-0·38, 0·58]
Giannetta 2012	7·27	1·08	25	6·67	1·08	29	17·4%	0·60 [0·02, 1·18]
Kirilmaz 2015	8·2	1·5	41	7·8	1·8	42	12·3%	0·40 [-0·31, 1·11]
Liu 2016	6	0·45	26	6·03	0·68	26	40·1%	-0·03 [-0·34, 0·28]
Morano 2007	8	2·5	8	6·5	1·5	8	1·7%	1·50 [-0·52, 3·52]
**Subtotal (95% CI)**			**130**			**135**	**94·7%**	**0·22 [-0·08, 0·53]**
Heterogeneity: Tau² = 0·04; Chi² = 5·82, df = 4 (P = 0·21); I² = 31%								
Test for overall effect: Z = 1·42 (P = 0·16)								
**3·2·2 Other Trials**								
Grover-Páez 2007	7·49	2·57	20	7·67	0·24	20	5·3%	-0·18 [-1·31, 0·95]
**Subtotal (95% CI)**			**20**			**20**	**5·3%**	**-0·18 [-1·31, 0·95]**
Heterogeneity: Not applicable								
Test for overall effect: Z = 0·31 (P = 0·76)								
**Total (95% CI)**			**150**			**155**	**100·0%**	**0·18 [-0·09, 0·45]**
Heterogeneity: Tau² = 0·02; Chi² = 6·15, df = 5 (P = 0·29); I² = 19%								
Test for overall effect: Z = 1·32 (P = 0·19)								
Test for subgroup differences: Chi² = 0·45, df = 1 (P = 0·50), I² = 0%								

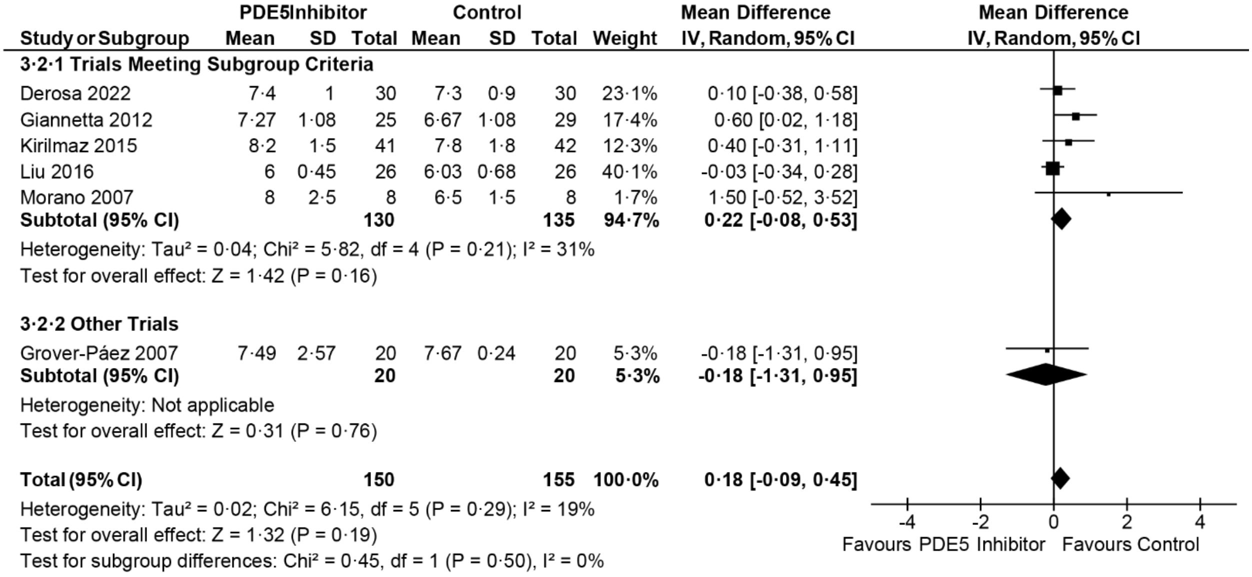

c)

Study or Subgroup	PDE5Inhibitor Mean	PDE5Inhibitor SD	PDE5Inhibitor Total	Control Mean	Control SD	Control Total	Weight	Mean Difference IV, Random, 95% CI
**3·3·1 Trials Meeting Subgroup Criteria**								
Hegazy 2024	72·5	20·8	30	103·2	25·4	30	4·6%	-30·70 [-42·45, -18·95]
Khazaal 2014	57·5	3·6	18	60·5	3·9	17	17·2%	-3·00 [-5·49, -0·51]
Lee 2022	49·1	5	23	56·2	3·4	45	17·6%	-7·10 [-9·37, -4·83]
Pofi 2022	49·1	4	55	51·4	4	43	18·7%	-2·30 [-3·90, -0·70]
Sáenz de Tejada 2002	64·7	14·7	136	66·3	16·1	55	12·5%	-1·60 [-6·52, 3·32]
Scheele 2016	54·2	12·5	164	55·7	11·1	62	15·6%	-1·50 [-4·86, 1·86]
**Subtotal (95% CI)**			**426**			**252**	**86·1%**	**-4·78 [-8·03, -1·53]**
Heterogeneity: Tau² = 12·22; Chi² = 34·46, df = 5 (P < 0·00001); I² = 85%								
Test for overall effect: Z = 2·89 (P = 0·004)								
**3·3·2 Other Trials**								
Fryk 2023	44·4	5·5	18	46·9	7·2	18	13·9%	-2·50 [-6·69, 1·69]
**Subtotal (95% CI)**			**18**			**18**	**13·9%**	**-2·50 [-6·69, 1·69]**
Heterogeneity: Not applicable								
Test for overall effect: Z = 1·17 (P = 0·24)								
**Total (95% CI)**			**444**			**270**	**100·0%**	**-4·38 [-7·23, -1·52]**
Heterogeneity: Tau² = 10·72; Chi² = 34·71, df = 6 (P < 0·00001); I² = 83%								
Test for overall effect: Z = 3·00 (P = 0·003)								
Test for subgroup differences: Chi² = 0·71, df = 1 (P = 0·40), I² = 0%								

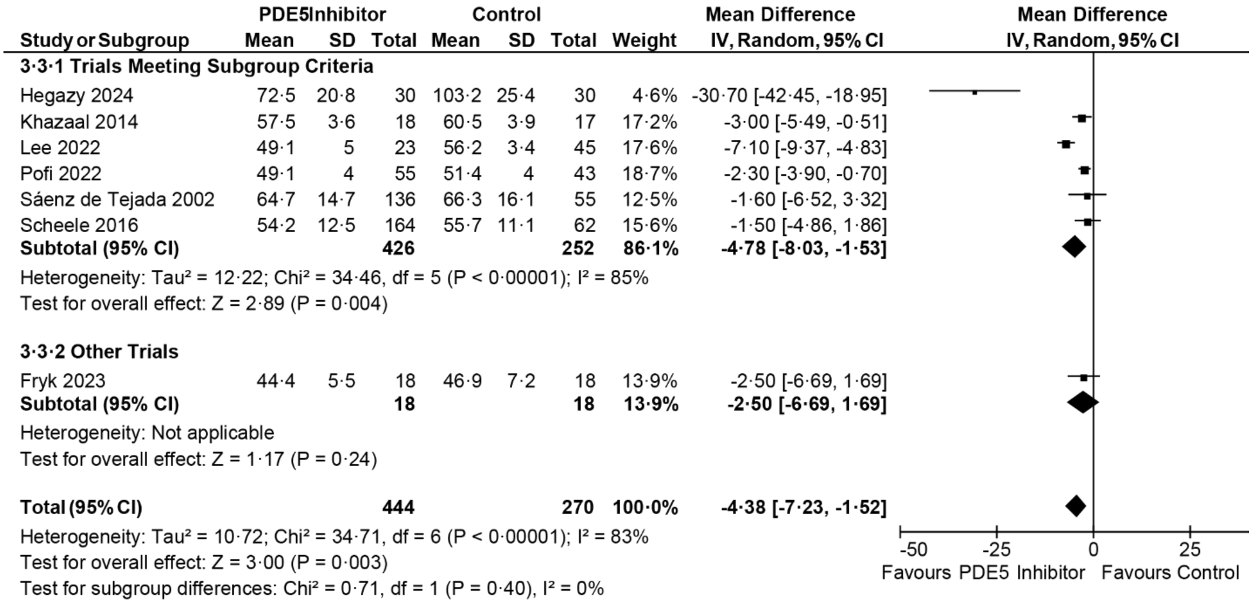

d)

Study or Subgroup	PDE5Inhibitor Mean	PDE5Inhibitor SD	PDE5Inhibitor Total	Control Mean	Control SD	Control Total	Weight	Mean Difference IV, Random, 95% CI
**3·4·1 Trials Meeting Subgroup Criteria**								
Derosa 2022	57·4	10·9	30	56·3	9·8	30	23·1%	1·10 [-4·15, 6·35]
Giannetta 2012	56	11·8	25	49·4	11·8	29	17·4%	6·60 [0·29, 12·91]
Kirilmaz 2015	66·1	16·4	41	61·8	19·7	42	12·3%	4·30 [-3·49, 12·09]
Liu 2016	42·1	4·9	26	42·4	7·4	26	40·2%	-0·30 [-3·71, 3·11]
Morano 2007	63·9	27·3	8	47·5	16·4	8	1·7%	16·40 [-5·67, 38·47]
**Subtotal (95% CI)**			**130**			**135**	**94·7%**	**2·43 [-0·90, 5·75]**
Heterogeneity: Tau² = 4·38; Chi² = 5·82, df = 4 (P = 0·21); I² = 31%								
Test for overall effect: Z = 1·43 (P = 0·15)								
**3·4·2 Other Trials**								
Grover-Páez 2007	58·4	28·1	20	60·4	2·6	20	5·3%	-2·00 [-14·37, 10·37]
**Subtotal (95% CI)**			**20**			**20**	**5·3%**	**-2·00 [-14·37, 10·37]**
Heterogeneity: Not applicable								
Test for overall effect: Z = 0·32 (P = 0·75)								
**Total (95% CI)**			**150**			**155**	**100·0%**	**1·99 [-0·95, 4·93]**
Heterogeneity: Tau² = 2·57; Chi² = 6·15, df = 5 (P = 0·29); I² = 19%								
Test for overall effect: Z = 1·32 (P = 0·19)								
Test for subgroup differences: Chi² = 0·46, df = 1 (P = 0·50), I² = 0%								

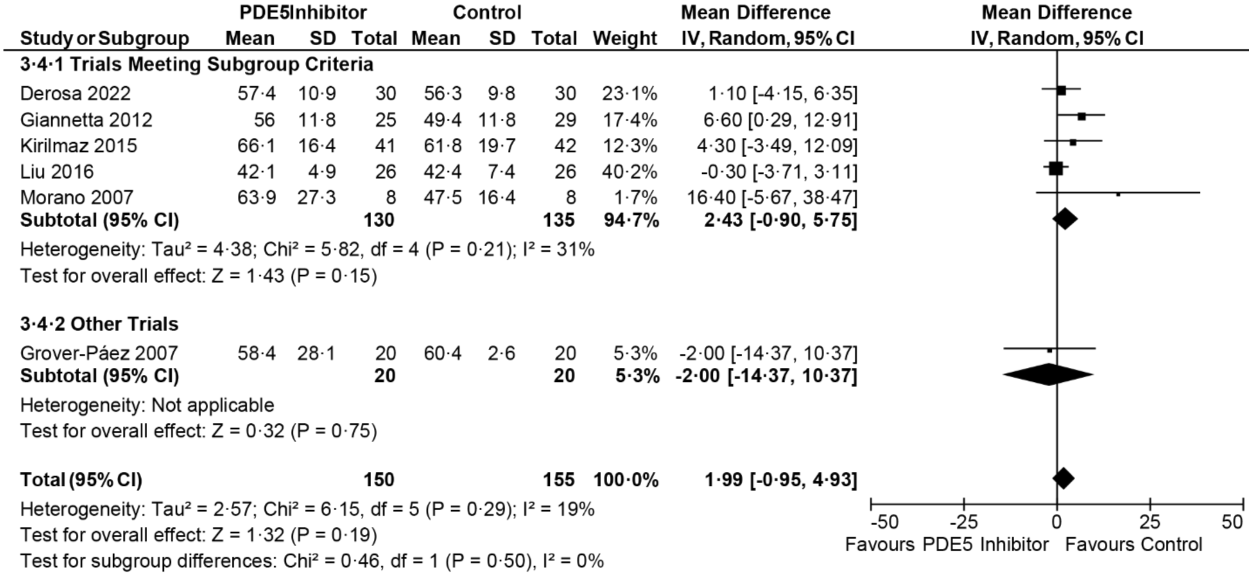

Supplementary Fig. 9: Sensitivity analysis of meta-analysis containing only trials with only participants with type 2 diabetes and with a treatment period of at least 8 weeks

Sensitivity analysis was performed on a potential decision to include trials which ran for at least 8 weeks and only included participants with type 2 diabetes, as well as on a potential decision to represent data in IFCC units (mmol/mol) or NGSP units (%). The following conditions were used for each meta-analysis: a) long half-life PDE5 inhibitors using NGSP units; b) short half-life PDE5 inhibitors using NGSP units; c) long half-life PDE5 inhibitors using IFCC units; d) short half-life PDE5 inhibitors using IFCC units.

a)

	PDE5Inhibitor			Control				Mean Difference
Study or Subgroup	Mean	SD	Total	Mean	SD	Total	Weight	IV, Random, 95% CI
**4·1·1 Trials Meeting Subgroup Criteria**								
Hegazy 2024	8·78	1·9	30	11·59	2·32	30	4·5%	-2·81 [-3·88, -1·74]
Khazaal 2014	7·41	0·33	18	7·69	0·35	17	17·3%	-0·28 [-0·51, -0·05]
Lee 2022	6·64	0·46	23	7·29	0·31	45	17·6%	-0·65 [-0·86, -0·44]
Pofi 2022	6·64	0·37	55	6·86	0·37	43	18·7%	-0·22 [-0·37, -0·07]
Scheele 2016	7·11	1·14	164	7·25	1·02	62	15·6%	-0·14 [-0·45, 0·17]
**Subtotal (95% CI)**			**290**			**197**	**73·7%**	**-0·50 [-0·83, -0·17]**
Heterogeneity: Tau² = 0·11; Chi² = 33·25, df = 4 (P < 0·00001); I² = 88%								
Test for overall effect: Z = 2·95 (P = 0·003)								
**4·1·2 Other Trials**								
Fryk 2023	6·21	0·51	18	6·44	0·66	18	13·9%	-0·23 [-0·62, 0·16]
Sáenz de Tejada 2002	8·07	1·34	136	8·21	1·48	55	12·4%	-0·14 [-0·59, 0·31]
**Subtotal (95% CI)**			**154**			**73**	**26·3%**	**-0·19 [-0·49, 0·10]**
Heterogeneity: Tau² = 0·00; Chi² = 0·09, df = 1 (P = 0·77); I² = 0%								
Test for overall effect: Z = 1·28 (P = 0·20)								
**Total (95% CI)**			**444**			**270**	**100·0%**	**-0·40 [-0·66, -0·14]**
Heterogeneity: Tau² = 0·09; Chi² = 34·27, df = 6 (P < 0·00001); I² = 82%								
Test for overall effect: Z = 3·03 (P = 0·002)								
Test for subgroup differences: Chi² = 1·86, df = 1 (P = 0·17), I² = 46·2%								

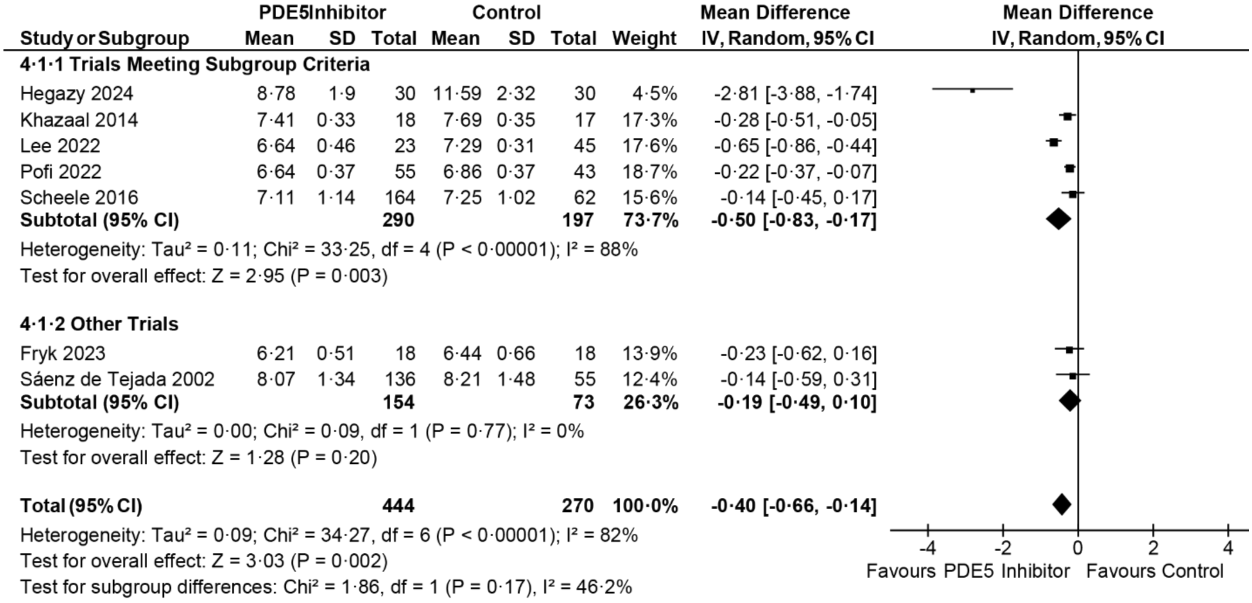

b)

	PDE5Inhibitor			Control				Mean Difference
Study or Subgroup	Mean	SD	Total	Mean	SD	Total	Weight	IV, Random, 95% CI
**4·2·1 Trials Meeting Subgroup Criteria**								
Derosa 2022	7·4	1	30	7·3	0·9	30	23·1%	0·10 [-0·38, 0·58]
Giannetta 2012	7·27	1·08	25	6·67	1·08	29	17·4%	0·60 [0·02, 1·18]
Kirilmaz 2015	8·2	1·5	41	7·8	1·8	42	12·3%	0·40 [-0·31, 1·11]
Morano 2007	8	2·5	8	6·5	1·5	8	1·7%	1·50 [-0·52, 3·52]
**Subtotal (95% CI)**			**104**			**109**	**54·6%**	**0·36 [0·03, 0·68]**
Heterogeneity: Tau² = 0·00; Chi² = 3·02, df = 3 (P = 0·39); I² = 1%								
Test for overall effect: Z = 2·14 (P = 0·03)								
**4·2·2 Other Trials**								
Grover-Páez 2007	7·49	2·57	20	7·67	0·24	20	5·3%	-0·18 [-1·31, 0·95]
Liu 2016	6	0·45	26	6·03	0·68	26	40·1%	-0·03 [-0·34, 0·28]
**Subtotal (95% CI)**			**46**			**46**	**45·4%**	**-0·04 [-0·34, 0·26]**
Heterogeneity: Tau² = 0·00; Chi² = 0·06, df = 1 (P = 0·80); I² = 0%								
Test for overall effect: Z = 0·26 (P = 0·79)								
**Total (95% CI)**			**150**			**155**	**100·0%**	**0·18 [-0·09, 0·45]**
Heterogeneity: Tau² = 0·02; Chi² = 6·15, df = 5 (P = 0·29); I² = 19%								
Test for overall effect: Z = 1·32 (P = 0·19)								
Test for subgroup differences: Chi² = 3·07, df = 1 (P = 0·08), I² = 67·4%								

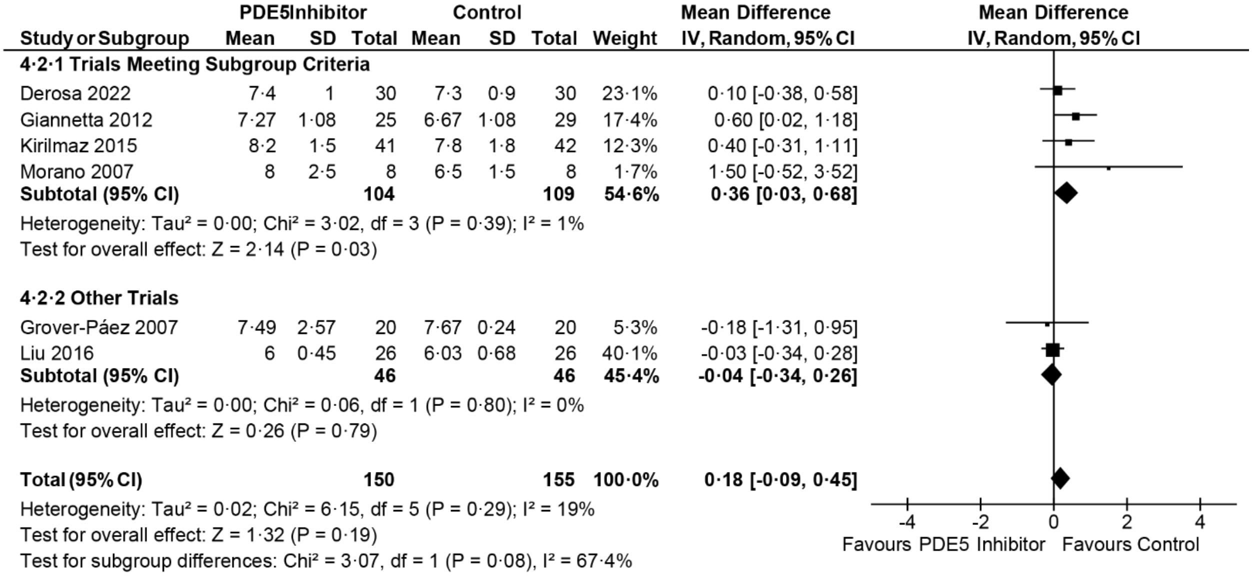

c)

Study or Subgroup	PDE5 Inhibitor Mean	SD	Total	Control Mean	SD	Total	Weight	Mean Difference IV, Random, 95% CI
**4·3·1 Trials Meeting Subgroup Criteria**								
Hegazy 2024	72·5	20·8	30	103·2	25·4	30	4·6%	-30·70 [-42·45, -18·95]
Khazaal 2014	57·5	3·6	18	60·5	3·9	17	17·2%	-3·00 [-5·49, -0·51]
Lee 2022	49·1	5	23	56·2	3·4	45	17·6%	-7·10 [-9·37, -4·83]
Pofi 2022	49·1	4	55	51·4	4	43	18·7%	-2·30 [-3·90, -0·70]
Scheele 2016	54·2	12·5	164	55·7	11·1	62	15·6%	-1·50 [-4·86, 1·86]
**Subtotal (95% CI)**			**290**			**197**	**73·6%**	**-5·44 [-9·10, -1·77]**
Heterogeneity: Tau² = 13·47; Chi² = 33·80, df = 4 (P < 0·00001); I² = 88%								
Test for overall effect: Z = 2·91 (P = 0·004)								
**4·3·2 Other Trials**								
Fryk 2023	44·4	5·5	18	46·9	7·2	18	13·9%	-2·50 [-6·69, 1·69]
Sáenz de Tejada 2002	64·7	14·7	136	66·3	16·1	55	12·5%	-1·60 [-6·52, 3·32]
**Subtotal (95% CI)**			**154**			**73**	**26·4%**	**-2·12 [-5·31, 1·07]**
Heterogeneity: Tau² = 0·00; Chi² = 0·07, df = 1 (P = 0·78); I² = 0%								
Test for overall effect: Z = 1·30 (P = 0·19)								
**Total (95% CI)**			**444**			**270**	**100·0%**	**-4·38 [-7·23, -1·52]**
Heterogeneity: Tau² = 10·72; Chi² = 34·71, df = 6 (P < 0·00001); I² = 83%								
Test for overall effect: Z = 3·00 (P = 0·003)								
Test for subgroup differences: Chi² = 1·79, df = 1 (P = 0·18), I² = 44·1%								

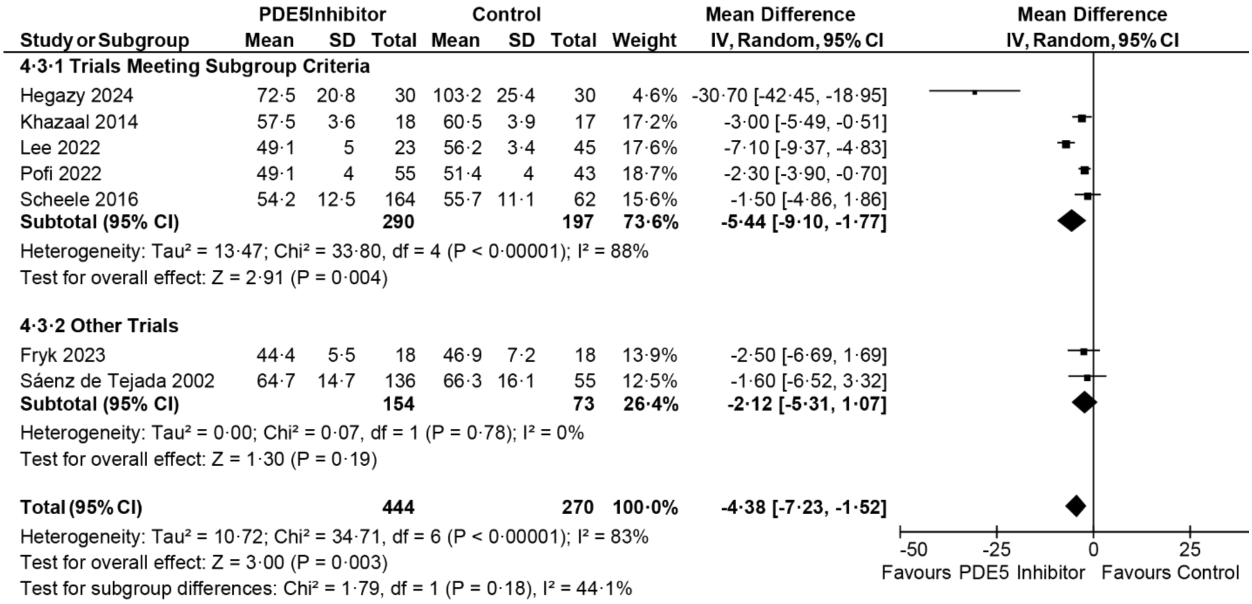

d)

Study or Subgroup	PDE5 Inhibitor Mean	SD	Total	Control Mean	SD	Total	Weight	Mean Difference IV, Random, 95% CI
**4·4·1 Trials Meeting Subgroup Criteria**								
Derosa 2022	57·4	10·9	30	56·3	9·8	30	23·1%	1·10 [-4·15, 6·35]
Giannetta 2012	56	11·8	25	49·4	11·8	29	17·4%	6·60 [0·29, 12·91]
Kirilmaz 2015	66·1	16·4	41	61·8	19·7	42	12·3%	4·30 [-3·49, 12·09]
Morano 2007	63·9	27·3	8	47·5	16·4	8	1·7%	16·40 [-5·67, 38·47]
**Subtotal (95% CI)**			**104**			**109**	**54·5%**	**3·90 [0·32, 7·47]**
Heterogeneity: Tau² = 0·20; Chi² = 3·04, df = 3 (P = 0·39); I² = 1%								
Test for overall effect: Z = 2·14 (P = 0·03)								
**4·4·2 Other Trials**								
Grover-Páez 2007	58·4	28·1	20	60·4	2·6	20	5·3%	-2·00 [-14·37, 10·37]
Liu 2016	42·1	4·9	26	42·4	7·4	26	40·2%	-0·30 [-3·71, 3·11]
**Subtotal (95% CI)**			**46**			**46**	**45·5%**	**-0·42 [-3·71, 2·87]**
Heterogeneity: Tau² = 0·00; Chi² = 0·07, df = 1 (P = 0·80); I² = 0%								
Test for overall effect: Z = 0·25 (P = 0·80)								
**Total (95% CI)**			**150**			**155**	**100·0%**	**1·99 [-0·95, 4·93]**
Heterogeneity: Tau² = 2·57; Chi² = 6·15, df = 5 (P = 0·29); I² = 19%								
Test for overall effect: Z = 1·32 (P = 0·19)								
Test for subgroup differences: Chi² = 3·03, df = 1 (P = 0·08), I² = 67·0%								

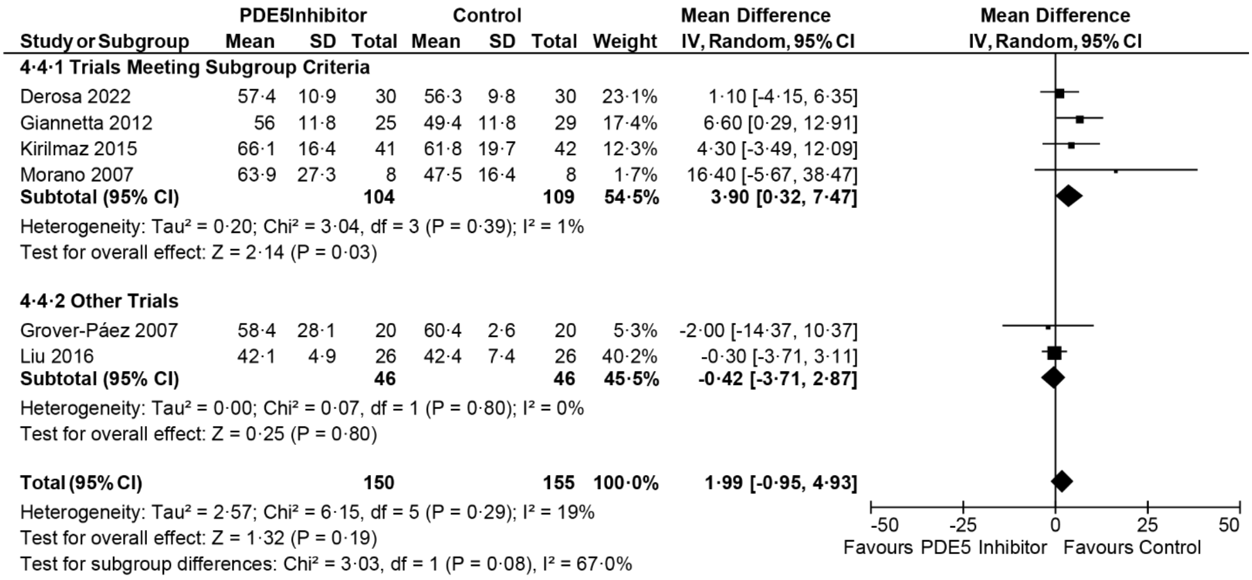

Supplementary Fig. 10: Sensitivity analysis of meta-analysis containing only trials with participants with a mean baseline HbA1c of at least 6.5%

Sensitivity analysis was performed on a potential decision to include trials which only included participants with a mean baseline HbA1c of at least 6.5%, as well as on a potential decision to represent data in IFCC units (mmol/mol) or NGSP units (%). The following conditions were used for each meta-analysis: a) long half-life PDE5 inhibitors using NGSP units; b) short half-life PDE5 inhibitors using NGSP units; c) long half-life PDE5 inhibitors using IFCC units; d) short half-life PDE5 inhibitors using IFCC units.

a)

	PDE5Inhibitor			Control				Mean Difference
Study or Subgroup	Mean	SD	Total	Mean	SD	Total	Weight	IV, Random, 95% CI
**5·1·1 Trials Meeting Subgroup Criteria**								
Hegazy 2024	8·78	1·9	30	11·59	2·32	30	4·5%	-2·81 [-3·88, -1·74]
Khazaal 2014	7·41	0·33	18	7·69	0·35	17	17·3%	-0·28 [-0·51, -0·05]
Lee 2022	6·64	0·46	23	7·29	0·31	45	17·6%	-0·65 [-0·86, -0·44]
Pofi 2022	6·64	0·37	55	6·86	0·37	43	18·7%	-0·22 [-0·37, -0·07]
Sáenz de Tejada 2002	8·07	1·34	136	8·21	1·48	55	12·4%	-0·14 [-0·59, 0·31]
Scheele 2016	7·11	1·14	164	7·25	1·02	62	15·6%	-0·14 [-0·45, 0·17]
**Subtotal (95% CI)**			**426**			**252**	**86·1%**	**-0·44 [-0·74, -0·14]**
Heterogeneity: Tau² = 0·10; Chi² = 34·01, df = 5 (P < 0·00001); I² = 85%								
Test for overall effect: Z = 2·92 (P = 0·004)								
**5·1·2 Other Trials**								
Fryk 2023	6·21	0·51	18	6·44	0·66	18	13·9%	-0·23 [-0·62, 0·16]
**Subtotal (95% CI)**			**18**			**18**	**13·9%**	**-0·23 [-0·62, 0·16]**
Heterogeneity: Not applicable								
Test for overall effect: Z = 1·17 (P = 0·24)								
**Total (95% CI)**			**444**			**270**	**100·0%**	**-0·40 [-0·66, -0·14]**
Heterogeneity: Tau² = 0·09; Chi² = 34·27, df = 6 (P < 0·00001); I² = 82%								
Test for overall effect: Z = 3·03 (P = 0·002)								
Test for subgroup differences: Chi² = 0·72, df = 1 (P = 0·40), I² = 0%								

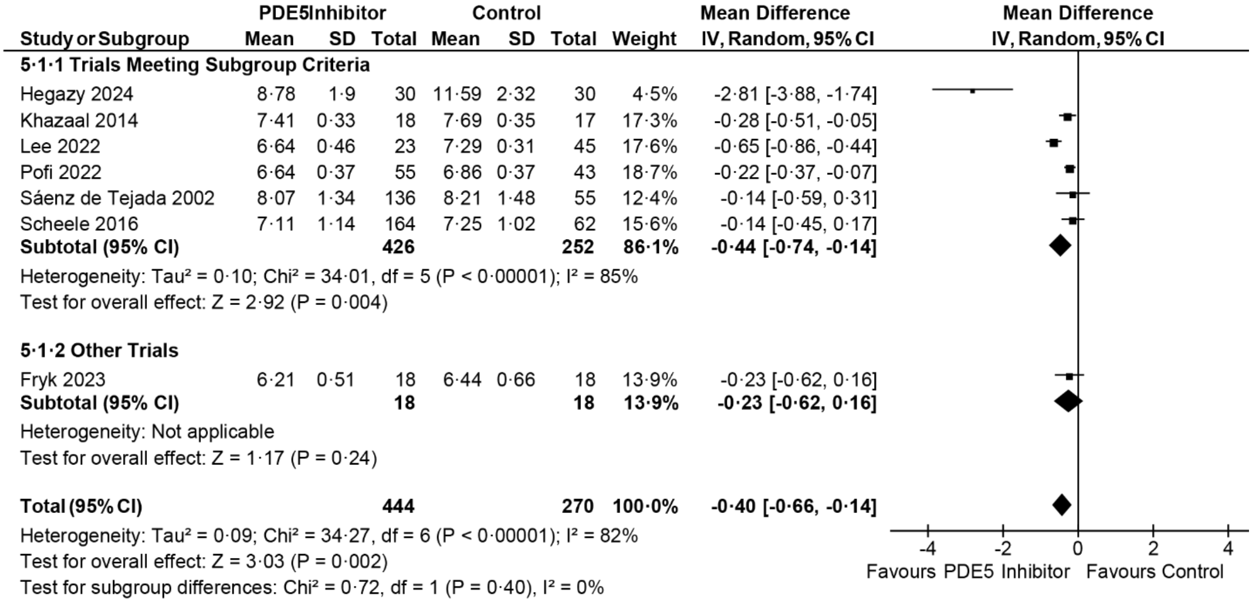

b)

	PDE5Inhibitor			Control				Mean Difference
Study or Subgroup	Mean	SD	Total	Mean	SD	Total	Weight	IV, Random, 95% CI
**5·2·1 Trials Meeting Subgroup Criteria**								
Derosa 2022	7·4	1	30	7·3	0·9	30	23·1%	0·10 [-0·38, 0·58]
Giannetta 2012	7·27	1·08	25	6·67	1·08	29	17·4%	0·60 [0·02, 1·18]
Grover-Páez 2007	7·49	2·57	20	7·67	0·24	20	5·3%	-0·18 [-1·31, 0·95]
Kirilmaz 2015	8·2	1·5	41	7·8	1·8	42	12·3%	0·40 [-0·31, 1·11]
Morano 2007	8	2·5	8	6·5	1·5	8	1·7%	1·50 [-0·52, 3·52]
**Subtotal (95% CI)**			**124**			**129**	**59·9%**	**0·31 [0·00, 0·63]**
Heterogeneity: Tau² = 0·00; Chi² = 3·81, df = 4 (P = 0·43); I² = 0%								
Test for overall effect: Z = 1·98 (P = 0·05)								
**5·2·2 Other Trials**								
Liu 2016	6	0·45	26	6·03	0·68	26	40·1%	-0·03 [-0·34, 0·28]
**Subtotal (95% CI)**			**26**			**26**	**40·1%**	**-0·03 [-0·34, 0·28]**
Heterogeneity: Not applicable								
Test for overall effect: Z = 0·19 (P = 0·85)								
**Total (95% CI)**			**150**			**155**	**100·0%**	**0·18 [-0·09, 0·45]**
Heterogeneity: Tau² = 0·02; Chi² = 6·15, df = 5 (P = 0·29); I² = 19%								
Test for overall effect: Z = 1·32 (P = 0·19)								
Test for subgroup differences: Chi² = 2·34, df = 1 (P = 0·13), I² = 57·2%								

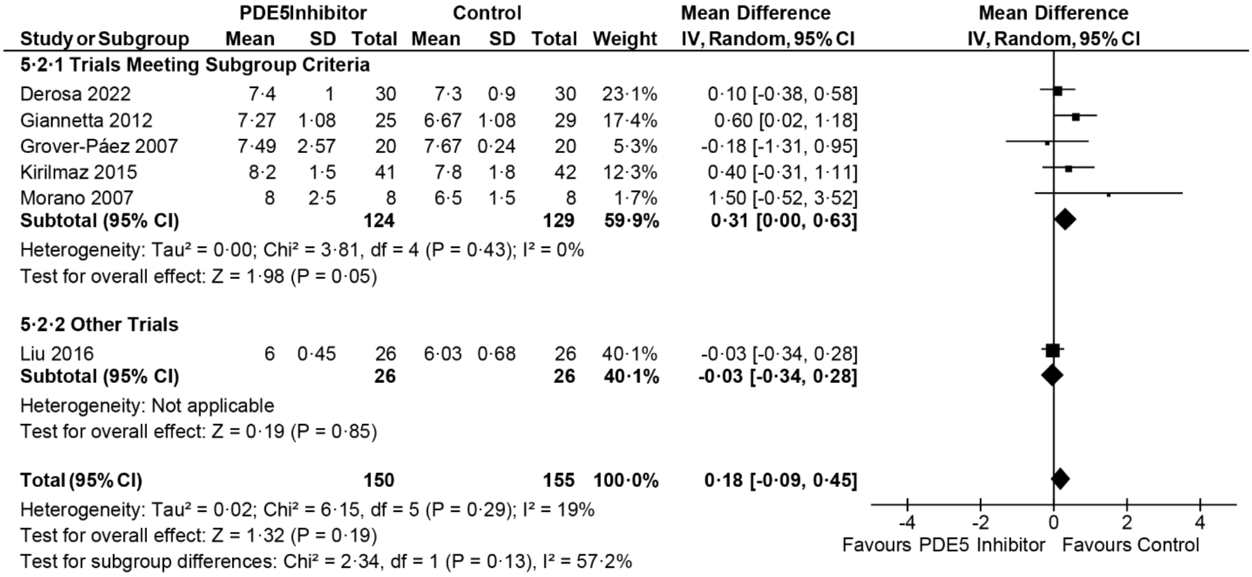

c)

Study or Subgroup	PDE5Inhibitor Mean	SD	Total	Control Mean	SD	Total	Weight	Mean Difference IV, Random, 95% CI
**5·3·1 Trials Meeting Subgroup Criteria**								
Hegazy 2024	72·5	20·8	30	103·2	25·4	30	4·6%	-30·70 [-42·45, -18·95]
Khazaal 2014	57·5	3·6	18	60·5	3·9	17	17·2%	-3·00 [-5·49, -0·51]
Lee 2022	49·1	5	23	56·2	3·4	45	17·6%	-7·10 [-9·37, -4·83]
Pofi 2022	49·1	4	55	51·4	4	43	18·7%	-2·30 [-3·90, -0·70]
Sáenz de Tejada 2002	64·7	14·7	136	66·3	16·1	55	12·5%	-1·60 [-6·52, 3·32]
Scheele 2016	54·2	12·5	164	55·7	11·1	62	15·6%	-1·50 [-4·86, 1·86]
**Subtotal (95% CI)**			**426**			**252**	**86·1%**	**-4·78 [-8·03, -1·53]**
Heterogeneity: Tau² = 12·22; Chi² = 34·46, df = 5 (P < 0·00001); I² = 85%								
Test for overall effect: Z = 2·89 (P = 0·004)								
**5·3·2 Other Trials**								
Fryk 2023	44·4	5·5	18	46·9	7·2	18	13·9%	-2·50 [-6·69, 1·69]
**Subtotal (95% CI)**			**18**			**18**	**13·9%**	**-2·50 [-6·69, 1·69]**
Heterogeneity: Not applicable								
Test for overall effect: Z = 1·17 (P = 0·24)								
**Total (95% CI)**			**444**			**270**	**100·0%**	**-4·38 [-7·23, -1·52]**
Heterogeneity: Tau² = 10·72; Chi² = 34·71, df = 6 (P < 0·00001); I² = 83%								
Test for overall effect: Z = 3·00 (P = 0·003)								
Test for subgroup differences: Chi² = 0·71, df = 1 (P = 0·40), I² = 0%								

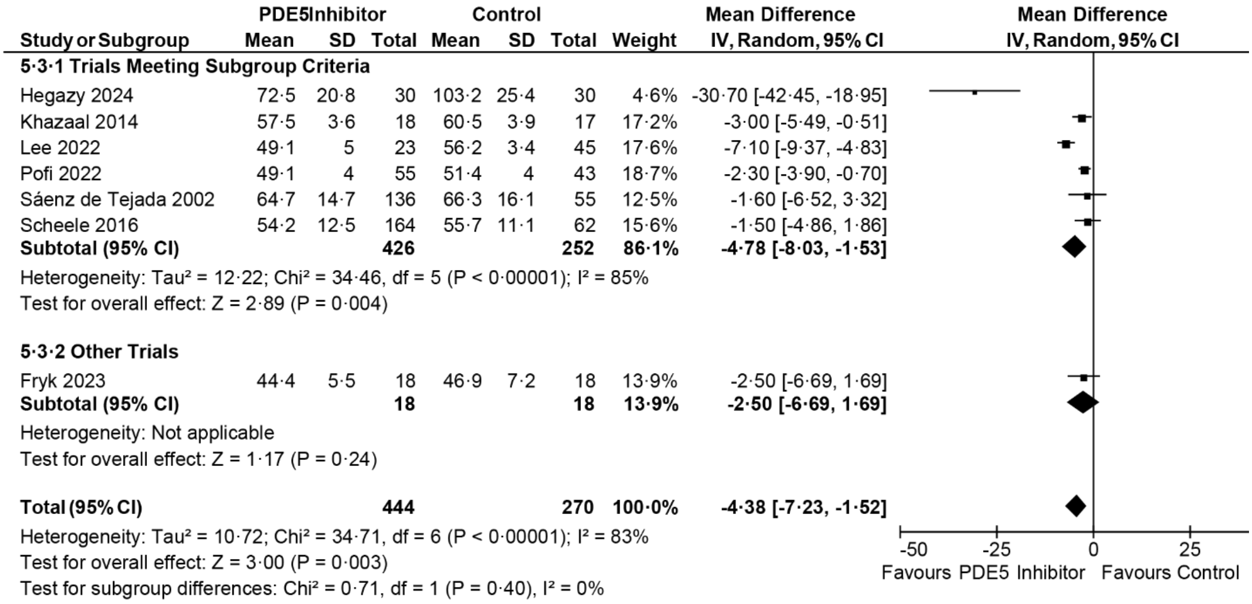

d)

Study or Subgroup	PDE5Inhibitor Mean	SD	Total	Control Mean	SD	Total	Weight	Mean Difference IV, Random, 95% CI
**5·4·1 Trials Meeting Subgroup Criteria**								
Derosa 2022	57·4	10·9	30	56·3	9·8	30	23·1%	1·10 [-4·15, 6·35]
Giannetta 2012	56	11·8	25	49·4	11·8	29	17·4%	6·60 [0·29, 12·91]
Grover-Páez 2007	58·4	28·1	20	60·4	2·6	20	5·3%	-2·00 [-14·37, 10·37]
Kirilmaz 2015	66·1	16·4	41	61·8	19·7	42	12·3%	4·30 [-3·49, 12·09]
Morano 2007	63·9	27·3	8	47·5	16·4	8	1·7%	16·40 [-5·67, 38·47]
**Subtotal (95% CI)**			**124**			**129**	**59·8%**	**3·43 [0·03, 6·83]**
Heterogeneity: Tau² = 0·00; Chi² = 3·84, df = 4 (P = 0·43); I² = 0%								
Test for overall effect: Z = 1·98 (P = 0·05)								
**5·4·2 Other Trials**								
Liu 2016	42·1	4·9	26	42·4	7·4	26	40·2%	-0·30 [-3·71, 3·11]
**Subtotal (95% CI)**			**26**			**26**	**40·2%**	**-0·30 [-3·71, 3·11]**
Heterogeneity: Not applicable								
Test for overall effect: Z = 0·17 (P = 0·86)								
**Total (95% CI)**			**150**			**155**	**100·0%**	**1·99 [-0·95, 4·93]**
Heterogeneity: Tau² = 2·57; Chi² = 6·15, df = 5 (P = 0·29); I² = 19%								
Test for overall effect: Z = 1·32 (P = 0·19)								
Test for subgroup differences: Chi² = 2·31, df = 1 (P = 0·13), I² = 56·7%								

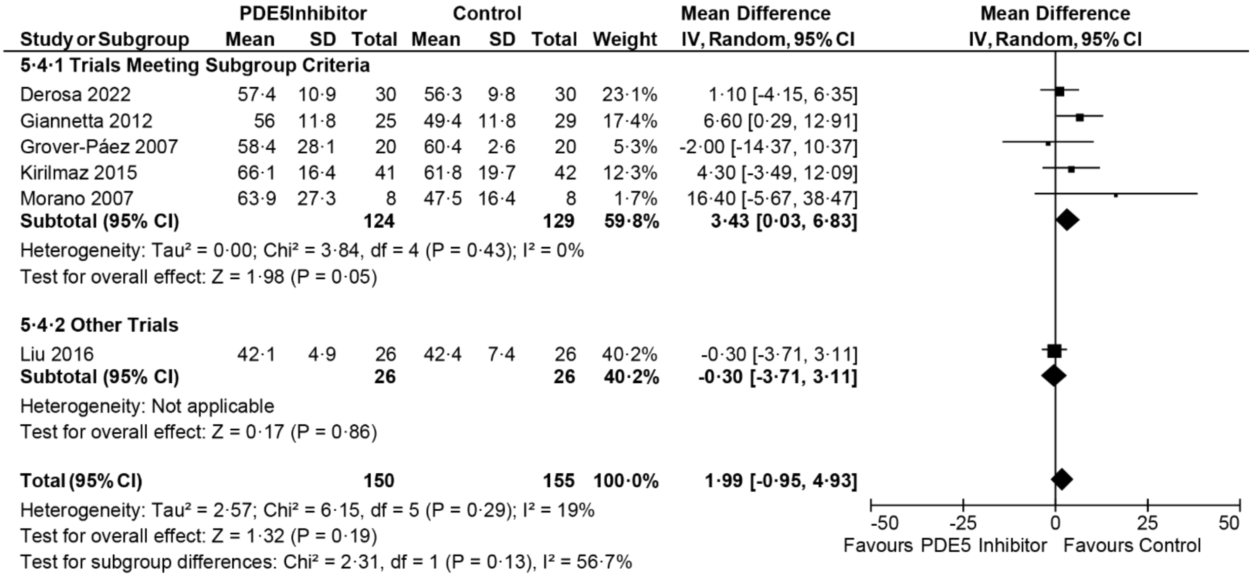

Supplementary Fig. 11: Sensitivity analysis of meta-analysis containing only trials with participants with type 2 diabetes and a mean baseline HbA1c of at least 6.5%

Sensitivity analysis was performed on a potential decision to include trials which only included participants with type 2 diabetes and a mean baseline HbA1c of at least 6.5%, as well as on a potential decision to represent data in IFCC units (mmol/mol) or NGSP units (%). The following conditions were used for each meta-analysis: a) long half-life PDE5 inhibitors using NGSP units; b) short half-life PDE5 inhibitors using NGSP units; c) long half-life PDE5 inhibitors using IFCC units; d) short half-life PDE5 inhibitors using IFCC units.

a)

	PDE5Inhibitor			Control				Mean Difference
Study or Subgroup	Mean	SD	Total	Mean	SD	Total	Weight	IV, Random, 95% CI
**6·1·1 Trials Meeting Subgroup Criteria**								
Hegazy 2024	8·78	1·9	30	11·59	2·32	30	4·5%	-2·81 [-3·88, -1·74]
Khazaal 2014	7·41	0·33	18	7·69	0·35	17	17·3%	-0·28 [-0·51, -0·05]
Lee 2022	6·64	0·46	23	7·29	0·31	45	17·6%	-0·65 [-0·86, -0·44]
Pofi 2022	6·64	0·37	55	6·86	0·37	43	18·7%	-0·22 [-0·37, -0·07]
Scheele 2016	7·11	1·14	164	7·25	1·02	62	15·6%	-0·14 [-0·45, 0·17]
**Subtotal (95% CI)**			**290**			**197**	**73·7%**	**-0·50 [-0·83, -0·17]**
Heterogeneity: Tau² = 0·11; Chi² = 33·25, df = 4 (P < 0·00001); I² = 88%								
Test for overall effect: Z = 2·95 (P = 0·003)								
**6·1·2 Other Trials**								
Fryk 2023	6·21	0·51	18	6·44	0·66	18	13·9%	-0·23 [-0·62, 0·16]
Sáenz de Tejada 2002	8·07	1·34	136	8·21	1·48	55	12·4%	-0·14 [-0·59, 0·31]
**Subtotal (95% CI)**			**154**			**73**	**26·3%**	**-0·19 [-0·49, 0·10]**
Heterogeneity: Tau² = 0·00; Chi² = 0·09, df = 1 (P = 0·77); I² = 0%								
Test for overall effect: Z = 1·28 (P = 0·20)								
**Total (95% CI)**			**444**			**270**	**100·0%**	**-0·40 [-0·66, -0·14]**
Heterogeneity: Tau² = 0·09; Chi² = 34·27, df = 6 (P < 0·00001); I² = 82%								
Test for overall effect: Z = 3·03 (P = 0·002)								
Test for subgroup differences: Chi² = 1·86, df = 1 (P = 0·17), I² = 46·2%								

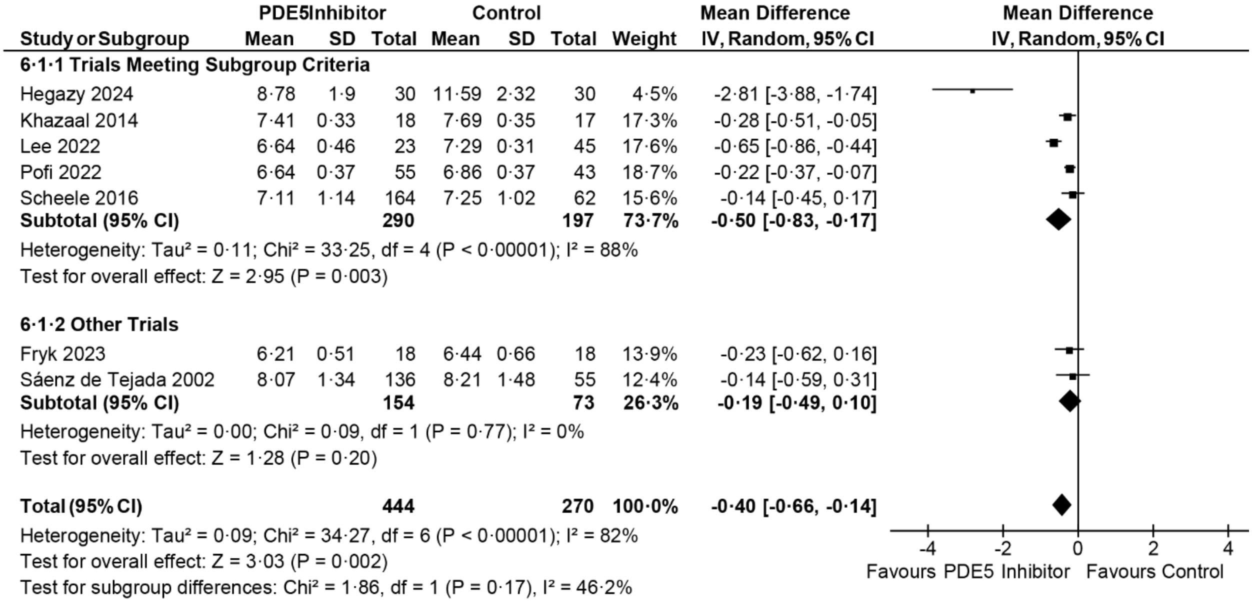

b)

	PDE5Inhibitor			Control				Mean Difference
Study or Subgroup	Mean	SD	Total	Mean	SD	Total	Weight	IV, Random, 95% CI
**6·2·1 Trials Meeting Subgroup Criteria**								
Derosa 2022	7·4	1	30	7·3	0·9	30	23·1%	0·10 [-0·38, 0·58]
Giannetta 2012	7·27	1·08	25	6·67	1·08	29	17·4%	0·60 [0·02, 1·18]
Grover-Páez 2007	7·49	2·57	20	7·67	0·24	20	5·3%	-0·18 [-1·31, 0·95]
Kirilmaz 2015	8·2	1·5	41	7·8	1·8	42	12·3%	0·40 [-0·31, 1·11]
Morano 2007	8	2·5	8	6·5	1·5	8	1·7%	1·50 [-0·52, 3·52]
**Subtotal (95% CI)**			**124**			**129**	**59·9%**	**0·31 [0·00, 0·63]**
Heterogeneity: Tau² = 0·00; Chi² = 3·81, df = 4 (P = 0·43); I² = 0%								
Test for overall effect: Z = 1·98 (P = 0·05)								
**6·2·2 Other Trials**								
Liu 2016	6	0·45	26	6·03	0·68	26	40·1%	-0·03 [-0·34, 0·28]
**Subtotal (95% CI)**			**26**			**26**	**40·1%**	**-0·03 [-0·34, 0·28]**
Heterogeneity: Not applicable								
Test for overall effect: Z = 0·19 (P = 0·85)								
**Total (95% CI)**			**150**			**155**	**100·0%**	**0·18 [-0·09, 0·45]**
Heterogeneity: Tau² = 0·02; Chi² = 6·15, df = 5 (P = 0·29); I² = 19%								
Test for overall effect: Z = 1·32 (P = 0·19)								
Test for subgroup differences: Chi² = 2·34, df = 1 (P = 0·13), I² = 57·2%								

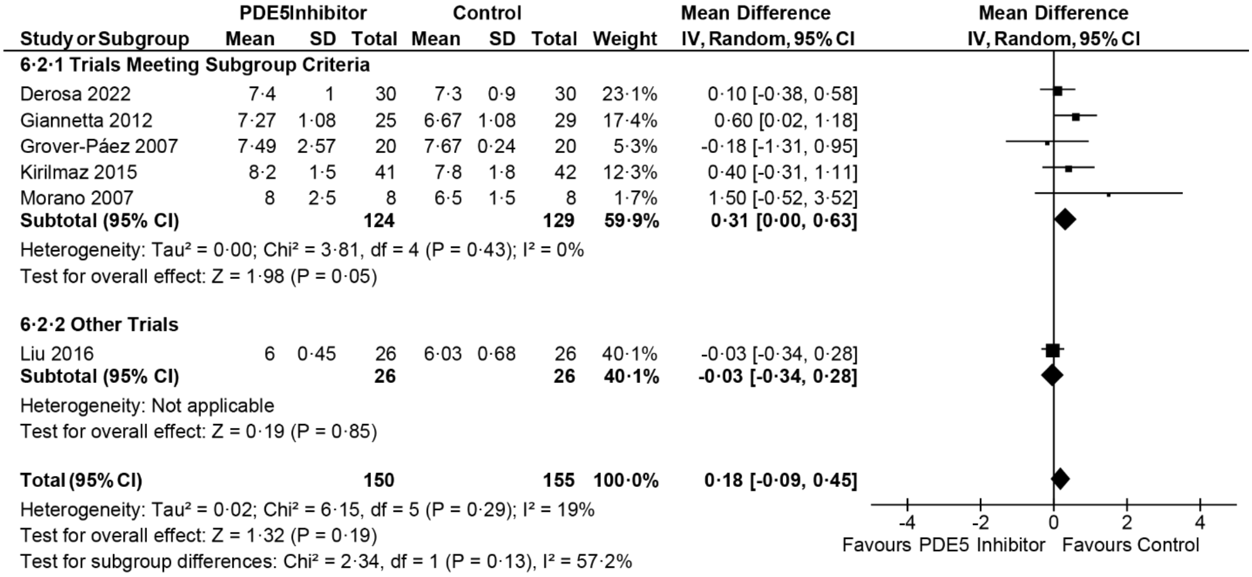

c)

Study or Subgroup	PDE5Inhibitor Mean	PDE5Inhibitor SD	PDE5Inhibitor Total	Control Mean	Control SD	Control Total	Weight	Mean Difference IV, Random, 95% CI
**6·3·1 Trials Meeting Subgroup Criteria**								
Hegazy 2024	72·5	20·8	30	103·2	25·4	30	4·6%	-30·70 [-42·45, -18·95]
Khazaal 2014	57·5	3·6	18	60·5	3·9	17	17·2%	-3·00 [-5·49, -0·51]
Lee 2022	49·1	5	23	56·2	3·4	45	17·6%	-7·10 [-9·37, -4·83]
Pofi 2022	49·1	4	55	51·4	4	43	18·7%	-2·30 [-3·90, -0·70]
Scheele 2016	54·2	12·5	164	55·7	11·1	62	15·6%	-1·50 [-4·86, 1·86]
**Subtotal (95% CI)**			**290**			**197**	**73·6%**	**-5·44 [-9·10, -1·77]**
Heterogeneity: Tau² = 13·47; Chi² = 33·80, df = 4 (P < 0·00001); I² = 88%								
Test for overall effect: Z = 2·91 (P = 0·004)								
**6·3·2 Other Trials**								
Fryk 2023	44·4	5·5	18	46·9	7·2	18	13·9%	-2·50 [-6·69, 1·69]
Sáenz de Tejada 2002	64·7	14·7	136	66·3	16·1	55	12·5%	-1·60 [-6·52, 3·32]
**Subtotal (95% CI)**			**154**			**73**	**26·4%**	**-2·12 [-5·31, 1·07]**
Heterogeneity: Tau² = 0·00; Chi² = 0·07, df = 1 (P = 0·78); I² = 0%								
Test for overall effect: Z = 1·30 (P = 0·19)								
**Total (95% CI)**			**444**			**270**	**100·0%**	**-4·38 [-7·23, -1·52]**
Heterogeneity: Tau² = 10·72; Chi² = 34·71, df = 6 (P < 0·00001); I² = 83%								
Test for overall effect: Z = 3·00 (P = 0·003)								
Test for subgroup differences: Chi² = 1·79, df = 1 (P = 0·18), I² = 44·1%								

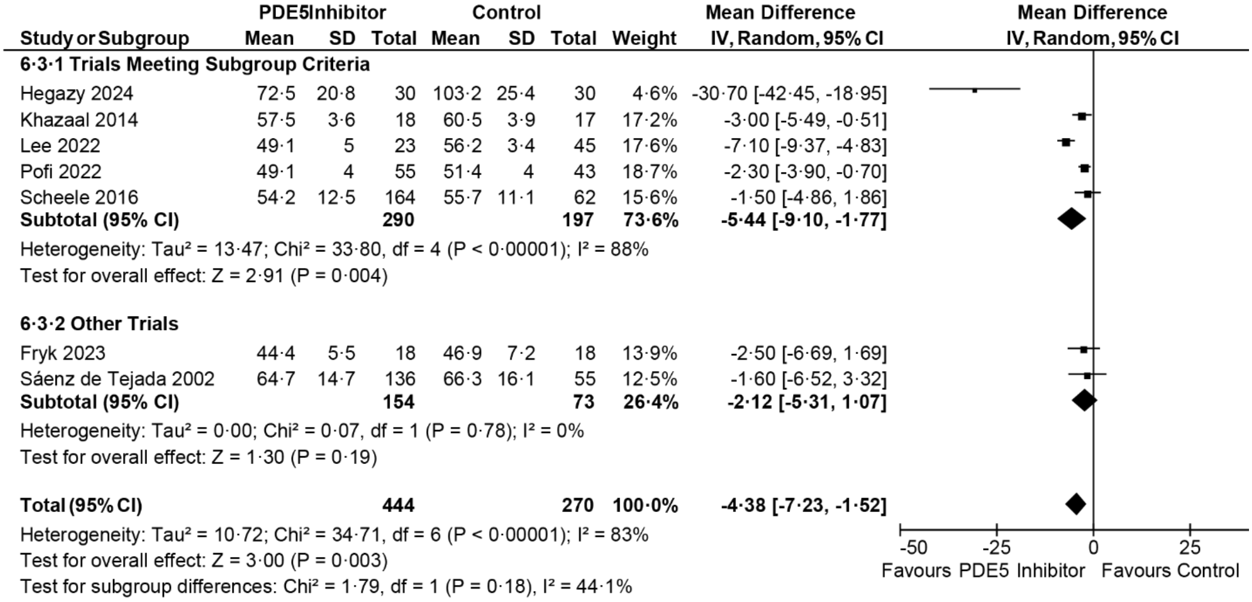

d)

Study or Subgroup	PDE5Inhibitor Mean	PDE5Inhibitor SD	PDE5Inhibitor Total	Control Mean	Control SD	Control Total	Weight	Mean Difference IV, Random, 95% CI
**6·4·1 Trials Meeting Subgroup Criteria**								
Derosa 2022	57·4	10·9	30	56·3	9·8	30	23·1%	1·10 [-4·15, 6·35]
Giannetta 2012	56	11·8	25	49·4	11·8	29	17·4%	6·60 [0·29, 12·91]
Grover-Páez 2007	58·4	28·1	20	60·4	2·6	20	5·3%	-2·00 [-14·37, 10·37]
Kirilmaz 2015	66·1	16·4	41	61·8	19·7	42	12·3%	4·30 [-3·49, 12·09]
Morano 2007	63·9	27·3	8	47·5	16·4	8	1·7%	16·40 [-5·67, 38·47]
**Subtotal (95% CI)**			**124**			**129**	**59·8%**	**3·43 [0·03, 6·83]**
Heterogeneity: Tau² = 0·00; Chi² = 3·84, df = 4 (P = 0·43); I² = 0%								
Test for overall effect: Z = 1·98 (P = 0·05)								
**6·4·2 Other Trials**								
Liu 2016	42·1	4·9	26	42·4	7·4	26	40·2%	-0·30 [-3·71, 3·11]
**Subtotal (95% CI)**			**26**			**26**	**40·2%**	**-0·30 [-3·71, 3·11]**
Heterogeneity: Not applicable								
Test for overall effect: Z = 0·17 (P = 0·86)								
**Total (95% CI)**			**150**			**155**	**100·0%**	**1·99 [-0·95, 4·93]**
Heterogeneity: Tau² = 2·57; Chi² = 6·15, df = 5 (P = 0·29); I² = 19%								
Test for overall effect: Z = 1·32 (P = 0·19)								
Test for subgroup differences: Chi² = 2·31, df = 1 (P = 0·13), I² = 56·7%								

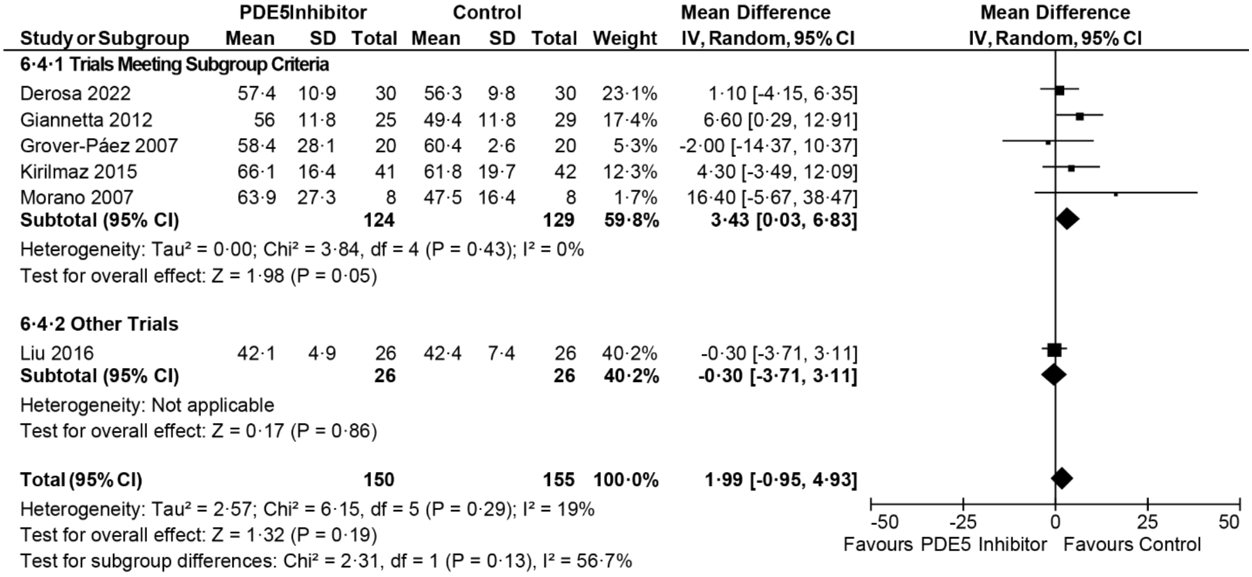

Supplementary Fig. 12: Sensitivity analysis of meta-analysis containing only trials with participants with a mean baseline HbA1c of at least 6.5% and with a treatment period of at least 8 weeks

Sensitivity analysis was performed on a potential decision to include trials which ran for at least 8 weeks and only included participants with a mean baseline HbA1c of at least 6.5%, as well as on a potential decision to represent data in IFCC units (mmol/mol) or NGSP units (%). The following conditions were used for each meta-analysis: a) long half-life PDE5 inhibitors using NGSP units; b) short half-life PDE5 inhibitors using NGSP units; c) long half-life PDE5 inhibitors using IFCC units; d) short half-life PDE5 inhibitors using IFCC units.

a)

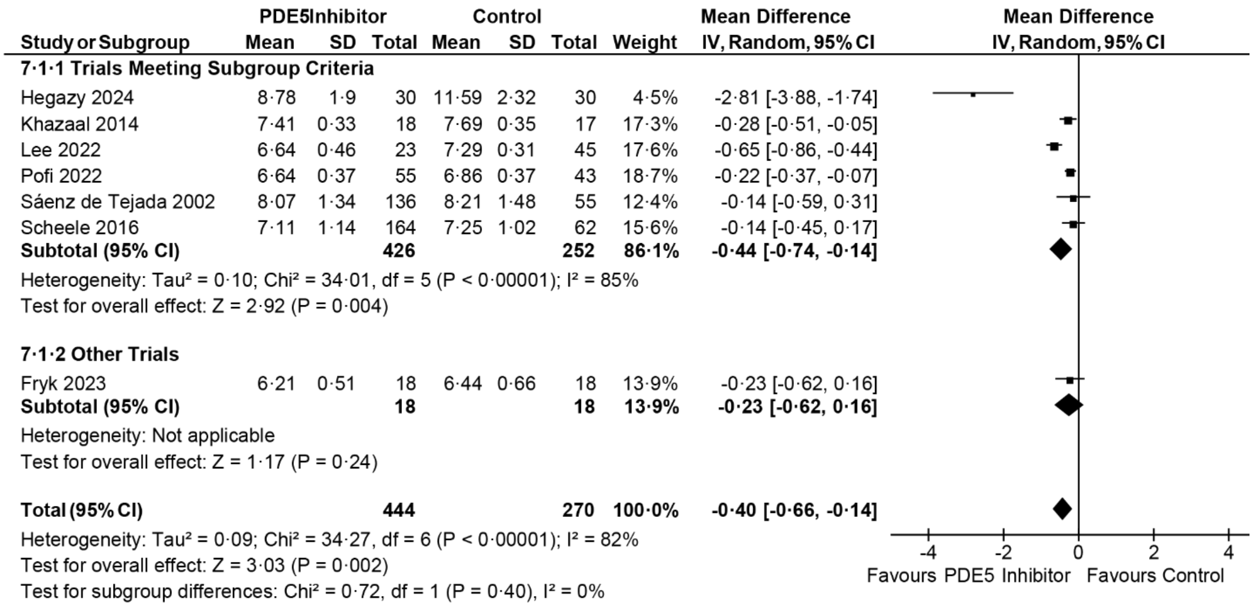

b)

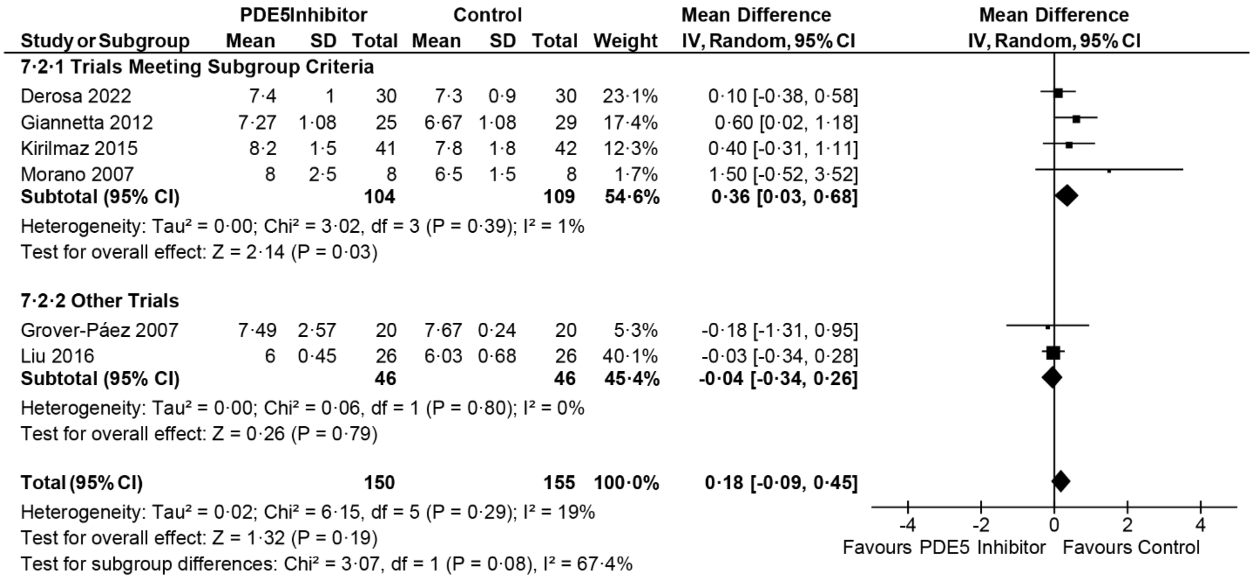

c)

Study or Subgroup	PDE5Inhibitor Mean	PDE5Inhibitor SD	PDE5Inhibitor Total	Control Mean	Control SD	Control Total	Weight	Mean Difference IV, Random, 95% CI
**7·3·1 Trials Meeting Subgroup Criteria**								
Hegazy 2024	72·5	20·8	30	103·2	25·4	30	4·6%	-30·70 [-42·45, -18·95]
Khazaal 2014	57·5	3·6	18	60·5	3·9	17	17·2%	-3·00 [-5·49, -0·51]
Lee 2022	49·1	5	23	56·2	3·4	45	17·6%	-7·10 [-9·37, -4·83]
Pofi 2022	49·1	4	55	51·4	4	43	18·7%	-2·30 [-3·90, -0·70]
Sáenz de Tejada 2002	64·7	14·7	136	66·3	16·1	55	12·5%	-1·60 [-6·52, 3·32]
Scheele 2016	54·2	12·5	164	55·7	11·1	62	15·6%	-1·50 [-4·86, 1·86]
**Subtotal (95% CI)**			**426**			**252**	**86·1%**	**-4·78 [-8·03, -1·53]**
Heterogeneity: Tau² = 12·22; Chi² = 34·46, df = 5 (P < 0·00001); I² = 85%								
Test for overall effect: Z = 2·89 (P = 0·004)								
**7·3·2 Other Trials**								
Fryk 2023	44·4	5·5	18	46·9	7·2	18	13·9%	-2·50 [-6·69, 1·69]
**Subtotal (95% CI)**			**18**			**18**	**13·9%**	**-2·50 [-6·69, 1·69]**
Heterogeneity: Not applicable								
Test for overall effect: Z = 1·17 (P = 0·24)								
**Total (95% CI)**			**444**			**270**	**100·0%**	**-4·38 [-7·23, -1·52]**
Heterogeneity: Tau² = 10·72; Chi² = 34·71, df = 6 (P < 0·00001); I² = 83%								
Test for overall effect: Z = 3·00 (P = 0·003)								
Test for subgroup differences: Chi² = 0·71, df = 1 (P = 0·40), I² = 0%								

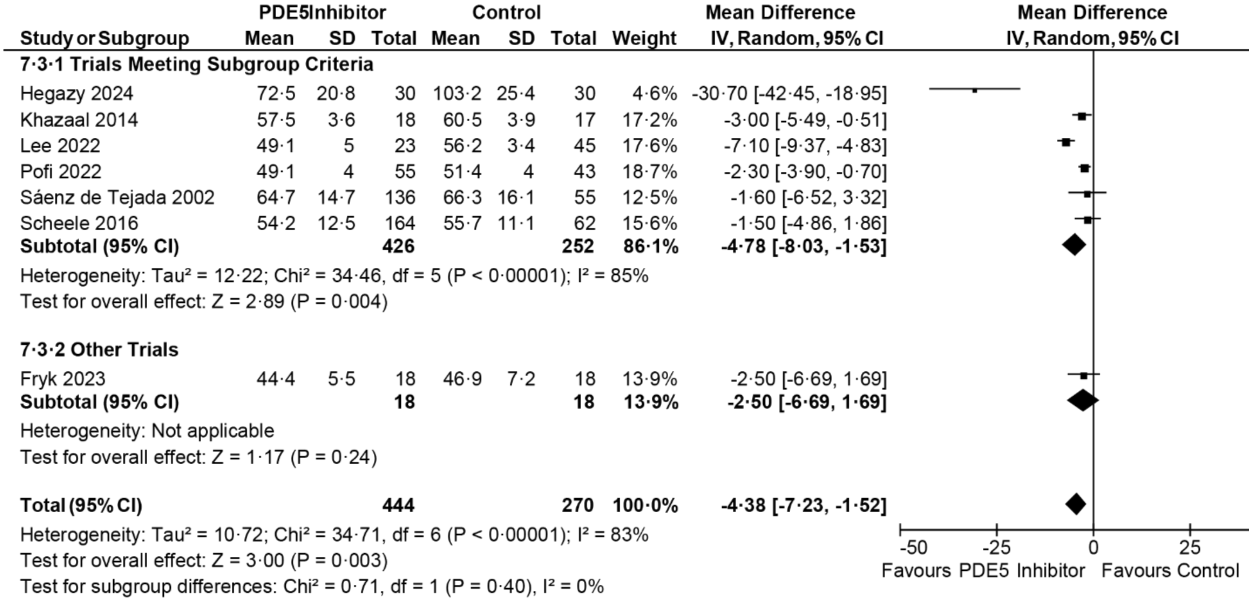

d)

Study or Subgroup	PDE5Inhibitor Mean	PDE5Inhibitor SD	PDE5Inhibitor Total	Control Mean	Control SD	Control Total	Weight	Mean Difference IV, Random, 95% CI
**7·4·1 Trials Meeting Subgroup Criteria**								
Derosa 2022	57·4	10·9	30	56·3	9·8	30	23·1%	1·10 [-4·15, 6·35]
Giannetta 2012	56	11·8	25	49·4	11·8	29	17·4%	6·60 [0·29, 12·91]
Kirilmaz 2015	66·1	16·4	41	61·8	19·7	42	12·3%	4·30 [-3·49, 12·09]
Morano 2007	63·9	27·3	8	47·5	16·4	8	1·7%	16·40 [-5·67, 38·47]
**Subtotal (95% CI)**			**104**			**109**	**54·5%**	**3·90 [0·32, 7·47]**
Heterogeneity: Tau² = 0·20; Chi² = 3·04, df = 3 (P = 0·39); I² = 1%								
Test for overall effect: Z = 2·14 (P = 0·03)								
**7·4·2 Other Trials**								
Grover-Páez 2007	58·4	28·1	20	60·4	2·6	20	5·3%	-2·00 [-14·37, 10·37]
Liu 2016	42·1	4·9	26	42·4	7·4	26	40·2%	-0·30 [-3·71, 3·11]
**Subtotal (95% CI)**			**46**			**46**	**45·5%**	**-0·42 [-3·71, 2·87]**
Heterogeneity: Tau² = 0·00; Chi² = 0·07, df = 1 (P = 0·80); I² = 0%								
Test for overall effect: Z = 0·25 (P = 0·80)								
**Total (95% CI)**			**150**			**155**	**100·0%**	**1·99 [-0·95, 4·93]**
Heterogeneity: Tau² = 2·57; Chi² = 6·15, df = 5 (P = 0·29); I² = 19%								
Test for overall effect: Z = 1·32 (P = 0·19)								
Test for subgroup differences: Chi² = 3·03, df = 1 (P = 0·08), I² = 67·0%								

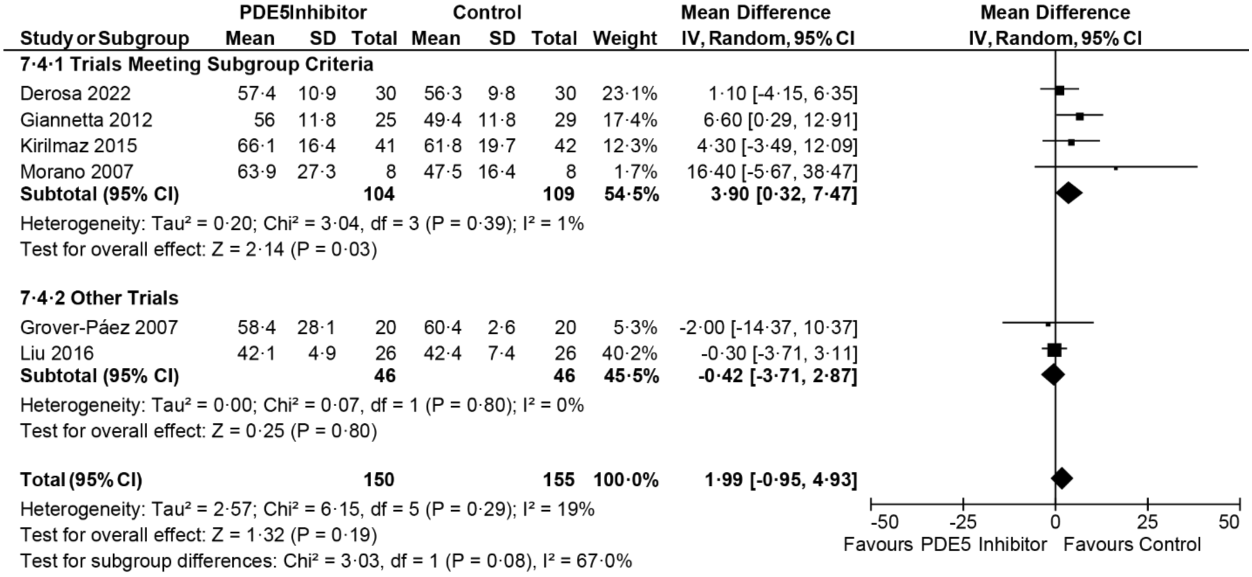

Supplementary Fig. 13: Sensitivity analysis of meta-analysis containing only trials with only participants with type 2 diabetes and a mean baseline HbA1c of at least 6.5% and with a treatment period of at least 8 weeks

Sensitivity analysis was performed on a potential decision to include trials which ran for at least 8 weeks and only included participants with type 2 diabetes and a mean baseline HbA1c of at least 6.5%, as well as on a potential decision to represent data in IFCC units (mmol/mol) or NGSP units (%). The following conditions were used for each meta-analysis: a) long half-life PDE5 inhibitors using NGSP units; b) short half-life PDE5 inhibitors using NGSP units; c) long half-life PDE5 inhibitors using IFCC units; d) short half-life PDE5 inhibitors using IFCC units.

a)

Study or Subgroup	PDE5Inhibitor Mean	SD	Total	Control Mean	SD	Total	Weight	Mean Difference IV, Random, 95% CI
**8·1·1 Trials Meeting Subgroup Criteria**								
Hegazy 2024	8·78	1·9	30	11·59	2·32	30	4·5%	-2·81 [-3·88, -1·74]
Khazaal 2014	7·41	0·33	18	7·69	0·35	17	17·3%	-0·28 [-0·51, -0·05]
Lee 2022	6·64	0·46	23	7·29	0·31	45	17·6%	-0·65 [-0·86, -0·44]
Pofi 2022	6·64	0·37	55	6·86	0·37	43	18·7%	-0·22 [-0·37, -0·07]
Scheele 2016	7·11	1·14	164	7·25	1·02	62	15·6%	-0·14 [-0·45, 0·17]
**Subtotal (95% CI)**			**290**			**197**	**73·7%**	**-0·50 [-0·83, -0·17]**
Heterogeneity: Tau² = 0·11; Chi² = 33·25, df = 4 (P < 0·00001); I² = 88%								
Test for overall effect: Z = 2·95 (P = 0·003)								
**8·1·2 Other Trials**								
Fryk 2023	6·21	0·51	18	6·44	0·66	18	13·9%	-0·23 [-0·62, 0·16]
Sáenz de Tejada 2002	8·07	1·34	136	8·21	1·48	55	12·4%	-0·14 [-0·59, 0·31]
**Subtotal (95% CI)**			**154**			**73**	**26·3%**	**-0·19 [-0·49, 0·10]**
Heterogeneity: Tau² = 0·00; Chi² = 0·09, df = 1 (P = 0·77); I² = 0%								
Test for overall effect: Z = 1·28 (P = 0·20)								
**Total (95% CI)**			**444**			**270**	**100·0%**	**-0·40 [-0·66, -0·14]**
Heterogeneity: Tau² = 0·09; Chi² = 34·27, df = 6 (P < 0·00001); I² = 82%								
Test for overall effect: Z = 3·03 (P = 0·002)								
Test for subgroup differences: Chi² = 1·86, df = 1 (P = 0·17), I² = 46·2%								

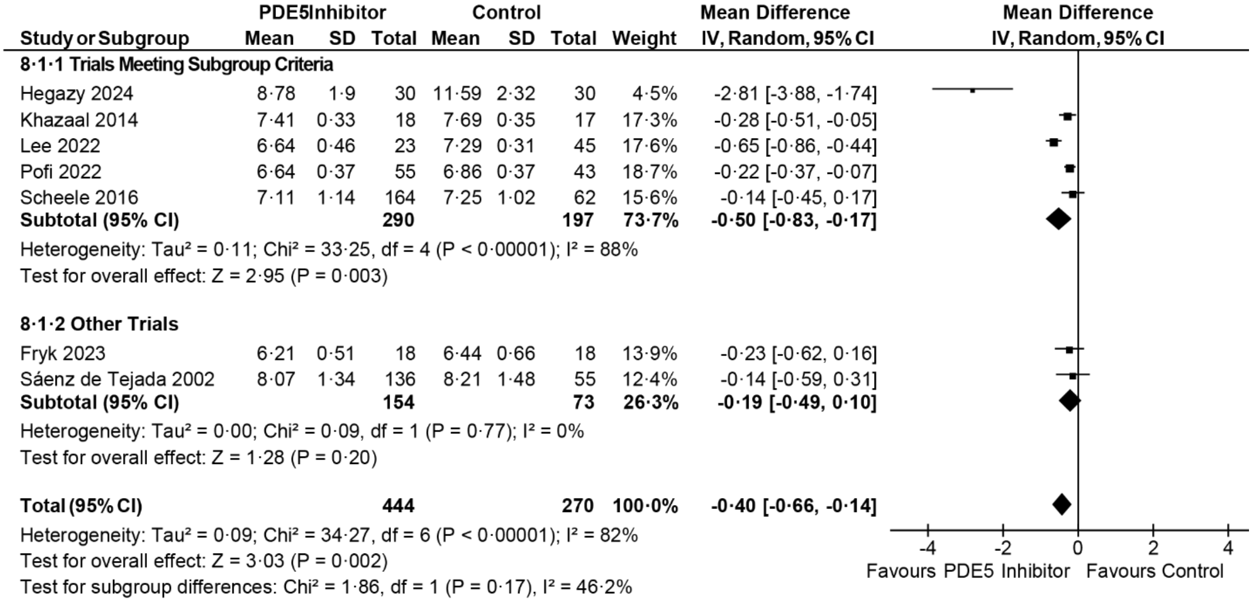

b)

Study or Subgroup	PDE5Inhibitor Mean	SD	Total	Control Mean	SD	Total	Weight	Mean Difference IV, Random, 95% CI
**8·2·1 Trials Meeting Subgroup Criteria**								
Derosa 2022	7·4	1	30	7·3	0·9	30	23·1%	0·10 [-0·38, 0·58]
Giannetta 2012	7·27	1·08	25	6·67	1·08	29	17·4%	0·60 [0·02, 1·18]
Kirilmaz 2015	8·2	1·5	41	7·8	1·8	42	12·3%	0·40 [-0·31, 1·11]
Morano 2007	8	2·5	8	6·5	1·5	8	1·7%	1·50 [-0·52, 3·52]
**Subtotal (95% CI)**			**104**			**109**	**54·6%**	**0·36 [0·03, 0·68]**
Heterogeneity: Tau² = 0·00; Chi² = 3·02, df = 3 (P = 0·39); I² = 1%								
Test for overall effect: Z = 2·14 (P = 0·03)								
**8·2·2 Other Trials**								
Grover-Páez 2007	7·49	2·57	20	7·67	0·24	20	5·3%	-0·18 [-1·31, 0·95]
Liu 2016	6	0·45	26	6·03	0·68	26	40·1%	-0·03 [-0·34, 0·28]
**Subtotal (95% CI)**			**46**			**46**	**45·4%**	**-0·04 [-0·34, 0·26]**
Heterogeneity: Tau² = 0·00; Chi² = 0·06, df = 1 (P = 0·80); I² = 0%								
Test for overall effect: Z = 0·26 (P = 0·79)								
**Total (95% CI)**			**150**			**155**	**100·0%**	**0·18 [-0·09, 0·45]**
Heterogeneity: Tau² = 0·02; Chi² = 6·15, df = 5 (P = 0·29); I² = 19%								
Test for overall effect: Z = 1·32 (P = 0·19)								
Test for subgroup differences: Chi² = 3·07, df = 1 (P = 0·08), I² = 67·4%								

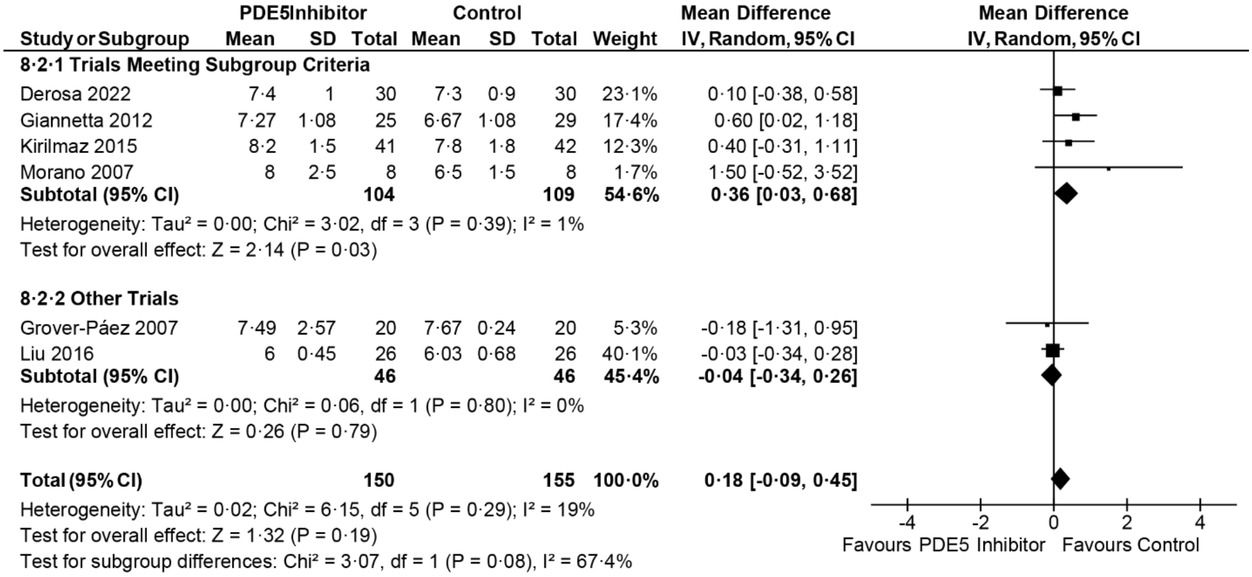

c)

Study or Subgroup	PDE5 Inhibitor Mean	SD	Total	Control Mean	SD	Total	Weight	Mean Difference IV, Random, 95% CI
**8·3·1 Trials Meeting Subgroup Criteria**								
Hegazy 2024	72·5	20·8	30	103·2	25·4	30	4·6%	-30·70 [-42·45, -18·95]
Khazaal 2014	57·5	3·6	18	60·5	3·9	17	17·2%	-3·00 [-5·49, -0·51]
Lee 2022	49·1	5	23	56·2	3·4	45	17·6%	-7·10 [-9·37, -4·83]
Pofi 2022	49·1	4	55	51·4	4	43	18·7%	-2·30 [-3·90, -0·70]
Scheele 2016	54·2	12·5	164	55·7	11·1	62	15·6%	-1·50 [-4·86, 1·86]
**Subtotal (95% CI)**			**290**			**197**	**73·6%**	**-5·44 [-9·10, -1·77]**
Heterogeneity: Tau² = 13·47; Chi² = 33·80, df = 4 (P < 0·00001); I² = 88%								
Test for overall effect: Z = 2·91 (P = 0·004)								
**8·3·2 Other Trials**								
Fryk 2023	44·4	5·5	18	46·9	7·2	18	13·9%	-2·50 [-6·69, 1·69]
Sáenz de Tejada 2002	64·7	14·7	136	66·3	16·1	55	12·5%	-1·60 [-6·52, 3·32]
**Subtotal (95% CI)**			**154**			**73**	**26·4%**	**-2·12 [-5·31, 1·07]**
Heterogeneity: Tau² = 0·00; Chi² = 0·07, df = 1 (P = 0·78); I² = 0%								
Test for overall effect: Z = 1·30 (P = 0·19)								
**Total (95% CI)**			**444**			**270**	**100·0%**	**-4·38 [-7·23, -1·52]**
Heterogeneity: Tau² = 10·72; Chi² = 34·71, df = 6 (P < 0·00001); I² = 83%								
Test for overall effect: Z = 3·00 (P = 0·003)								
Test for subgroup differences: Chi² = 1·79, df = 1 (P = 0·18), I² = 44·1%								

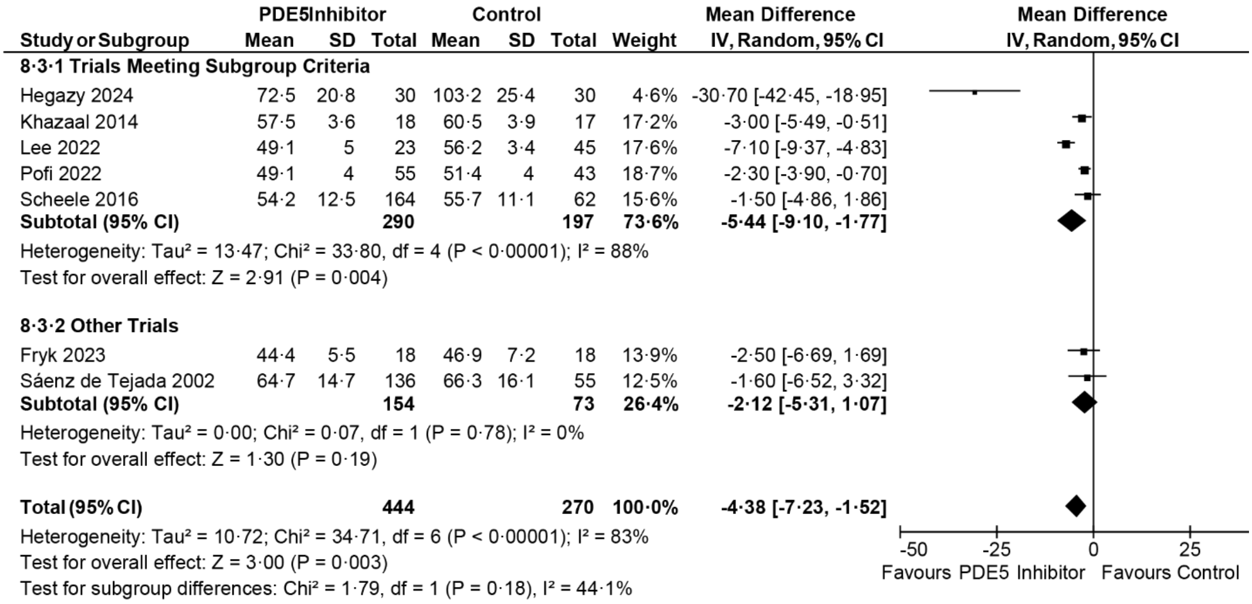

d)

Study or Subgroup	PDE5 Inhibitor Mean	SD	Total	Control Mean	SD	Total	Weight	Mean Difference IV, Random, 95% CI
**8·4·1 Trials Meeting Subgroup Criteria**								
Derosa 2022	57·4	10·9	30	56·3	9·8	30	23·1%	1·10 [-4·15, 6·35]
Giannetta 2012	56	11·8	25	49·4	11·8	29	17·4%	6·60 [0·29, 12·91]
Kirilmaz 2015	66·1	16·4	41	61·8	19·7	42	12·3%	4·30 [-3·49, 12·09]
Morano 2007	63·9	27·3	8	47·5	16·4	8	1·7%	16·40 [-5·67, 38·47]
**Subtotal (95% CI)**			**104**			**109**	**54·5%**	**3·90 [0·32, 7·47]**
Heterogeneity: Tau² = 0·20; Chi² = 3·04, df = 3 (P = 0·39); I² = 1%								
Test for overall effect: Z = 2·14 (P = 0·03)								
**8·4·2 Other Trials**								
Grover-Páez 2007	58·4	28·1	20	60·4	2·6	20	5·3%	-2·00 [-14·37, 10·37]
Liu 2016	42·1	4·9	26	42·4	7·4	26	40·2%	-0·30 [-3·71, 3·11]
**Subtotal (95% CI)**			**46**			**46**	**45·5%**	**-0·42 [-3·71, 2·87]**
Heterogeneity: Tau² = 0·00; Chi² = 0·07, df = 1 (P = 0·80); I² = 0%								
Test for overall effect: Z = 0·25 (P = 0·80)								
**Total (95% CI)**			**150**			**155**	**100·0%**	**1·99 [-0·95, 4·93]**
Heterogeneity: Tau² = 2·57; Chi² = 6·15, df = 5 (P = 0·29); I² = 19%								
Test for overall effect: Z = 1·32 (P = 0·19)								
Test for subgroup differences: Chi² = 3·03, df = 1 (P = 0·08), I² = 67·0%								

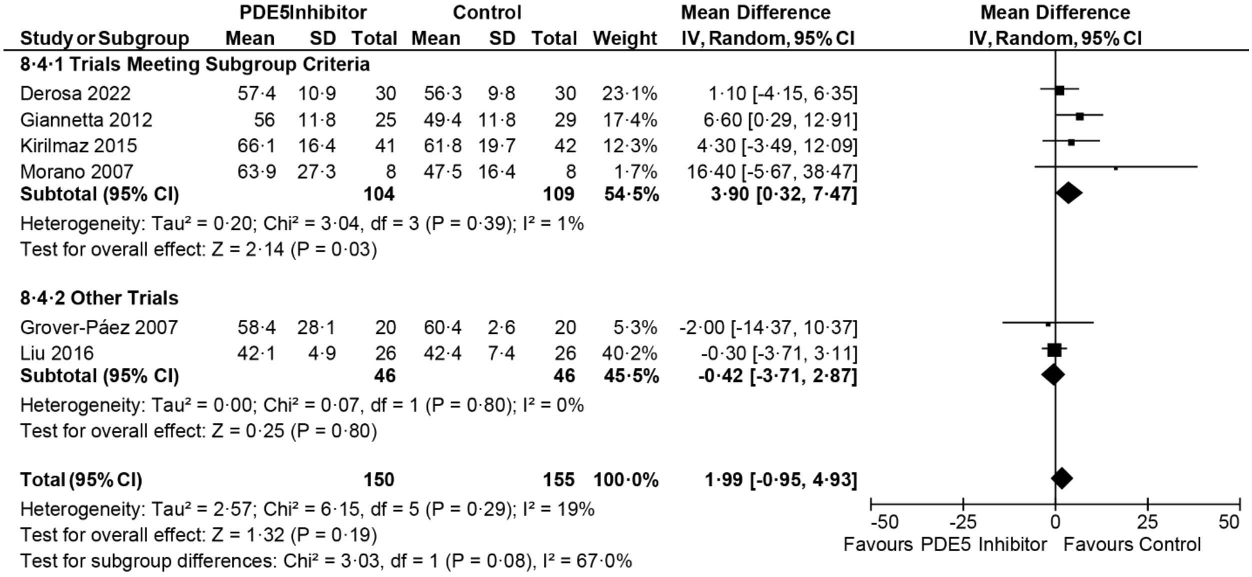

Supplementary Fig. 14: Sensitivity analysis of meta-analysis containing all trials, using lowest dosage data

Sensitivity analysis was performed on a potential decision to include all trials while only including data from participants that took the lowest possible dose, as well as on a potential decision to represent data in IFCC units (mmol/mol) or NGSP units (%). Only one trial had multiple dosages and was thus eligible for such a decision. The following conditions were used for each meta-analysis: a) long half-life PDE5 inhibitors using NGSP units; b) short half-life PDE5 inhibitors using NGSP units; c) long half-life PDE5 inhibitors using IFCC units; d) short half-life PDE5 inhibitors using IFCC units.

a)

	PDE5Inhibitor			Control				Mean Difference
**Study or Subgroup**	**Mean**	**SD**	**Total**	**Mean**	**SD**	**Total**	**Weight**	**IV, Random, 95% CI**
Fryk 2023	6·21	0·51	18	6·44	0·66	18	13·9%	-0·23 [-0·62, 0·16]
Hegazy 2024	8·78	1·9	30	11·59	2·32	30	4·6%	-2·81 [-3·88, -1·74]
Khazaal 2014	7·41	0·33	18	7·69	0·35	17	17·4%	-0·28 [-0·51, -0·05]
Lee 2022	6·64	0·46	23	7·29	0·31	45	17·7%	-0·65 [-0·86, -0·44]
Pofi 2022	6·64	0·37	55	6·86	0·37	43	18·7%	-0·22 [-0·37, -0·07]
Sáenz de Tejada 2002	8·11	1·2	73	8·21	1·48	55	12·0%	-0·10 [-0·58, 0·38]
Scheele 2016	7·11	1·14	164	7·25	1·02	62	15·6%	-0·14 [-0·45, 0·17]
**Total (95% CI)**			**381**			**270**	**100·0%**	**-0·40 [-0·66, -0·14]**

Heterogeneity: Tau² = 0·09; Chi² = 34·48, df = 6 (P < 0·00001); I² = 83%
Test for overall effect: Z = 2·99 (P = 0·003)

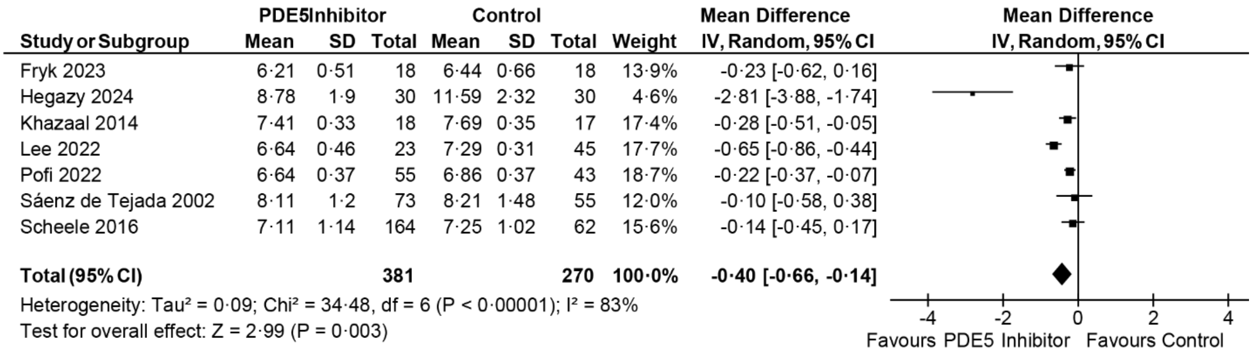

b)

	PDE5Inhibitor			Control				Mean Difference
**Study or Subgroup**	**Mean**	**SD**	**Total**	**Mean**	**SD**	**Total**	**Weight**	**IV, Random, 95% CI**
Derosa 2022	7·4	1	30	7·3	0·9	30	23·1%	0·10 [-0·38, 0·58]
Giannetta 2012	7·27	1·08	25	6·67	1·08	29	17·4%	0·60 [0·02, 1·18]
Grover-Páez 2007	7·49	2·57	20	7·67	0·24	20	5·3%	-0·18 [-1·31, 0·95]
Kirilmaz 2015	8·2	1·5	41	7·8	1·8	42	12·3%	0·40 [-0·31, 1·11]
Liu 2016	6	0·45	26	6·03	0·68	26	40·1%	-0·03 [-0·34, 0·28]
Morano 2007	8	2·5	8	6·5	1·5	8	1·7%	1·50 [-0·52, 3·52]
**Total (95% CI)**			**150**			**155**	**100·0%**	**0·18 [-0·09, 0·45]**

Heterogeneity: Tau² = 0·02; Chi² = 6·15, df = 5 (P = 0·29); I² = 19%
Test for overall effect: Z = 1·32 (P = 0·19)

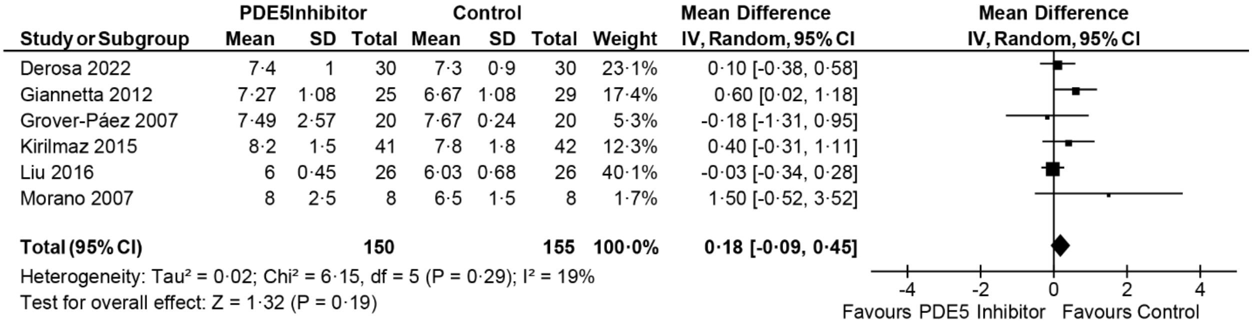

c)

	PDE5Inhibitor			Control				Mean Difference
**Study or Subgroup**	**Mean**	**SD**	**Total**	**Mean**	**SD**	**Total**	**Weight**	**IV, Random, 95% CI**
Fryk 2023	44·4	5·5	18	46·9	7·2	18	14·0%	-2·50 [-6·69, 1·69]
Hegazy 2024	72·5	20·8	30	103·2	25·4	30	4·6%	-30·70 [-42·45, -18·95]
Khazaal 2014	57·5	3·6	18	60·5	3·9	17	17·3%	-3·00 [-5·49, -0·51]
Lee 2022	49·1	5	23	56·2	3·4	45	17·7%	-7·10 [-9·37, -4·83]
Pofi 2022	49·1	4	55	51·4	4	43	18·7%	-2·30 [-3·90, -0·70]
Sáenz de Tejada 2002	65·1	13·1	73	66·3	16·1	55	12·0%	-1·20 [-6·41, 4·01]
Scheele 2016	54·2	12·5	164	55·7	11·1	62	15·6%	-1·50 [-4·86, 1·86]
**Total (95% CI)**			**381**			**270**	**100·0%**	**-4·35 [-7·23, -1·47]**

Heterogeneity: Tau² = 10·87; Chi² = 34·89, df = 6 (P < 0·00001); I² = 83%
Test for overall effect: Z = 2·96 (P = 0·003)

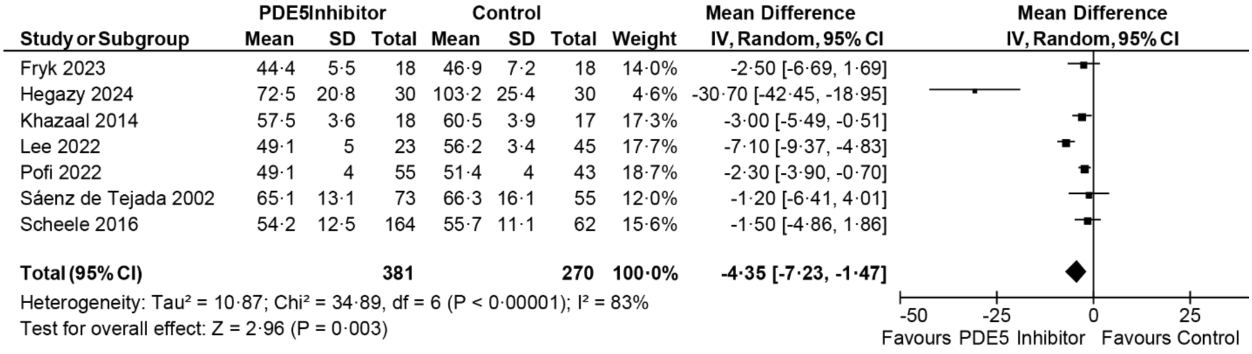

d)

Study or Subgroup	PDE5 Inhibitor Mean	PDE5 Inhibitor SD	PDE5 Inhibitor Total	Control Mean	Control SD	Control Total	Weight	Mean Difference IV, Random, 95% CI
Derosa 2022	57·4	10·9	30	56·3	9·8	30	23·1%	1·10 [-4·15, 6·35]
Giannetta 2012	56	11·8	25	49·4	11·8	29	17·4%	6·60 [0·29, 12·91]
Grover-Páez 2007	58·4	28·1	20	60·4	2·6	20	5·3%	-2·00 [-14·37, 10·37]
Kirilmaz 2015	66·1	16·4	41	61·8	19·7	42	12·3%	4·30 [-3·49, 12·09]
Liu 2016	42·1	4·9	26	42·4	7·4	26	40·2%	-0·30 [-3·71, 3·11]
Morano 2007	63·9	27·3	8	47·5	16·4	8	1·7%	16·40 [-5·67, 38·47]
**Total (95% CI)**			**150**			**155**	**100·0%**	**1·99 [-0·95, 4·93]**

Heterogeneity: Tau² = 2·57; Chi² = 6·15, df = 5 (P = 0·29); I² = 19%

Test for overall effect: Z = 1·32 (P = 0·19)

Mean Difference IV, Random, 95% CI

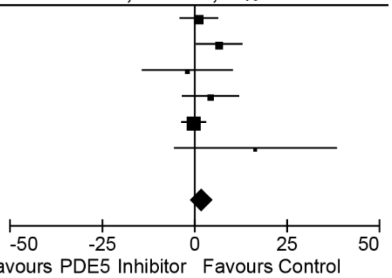

Supplementary Fig. 15: Sensitivity analysis of meta-analysis containing all trials, using highest dosage data

Sensitivity analysis was performed on a potential decision to include all trials while only including data from participants that took the highest possible dose, as well as on a potential decision to represent data in IFCC units (mmol/mol) or NGSP units (%). Only one trial had multiple dosages and was thus eligible for such a decision. The following conditions were used for each meta-analysis: a) long half-life PDE5 inhibitors using NGSP units; b) short half-life PDE5 inhibitors using NGSP units; c) long half-life PDE5 inhibitors using IFCC units; d) short half-life PDE5 inhibitors using IFCC units.

a)

	PDE5Inhibitor			Control				Mean Difference
**Study or Subgroup**	**Mean**	**SD**	**Total**	**Mean**	**SD**	**Total**	**Weight**	**IV, Random, 95% CI**
Fryk 2023	6·21	0·51	18	6·44	0·66	18	14·2%	-0·23 [-0·62, 0·16]
Hegazy 2024	8·78	1·9	30	11·59	2·32	30	5·0%	-2·81 [-3·88, -1·74]
Khazaal 2014	7·41	0·33	18	7·69	0·35	17	17·3%	-0·28 [-0·51, -0·05]
Lee 2022	6·64	0·46	23	7·29	0·31	45	17·6%	-0·65 [-0·86, -0·44]
Pofi 2022	6·64	0·37	55	6·86	0·37	43	18·5%	-0·22 [-0·37, -0·07]
Sáenz de Tejada 2002	8·43	1·47	72	8·21	1·48	55	11·6%	0·22 [-0·30, 0·74]
Scheele 2016	7·11	1·14	164	7·25	1·02	62	15·8%	-0·14 [-0·45, 0·17]
**Total (95% CI)**			**380**			**270**	**100·0%**	**-0·37 [-0·65, -0·10]**

Heterogeneity: Tau² = 0·10; Chi² = 37·87, df = 6 (P < 0·00001); I² = 84%
Test for overall effect: Z = 2·64 (P = 0·008)

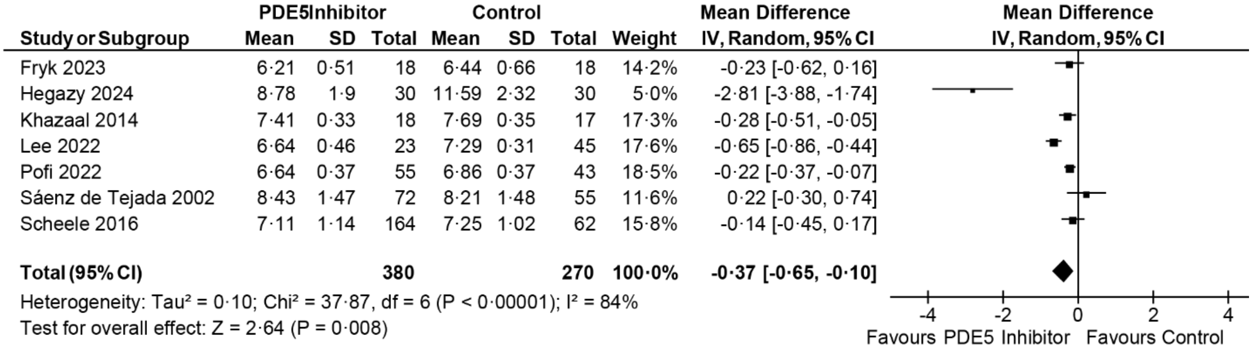

b)

	PDE5Inhibitor			Control				Mean Difference
**Study or Subgroup**	**Mean**	**SD**	**Total**	**Mean**	**SD**	**Total**	**Weight**	**IV, Random, 95% CI**
Derosa 2022	7·4	1	30	7·3	0·9	30	23·1%	0·10 [-0·38, 0·58]
Giannetta 2012	7·27	1·08	25	6·67	1·08	29	17·4%	0·60 [0·02, 1·18]
Grover-Páez 2007	7·49	2·57	20	7·67	0·24	20	5·3%	-0·18 [-1·31, 0·95]
Kirilmaz 2015	8·2	1·5	41	7·8	1·8	42	12·3%	0·40 [-0·31, 1·11]
Liu 2016	6	0·45	26	6·03	0·68	26	40·1%	-0·03 [-0·34, 0·28]
Morano 2007	8	2·5	8	6·5	1·5	8	1·7%	1·50 [-0·52, 3·52]
**Total (95% CI)**			**150**			**155**	**100·0%**	**0·18 [-0·09, 0·45]**

Heterogeneity: Tau² = 0·02; Chi² = 6·15, df = 5 (P = 0·29); I² = 19%
Test for overall effect: Z = 1·32 (P = 0·19)

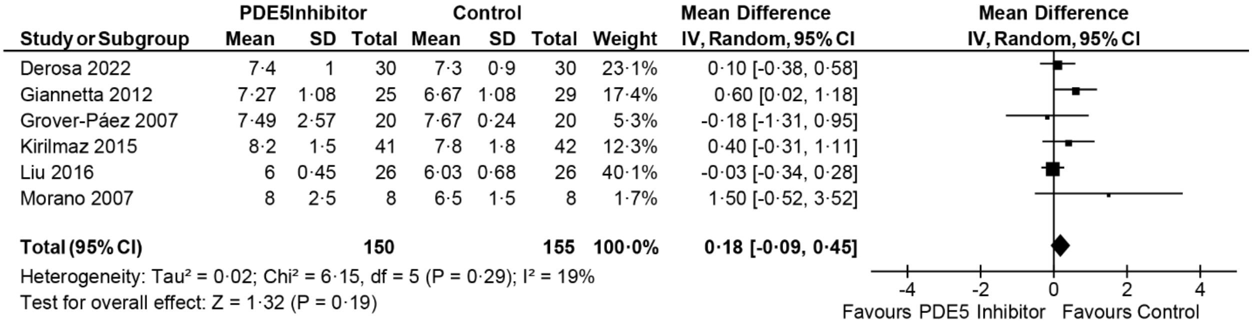

c)

	PDE5Inhibitor			Control				Mean Difference
**Study or Subgroup**	**Mean**	**SD**	**Total**	**Mean**	**SD**	**Total**	**Weight**	**IV, Random, 95% CI**
Fryk 2023	44·4	5·5	18	46·9	7·2	18	14·2%	-2·50 [-6·69, 1·69]
Hegazy 2024	72·5	20·8	30	103·2	25·4	30	5·0%	-30·70 [-42·45, -18·95]
Khazaal 2014	57·5	3·6	18	60·5	3·9	17	17·3%	-3·00 [-5·49, -0·51]
Lee 2022	49·1	5	23	56·2	3·4	45	17·6%	-7·10 [-9·37, -4·83]
Pofi 2022	49·1	4	55	51·4	4	43	18·5%	-2·30 [-3·90, -0·70]
Sáenz de Tejada 2002	68·7	16	72	66·3	16·1	55	11·7%	2·40 [-3·24, 8·04]
Scheele 2016	54·2	12·5	164	55·7	11·1	62	15·8%	-1·50 [-4·86, 1·86]
**Total (95% CI)**			**380**			**270**	**100·0%**	**-4·03 [-7·07, -1·00]**

Heterogeneity: Tau² = 12·28; Chi² = 38·33, df = 6 (P < 0·00001); I² = 84%
Test for overall effect: Z = 2·60 (P = 0·009)

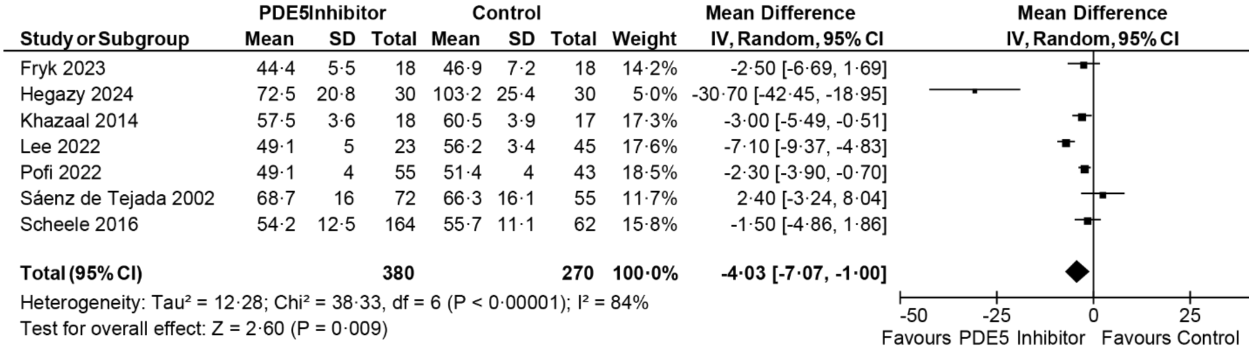

d)

Study or Subgroup	PDE5 Inhibitor Mean	PDE5 Inhibitor SD	PDE5 Inhibitor Total	Control Mean	Control SD	Control Total	Weight	Mean Difference IV, Random, 95% CI
Derosa 2022	57·4	10·9	30	56·3	9·8	30	23·1%	1·10 [-4·15, 6·35]
Giannetta 2012	56	11·8	25	49·4	11·8	29	17·4%	6·60 [0·29, 12·91]
Grover-Páez 2007	58·4	28·1	20	60·4	2·6	20	5·3%	-2·00 [-14·37, 10·37]
Kirilmaz 2015	66·1	16·4	41	61·8	19·7	42	12·3%	4·30 [-3·49, 12·09]
Liu 2016	42·1	4·9	26	42·4	7·4	26	40·2%	-0·30 [-3·71, 3·11]
Morano 2007	63·9	27·3	8	47·5	16·4	8	1·7%	16·40 [-5·67, 38·47]
**Total (95% CI)**			**150**			**155**	**100·0%**	**1·99 [-0·95, 4·93]**

Heterogeneity: Tau² = 2·57; Chi² = 6·15, df = 5 (P = 0·29); I² = 19%

Test for overall effect: Z = 1·32 (P = 0·19)

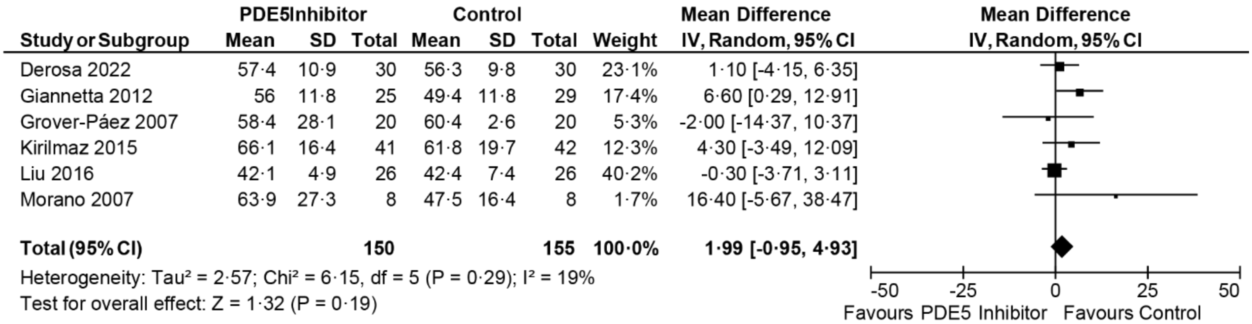

Supplementary Fig. 16: Sensitivity analysis of meta-analysis containing all trials, using shorter time period data

Sensitivity analysis was performed on a potential decision to include all trials while only including data taken from a shorter time period, as well as on a potential decision to represent data in IFCC units (mmol/mol) or NGSP units (%). Only one trial had multiple dosages and was thus eligible for such a decision. The following conditions were used for each meta-analysis: a) long half-life PDE5 inhibitors using NGSP units; b) short half-life PDE5 inhibitors using NGSP units; c) long half-life PDE5 inhibitors using IFCC units; d) short half-life PDE5 inhibitors using IFCC units.

a)

	PDE5Inhibitor			Control				Mean Difference
**Study or Subgroup**	**Mean**	**SD**	**Total**	**Mean**	**SD**	**Total**	**Weight**	**IV, Random, 95% CI**
Fryk 2023	6·21	0·51	18	6·44	0·66	18	13·7%	-0·23 [-0·62, 0·16]
Hegazy 2024	8·78	1·9	30	11·59	2·32	30	4·4%	-2·81 [-3·88, -1·74]
Khazaal 2014	7·41	0·33	18	7·69	0·35	17	17·2%	-0·28 [-0·51, -0·05]
Lee 2022	6·67	0·43	45	7·29	0·31	45	18·5%	-0·62 [-0·77, -0·47]
Pofi 2022	6·64	0·37	55	6·86	0·37	43	18·6%	-0·22 [-0·37, -0·07]
Sáenz de Tejada 2002	8·07	1·34	136	8·21	1·48	55	12·3%	-0·14 [-0·59, 0·31]
Scheele 2016	7·11	1·14	164	7·25	1·02	62	15·4%	-0·14 [-0·45, 0·17]
**Total (95% CI)**			**466**			**270**	**100·0%**	**-0·40 [-0·65, -0·14]**

Heterogeneity: Tau² = 0·09; Chi² = 38·08, df = 6 (P < 0·00001); I² = 84%
Test for overall effect: Z = 3·04 (P = 0·002)

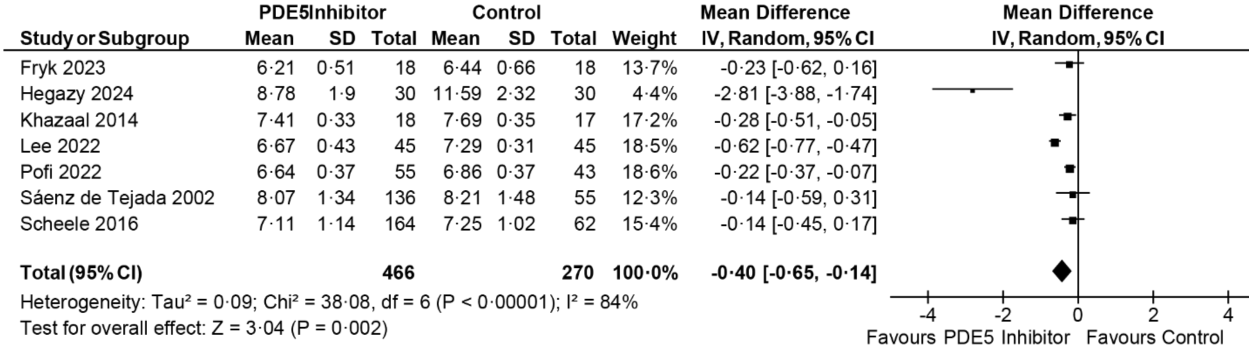

b)

	PDE5Inhibitor			Control				Mean Difference
**Study or Subgroup**	**Mean**	**SD**	**Total**	**Mean**	**SD**	**Total**	**Weight**	**IV, Random, 95% CI**
Derosa 2022	7·4	1	30	7·3	0·9	30	23·1%	0·10 [-0·38, 0·58]
Giannetta 2012	7·27	1·08	25	6·67	1·08	29	17·4%	0·60 [0·02, 1·18]
Grover-Páez 2007	7·49	2·57	20	7·67	0·24	20	5·3%	-0·18 [-1·31, 0·95]
Kirilmaz 2015	8·2	1·5	41	7·8	1·8	42	12·3%	0·40 [-0·31, 1·11]
Liu 2016	6	0·45	26	6·03	0·68	26	40·1%	-0·03 [-0·34, 0·28]
Morano 2007	8	2·5	8	6·5	1·5	8	1·7%	1·50 [-0·52, 3·52]
**Total (95% CI)**			**150**			**155**	**100·0%**	**0·18 [-0·09, 0·45]**

Heterogeneity: Tau² = 0·02; Chi² = 6·15, df = 5 (P = 0·29); I² = 19%
Test for overall effect: Z = 1·32 (P = 0·19)

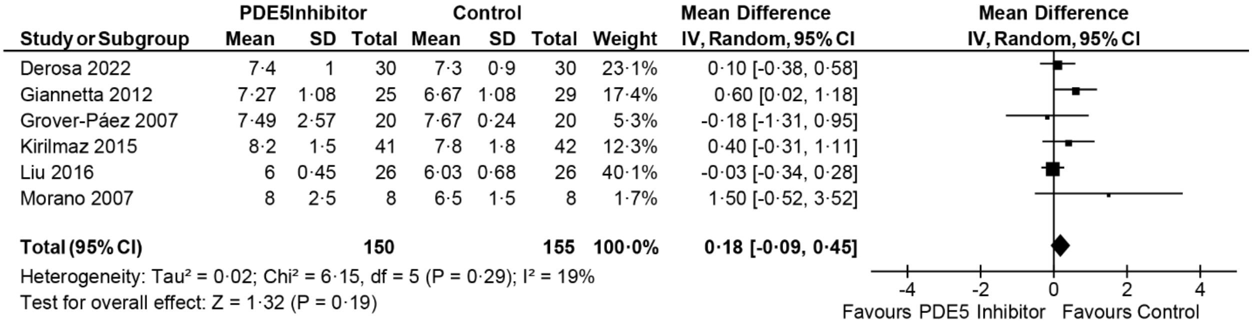

c)

	PDE5Inhibitor			Control				Mean Difference
**Study or Subgroup**	**Mean**	**SD**	**Total**	**Mean**	**SD**	**Total**	**Weight**	**IV, Random, 95% CI**
Fryk 2023	44·4	5·5	18	46·9	7·2	18	13·8%	-2·50 [-6·69, 1·69]
Hegazy 2024	72·5	20·8	30	103·2	25·4	30	4·5%	-30·70 [-42·45, -18·95]
Khazaal 2014	57·5	3·6	18	60·5	3·9	17	17·1%	-3·00 [-5·49, -0·51]
Lee 2022	49·4	4·7	45	56·2	3·4	45	18·4%	-6·80 [-8·49, -5·11]
Pofi 2022	49·1	4	55	51·4	4	43	18·5%	-2·30 [-3·90, -0·70]
Sáenz de Tejada 2002	64·7	14·7	136	66·3	16·1	55	12·3%	-1·60 [-6·52, 3·32]
Scheele 2016	54·2	12·5	164	55·7	11·1	62	15·4%	-1·50 [-4·86, 1·86]
**Total (95% CI)**			**466**			**270**	**100·0%**	**-4·34 [-7·16, -1·51]**

Heterogeneity: Tau² = 10·56; Chi² = 38·82, df = 6 (P < 0·00001); I² = 85%
Test for overall effect: Z = 3·01 (P = 0·003)

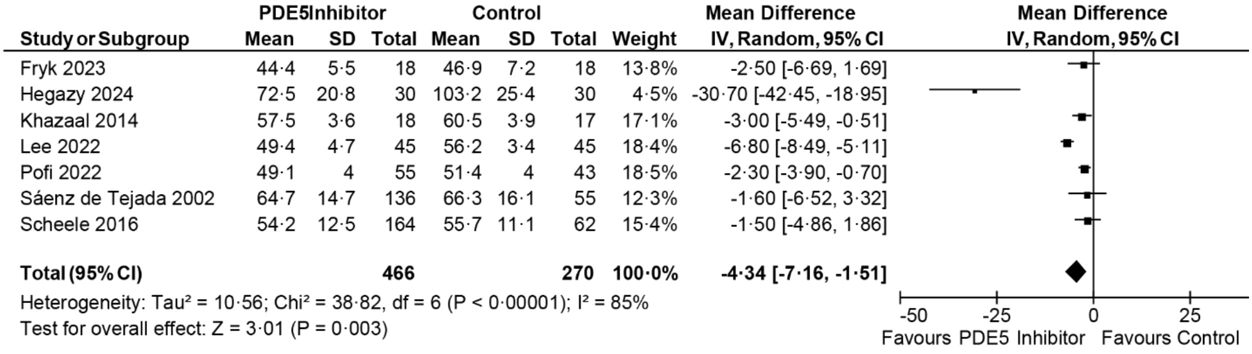

d)

Study or Subgroup	PDE5Inhibitor Mean	SD	Total	Control Mean	SD	Total	Weight	Mean Difference IV, Random, 95% CI
Derosa 2022	57·4	10·9	30	56·3	9·8	30	23·1%	1·10 [-4·15, 6·35]
Giannetta 2012	56	11·8	25	49·4	11·8	29	17·4%	6·60 [0·29, 12·91]
Grover-Páez 2007	58·4	28·1	20	60·4	2·6	20	5·3%	-2·00 [-14·37, 10·37]
Kirilmaz 2015	66·1	16·4	41	61·8	19·7	42	12·3%	4·30 [-3·49, 12·09]
Liu 2016	42·1	4·9	26	42·4	7·4	26	40·2%	-0·30 [-3·71, 3·11]
Morano 2007	63·9	27·3	8	47·5	16·4	8	1·7%	16·40 [-5·67, 38·47]
**Total (95% CI)**			**150**			**155**	**100·0%**	**1·99 [-0·95, 4·93]**

Heterogeneity: Tau² = 2·57; Chi² = 6·15, df = 5 (P = 0·29); I² = 19%

Test for overall effect: Z = 1·32 (P = 0·19)

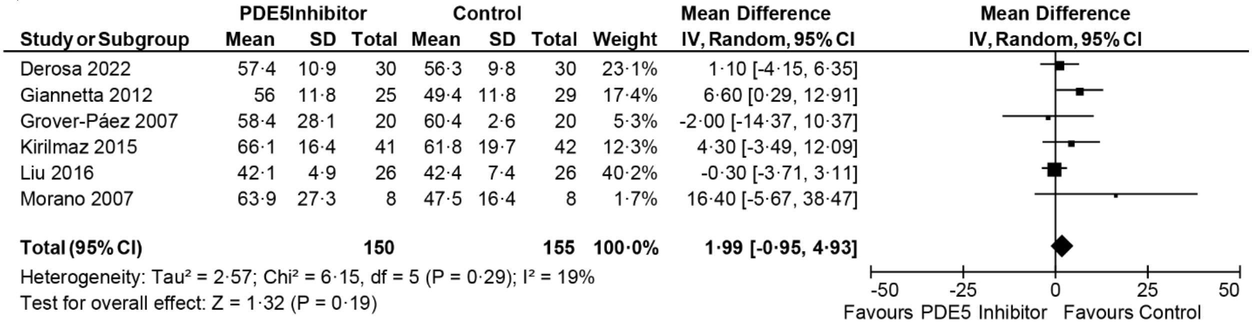

Supplementary Fig. 17: Sensitivity analysis of meta-analysis grouped by intervention

Sensitivity analysis was performed on a potential decision to include all trials grouped by the same pharmaceutical intervention, as well as on a potential decision to represent data in IFCC units (mmol/mol) or NGSP units (%). The following conditions were used for each meta-analysis: a) long half-life PDE5 inhibitors using NGSP units; b) short half-life PDE5 inhibitors using NGSP units; c) long half-life PDE5 inhibitors using IFCC units; d) short half-life PDE5 inhibitors using IFCC units.

a)

	PDE5Inhibitor			Control				Mean Difference
**Study or Subgroup**	**Mean**	**SD**	**Total**	**Mean**	**SD**	**Total**	**Weight**	**IV, Random, 95% CI**
**12·1·1 Tadalafil**								
Fryk 2023	6·21	0·51	18	6·44	0·66	18	13·9%	-0·23 [-0·62, 0·16]
Hegazy 2024	8·78	1·9	30	11·59	2·32	30	4·5%	-2·81 [-3·88, -1·74]
Khazaal 2014	7·41	0·33	18	7·69	0·35	17	17·3%	-0·28 [-0·51, -0·05]
Lee 2022	6·64	0·46	23	7·29	0·31	45	17·6%	-0·65 [-0·86, -0·44]
Pofi 2022	6·64	0·37	55	6·86	0·37	43	18·7%	-0·22 [-0·37, -0·07]
Sáenz de Tejada 2002	8·07	1·34	136	8·21	1·48	55	12·4%	-0·14 [-0·59, 0·31]
**Subtotal (95% CI)**			**280**			**208**	**84·4%**	**-0·46 [-0·76, -0·16]**
Heterogeneity: Tau² = 0·10; Chi² = 32·68, df = 5 (P < 0·00001); I² = 85%								
Test for overall effect: Z = 3·01 (P = 0·003)								
**12·1·2 PF-00489791**								
Scheele 2016	7·11	1·14	164	7·25	1·02	62	15·6%	-0·14 [-0·45, 0·17]
**Subtotal (95% CI)**			**164**			**62**	**15·6%**	**-0·14 [-0·45, 0·17]**
Heterogeneity: Not applicable								
Test for overall effect: Z = 0·89 (P = 0·37)								
**Total (95% CI)**			**444**			**270**	**100·0%**	**-0·40 [-0·66, -0·14]**
Heterogeneity: Tau² = 0·09; Chi² = 34·27, df = 6 (P < 0·00001); I² = 82%								
Test for overall effect: Z = 3·03 (P = 0·002)								
Test for subgroup differences: Chi² = 2·14, df = 1 (P = 0·14), I² = 53·3%								

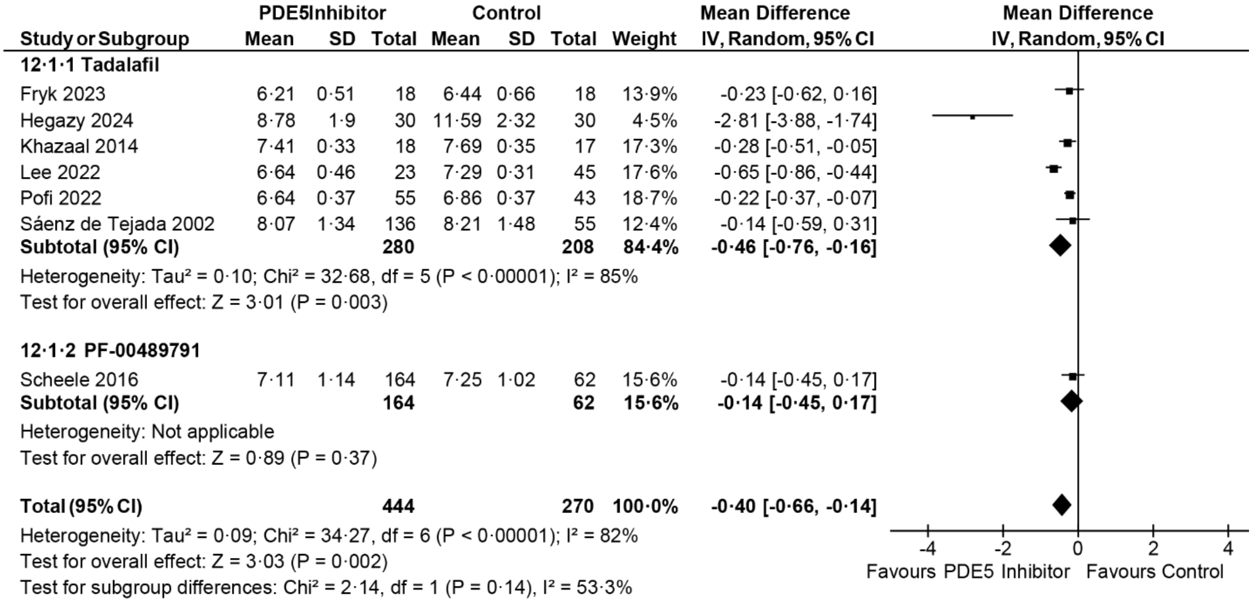

b)

	PDE5Inhibitor			Control				Mean Difference
**Study or Subgroup**	**Mean**	**SD**	**Total**	**Mean**	**SD**	**Total**	**Weight**	**IV, Random, 95% CI**
**12·2·1 Sildenafil**								
Giannetta 2012	7·27	1·08	25	6·67	1·08	29	17·4%	0·60 [0·02, 1·18]
Grover-Páez 2007	7·49	2·57	20	7·67	0·24	20	5·3%	-0·18 [-1·31, 0·95]
Kirilmaz 2015	8·2	1·5	41	7·8	1·8	42	12·3%	0·40 [-0·31, 1·11]
Liu 2016	6	0·45	26	6·03	0·68	26	40·1%	-0·03 [-0·34, 0·28]
Morano 2007	8	2·5	8	6·5	1·5	8	1·7%	1·50 [-0·52, 3·52]
**Subtotal (95% CI)**			**120**			**125**	**76·9%**	**0·25 [-0·13, 0·63]**
Heterogeneity: Tau² = 0·06; Chi² = 6·11, df = 4 (P = 0·19); I² = 35%								
Test for overall effect: Z = 1·28 (P = 0·20)								
**12·2·2 Avanafil**								
Derosa 2022	7·4	1	30	7·3	0·9	30	23·1%	0·10 [-0·38, 0·58]
**Subtotal (95% CI)**			**30**			**30**	**23·1%**	**0·10 [-0·38, 0·58]**
Heterogeneity: Not applicable								
Test for overall effect: Z = 0·41 (P = 0·68)								
**Total (95% CI)**			**150**			**155**	**100·0%**	**0·18 [-0·09, 0·45]**
Heterogeneity: Tau² = 0·02; Chi² = 6·15, df = 5 (P = 0·29); I² = 19%								
Test for overall effect: Z = 1·32 (P = 0·19)								
Test for subgroup differences: Chi² = 0·22, df = 1 (P = 0·64), I² = 0%								

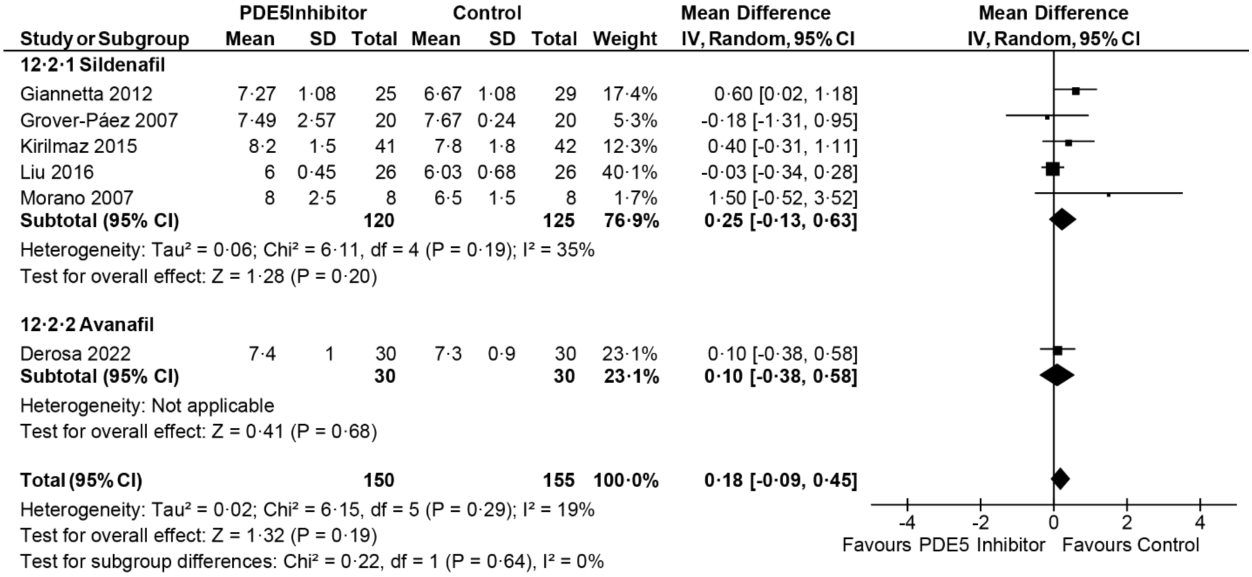

c)

Study or Subgroup	PDE5Inhibitor Mean	SD	Total	Control Mean	SD	Total	Weight	Mean Difference IV, Random, 95% CI
**12·3·1 Tadalafil**								
Fryk 2023	44·4	5·5	18	46·9	7·2	18	13·9%	-2·50 [-6·69, 1·69]
Hegazy 2024	72·5	20·8	30	103·2	25·4	30	4·6%	-30·70 [-42·45, -18·95]
Khazaal 2014	57·5	3·6	18	60·5	3·9	17	17·2%	-3·00 [-5·49, -0·51]
Lee 2022	49·1	5	23	56·2	3·4	45	17·6%	-7·10 [-9·37, -4·83]
Pofi 2022	49·1	4	55	51·4	4	43	18·7%	-2·30 [-3·90, -0·70]
Sáenz de Tejada 2002	64·7	14·7	136	66·3	16·1	55	12·5%	-1·60 [-6·52, 3·32]
**Subtotal (95% CI)**			**280**			**208**	**84·4%**	**-5·02 [-8·31, -1·72]**
Heterogeneity: Tau² = 12·38; Chi² = 33·16, df = 5 (P < 0·00001); I² = 85%								
Test for overall effect: Z = 2·99 (P = 0·003)								
**12·3·2 PF-00489791**								
Scheele 2016	54·2	12·5	164	55·7	11·1	62	15·6%	-1·50 [-4·86, 1·86]
**Subtotal (95% CI)**			**164**			**62**	**15·6%**	**-1·50 [-4·86, 1·86]**
Heterogeneity: Not applicable								
Test for overall effect: Z = 0·87 (P = 0·38)								
**Total (95% CI)**			**444**			**270**	**100·0%**	**-4·38 [-7·23, -1·52]**
Heterogeneity: Tau² = 10·72; Chi² = 34·71, df = 6 (P < 0·00001); I² = 83%								
Test for overall effect: Z = 3·00 (P = 0·003)								
Test for subgroup differences: Chi² = 2·14, df = 1 (P = 0·14), I² = 53·4%								

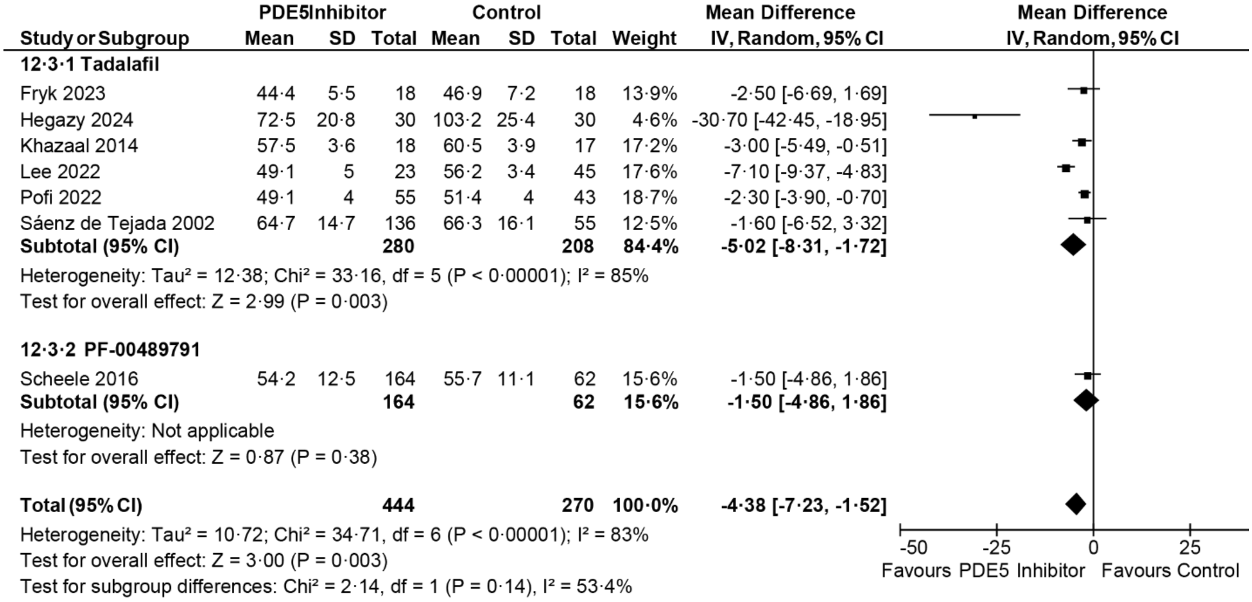

d)

Study or Subgroup	PDE5Inhibitor Mean	SD	Total	Control Mean	SD	Total	Weight	Mean Difference IV, Random, 95% CI
**12·4·1 Sildenafil**								
Giannetta 2012	56	11·8	25	49·4	11·8	29	17·4%	6·60 [0·29, 12·91]
Grover-Páez 2007	58·4	28·1	20	60·4	2·6	20	5·3%	-2·00 [-14·37, 10·37]
Kirilmaz 2015	66·1	16·4	41	61·8	19·7	42	12·3%	4·30 [-3·49, 12·09]
Liu 2016	42·1	4·9	26	42·4	7·4	26	40·2%	-0·30 [-3·71, 3·11]
Morano 2007	63·9	27·3	8	47·5	16·4	8	1·7%	16·40 [-5·67, 38·47]
**Subtotal (95% CI)**			**120**			**125**	**76·9%**	**2·71 [-1·42, 6·85]**
Heterogeneity: Tau² = 7·34; Chi² = 6·11, df = 4 (P = 0·19); I² = 35%								
Test for overall effect: Z = 1·29 (P = 0·20)								
**12·4·2 Avanafil**								
Derosa 2022	57·4	10·9	30	56·3	9·8	30	23·1%	1·10 [-4·15, 6·35]
**Subtotal (95% CI)**			**30**			**30**	**23·1%**	**1·10 [-4·15, 6·35]**
Heterogeneity: Not applicable								
Test for overall effect: Z = 0·41 (P = 0·68)								
**Total (95% CI)**			**150**			**155**	**100·0%**	**1·99 [-0·95, 4·93]**
Heterogeneity: Tau² = 2·57; Chi² = 6·15, df = 5 (P = 0·29); I² = 19%								
Test for overall effect: Z = 1·32 (P = 0·19)								
Test for subgroup differences: Chi² = 0·22, df = 1 (P = 0·64), I² = 0%								

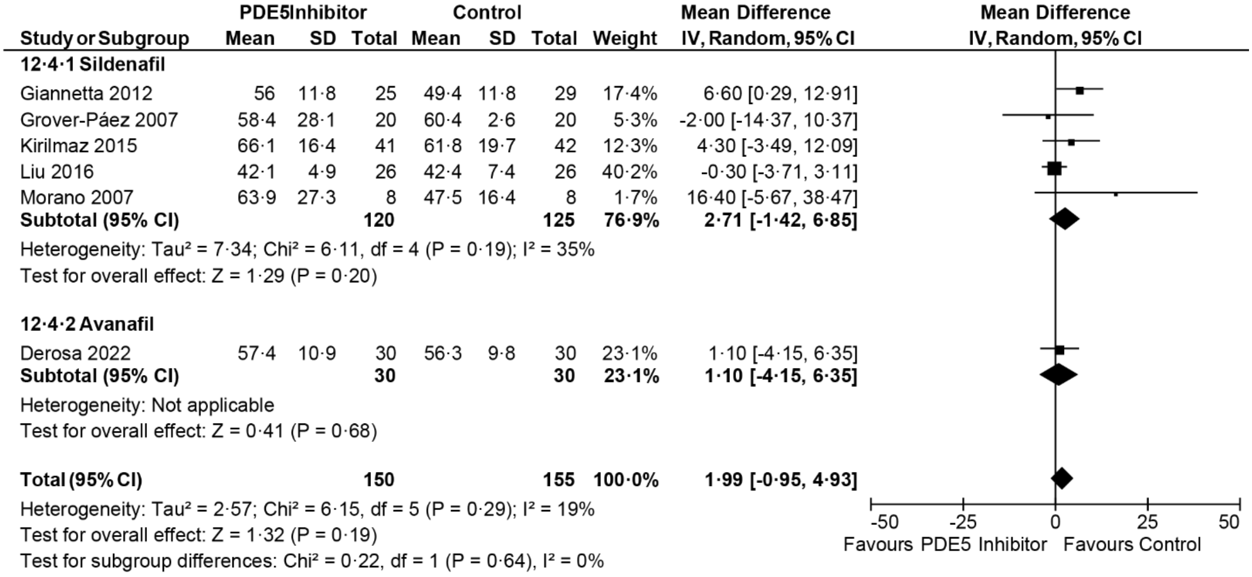

Supplementary Fig. 18: Sensitivity analysis of meta-analysis containing only trials with consistent, time-based intervention administration

Sensitivity analysis was performed on a potential decision to only include trials that enforced a consistent, time-based intervention administration, as well as on a potential decision to represent data in IFCC units (mmol/mol) or NGSP units (%). The following conditions were used for each meta-analysis: a) long half-life PDE5 inhibitors using NGSP units; b) short half-life PDE5 inhibitors using NGSP units; c) long half-life PDE5 inhibitors using IFCC units; d) short half-life PDE5 inhibitors using IFCC units.

a)

	PDE5Inhibitor			Control				Mean Difference
Study or Subgroup	Mean	SD	Total	Mean	SD	Total	Weight	IV, Random, 95% CI
**13·1·1 Trials With Time-Based Treatment**								
Fryk 2023	6·21	0·51	18	6·44	0·66	18	13·9%	-0·23 [-0·62, 0·16]
Hegazy 2024	8·78	1·9	30	11·59	2·32	30	4·5%	-2·81 [-3·88, -1·74]
Khazaal 2014	7·41	0·33	18	7·69	0·35	17	17·3%	-0·28 [-0·51, -0·05]
Lee 2022	6·64	0·46	23	7·29	0·31	45	17·6%	-0·65 [-0·86, -0·44]
Pofi 2022	6·64	0·37	55	6·86	0·37	43	18·7%	-0·22 [-0·37, -0·07]
Scheele 2016	7·11	1·14	164	7·25	1·02	62	15·6%	-0·14 [-0·45, 0·17]
**Subtotal (95% CI)**			**308**			**215**	**87·6%**	**-0·45 [-0·73, -0·16]**
Heterogeneity: Tau² = 0·10; Chi² = 33·57, df = 5 (P < 0·00001); I² = 85%								
Test for overall effect: Z = 3·04 (P = 0·002)								
**13·1·2 Other Trials**								
Sáenz de Tejada 2002	8·07	1·34	136	8·21	1·48	55	12·4%	-0·14 [-0·59, 0·31]
**Subtotal (95% CI)**			**136**			**55**	**12·4%**	**-0·14 [-0·59, 0·31]**
Heterogeneity: Not applicable								
Test for overall effect: Z = 0·61 (P = 0·54)								
**Total (95% CI)**			**444**			**270**	**100·0%**	**-0·40 [-0·66, -0·14]**
Heterogeneity: Tau² = 0·09; Chi² = 34·27, df = 6 (P < 0·00001); I² = 82%								
Test for overall effect: Z = 3·03 (P = 0·002)								
Test for subgroup differences: Chi² = 1·26, df = 1 (P = 0·26), I² = 20·5%								

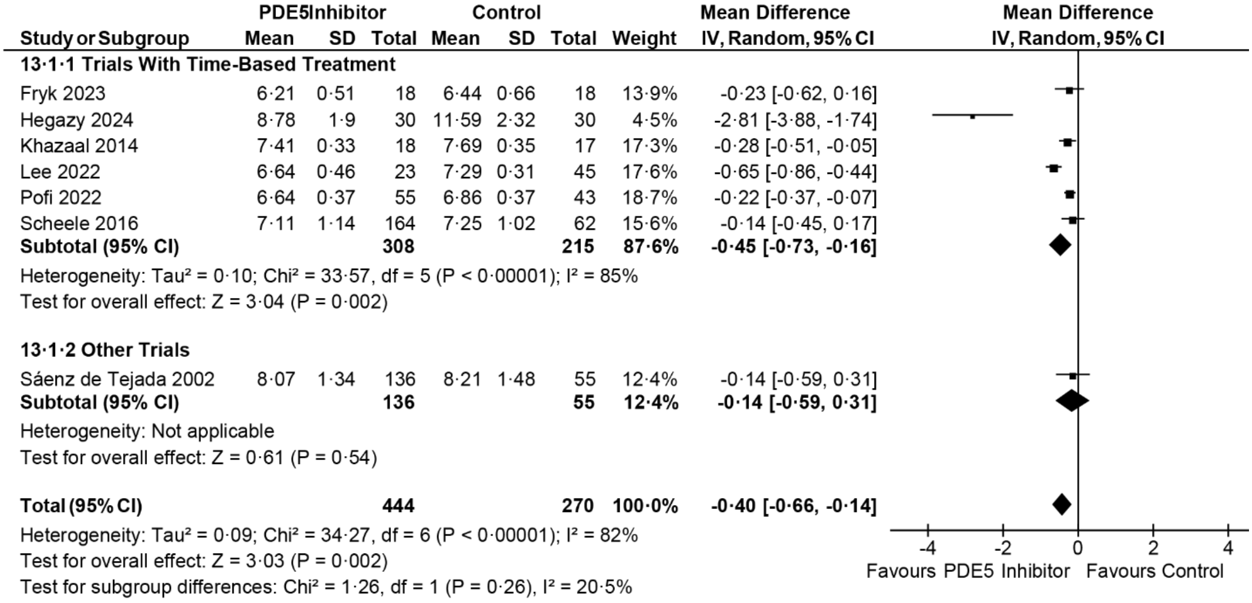

b)

	PDE5Inhibitor			Control				Mean Difference
Study or Subgroup	Mean	SD	Total	Mean	SD	Total	Weight	IV, Random, 95% CI
**13·2·1 Trials With Time-Based Treatment**								
Giannetta 2012	7·27	1·08	25	6·67	1·08	29	17·4%	0·60 [0·02, 1·18]
Grover-Páez 2007	7·49	2·57	20	7·67	0·24	20	5·3%	-0·18 [-1·31, 0·95]
Kirilmaz 2015	8·2	1·5	41	7·8	1·8	42	12·3%	0·40 [-0·31, 1·11]
Liu 2016	6	0·45	26	6·03	0·68	26	40·1%	-0·03 [-0·34, 0·28]
Morano 2007	8	2·5	8	6·5	1·5	8	1·7%	1·50 [-0·52, 3·52]
**Subtotal (95% CI)**			**120**			**125**	**76·9%**	**0·25 [-0·13, 0·63]**
Heterogeneity: Tau² = 0·06; Chi² = 6·11, df = 4 (P = 0·19); I² = 35%								
Test for overall effect: Z = 1·28 (P = 0·20)								
**13·2·2 Other Trials**								
Derosa 2022	7·4	1	30	7·3	0·9	30	23·1%	0·10 [-0·38, 0·58]
**Subtotal (95% CI)**			**30**			**30**	**23·1%**	**0·10 [-0·38, 0·58]**
Heterogeneity: Not applicable								
Test for overall effect: Z = 0·41 (P = 0·68)								
**Total (95% CI)**			**150**			**155**	**100·0%**	**0·18 [-0·09, 0·45]**
Heterogeneity: Tau² = 0·02; Chi² = 6·15, df = 5 (P = 0·29); I² = 19%								
Test for overall effect: Z = 1·32 (P = 0·19)								
Test for subgroup differences: Chi² = 0·22, df = 1 (P = 0·64), I² = 0%								

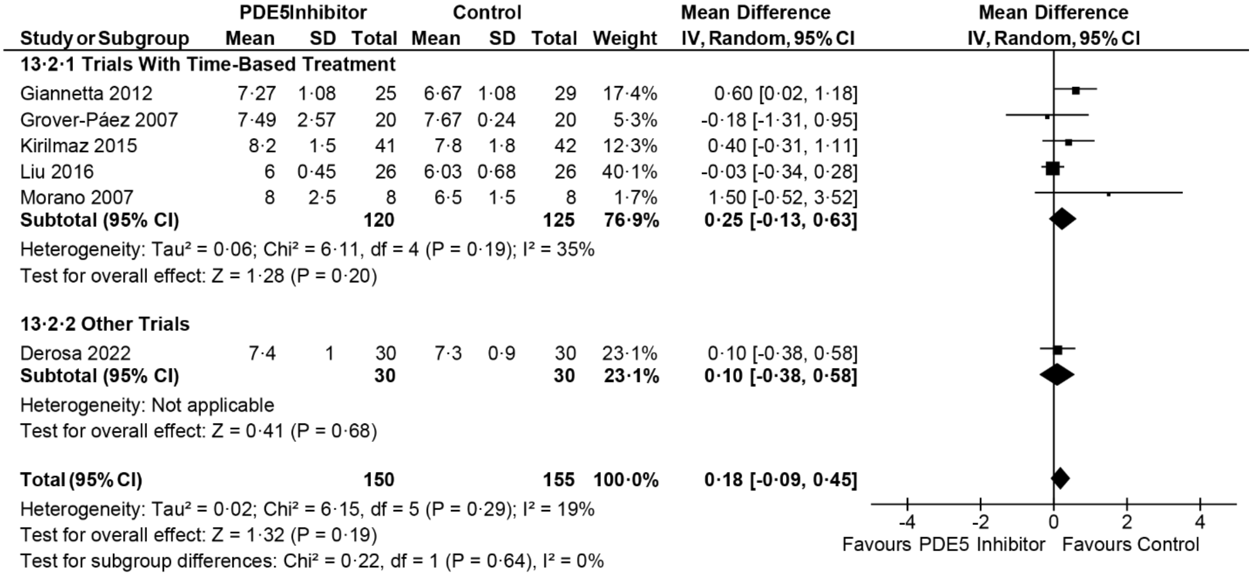

c)

Study or Subgroup	PDE5Inhibitor Mean	SD	Total	Control Mean	SD	Total	Weight	Mean Difference IV, Random, 95% CI
**13·3·1 Trials With Time-Based Treatment**								
Fryk 2023	44·4	5·5	18	46·9	7·2	18	13·9%	-2·50 [-6·69, 1·69]
Hegazy 2024	72·5	20·8	30	103·2	25·4	30	4·6%	-30·70 [-42·45, -18·95]
Khazaal 2014	57·5	3·6	18	60·5	3·9	17	17·2%	-3·00 [-5·49, -0·51]
Lee 2022	49·1	5	23	56·2	3·4	45	17·6%	-7·10 [-9·37, -4·83]
Pofi 2022	49·1	4	55	51·4	4	43	18·7%	-2·30 [-3·90, -0·70]
Scheele 2016	54·2	12·5	164	55·7	11·1	62	15·6%	-1·50 [-4·86, 1·86]
**Subtotal (95% CI)**			**308**			**215**	**87·5%**	**-4·84 [-8·01, -1·68]**
Heterogeneity: Tau² = 11·72; Chi² = 34·09, df = 5 (P < 0·00001); I² = 85%								
Test for overall effect: Z = 3·00 (P = 0·003)								
**13·3·2 Other Trials**								
Sáenz de Tejada 2002	64·7	14·7	136	66·3	16·1	55	12·5%	-1·60 [-6·52, 3·32]
**Subtotal (95% CI)**			**136**			**55**	**12·5%**	**-1·60 [-6·52, 3·32]**
Heterogeneity: Not applicable								
Test for overall effect: Z = 0·64 (P = 0·52)								
**Total (95% CI)**			**444**			**270**	**100·0%**	**-4·38 [-7·23, -1·52]**
Heterogeneity: Tau² = 10·72; Chi² = 34·71, df = 6 (P < 0·00001); I² = 83%								
Test for overall effect: Z = 3·00 (P = 0·003)								
Test for subgroup differences: Chi² = 1·18, df = 1 (P = 0·28), I² = 15·2%								

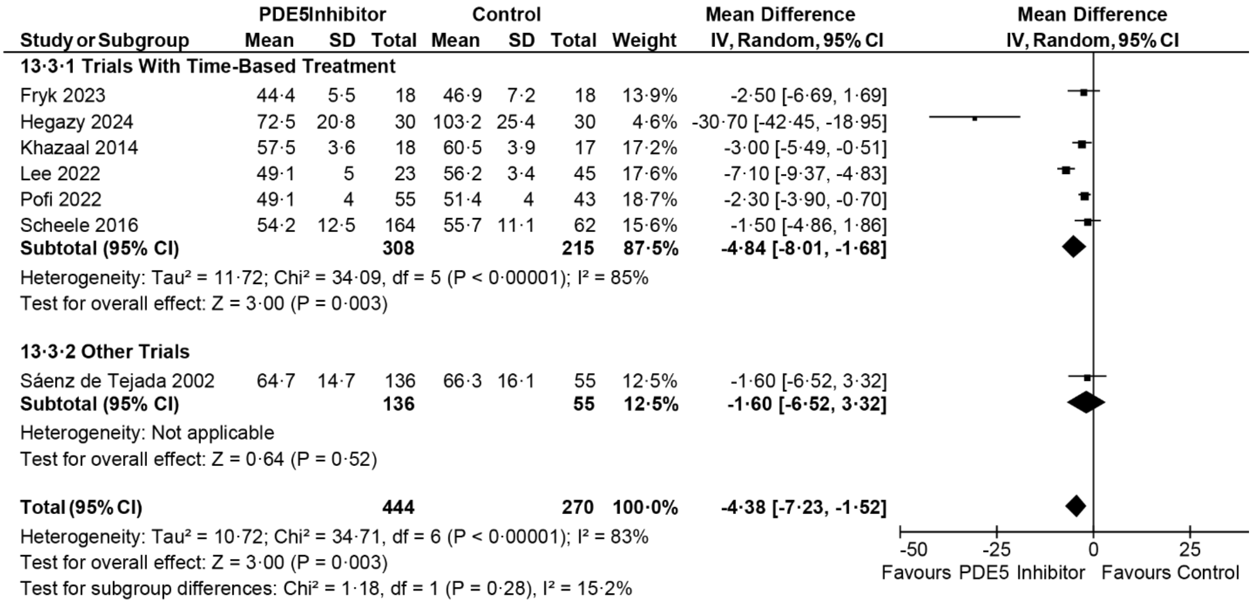

d)

Study or Subgroup	PDE5Inhibitor Mean	SD	Total	Control Mean	SD	Total	Weight	Mean Difference IV, Random, 95% CI
**13·4·1 Trials With Time-Based Treatment**								
Giannetta 2012	56	11·8	25	49·4	11·8	29	17·4%	6·60 [0·29, 12·91]
Grover-Páez 2007	58·4	28·1	20	60·4	2·6	20	5·3%	-2·00 [-14·37, 10·37]
Kirilmaz 2015	66·1	16·4	41	61·8	19·7	42	12·3%	4·30 [-3·49, 12·09]
Liu 2016	42·1	4·9	26	42·4	7·4	26	40·2%	-0·30 [-3·71, 3·11]
Morano 2007	63·9	27·3	8	47·5	16·4	8	1·7%	16·40 [-5·67, 38·47]
**Subtotal (95% CI)**			**120**			**125**	**76·9%**	**2·71 [-1·42, 6·85]**
Heterogeneity: Tau² = 7·34; Chi² = 6·11, df = 4 (P = 0·19); I² = 35%								
Test for overall effect: Z = 1·29 (P = 0·20)								
**13·4·2 Other Trials**								
Derosa 2022	57·4	10·9	30	56·3	9·8	30	23·1%	1·10 [-4·15, 6·35]
**Subtotal (95% CI)**			**30**			**30**	**23·1%**	**1·10 [-4·15, 6·35]**
Heterogeneity: Not applicable								
Test for overall effect: Z = 0·41 (P = 0·68)								
**Total (95% CI)**			**150**			**155**	**100·0%**	**1·99 [-0·95, 4·93]**
Heterogeneity: Tau² = 2·57; Chi² = 6·15, df = 5 (P = 0·29); I² = 19%								
Test for overall effect: Z = 1·32 (P = 0·19)								
Test for subgroup differences: Chi² = 0·22, df = 1 (P = 0·64), I² = 0%								

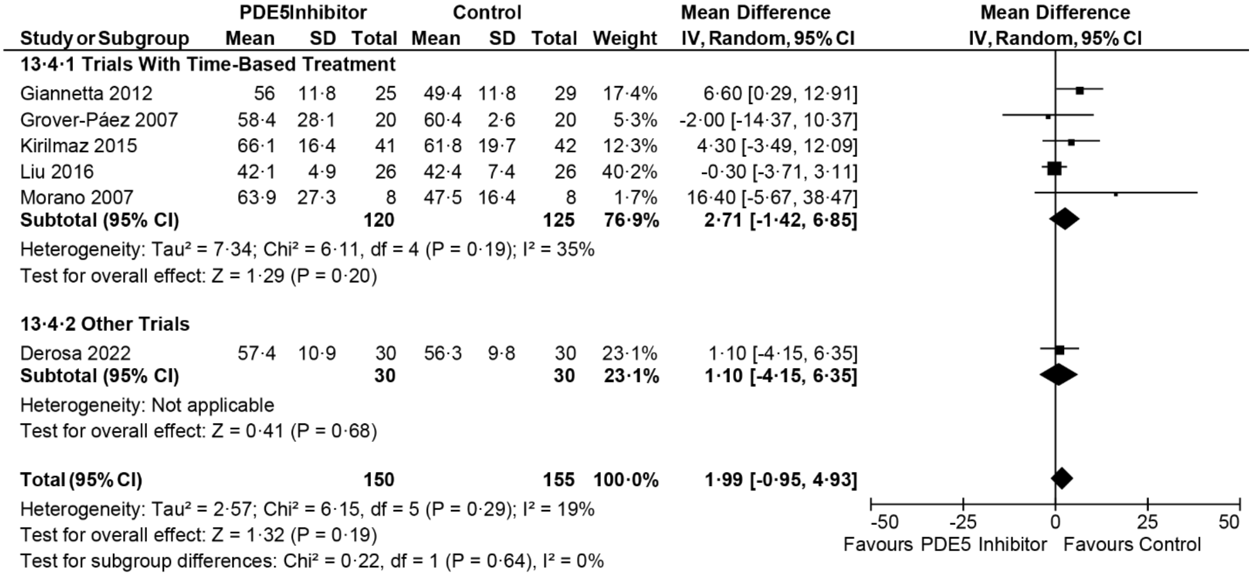

Supplementary Fig. 19: Sensitivity analysis of meta-analysis grouped by risk of bias

Sensitivity analysis was performed on a potential decision to include all trials grouped by risk of bias, as well as on a potential decision to represent data in IFCC units (mmol/mol) or NGSP units (%). The following conditions were used for each meta-analysis: a) long half-life PDE5 inhibitors using NGSP units; b) short half-life PDE5 inhibitors using NGSP units; c) long half-life PDE5 inhibitors using IFCC units; d) short half-life PDE5 inhibitors using IFCC units.

a)

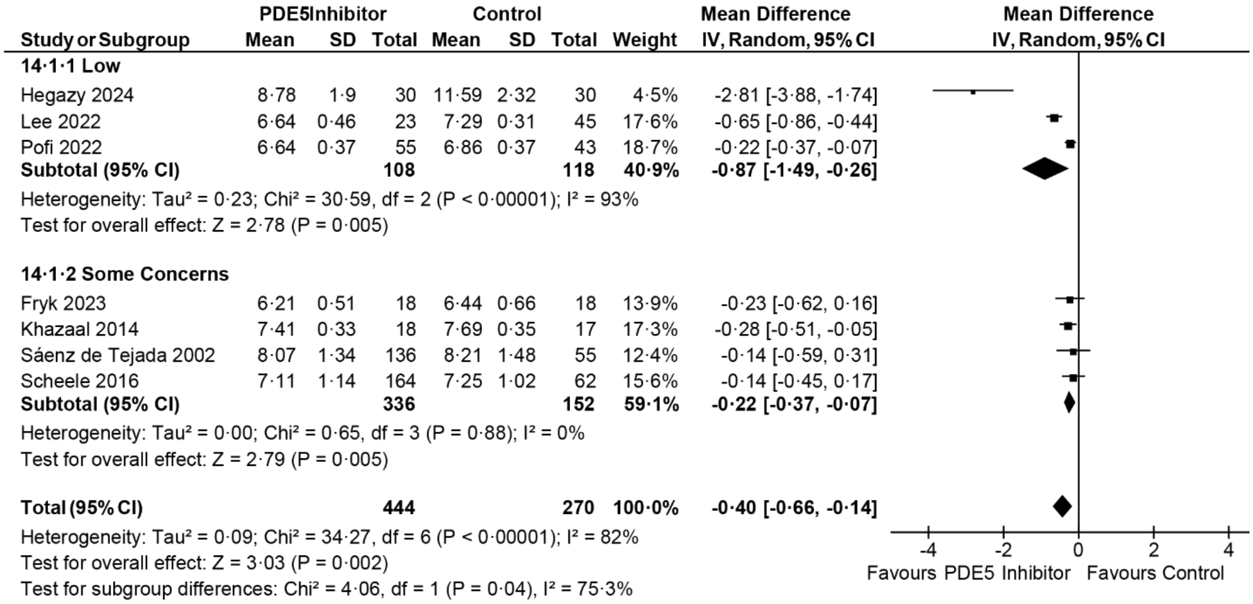

b)

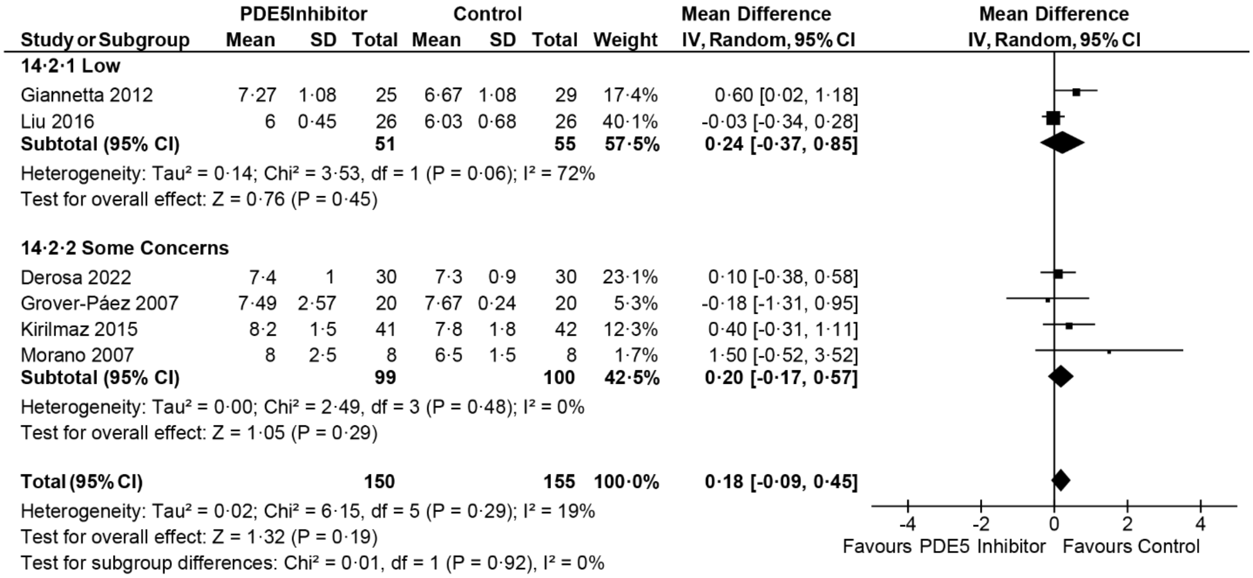

c)

Study or Subgroup	PDE5Inhibitor Mean	PDE5Inhibitor SD	PDE5Inhibitor Total	Control Mean	Control SD	Control Total	Weight	Mean Difference IV, Random, 95% CI
**14·3·1 Low**								
Hegazy 2024	72·5	20·8	30	103·2	25·4	30	4·6%	-30·70 [-42·45, -18·95]
Lee 2022	49·1	5	23	56·2	3·4	45	17·6%	-7·10 [-9·37, -4·83]
Pofi 2022	49·1	4	55	51·4	4	43	18·7%	-2·30 [-3·90, -0·70]
**Subtotal (95% CI)**			**108**			**118**	**40·8%**	**-9·49 [-16·25, -2·74]**
Heterogeneity: Tau² = 28·14; Chi² = 31·25, df = 2 (P < 0·00001); I² = 94%								
Test for overall effect: Z = 2·76 (P = 0·006)								
**14·3·2 Some Concerns**								
Fryk 2023	44·4	5·5	18	46·9	7·2	18	13·9%	-2·50 [-6·69, 1·69]
Khazaal 2014	57·5	3·6	18	60·5	3·9	17	17·2%	-3·00 [-5·49, -0·51]
Sáenz de Tejada 2002	64·7	14·7	136	66·3	16·1	55	12·5%	-1·60 [-6·52, 3·32]
Scheele 2016	54·2	12·5	164	55·7	11·1	62	15·6%	-1·50 [-4·86, 1·86]
**Subtotal (95% CI)**			**336**			**152**	**59·2%**	**-2·37 [-4·07, -0·68]**
Heterogeneity: Tau² = 0·00; Chi² = 0·60, df = 3 (P = 0·90); I² = 0%								
Test for overall effect: Z = 2·74 (P = 0·006)								
**Total (95% CI)**			**444**			**270**	**100·0%**	**-4·38 [-7·23, -1·52]**
Heterogeneity: Tau² = 10·72; Chi² = 34·71, df = 6 (P < 0·00001); I² = 83%								
Test for overall effect: Z = 3·00 (P = 0·003)								
Test for subgroup differences: Chi² = 4·02, df = 1 (P = 0·04), I² = 75·1%								

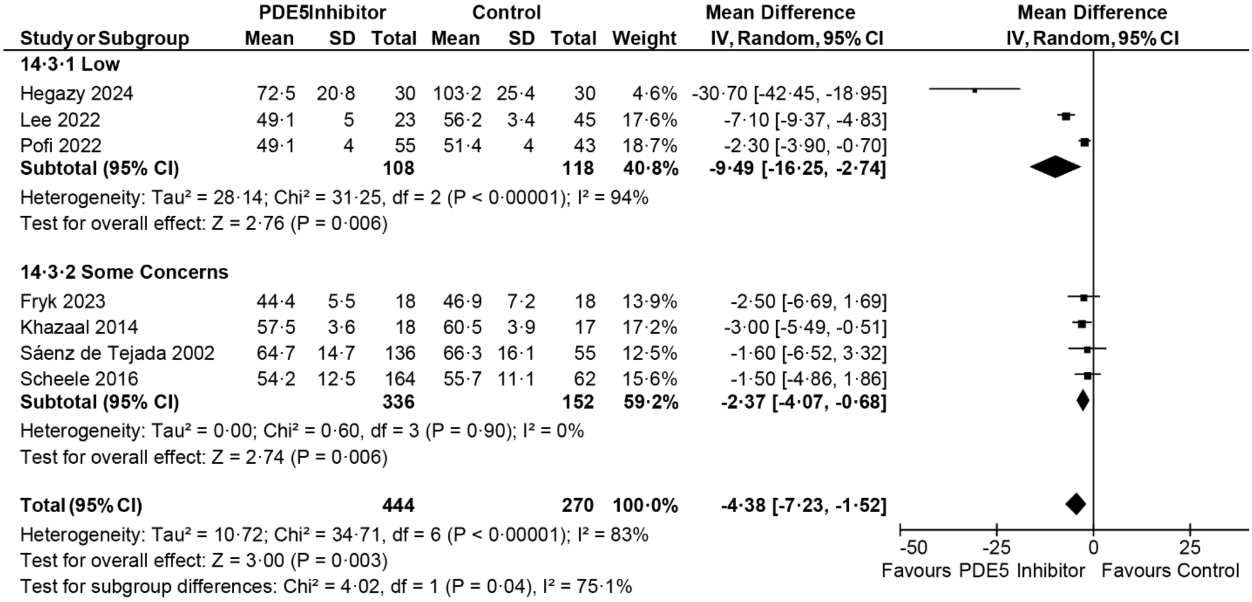

d)

Study or Subgroup	PDE5Inhibitor Mean	PDE5Inhibitor SD	PDE5Inhibitor Total	Control Mean	Control SD	Control Total	Weight	Mean Difference IV, Random, 95% CI
**14·4·1 Low**								
Giannetta 2012	56	11·8	25	49·4	11·8	29	17·4%	6·60 [0·29, 12·91]
Liu 2016	42·1	4·9	26	42·4	7·4	26	40·2%	-0·30 [-3·71, 3·11]
**Subtotal (95% CI)**			**51**			**55**	**57·6%**	**2·62 [-4·06, 9·30]**
Heterogeneity: Tau² = 17·10; Chi² = 3·55, df = 1 (P = 0·06); I² = 72%								
Test for overall effect: Z = 0·77 (P = 0·44)								
**14·4·2 Some Concerns**								
Derosa 2022	57·4	10·9	30	56·3	9·8	30	23·1%	1·10 [-4·15, 6·35]
Grover-Páez 2007	58·4	28·1	20	60·4	2·6	20	5·3%	-2·00 [-14·37, 10·37]
Kirilmaz 2015	66·1	16·4	41	61·8	19·7	42	12·3%	4·30 [-3·49, 12·09]
Morano 2007	63·9	27·3	8	47·5	16·4	8	1·7%	16·40 [-5·67, 38·47]
**Subtotal (95% CI)**			**99**			**100**	**42·4%**	**2·14 [-1·90, 6·18]**
Heterogeneity: Tau² = 0·00; Chi² = 2·48, df = 3 (P = 0·48); I² = 0%								
Test for overall effect: Z = 1·04 (P = 0·30)								
**Total (95% CI)**			**150**			**155**	**100·0%**	**1·99 [-0·95, 4·93]**
Heterogeneity: Tau² = 2·57; Chi² = 6·15, df = 5 (P = 0·29); I² = 19%								
Test for overall effect: Z = 1·32 (P = 0·19)								
Test for subgroup differences: Chi² = 0·01, df = 1 (P = 0·90), I² = 0%								

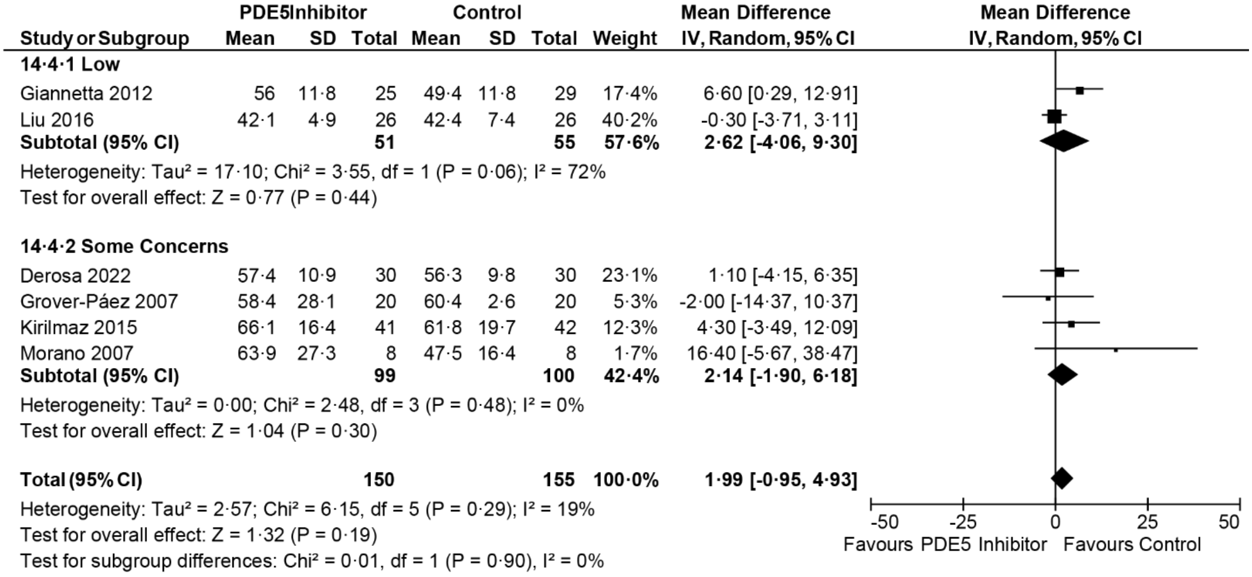

Supplementary Fig. 20: Sensitivity analysis of meta-analysis performed using a fixed-effects model

Sensitivity analysis was performed on a potential decision to include all trials using a fixed-effects model, as well as on a potential decision to represent data in IFCC units (mmol/mol) or NGSP units (%). The following conditions were used for each meta-analysis: a) long half-life PDE5 inhibitors using NGSP units; b) short half-life PDE5 inhibitors using NGSP units; c) long half-life PDE5 inhibitors using IFCC units; d) short half-life PDE5 inhibitors using IFCC units.

a)

	PDE5Inhibitor			Control				Mean Difference
**Study or Subgroup**	**Mean**	**SD**	**Total**	**Mean**	**SD**	**Total**	**Weight**	**IV, Fixed, 95% CI**
Fryk 2023	6·21	0·51	18	6·44	0·66	18	6·0%	-0·23 [-0·62, 0·16]
Hegazy 2024	8·78	1·9	30	11·59	2·32	30	0·8%	-2·81 [-3·88, -1·74]
Khazaal 2014	7·41	0·33	18	7·69	0·35	17	17·6%	-0·28 [-0·51, -0·05]
Lee 2022	6·64	0·46	23	7·29	0·31	45	20·6%	-0·65 [-0·86, -0·44]
Pofi 2022	6·64	0·37	55	6·86	0·37	43	41·1%	-0·22 [-0·37, -0·07]
Sáenz de Tejada 2002	8·07	1·34	136	8·21	1·48	55	4·4%	-0·14 [-0·59, 0·31]
Scheele 2016	7·11	1·14	164	7·25	1·02	62	9·4%	-0·14 [-0·45, 0·17]
**Total (95% CI)**			**444**			**270**	**100·0%**	**-0·33 [-0·42, -0·23]**

Heterogeneity: Chi² = 34·27, df = 6 (P < 0·00001); I² = 82%
Test for overall effect: Z = 6·81 (P < 0·00001)

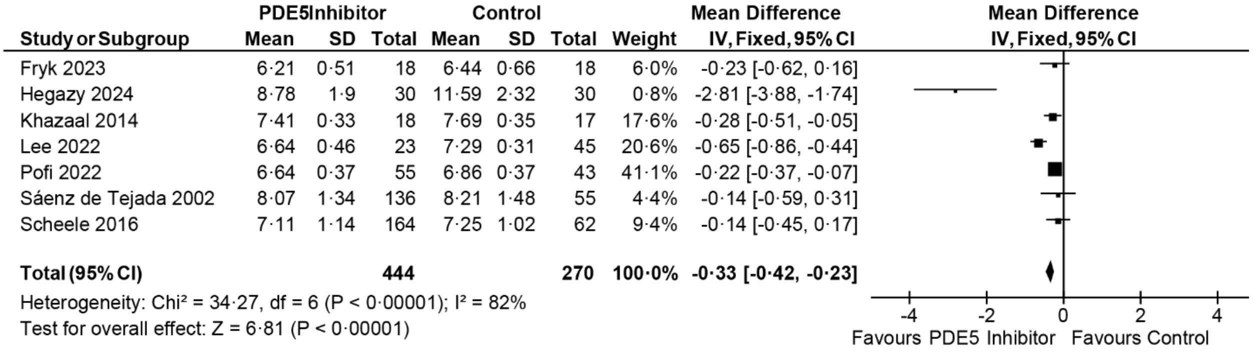

b)

	PDE5Inhibitor			Control				Mean Difference
**Study or Subgroup**	**Mean**	**SD**	**Total**	**Mean**	**SD**	**Total**	**Weight**	**IV, Fixed, 95% CI**
Derosa 2022	7·4	1	30	7·3	0·9	30	21·1%	0·10 [-0·38, 0·58]
Giannetta 2012	7·27	1·08	25	6·67	1·08	29	14·6%	0·60 [0·02, 1·18]
Grover-Páez 2007	7·49	2·57	20	7·67	0·24	20	3·8%	-0·18 [-1·31, 0·95]
Kirilmaz 2015	8·2	1·5	41	7·8	1·8	42	9·6%	0·40 [-0·31, 1·11]
Liu 2016	6	0·45	26	6·03	0·68	26	49·7%	-0·03 [-0·34, 0·28]
Morano 2007	8	2·5	8	6·5	1·5	8	1·2%	1·50 [-0·52, 3·52]
**Total (95% CI)**			**150**			**155**	**100·0%**	**0·14 [-0·08, 0·36]**

Heterogeneity: Chi² = 6·15, df = 5 (P = 0·29); I² = 19%
Test for overall effect: Z = 1·27 (P = 0·20)

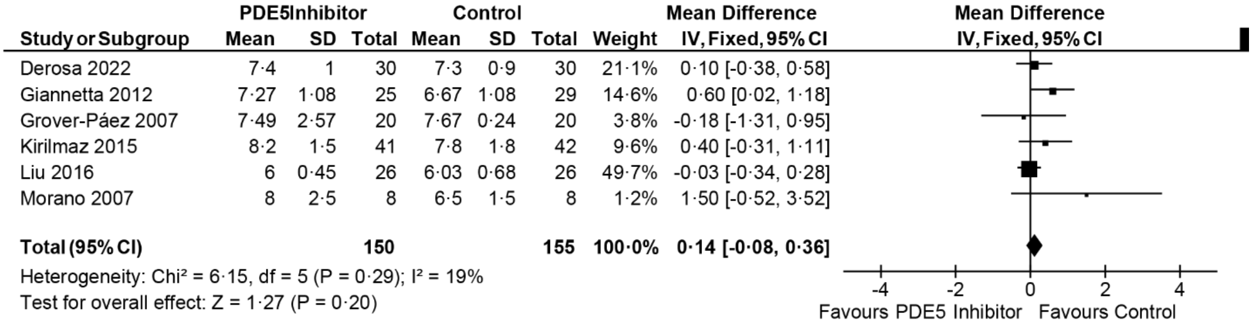

c)

	PDE5Inhibitor			Control				Mean Difference
**Study or Subgroup**	**Mean**	**SD**	**Total**	**Mean**	**SD**	**Total**	**Weight**	**IV, Fixed, 95% CI**
Fryk 2023	44·4	5·5	18	46·9	7·2	18	6·1%	-2·50 [-6·69, 1·69]
Hegazy 2024	72·5	20·8	30	103·2	25·4	30	0·8%	-30·70 [-42·45, -18·95]
Khazaal 2014	57·5	3·6	18	60·5	3·9	17	17·1%	-3·00 [-5·49, -0·51]
Lee 2022	49·1	5	23	56·2	3·4	45	20·6%	-7·10 [-9·37, -4·83]
Pofi 2022	49·1	4	55	51·4	4	43	41·7%	-2·30 [-3·90, -0·70]
Sáenz de Tejada 2002	64·7	14·7	136	66·3	16·1	55	4·4%	-1·60 [-6·52, 3·32]
Scheele 2016	54·2	12·5	164	55·7	11·1	62	9·4%	-1·50 [-4·86, 1·86]
**Total (95% CI)**			**444**			**270**	**100·0%**	**-3·53 [-4·56, -2·50]**

Heterogeneity: Chi² = 34·71, df = 6 (P < 0·00001); I² = 83%
Test for overall effect: Z = 6·72 (P < 0·00001)

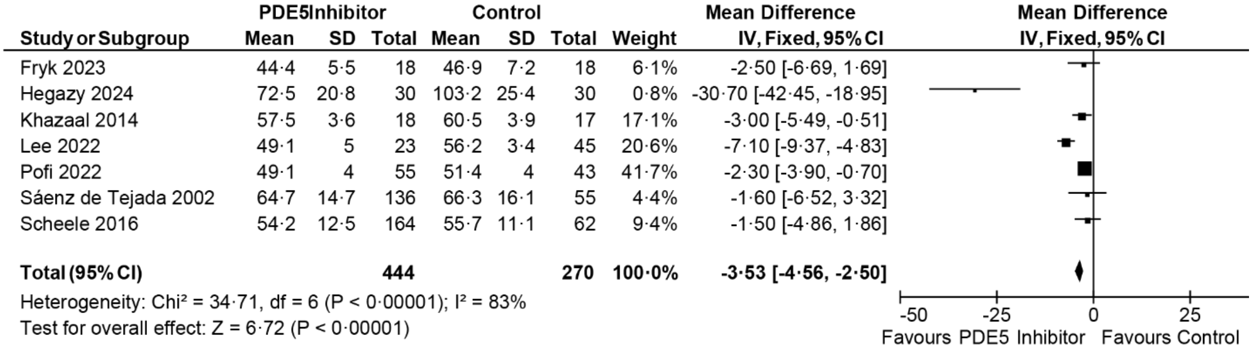

d)

	PDE5 Inhibitor			Control				Mean Difference
**Study or Subgroup**	**Mean**	**SD**	**Total**	**Mean**	**SD**	**Total**	**Weight**	**IV, Fixed, 95% CI**
Derosa 2022	57·4	10·9	30	56·3	9·8	30	21·1%	1·10 [-4·15, 6·35]
Giannetta 2012	56	11·8	25	49·4	11·8	29	14·6%	6·60 [0·29, 12·91]
Grover-Páez 2007	58·4	28·1	20	60·4	2·6	20	3·8%	-2·00 [-14·37, 10·37]
Kirilmaz 2015	66·1	16·4	41	61·8	19·7	42	9·6%	4·30 [-3·49, 12·09]
Liu 2016	42·1	4·9	26	42·4	7·4	26	49·8%	-0·30 [-3·71, 3·11]
Morano 2007	63·9	27·3	8	47·5	16·4	8	1·2%	16·40 [-5·67, 38·47]
**Total (95% CI)**			**150**			**155**	**100·0%**	**1·57 [-0·83, 3·98]**

Heterogeneity: Chi² = 6·15, df = 5 (P = 0·29); I² = 19%
Test for overall effect: Z = 1·28 (P = 0·20)

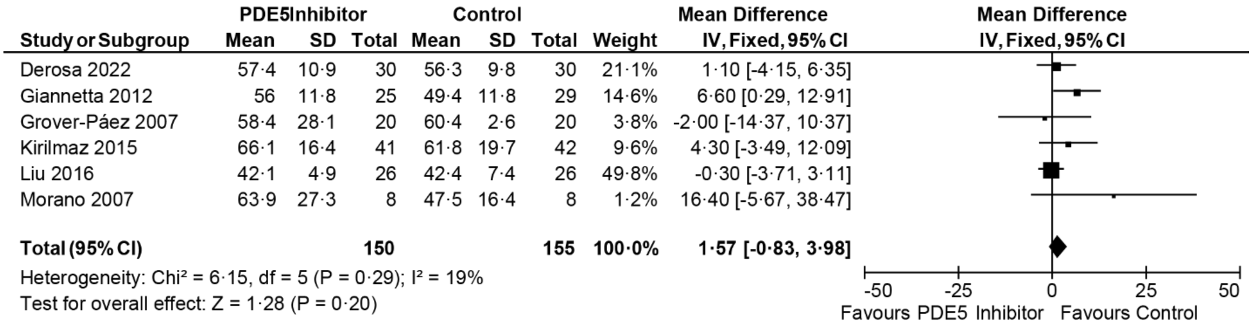

Supplementary Fig. 21: Sensitivity analysis of meta-analysis subgroup analysis performed using a fixed-effects model

Sensitivity analysis was performed on a potential decision to perform subgroup analysis using a fixed-effects model, as well as on a potential decision to represent data in IFCC units (mmol/mol) or NGSP units (%). The following conditions were used for each meta-analysis: a) long half-life PDE5 inhibitors using NGSP units; b) short half-life PDE5 inhibitors using NGSP units; c) long half-life PDE5 inhibitors using IFCC units; d) short half-life PDE5 inhibitors using IFCC units.

a)

	PDE5Inhibitor			Control				Mean Difference
**Study or Subgroup**	**Mean**	**SD**	**Total**	**Mean**	**SD**	**Total**	**Weight**	**IV, Fixed, 95% CI**
**16·1·1 Trials Meeting Subgroup Criteria**								
Hegazy 2024	8·78	1·9	30	11·59	2·32	30	0·8%	-2·81 [-3·88, -1·74]
Khazaal 2014	7·41	0·33	18	7·69	0·35	17	17·6%	-0·28 [-0·51, -0·05]
Lee 2022	6·64	0·46	23	7·29	0·31	45	20·6%	-0·65 [-0·86, -0·44]
Pofi 2022	6·64	0·37	55	6·86	0·37	43	41·1%	-0·22 [-0·37, -0·07]
Scheele 2016	7·11	1·14	164	7·25	1·02	62	9·4%	-0·14 [-0·45, 0·17]
**Subtotal (95% CI)**			**290**			**197**	**89·6%**	**-0·34 [-0·44, -0·24]**
Heterogeneity: Chi² = 33·25, df = 4 (P < 0·00001); I² = 88%								
Test for overall effect: Z = 6·75 (P < 0·00001)								
**16·1·2 Other Trials**								
Fryk 2023	6·21	0·51	18	6·44	0·66	18	6·0%	-0·23 [-0·62, 0·16]
Sáenz de Tejada 2002	8·07	1·34	136	8·21	1·48	55	4·4%	-0·14 [-0·59, 0·31]
**Subtotal (95% CI)**			**154**			**73**	**10·4%**	**-0·19 [-0·49, 0·10]**
Heterogeneity: Chi² = 0·09, df = 1 (P = 0·77); I² = 0%								
Test for overall effect: Z = 1·28 (P = 0·20)								
**Total (95% CI)**			**444**			**270**	**100·0%**	**-0·33 [-0·42, -0·23]**
Heterogeneity: Chi² = 34·27, df = 6 (P < 0·00001); I² = 82%								
Test for overall effect: Z = 6·81 (P < 0·00001)								
Test for subgroup differences: Chi² = 0·93, df = 1 (P = 0·33), I² = 0%								

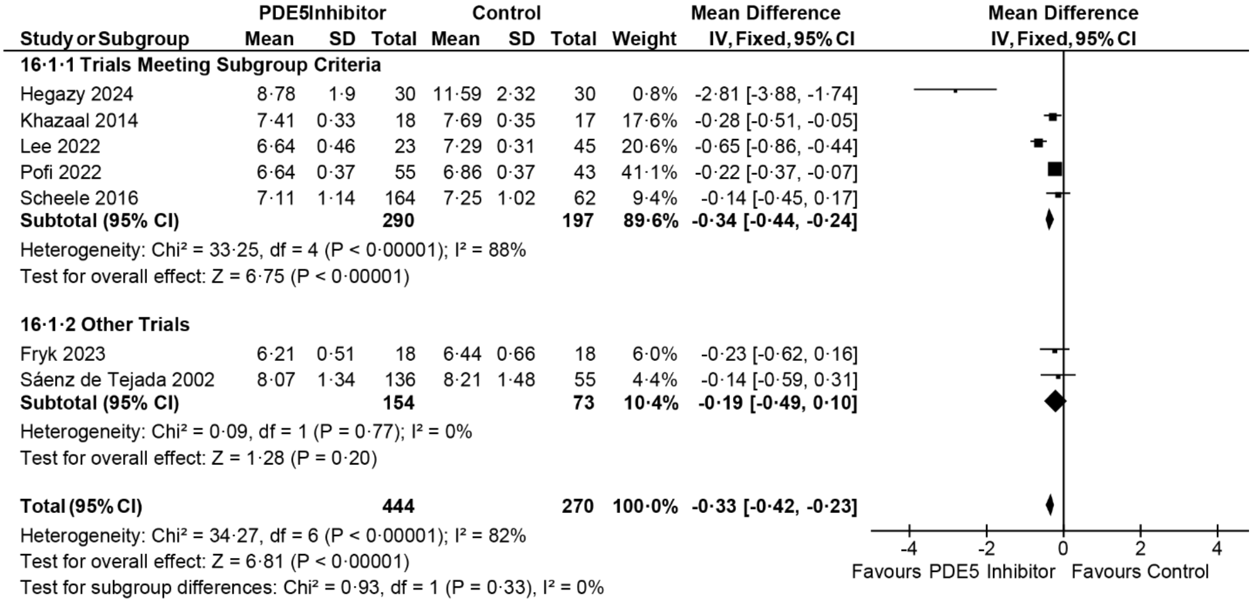

b)

	PDE5Inhibitor			Control				Mean Difference
**Study or Subgroup**	**Mean**	**SD**	**Total**	**Mean**	**SD**	**Total**	**Weight**	**IV, Fixed, 95% CI**
**16·2·1 Trials Meeting Subgroup Criteria**								
Derosa 2022	7·4	1	30	7·3	0·9	30	21·1%	0·10 [-0·38, 0·58]
Giannetta 2012	7·27	1·08	25	6·67	1·08	29	14·6%	0·60 [0·02, 1·18]
Kirilmaz 2015	8·2	1·5	41	7·8	1·8	42	9·6%	0·40 [-0·31, 1·11]
Morano 2007	8	2·5	8	6·5	1·5	8	1·2%	1·50 [-0·52, 3·52]
**Subtotal (95% CI)**			**104**			**109**	**46·5%**	**0·36 [0·03, 0·68]**
Heterogeneity: Chi² = 3·02, df = 3 (P = 0·39); I² = 1%								
Test for overall effect: Z = 2·15 (P = 0·03)								
**16·2·2 Other Trials**								
Grover-Páez 2007	7·49	2·57	20	7·67	0·24	20	3·8%	-0·18 [-1·31, 0·95]
Liu 2016	6	0·45	26	6·03	0·68	26	49·7%	-0·03 [-0·34, 0·28]
**Subtotal (95% CI)**			**46**			**46**	**53·5%**	**-0·04 [-0·34, 0·26]**
Heterogeneity: Chi² = 0·06, df = 1 (P = 0·80); I² = 0%								
Test for overall effect: Z = 0·26 (P = 0·79)								
**Total (95% CI)**			**150**			**155**	**100·0%**	**0·14 [-0·08, 0·36]**
Heterogeneity: Chi² = 6·15, df = 5 (P = 0·29); I² = 19%								
Test for overall effect: Z = 1·27 (P = 0·20)								
Test for subgroup differences: Chi² = 3·07, df = 1 (P = 0·08), I² = 67·4%								

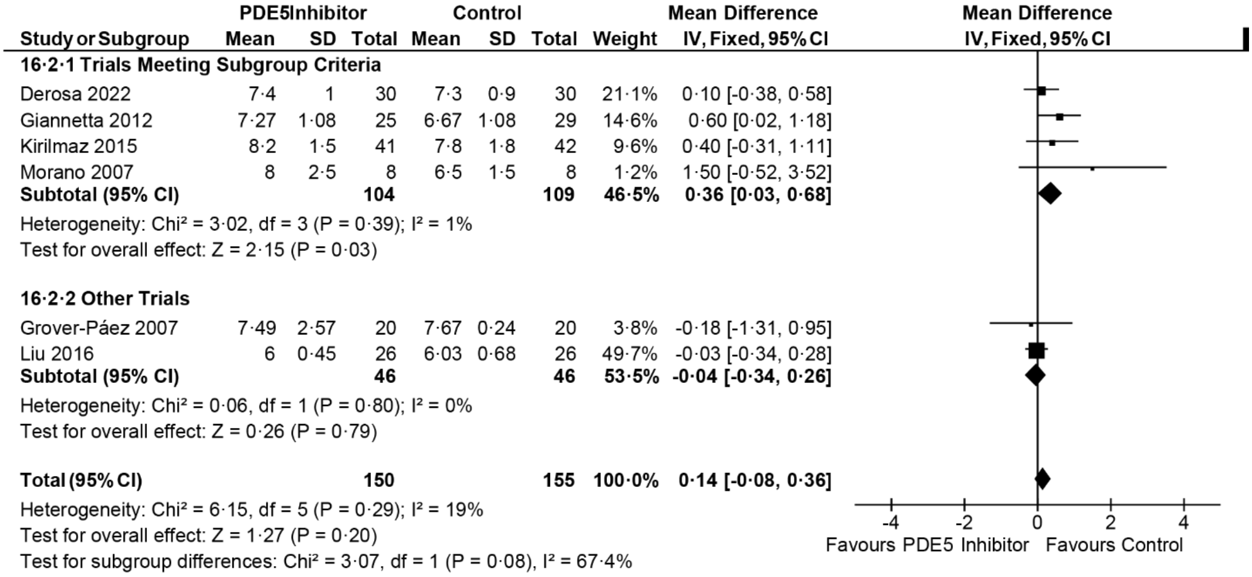

c)

Study or Subgroup	PDE5Inhibitor Mean	SD	Total	Control Mean	SD	Total	Weight	Mean Difference IV, Fixed, 95% CI
**16·3·1 Trials Meeting Subgroup Criteria**								
Hegazy 2024	72·5	20·8	30	103·2	25·4	30	0·8%	-30·70 [-42·45, -18·95]
Khazaal 2014	57·5	3·6	18	60·5	3·9	17	17·1%	-3·00 [-5·49, -0·51]
Lee 2022	49·1	5	23	56·2	3·4	45	20·6%	-7·10 [-9·37, -4·83]
Pofi 2022	49·1	4	55	51·4	4	43	41·7%	-2·30 [-3·90, -0·70]
Scheele 2016	54·2	12·5	164	55·7	11·1	62	9·4%	-1·50 [-4·86, 1·86]
**Subtotal (95% CI)**			**290**			**197**	**89·6%**	**-3·70 [-4·79, -2·61]**
Heterogeneity: Chi² = 33·80, df = 4 (P < 0·00001); I² = 88%								
Test for overall effect: Z = 6·65 (P < 0·00001)								
**16·3·2 Other Trials**								
Fryk 2023	44·4	5·5	18	46·9	7·2	18	6·1%	-2·50 [-6·69, 1·69]
Sáenz de Tejada 2002	64·7	14·7	136	66·3	16·1	55	4·4%	-1·60 [-6·52, 3·32]
**Subtotal (95% CI)**			**154**			**73**	**10·4%**	**-2·12 [-5·31, 1·07]**
Heterogeneity: Chi² = 0·07, df = 1 (P = 0·78); I² = 0%								
Test for overall effect: Z = 1·30 (P = 0·19)								
**Total (95% CI)**			**444**			**270**	**100·0%**	**-3·53 [-4·56, -2·50]**
Heterogeneity: Chi² = 34·71, df = 6 (P < 0·00001); I² = 83%								
Test for overall effect: Z = 6·72 (P < 0·00001)								
Test for subgroup differences: Chi² = 0·84, df = 1 (P = 0·36), I² = 0%								

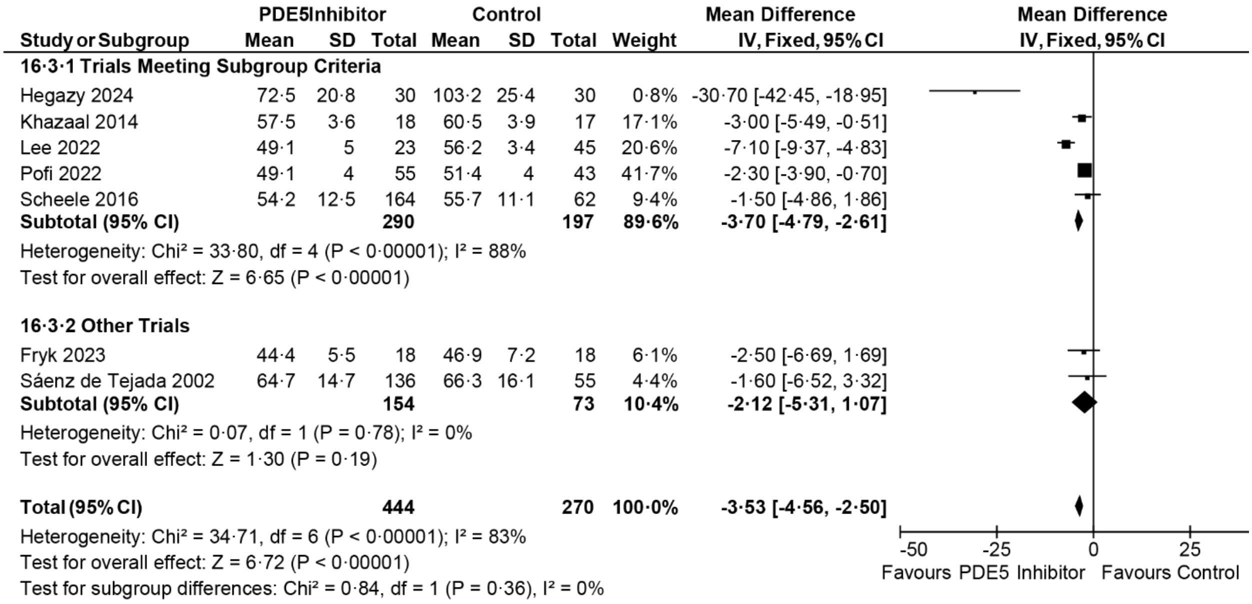

d)

Study or Subgroup	PDE5Inhibitor Mean	SD	Total	Control Mean	SD	Total	Weight	Mean Difference IV, Fixed, 95% CI
**16·4·1 Trials Meeting Subgroup Criteria**								
Derosa 2022	57·4	10·9	30	56·3	9·8	30	21·1%	1·10 [-4·15, 6·35]
Giannetta 2012	56	11·8	25	49·4	11·8	29	14·6%	6·60 [0·29, 12·91]
Kirilmaz 2015	66·1	16·4	41	61·8	19·7	42	9·6%	4·30 [-3·49, 12·09]
Morano 2007	63·9	27·3	8	47·5	16·4	8	1·2%	16·40 [-5·67, 38·47]
**Subtotal (95% CI)**			**104**			**109**	**46·4%**	**3·88 [0·34, 7·41]**
Heterogeneity: Chi² = 3·04, df = 3 (P = 0·39); I² = 1%								
Test for overall effect: Z = 2·15 (P = 0·03)								
**16·4·2 Other Trials**								
Grover-Páez 2007	58·4	28·1	20	60·4	2·6	20	3·8%	-2·00 [-14·37, 10·37]
Liu 2016	42·1	4·9	26	42·4	7·4	26	49·8%	-0·30 [-3·71, 3·11]
**Subtotal (95% CI)**			**46**			**46**	**53·6%**	**-0·42 [-3·71, 2·87]**
Heterogeneity: Chi² = 0·07, df = 1 (P = 0·80); I² = 0%								
Test for overall effect: Z = 0·25 (P = 0·80)								
**Total (95% CI)**			**150**			**155**	**100·0%**	**1·57 [-0·83, 3·98]**
Heterogeneity: Chi² = 6·15, df = 5 (P = 0·29); I² = 19%								
Test for overall effect: Z = 1·28 (P = 0·20)								
Test for subgroup differences: Chi² = 3·04, df = 1 (P = 0·08), I² = 67·1%								

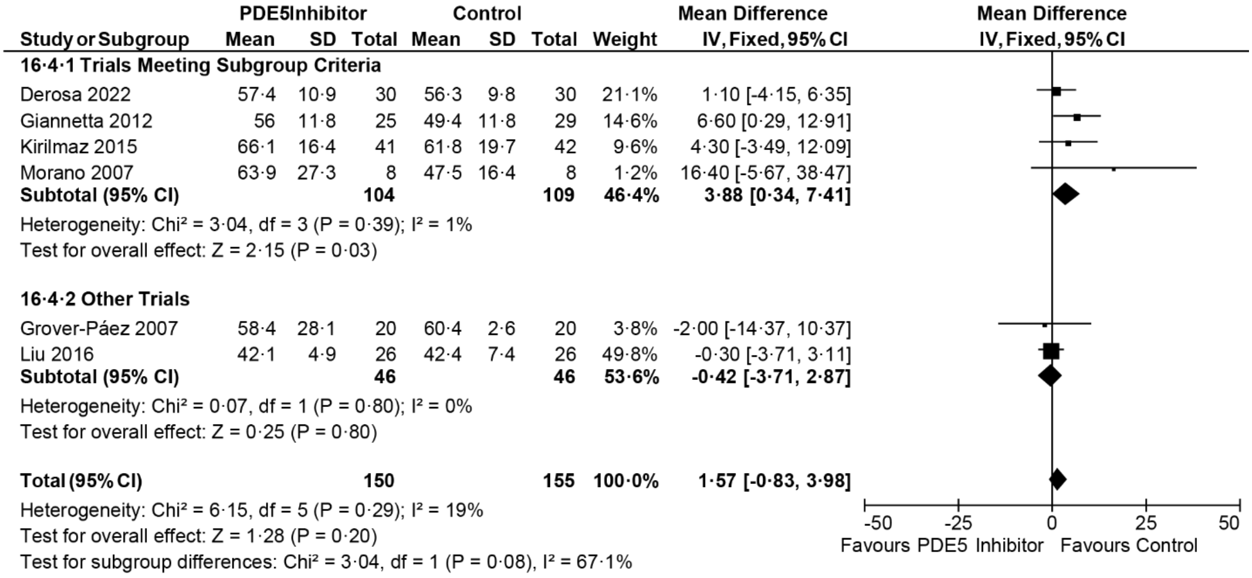

Supplementary Fig. 22: Sensitivity analysis of meta-analysis containing only trials not requiring data estimation

Sensitivity analysis was performed on a potential decision to only include trials which reported data in methods that did not require data estimation to convert to means and standard deviations (such as by using change-from-baseline statistics or by using median and quartile measures), as well as on a potential decision to represent data in IFCC units (mmol/mol) or NGSP units (%). The following conditions were used for each meta-analysis: a) long half-life PDE5 inhibitors using NGSP units; b) short half-life PDE5 inhibitors using NGSP units; c) long half-life PDE5 inhibitors using IFCC units; d) short half-life PDE5 inhibitors using IFCC units.

a)

Study or Subgroup	PDE5Inhibitor Mean	SD	Total	Control Mean	SD	Total	Weight	Mean Difference IV, Random, 95% CI
**17·1·1 Trials Meeting Subgroup Criteria**								
Fryk 2023	6·21	0·51	18	6·44	0·66	18	13·9%	-0·23 [-0·62, 0·16]
Hegazy 2024	8·78	1·9	30	11·59	2·32	30	4·5%	-2·81 [-3·88, -1·74]
Khazaal 2014	7·41	0·33	18	7·69	0·35	17	17·3%	-0·28 [-0·51, -0·05]
Lee 2022	6·64	0·46	23	7·29	0·31	45	17·6%	-0·65 [-0·86, -0·44]
**Subtotal (95% CI)**			**89**			**110**	**53·3%**	**-0·69 [-1·18, -0·21]**
Heterogeneity: Tau² = 0·19; Chi² = 25·29, df = 3 (P < 0·0001); I² = 88%								
Test for overall effect: Z = 2·82 (P = 0·005)								
**17·1·2 Other Trials**								
Pofi 2022	6·64	0·37	55	6·86	0·37	43	18·7%	-0·22 [-0·37, -0·07]
Sáenz de Tejada 2002	8·07	1·34	136	8·21	1·48	55	12·4%	-0·14 [-0·59, 0·31]
Scheele 2016	7·11	1·14	164	7·25	1·02	62	15·6%	-0·14 [-0·45, 0·17]
**Subtotal (95% CI)**			**355**			**160**	**46·7%**	**-0·20 [-0·33, -0·07]**
Heterogeneity: Tau² = 0·00; Chi² = 0·28, df = 2 (P = 0·87); I² = 0%								
Test for overall effect: Z = 3·07 (P = 0·002)								
**Total (95% CI)**			**444**			**270**	**100·0%**	**-0·40 [-0·66, -0·14]**
Heterogeneity: Tau² = 0·09; Chi² = 34·27, df = 6 (P < 0·00001); I² = 82%								
Test for overall effect: Z = 3·03 (P = 0·002)								
Test for subgroup differences: Chi² = 3·77, df = 1 (P = 0·05), I² = 73·5%								

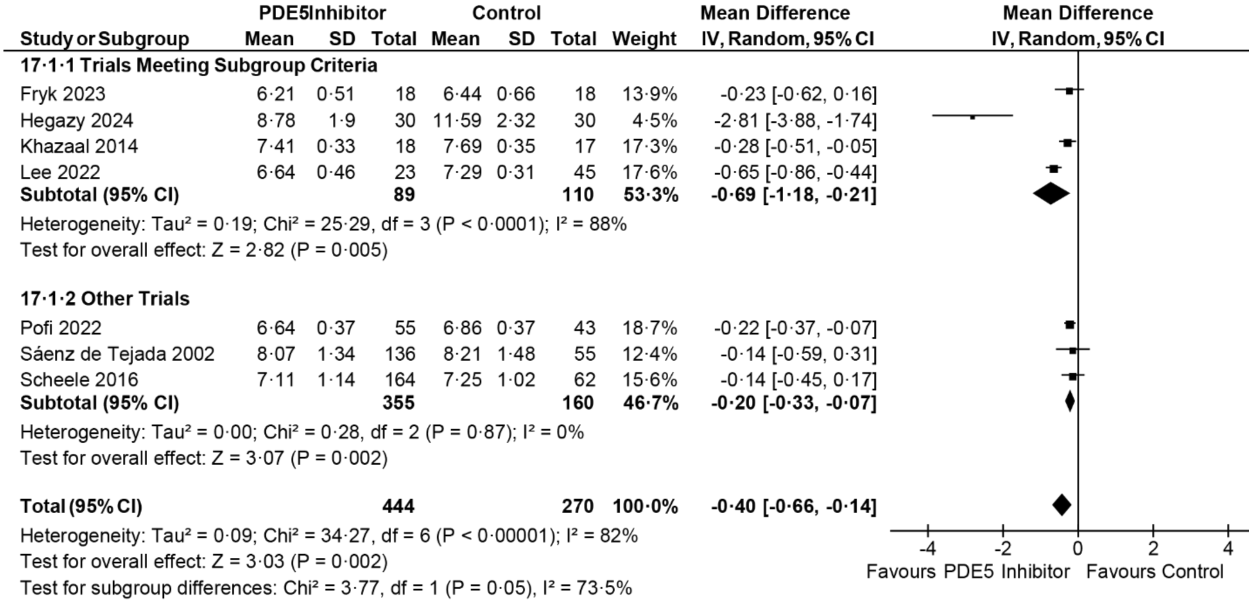

b)

Study or Subgroup	PDE5Inhibitor Mean	SD	Total	Control Mean	SD	Total	Weight	Mean Difference IV, Random, 95% CI
**17·2·1 Trials Meeting Subgroup Criteria**								
Derosa 2022	7·4	1	30	7·3	0·9	30	23·1%	0·10 [-0·38, 0·58]
Giannetta 2012	7·27	1·08	25	6·67	1·08	29	17·4%	0·60 [0·02, 1·18]
Kirilmaz 2015	8·2	1·5	41	7·8	1·8	42	12·3%	0·40 [-0·31, 1·11]
Morano 2007	8	2·5	8	6·5	1·5	8	1·7%	1·50 [-0·52, 3·52]
**Subtotal (95% CI)**			**104**			**109**	**54·6%**	**0·36 [0·03, 0·68]**
Heterogeneity: Tau² = 0·00; Chi² = 3·02, df = 3 (P = 0·39); I² = 1%								
Test for overall effect: Z = 2·14 (P = 0·03)								
**17·2·2 Other Trials**								
Grover-Páez 2007	7·49	2·57	20	7·67	0·24	20	5·3%	-0·18 [-1·31, 0·95]
Liu 2016	6	0·45	26	6·03	0·68	26	40·1%	-0·03 [-0·34, 0·28]
**Subtotal (95% CI)**			**46**			**46**	**45·4%**	**-0·04 [-0·34, 0·26]**
Heterogeneity: Tau² = 0·00; Chi² = 0·06, df = 1 (P = 0·80); I² = 0%								
Test for overall effect: Z = 0·26 (P = 0·79)								
**Total (95% CI)**			**150**			**155**	**100·0%**	**0·18 [-0·09, 0·45]**
Heterogeneity: Tau² = 0·02; Chi² = 6·15, df = 5 (P = 0·29); I² = 19%								
Test for overall effect: Z = 1·32 (P = 0·19)								
Test for subgroup differences: Chi² = 3·07, df = 1 (P = 0·08), I² = 67·4%								

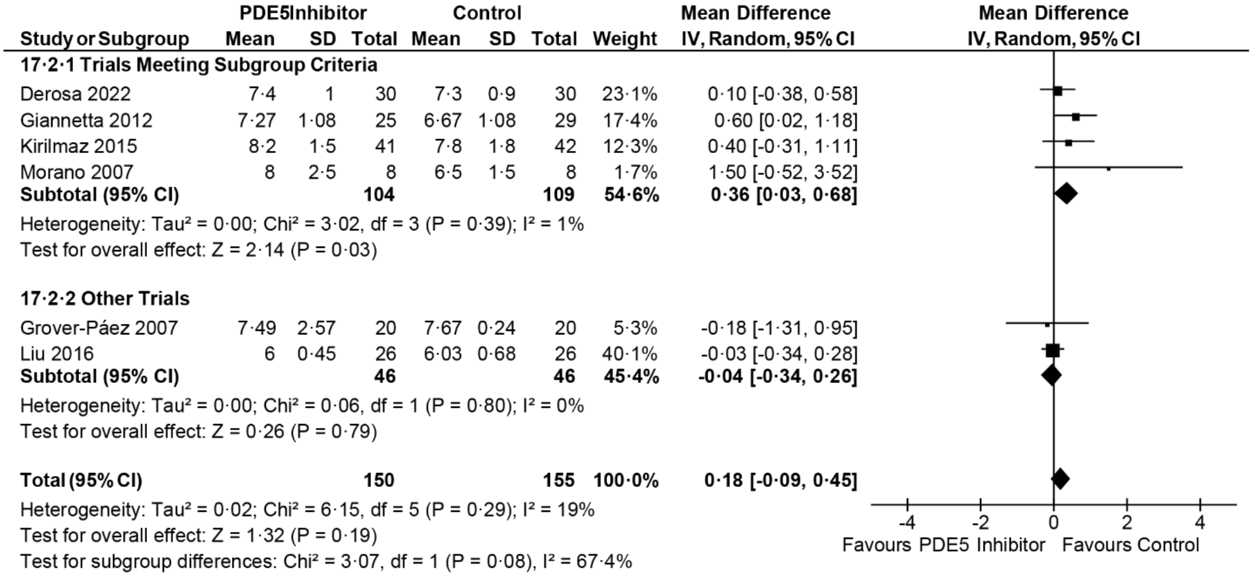

c)

Study or Subgroup	PDE5Inhibitor Mean	SD	Total	Control Mean	SD	Total	Weight	Mean Difference IV, Random, 95% CI
**17·3·1 Trials Meeting Subgroup Criteria**								
Fryk 2023	44·4	5·5	18	46·9	7·2	18	13·9%	-2·50 [-6·69, 1·69]
Hegazy 2024	72·5	20·8	30	103·2	25·4	30	4·6%	-30·70 [-42·45, -18·95]
Khazaal 2014	57·5	3·6	18	60·5	3·9	17	17·2%	-3·00 [-5·49, -0·51]
Lee 2022	49·1	5	23	56·2	3·4	45	17·6%	-7·10 [-9·37, -4·83]
**Subtotal (95% CI)**			**89**			**110**	**53·3%**	**-7·56 [-12·84, -2·28]**
Heterogeneity: Tau² = 22·40; Chi² = 25·36, df = 3 (P < 0·0001); I² = 88%								
Test for overall effect: Z = 2·81 (P = 0·005)								
**17·3·2 Other Trials**								
Pofi 2022	49·1	4	55	51·4	4	43	18·7%	-2·30 [-3·90, -0·70]
Sáenz de Tejada 2002	64·7	14·7	136	66·3	16·1	55	12·5%	-1·60 [-6·52, 3·32]
Scheele 2016	54·2	12·5	164	55·7	11·1	62	15·6%	-1·50 [-4·86, 1·86]
**Subtotal (95% CI)**			**355**			**160**	**46·7%**	**-2·11 [-3·49, -0·73]**
Heterogeneity: Tau² = 0·00; Chi² = 0·22, df = 2 (P = 0·89); I² = 0%								
Test for overall effect: Z = 2·99 (P = 0·003)								
**Total (95% CI)**			**444**			**270**	**100·0%**	**-4·38 [-7·23, -1·52]**
Heterogeneity: Tau² = 10·72; Chi² = 34·71, df = 6 (P < 0·00001); I² = 83%								
Test for overall effect: Z = 3·00 (P = 0·003)								
Test for subgroup differences: Chi² = 3·83, df = 1 (P = 0·05), I² = 73·9%								

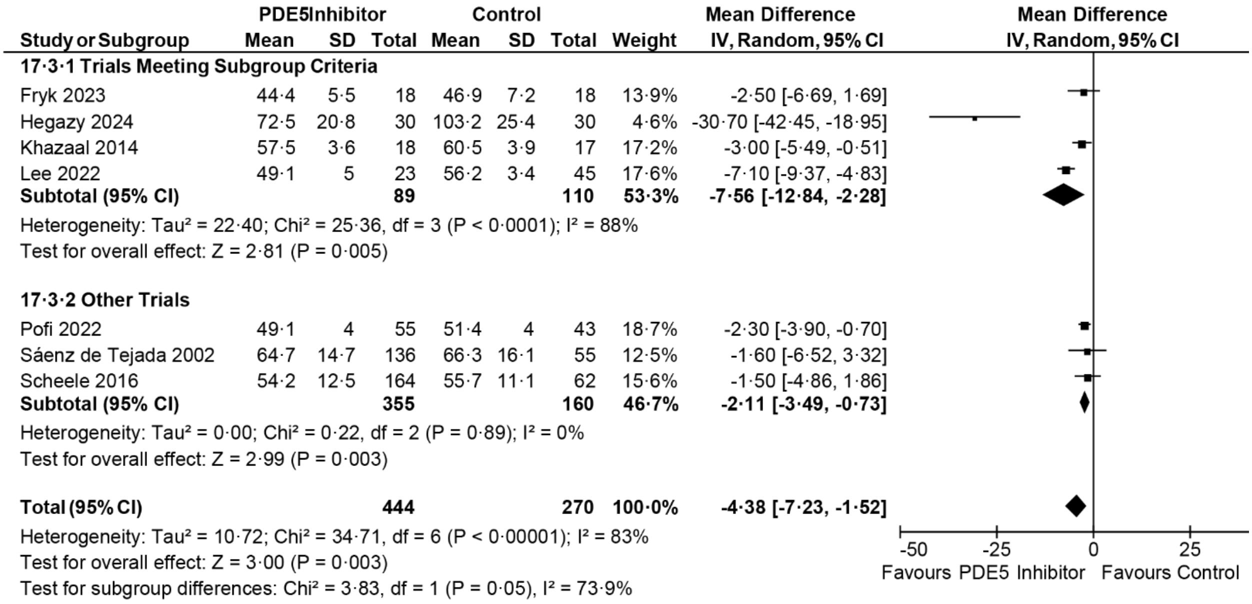

d)

Study or Subgroup	PDE5Inhibitor Mean	SD	Total	Control Mean	SD	Total	Weight	Mean Difference IV, Random, 95% CI
**17·4·1 Trials Meeting Subgroup Criteria**								
Derosa 2022	57·4	10·9	30	56·3	9·8	30	23·1%	1·10 [-4·15, 6·35]
Giannetta 2012	56	11·8	25	49·4	11·8	29	17·4%	6·60 [0·29, 12·91]
Kirilmaz 2015	66·1	16·4	41	61·8	19·7	42	12·3%	4·30 [-3·49, 12·09]
Morano 2007	63·9	27·3	8	47·5	16·4	8	1·7%	16·40 [-5·67, 38·47]
**Subtotal (95% CI)**			**104**			**109**	**54·5%**	**3·90 [0·32, 7·47]**
Heterogeneity: Tau² = 0·20; Chi² = 3·04, df = 3 (P = 0·39); I² = 1%								
Test for overall effect: Z = 2·14 (P = 0·03)								
**17·4·2 Other Trials**								
Grover-Páez 2007	58·4	28·1	20	60·4	2·6	20	5·3%	-2·00 [-14·37, 10·37]
Liu 2016	42·1	4·9	26	42·4	7·4	26	40·2%	-0·30 [-3·71, 3·11]
**Subtotal (95% CI)**			**46**			**46**	**45·5%**	**-0·42 [-3·71, 2·87]**
Heterogeneity: Tau² = 0·00; Chi² = 0·07, df = 1 (P = 0·80); I² = 0%								
Test for overall effect: Z = 0·25 (P = 0·80)								
**Total (95% CI)**			**150**			**155**	**100·0%**	**1·99 [-0·95, 4·93]**
Heterogeneity: Tau² = 2·57; Chi² = 6·15, df = 5 (P = 0·29); I² = 19%								
Test for overall effect: Z = 1·32 (P = 0·19)								
Test for subgroup differences: Chi² = 3·03, df = 1 (P = 0·08), I² = 67·0%								

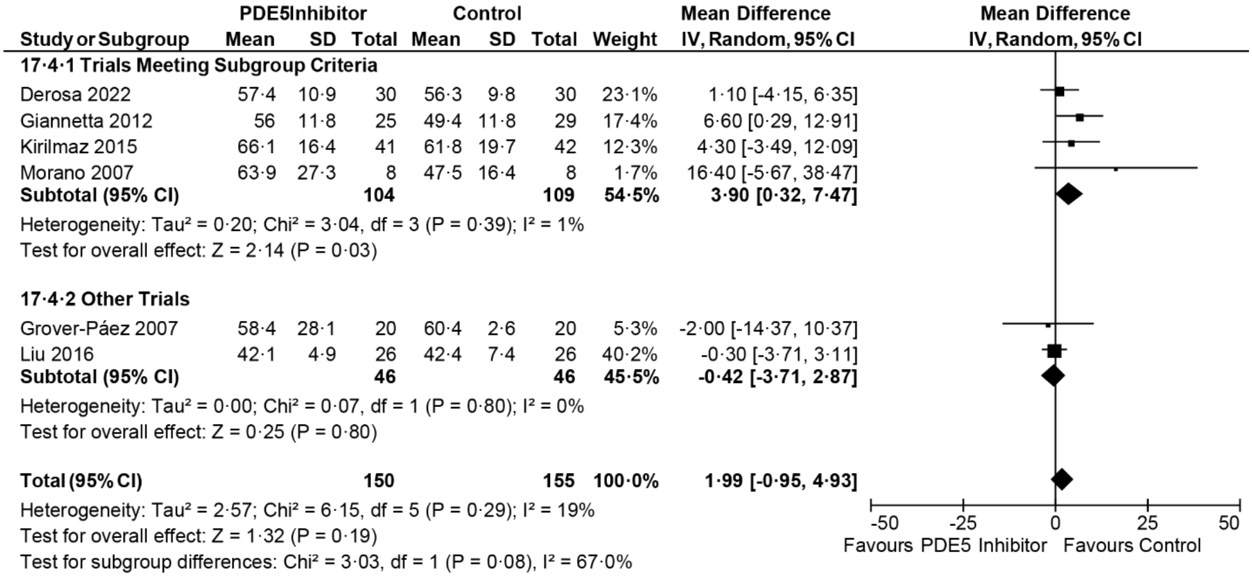

Supplementary Fig. 23: Sensitivity analysis of meta-analysis excluding subjective outliers

Sensitivity analysis was performed on a potential decision to include all trials regardless of whether they subjectively appears to be outliers, as well as on a potential decision to represent data in IFCC units (mmol/mol) or NGSP units (%). The following conditions were used for each meta-analysis: a) long half-life PDE5 inhibitors using NGSP units; b) short half-life PDE5 inhibitors using NGSP units; c) long half-life PDE5 inhibitors using IFCC units; d) short half-life PDE5 inhibitors using IFCC units.

a)

	PDE5Inhibitor			Control				Mean Difference
Study or Subgroup	Mean	SD	Total	Mean	SD	Total	Weight	IV, Random, 95% CI
**18·1·1 Subjective Outliers**								
Hegazy 2024	8·78	1·9	30	11·59	2·32	30	4·5%	-2·81 [-3·88, -1·74]
**Subtotal (95% CI)**			**30**			**30**	**4·5%**	**-2·81 [-3·88, -1·74]**
Heterogeneity: Not applicable								
Test for overall effect: Z = 5·13 (P < 0·00001)								
**18·1·2 Other Trials**								
Fryk 2023	6·21	0·51	18	6·44	0·66	18	13·9%	-0·23 [-0·62, 0·16]
Khazaal 2014	7·41	0·33	18	7·69	0·35	17	17·3%	-0·28 [-0·51, -0·05]
Lee 2022	6·64	0·46	23	7·29	0·31	45	17·6%	-0·65 [-0·86, -0·44]
Pofi 2022	6·64	0·37	55	6·86	0·37	43	18·7%	-0·22 [-0·37, -0·07]
Sáenz de Tejada 2002	8·07	1·34	136	8·21	1·48	55	12·4%	-0·14 [-0·59, 0·31]
Scheele 2016	7·11	1·14	164	7·25	1·02	62	15·6%	-0·14 [-0·45, 0·17]
**Subtotal (95% CI)**			**414**			**240**	**95·5%**	**-0·30 [-0·47, -0·13]**
Heterogeneity: Tau² = 0·03; Chi² = 13·57, df = 5 (P = 0·02); I² = 63%								
Test for overall effect: Z = 3·39 (P = 0·0007)								
**Total (95% CI)**			**444**			**270**	**100·0%**	**-0·40 [-0·66, -0·14]**
Heterogeneity: Tau² = 0·09; Chi² = 34·27, df = 6 (P < 0·00001); I² = 82%								
Test for overall effect: Z = 3·03 (P = 0·002)								
Test for subgroup differences: Chi² = 20·48, df = 1 (P < 0·00001), I² = 95·1%								

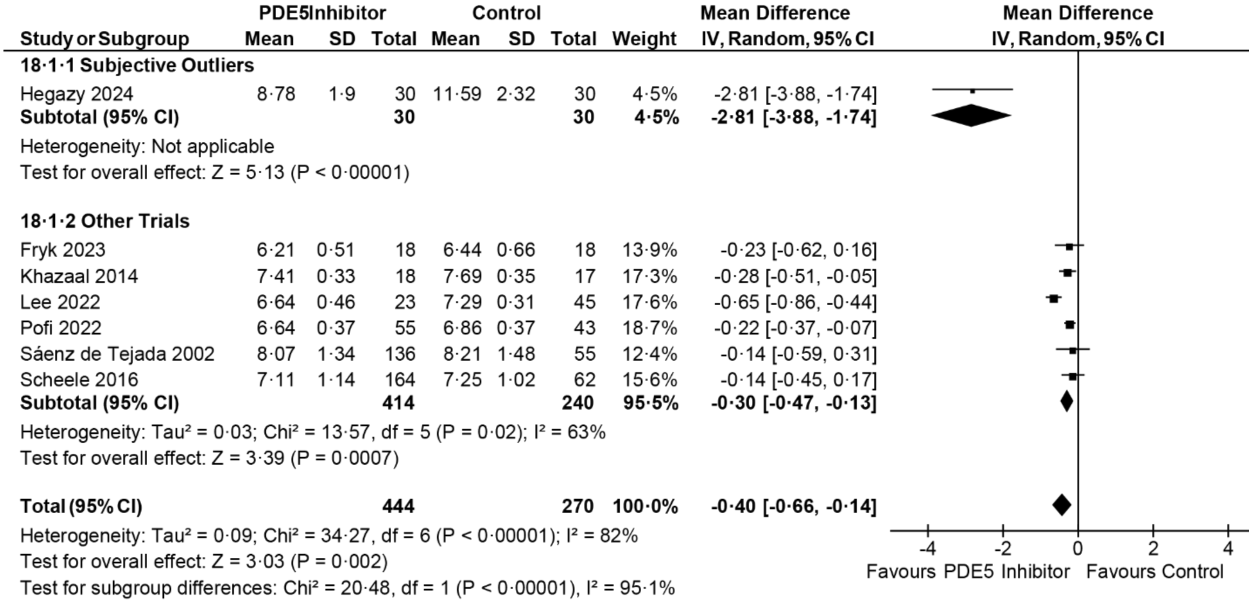

b)

	PDE5Inhibitor			Control				Mean Difference
Study or Subgroup	Mean	SD	Total	Mean	SD	Total	Weight	IV, Random, 95% CI
**18·2·1 Subjective Outliers**								
Morano 2007	8	2·5	8	6·5	1·5	8	1·7%	1·50 [-0·52, 3·52]
**Subtotal (95% CI)**			**8**			**8**	**1·7%**	**1·50 [-0·52, 3·52]**
Heterogeneity: Not applicable								
Test for overall effect: Z = 1·46 (P = 0·15)								
**18·2·2 Other Trials**								
Derosa 2022	7·4	1	30	7·3	0·9	30	23·1%	0·10 [-0·38, 0·58]
Giannetta 2012	7·27	1·08	25	6·67	1·08	29	17·4%	0·60 [0·02, 1·18]
Grover-Páez 2007	7·49	2·57	20	7·67	0·24	20	5·3%	-0·18 [-1·31, 0·95]
Kirilmaz 2015	8·2	1·5	41	7·8	1·8	42	12·3%	0·40 [-0·31, 1·11]
Liu 2016	6	0·45	26	6·03	0·68	26	40·1%	-0·03 [-0·34, 0·28]
**Subtotal (95% CI)**			**142**			**147**	**98·3%**	**0·14 [-0·10, 0·38]**
Heterogeneity: Tau² = 0·01; Chi² = 4·40, df = 4 (P = 0·35); I² = 9%								
Test for overall effect: Z = 1·14 (P = 0·25)								
**Total (95% CI)**			**150**			**155**	**100·0%**	**0·18 [-0·09, 0·45]**
Heterogeneity: Tau² = 0·02; Chi² = 6·15, df = 5 (P = 0·29); I² = 19%								
Test for overall effect: Z = 1·32 (P = 0·19)								
Test for subgroup differences: Chi² = 1·71, df = 1 (P = 0·19), I² = 41·6%								

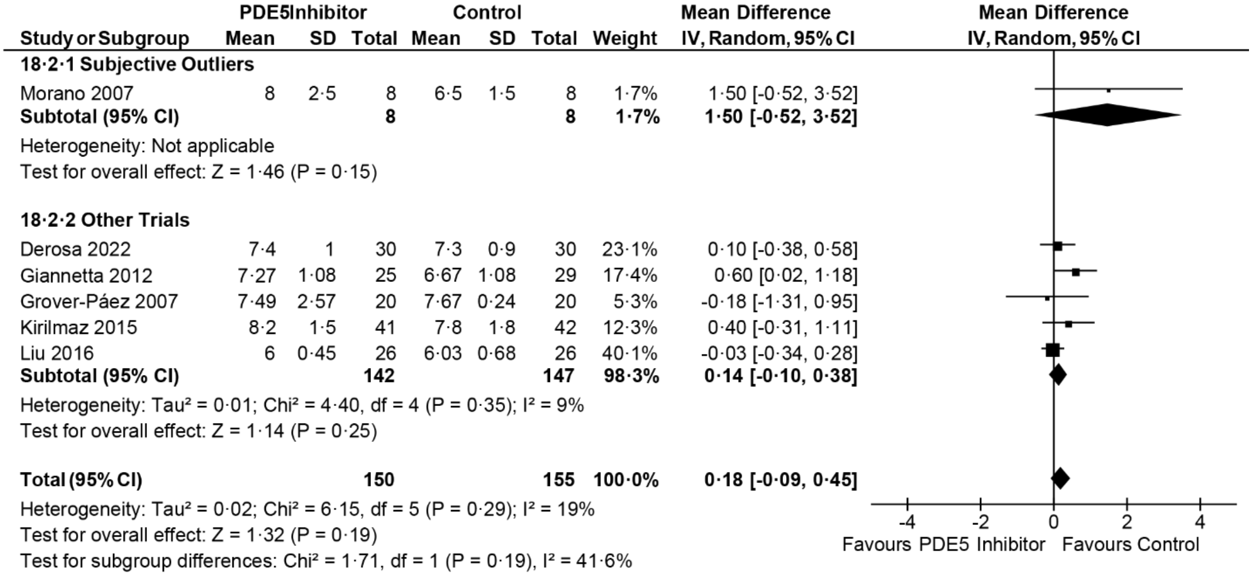

c)

Study or Subgroup	PDE5 Inhibitor Mean	PDE5 Inhibitor SD	PDE5 Inhibitor Total	Control Mean	Control SD	Control Total	Weight	Mean Difference IV, Random, 95% CI
**18·3·1 Subjective Outliers**								
Hegazy 2024	72·5	20·8	30	103·2	25·4	30	4·6%	-30·70 [-42·45, -18·95]
**Subtotal (95% CI)**			**30**			**30**	**4·6%**	**-30·70 [-42·45, -18·95]**
Heterogeneity: Not applicable								
Test for overall effect: Z = 5·12 (P < 0·00001)								
**18·3·2 Other Trials**								
Fryk 2023	44·4	5·5	18	46·9	7·2	18	13·9%	-2·50 [-6·69, 1·69]
Khazaal 2014	57·5	3·6	18	60·5	3·9	17	17·2%	-3·00 [-5·49, -0·51]
Lee 2022	49·1	5	23	56·2	3·4	45	17·6%	-7·10 [-9·37, -4·83]
Pofi 2022	49·1	4	55	51·4	4	43	18·7%	-2·30 [-3·90, -0·70]
Sáenz de Tejada 2002	64·7	14·7	136	66·3	16·1	55	12·5%	-1·60 [-6·52, 3·32]
Scheele 2016	54·2	12·5	164	55·7	11·1	62	15·6%	-1·50 [-4·86, 1·86]
**Subtotal (95% CI)**			**414**			**240**	**95·4%**	**-3·24 [-5·16, -1·32]**
Heterogeneity: Tau² = 3·41; Chi² = 14·01, df = 5 (P = 0·02); I² = 64%								
Test for overall effect: Z = 3·30 (P = 0·0010)								
**Total (95% CI)**			**444**			**270**	**100·0%**	**-4·38 [-7·23, -1·52]**
Heterogeneity: Tau² = 10·72; Chi² = 34·71, df = 6 (P < 0·00001); I² = 83%								
Test for overall effect: Z = 3·00 (P = 0·003)								
Test for subgroup differences: Chi² = 20·45, df = 1 (P < 0·00001), I² = 95·1%								

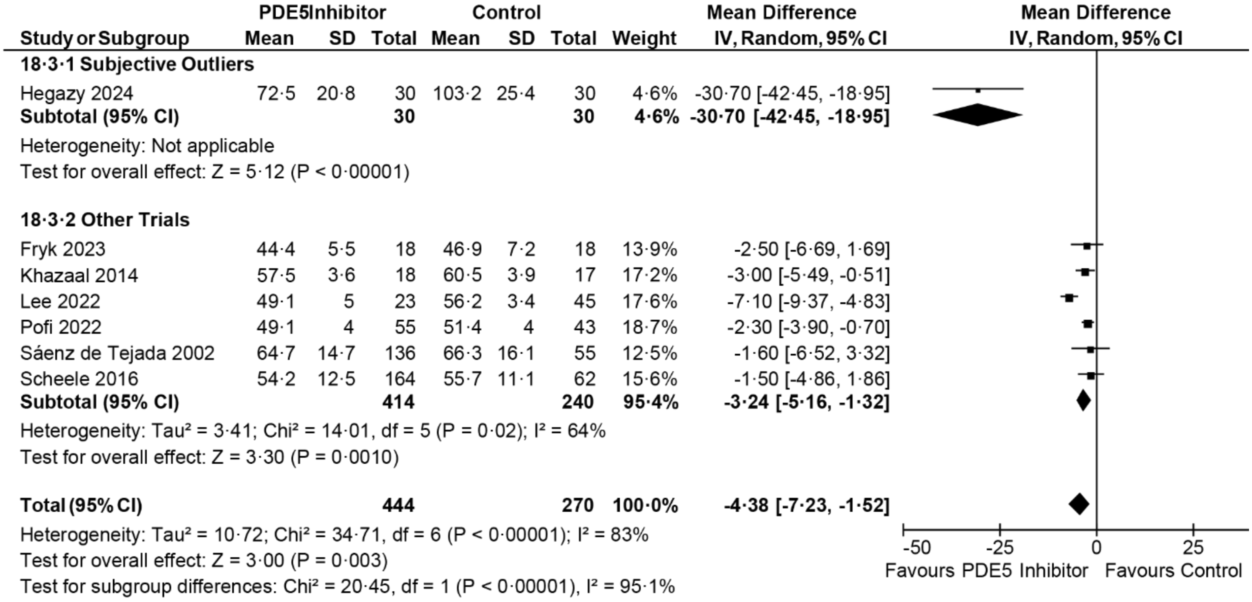

d)

Study or Subgroup	PDE5 Inhibitor Mean	PDE5 Inhibitor SD	PDE5 Inhibitor Total	Control Mean	Control SD	Control Total	Weight	Mean Difference IV, Random, 95% CI
**18·4·1 Subjective Outliers**								
Morano 2007	63·9	27·3	8	47·5	16·4	8	1·7%	16·40 [-5·67, 38·47]
**Subtotal (95% CI)**			**8**			**8**	**1·7%**	**16·40 [-5·67, 38·47]**
Heterogeneity: Not applicable								
Test for overall effect: Z = 1·46 (P = 0·15)								
**18·4·2 Other Trials**								
Derosa 2022	57·4	10·9	30	56·3	9·8	30	23·1%	1·10 [-4·15, 6·35]
Giannetta 2012	56	11·8	25	49·4	11·8	29	17·4%	6·60 [0·29, 12·91]
Grover-Páez 2007	58·4	28·1	20	60·4	2·6	20	5·3%	-2·00 [-14·37, 10·37]
Kirilmaz 2015	66·1	16·4	41	61·8	19·7	42	12·3%	4·30 [-3·49, 12·09]
Liu 2016	42·1	4·9	26	42·4	7·4	26	40·2%	-0·30 [-3·71, 3·11]
**Subtotal (95% CI)**			**142**			**147**	**98·3%**	**1·55 [-1·09, 4·19]**
Heterogeneity: Tau² = 0·91; Chi² = 4·40, df = 4 (P = 0·36); I² = 9%								
Test for overall effect: Z = 1·15 (P = 0·25)								
**Total (95% CI)**			**150**			**155**	**100·0%**	**1·99 [-0·95, 4·93]**
Heterogeneity: Tau² = 2·57; Chi² = 6·15, df = 5 (P = 0·29); I² = 19%								
Test for overall effect: Z = 1·32 (P = 0·19)								
Test for subgroup differences: Chi² = 1·72, df = 1 (P = 0·19), I² = 41·7%								

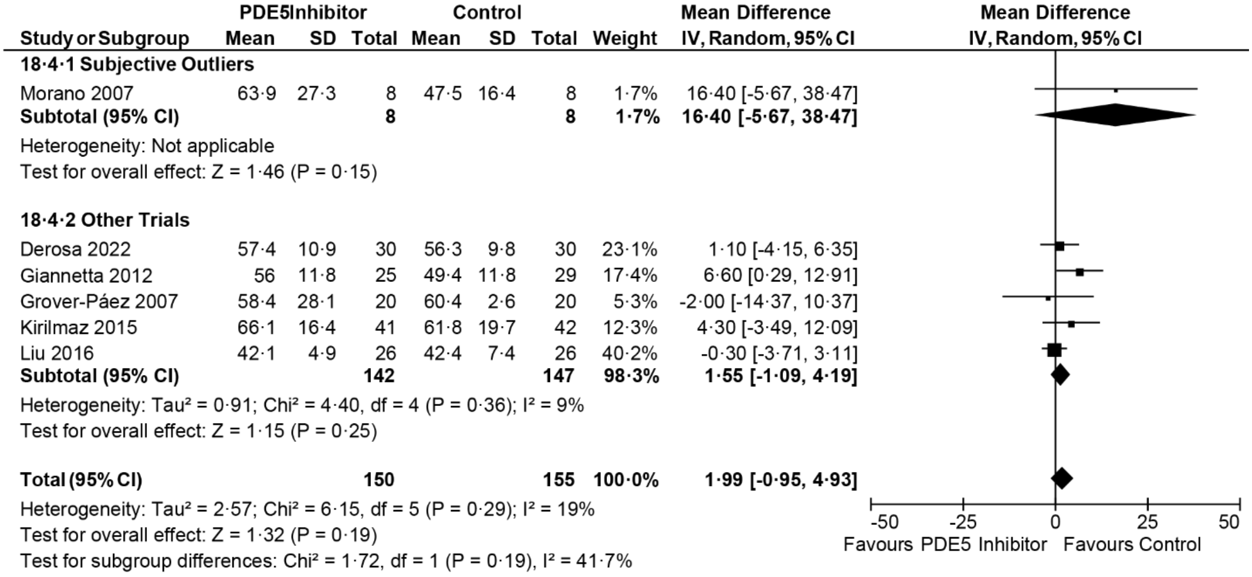

Supplementary Fig. 24: Sensitivity analysis of meta-analysis subgroup analysis excluding subjective outliers

Sensitivity analysis was performed on a potential decision to perform subgroup analysis on trials regardless of whether they subjectively appears to be outliers, as well as on a potential decision to represent data in IFCC units (mmol/mol) or NGSP units (%). The following conditions were used for each meta-analysis: a) long half-life PDE5 inhibitors using NGSP units; b) short half-life PDE5 inhibitors using NGSP units; c) long half-life PDE5 inhibitors using IFCC units; d) short half-life PDE5 inhibitors using IFCC units.

a)

	PDE5Inhibitor			Control				Mean Difference
Study or Subgroup	Mean	SD	Total	Mean	SD	Total	Weight	IV, Random, 95% CI
**19·1·1 Subjective Outliers**								
Hegazy 2024	8·78	1·9	30	11·59	2·32	30	7·0%	-2·81 [-3·88, -1·74]
**Subtotal (95% CI)**			**30**			**30**	**7·0%**	**-2·81 [-3·88, -1·74]**
Heterogeneity: Not applicable								
Test for overall effect: Z = 5·13 (P < 0·00001)								
**19·1·2 Other Trials**								
Khazaal 2014	7·41	0·33	18	7·69	0·35	17	23·3%	-0·28 [-0·51, -0·05]
Lee 2022	6·64	0·46	23	7·29	0·31	45	23·6%	-0·65 [-0·86, -0·44]
Pofi 2022	6·64	0·37	55	6·86	0·37	43	24·8%	-0·22 [-0·37, -0·07]
Scheele 2016	7·11	1·14	164	7·25	1·02	62	21·3%	-0·14 [-0·45, 0·17]
**Subtotal (95% CI)**			**260**			**167**	**93·0%**	**-0·33 [-0·55, -0·11]**
Heterogeneity: Tau² = 0·04; Chi² = 12·80, df = 3 (P = 0·005); I² = 77%								
Test for overall effect: Z = 2·93 (P = 0·003)								
**Total (95% CI)**			**290**			**197**	**100·0%**	**-0·50 [-0·83, -0·17]**
Heterogeneity: Tau² = 0·11; Chi² = 33·25, df = 4 (P < 0·00001); I² = 88%								
Test for overall effect: Z = 2·95 (P = 0·003)								
Test for subgroup differences: Chi² = 19·69, df = 1 (P < 0·00001), I² = 94·9%								

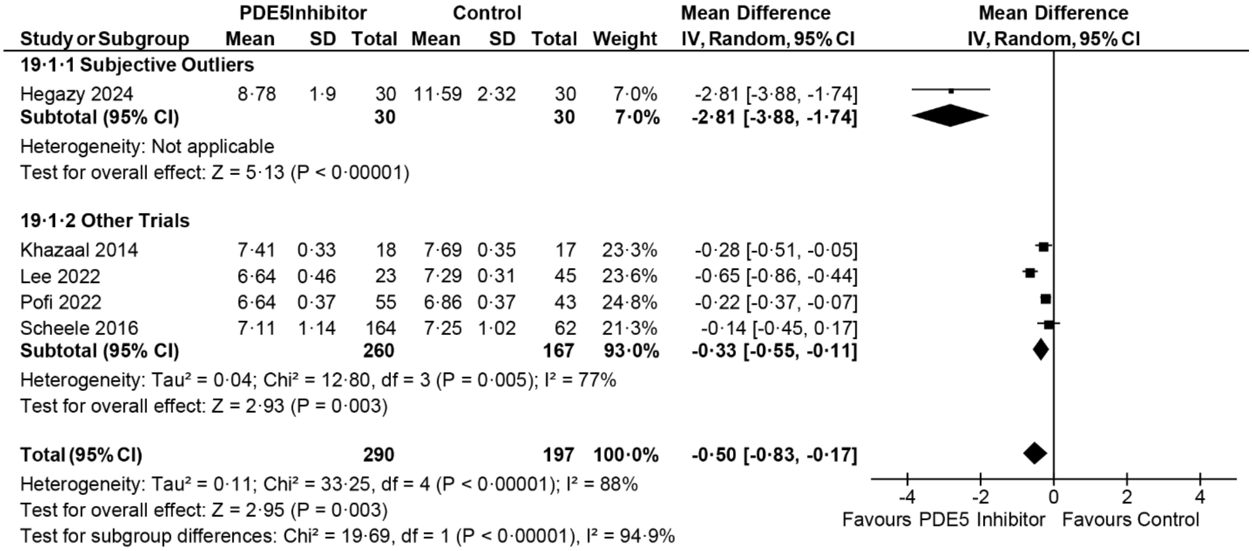

b)

	PDE5Inhibitor			Control				Mean Difference
Study or Subgroup	Mean	SD	Total	Mean	SD	Total	Weight	IV, Random, 95% CI
**19·2·1 Subjective Outliers**								
Morano 2007	8	2·5	8	6·5	1·5	8	2·6%	1·50 [-0·52, 3·52]
**Subtotal (95% CI)**			**8**			**8**	**2·6%**	**1·50 [-0·52, 3·52]**
Heterogeneity: Not applicable								
Test for overall effect: Z = 1·46 (P = 0·15)								
**19·2·2 Other Trials**								
Derosa 2022	7·4	1	30	7·3	0·9	30	45·2%	0·10 [-0·38, 0·58]
Giannetta 2012	7·27	1·08	25	6·67	1·08	29	31·5%	0·60 [0·02, 1·18]
Kirilmaz 2015	8·2	1·5	41	7·8	1·8	42	20·8%	0·40 [-0·31, 1·11]
**Subtotal (95% CI)**			**96**			**101**	**97·4%**	**0·33 [-0·00, 0·65]**
Heterogeneity: Tau² = 0·00; Chi² = 1·75, df = 2 (P = 0·42); I² = 0%								
Test for overall effect: Z = 1·94 (P = 0·05)								
**Total (95% CI)**			**104**			**109**	**100·0%**	**0·36 [0·03, 0·68]**
Heterogeneity: Tau² = 0·00; Chi² = 3·02, df = 3 (P = 0·39); I² = 1%								
Test for overall effect: Z = 2·14 (P = 0·03)								
Test for subgroup differences: Chi² = 1·27, df = 1 (P = 0·26), I² = 21·0%								

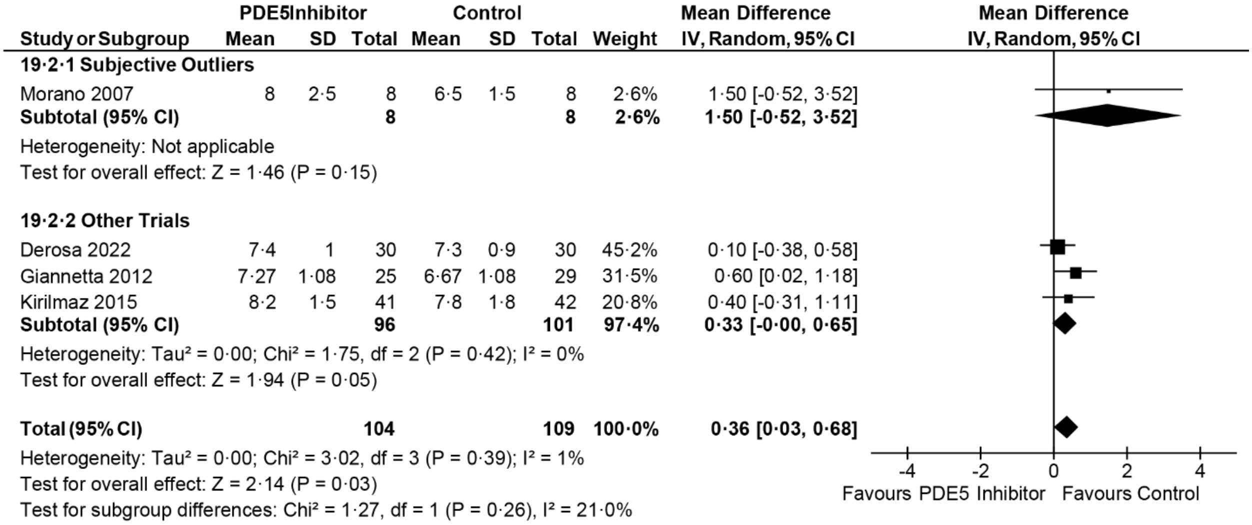

c)

Study or Subgroup	PDE5Inhibitor Mean	SD	Total	Control Mean	SD	Total	Weight	Mean Difference IV, Random, 95% CI
**19·3·1 Subjective Outliers**								
Hegazy 2024	72·5	20·8	30	103·2	25·4	30	7·1%	-30·70 [-42·45, -18·95]
**Subtotal (95% CI)**			**30**			**30**	**7·1%**	**-30·70 [-42·45, -18·95]**
Heterogeneity: Not applicable								
Test for overall effect: Z = 5·12 (P < 0·00001)								
**19·3·2 Other Trials**								
Khazaal 2014	57·5	3·6	18	60·5	3·9	17	23·2%	-3·00 [-5·49, -0·51]
Lee 2022	49·1	5	23	56·2	3·4	45	23·6%	-7·10 [-9·37, -4·83]
Pofi 2022	49·1	4	55	51·4	4	43	24·8%	-2·30 [-3·90, -0·70]
Scheele 2016	54·2	12·5	164	55·7	11·1	62	21·3%	-1·50 [-4·86, 1·86]
**Subtotal (95% CI)**			**260**			**167**	**92·9%**	**-3·55 [-6·00, -1·09]**
Heterogeneity: Tau² = 4·75; Chi² = 13·33, df = 3 (P = 0·004); I² = 77%								
Test for overall effect: Z = 2·83 (P = 0·005)								
**Total (95% CI)**			**290**			**197**	**100·0%**	**-5·44 [-9·10, -1·77]**
Heterogeneity: Tau² = 13·47; Chi² = 33·80, df = 4 (P < 0·00001); I² = 88%								
Test for overall effect: Z = 2·91 (P = 0·004)								
Test for subgroup differences: Chi² = 19·66, df = 1 (P < 0·00001), I² = 94·9%								

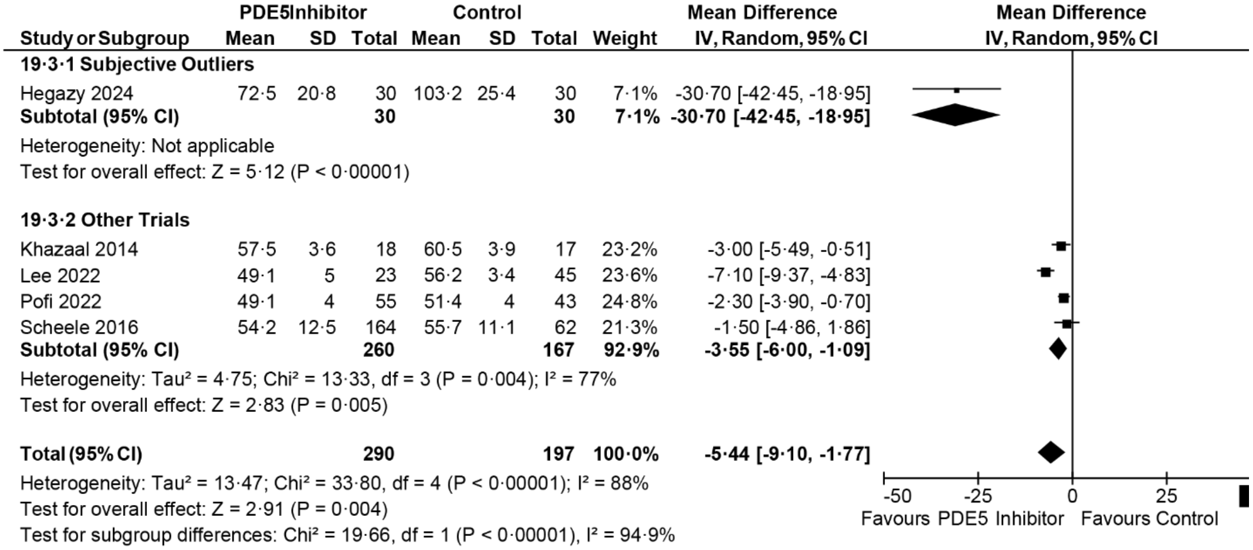

d)

Study or Subgroup	PDE5Inhibitor Mean	SD	Total	Control Mean	SD	Total	Weight	Mean Difference IV, Random, 95% CI
**19·4·1 Subjective Outliers**								
Morano 2007	63·9	27·3	8	47·5	16·4	8	2·6%	16·40 [-5·67, 38·47]
**Subtotal (95% CI)**			**8**			**8**	**2·6%**	**16·40 [-5·67, 38·47]**
Heterogeneity: Not applicable								
Test for overall effect: Z = 1·46 (P = 0·15)								
**19·4·2 Other Trials**								
Derosa 2022	57·4	10·9	30	56·3	9·8	30	45·2%	1·10 [-4·15, 6·35]
Giannetta 2012	56	11·8	25	49·4	11·8	29	31·4%	6·60 [0·29, 12·91]
Kirilmaz 2015	66·1	16·4	41	61·8	19·7	42	20·8%	4·30 [-3·49, 12·09]
**Subtotal (95% CI)**			**96**			**101**	**97·4%**	**3·55 [-0·03, 7·13]**
Heterogeneity: Tau² = 0·00; Chi² = 1·77, df = 2 (P = 0·41); I² = 0%								
Test for overall effect: Z = 1·94 (P = 0·05)								
**Total (95% CI)**			**104**			**109**	**100·0%**	**3·90 [0·32, 7·47]**
Heterogeneity: Tau² = 0·20; Chi² = 3·04, df = 3 (P = 0·39); I² = 1%								
Test for overall effect: Z = 2·14 (P = 0·03)								
Test for subgroup differences: Chi² = 1·27, df = 1 (P = 0·26), I² = 21·2%								

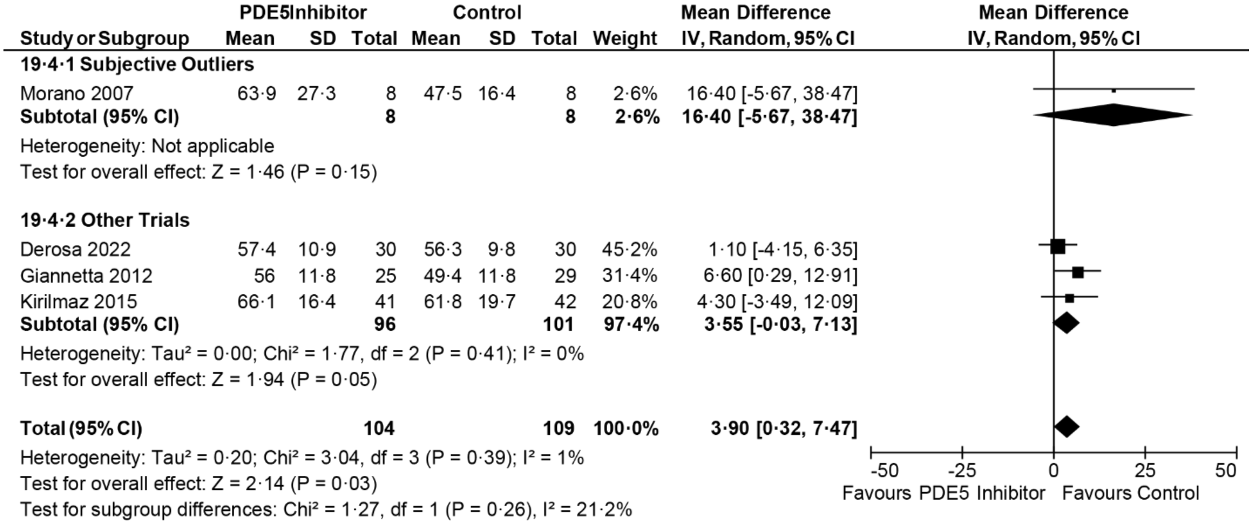

Supplementary Fig. 25: Sensitivity analysis of meta-analysis grouping PDE5 inhibitors by half-life

Sensitivity analysis was performed on a potential decision to group all trials regardless of half-life of the PDE5 inhibitor intervention, as well as on a potential decision to represent data in IFCC units (mmol/mol) or NGSP units (%). The following conditions were used for each meta-analysis: a) all PDE5 inhibitors using NGSP units; b) all PDE5 inhibitors using IFCC units.

a)

	PDE5Inhibitor			Control				Mean Difference
**Study or Subgroup**	**Mean**	**SD**	**Total**	**Mean**	**SD**	**Total**	**Weight**	**IV, Random, 95% CI**
**20·1·1 Long Half Life PDE5 Inhibitor**								
Fryk 2023	6·21	0·51	18	6·44	0·66	18	9·2%	-0·23 [-0·62, 0·16]
Hegazy 2024	8·78	1·9	30	11·59	2·32	30	3·3%	-2·81 [-3·88, -1·74]
Khazaal 2014	7·41	0·33	18	7·69	0·35	17	11·1%	-0·28 [-0·51, -0·05]
Lee 2022	6·64	0·46	23	7·29	0·31	45	11·3%	-0·65 [-0·86, -0·44]
Pofi 2022	6·64	0·37	55	6·86	0·37	43	11·9%	-0·22 [-0·37, -0·07]
Sáenz de Tejada 2002	8·07	1·34	136	8·21	1·48	55	8·3%	-0·14 [-0·59, 0·31]
Scheele 2016	7·11	1·14	164	7·25	1·02	62	10·2%	-0·14 [-0·45, 0·17]
**Subtotal (95% CI)**			**444**			**270**	**65·3%**	**-0·40 [-0·66, -0·14]**
Heterogeneity: Tau² = 0·09; Chi² = 34·27, df = 6 (P < 0·00001); I² = 82%								
Test for overall effect: Z = 3·03 (P = 0·002)								
**20·1·2 Short Half Life PDE5 Inhibitor**								
Derosa 2022	7·4	1	30	7·3	0·9	30	8·0%	0·10 [-0·38, 0·58]
Giannetta 2012	7·27	1·08	25	6·67	1·08	29	6·9%	0·60 [0·02, 1·18]
Grover-Páez 2007	7·49	2·57	20	7·67	0·24	20	3·0%	-0·18 [-1·31, 0·95]
Kirilmaz 2015	8·2	1·5	41	7·8	1·8	42	5·6%	0·40 [-0·31, 1·11]
Liu 2016	6	0·45	26	6·03	0·68	26	10·1%	-0·03 [-0·34, 0·28]
Morano 2007	8	2·5	8	6·5	1·5	8	1·1%	1·50 [-0·52, 3·52]
**Subtotal (95% CI)**			**150**			**155**	**34·7%**	**0·18 [-0·09, 0·45]**
Heterogeneity: Tau² = 0·02; Chi² = 6·15, df = 5 (P = 0·29); I² = 19%								
Test for overall effect: Z = 1·32 (P = 0·19)								
**Total (95% CI)**			**594**			**425**	**100·0%**	**-0·19 [-0·42, 0·04]**
Heterogeneity: Tau² = 0·11; Chi² = 55·25, df = 12 (P < 0·00001); I² = 78%								
Test for overall effect: Z = 1·65 (P = 0·10)								
Test for subgroup differences: Chi² = 9·34, df = 1 (P = 0·002), I² = 89·3%								

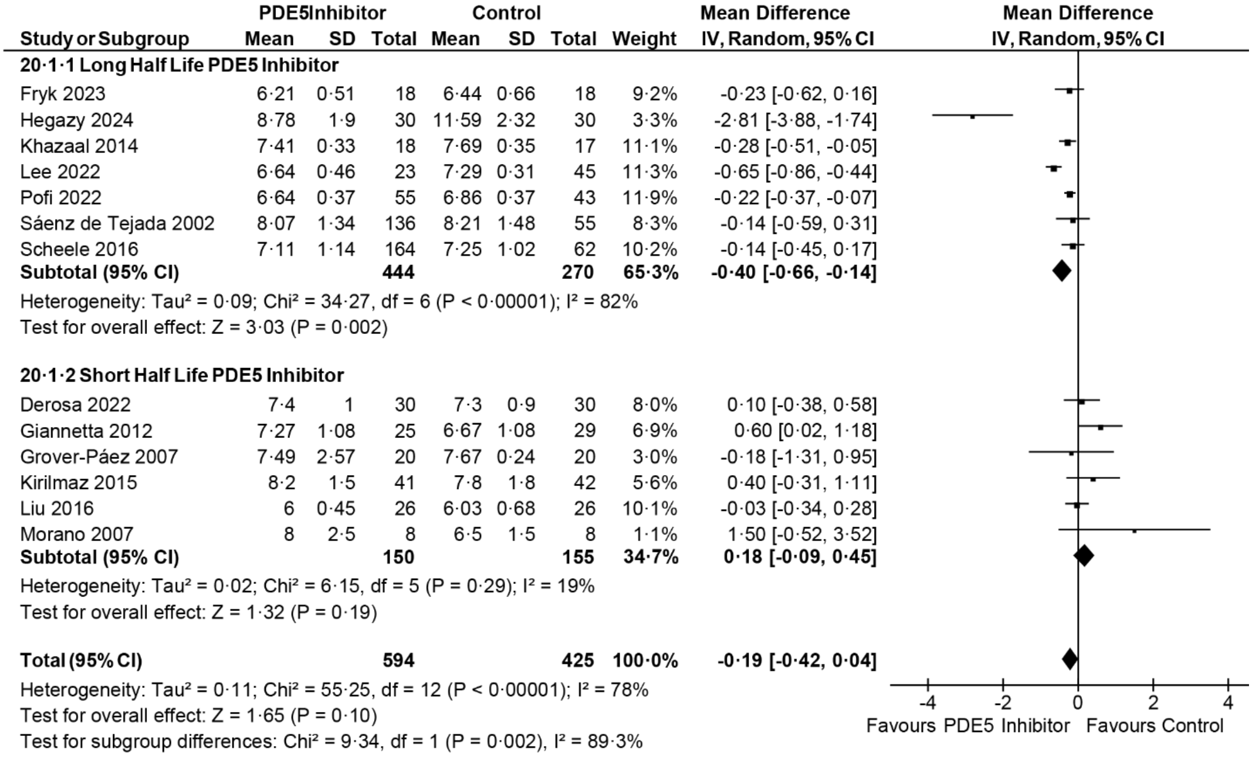

b)

Study or Subgroup	PDE5Inhibitor Mean	SD	Total	Control Mean	SD	Total	Weight	Mean Difference IV, Random, 95% CI
**20·3·1 Long Half Life PDE5 Inhibitor**								
Fryk 2023	44·4	5·5	18	46·9	7·2	18	9·2%	-2·50 [-6·69, 1·69]
Hegazy 2024	72·5	20·8	30	103·2	25·4	30	3·3%	-30·70 [-42·45, -18·95]
Khazaal 2014	57·5	3·6	18	60·5	3·9	17	11·1%	-3·00 [-5·49, -0·51]
Lee 2022	49·1	5	23	56·2	3·4	45	11·3%	-7·10 [-9·37, -4·83]
Pofi 2022	49·1	4	55	51·4	4	43	11·9%	-2·30 [-3·90, -0·70]
Sáenz de Tejada 2002	64·7	14·7	136	66·3	16·1	55	8·4%	-1·60 [-6·52, 3·32]
Scheele 2016	54·2	12·5	164	55·7	11·1	62	10·2%	-1·50 [-4·86, 1·86]
**Subtotal (95% CI)**			**444**			**270**	**65·3%**	**-4·38 [-7·23, -1·52]**
Heterogeneity: Tau² = 10·72; Chi² = 34·71, df = 6 (P < 0·00001); I² = 83%								
Test for overall effect: Z = 3·00 (P = 0·003)								
**20·3·2 Short Half Life PDE5 Inhibitor**								
Derosa 2022	57·4	10·9	30	56·3	9·8	30	8·0%	1·10 [-4·15, 6·35]
Giannetta 2012	56	11·8	25	49·4	11·8	29	6·9%	6·60 [0·29, 12·91]
Grover-Páez 2007	58·4	28·1	20	60·4	2·6	20	3·0%	-2·00 [-14·37, 10·37]
Kirilmaz 2015	66·1	16·4	41	61·8	19·7	42	5·6%	4·30 [-3·49, 12·09]
Liu 2016	42·1	4·9	26	42·4	7·4	26	10·1%	-0·30 [-3·71, 3·11]
Morano 2007	63·9	27·3	8	47·5	16·4	8	1·1%	16·40 [-5·67, 38·47]
**Subtotal (95% CI)**			**150**			**155**	**34·7%**	**1·99 [-0·95, 4·93]**
Heterogeneity: Tau² = 2·57; Chi² = 6·15, df = 5 (P = 0·29); I² = 19%								
Test for overall effect: Z = 1·32 (P = 0·19)								
**Total (95% CI)**			**594**			**425**	**100·0%**	**-2·05 [-4·51, 0·41]**

Heterogeneity: Tau² = 12·59; Chi² = 55·46, df = 12 (P < 0·00001); I² = 78%
Test for overall effect: Z = 1·63 (P = 0·10)
Test for subgroup differences: Chi² = 9·25, df = 1 (P = 0·002), I² = 89·2%

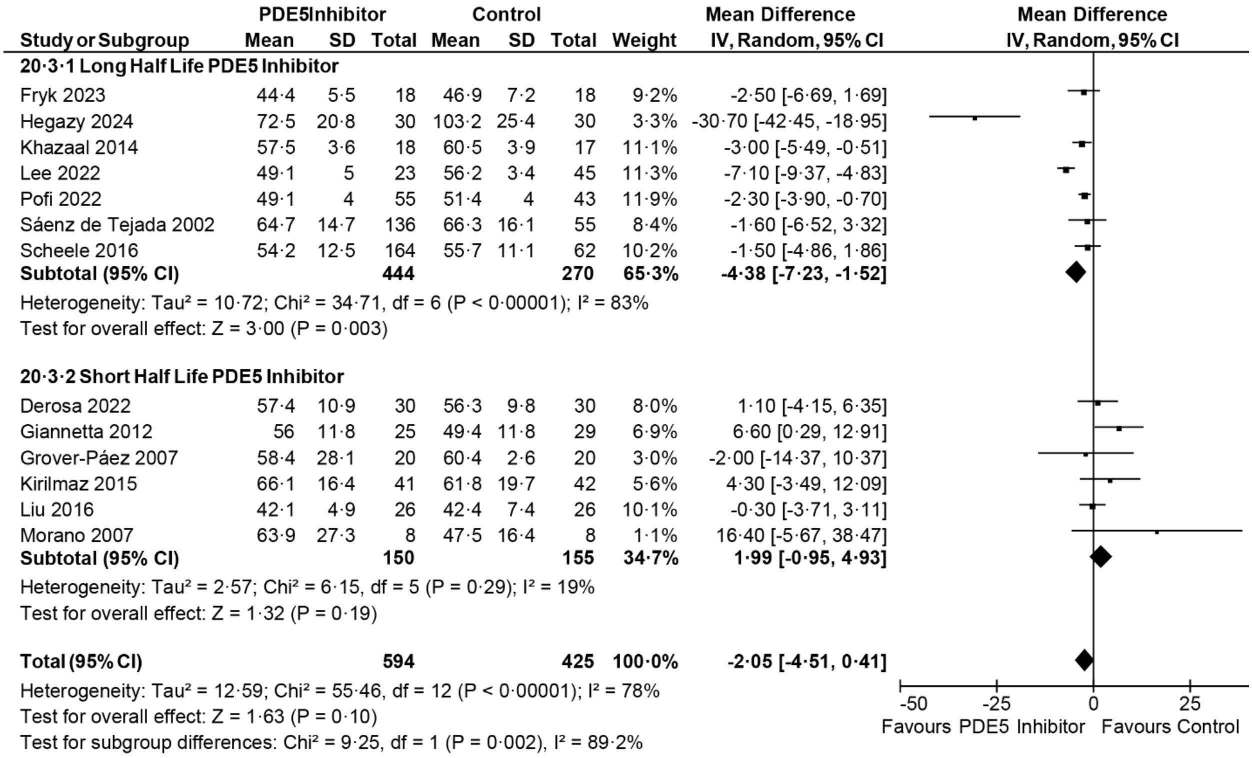

Supplementary Fig. 26: Secondary analysis on HOMA-IR

Secondary analysis on HOMA-IR among trials reporting HbA1c was conducted to investigate the effects of other metabolic parameters. The following conditions were used for each meta-analysis: a) long half-life PDE5 inhibitor; b) short half-life PDE5 inhibitor.

a)

	PDE5Inhibitor			Control				Mean Difference
**Study or Subgroup**	**Mean**	**SD**	**Total**	**Mean**	**SD**	**Total**	**Weight**	**IV, Random, 95% CI**
Fryk 2023	5·26	2·43	18	5·82	2·29	18	28·5%	-0·56 [-2·10, 0·98]
Lee 2022	2·54	3·32	23	3·24	2·51	45	28·5%	-0·70 [-2·24, 0·84]
Pofi 2022	4·85	3·85	55	4·44	2·45	43	43·1%	0·41 [-0·84, 1·66]
**Total (95% CI)**			**96**			**106**	**100·0%**	**-0·18 [-1·00, 0·64]**

Heterogeneity: Tau² = 0·00; Chi² = 1·52, df = 2 (P = 0·47); I² = 0%

Test for overall effect: Z = 0·43 (P = 0·66)

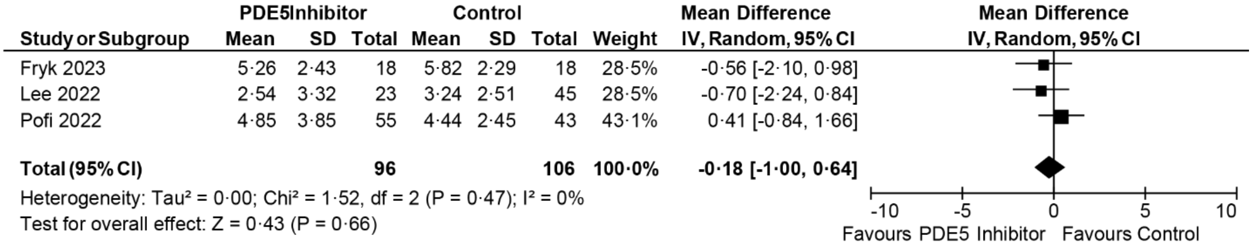

b)

	PDE5Inhibitor			Control				Mean Difference
**Study or Subgroup**	**Mean**	**SD**	**Total**	**Mean**	**SD**	**Total**	**Weight**	**IV, Random, 95% CI**
Derosa 2022	5·3	1·2	30	5·3	1·2	30	62·8%	0·00 [-0·61, 0·61]
Giannetta 2012	7·58	7·72	25	4·44	2·57	29	37·2%	3·14 [-0·03, 6·31]
**Total (95% CI)**			**55**			**59**	**100·0%**	**1·17 [-1·81, 4·14]**

Heterogeneity: Tau² = 3·58; Chi² = 3·64, df = 1 (P = 0·06); I² = 73%

Test for overall effect: Z = 0·77 (P = 0·44)

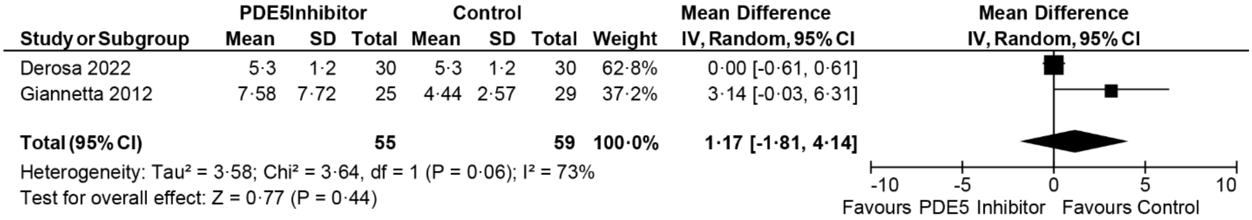

Supplementary Fig. 27: Secondary analysis on BMI

Secondary analysis on BMI among trials reporting HbA1c was conducted to investigate the effects of other metabolic parameters. Units are in kg/m^2. The following conditions were used for each meta-analysis: a) long half-life PDE5 inhibitor; b) short half-life PDE5 inhibitor.

a)

	PDE5 Inhibitor			Control				Mean Difference
**Study or Subgroup**	**Mean**	**SD**	**Total**	**Mean**	**SD**	**Total**	**Weight**	**IV, Random, 95% CI**
Fryk 2023	31·5	3·92	18	31·3	3·92	18	20·0%	0·20 [-2·36, 2·76]
Khazaal 2014	32·46	2·08	18	33·81	1·91	17	29·4%	-1·35 [-2·67, -0·03]
Lee 2022	25·8	3·05	23	27·64	3·69	45	26·8%	-1·84 [-3·49, -0·19]
Pofi 2022	29·24	5·54	55	27·05	4·77	43	23·8%	2·19 [0·15, 4·23]
**Total (95% CI)**			**114**			**123**	**100·0%**	**-0·33 [-2·07, 1·41]**

Heterogeneity: Tau² = 2·23; Chi² = 10·92, df = 3 (P = 0·01); I² = 73%

Test for overall effect: Z = 0·37 (P = 0·71)

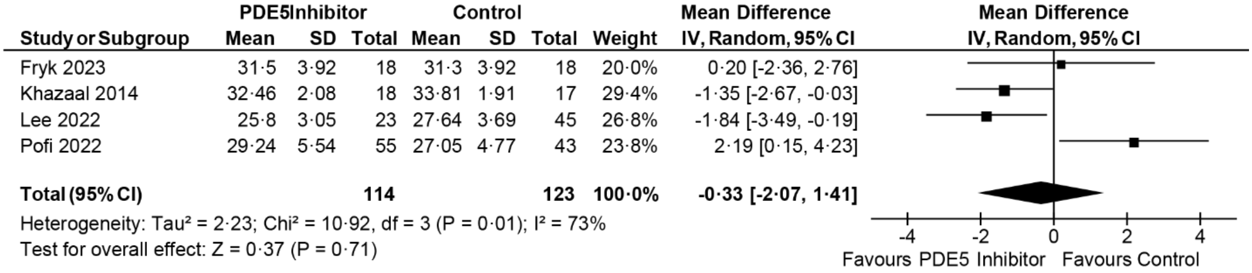

b)

	PDE5 Inhibitor			Control				Mean Difference
**Study or Subgroup**	**Mean**	**SD**	**Total**	**Mean**	**SD**	**Total**	**Weight**	**IV, Random, 95% CI**
Derosa 2022	28·5	2·3	30	28·2	2·1	30	83·5%	0·30 [-0·81, 1·41]
Giannetta 2012	28·31	4·94	25	27·32	4·38	29	16·5%	0·99 [-1·52, 3·50]
**Total (95% CI)**			**55**			**59**	**100·0%**	**0·41 [-0·60, 1·43]**

Heterogeneity: Tau² = 0·00; Chi² = 0·24, df = 1 (P = 0·62); I² = 0%

Test for overall effect: Z = 0·80 (P = 0·43)

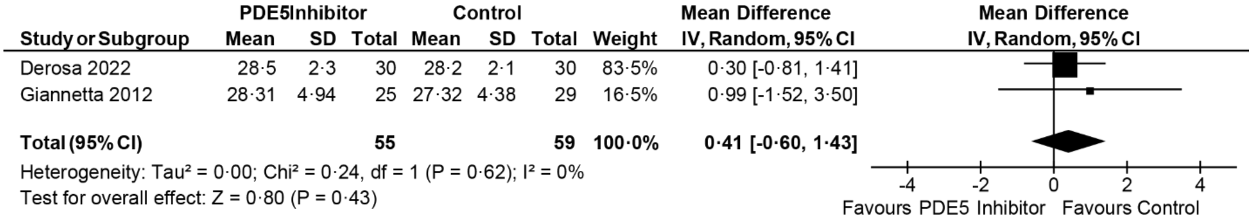

Supplementary Fig. 28: Secondary analysis on fasting glucose

Secondary analysis on fasting glucose among trials reporting HbA1c was conducted to investigate the effects of other metabolic parameters. Units are in mg/dL. The following conditions were used for each meta-analysis: a) long half-life PDE5 inhibitor; b) short half-life PDE5 inhibitor.

a)

	PDE5Inhibitor			Control				Mean Difference
**Study or Subgroup**	**Mean**	**SD**	**Total**	**Mean**	**SD**	**Total**	**Weight**	**IV, Random, 95% CI**
Fryk 2023	132·66	24·61	18	137·88	24·61	18	27·5%	-5·22 [-21·30, 10·86]
Hegazy 2024	171·65	67·3	30	238·5	97·13	30	10·4%	-66·85 [-109·13, -24·57]
Khazaal 2014	142·47	18·97	18	135·83	20·08	17	30·4%	6·64 [-6·32, 19·60]
Lee 2022	121·62	24·95	23	126·05	18·34	45	31·7%	-4·43 [-15·95, 7·09]
**Total (95% CI)**			**89**			**110**	**100·0%**	**-7·76 [-23·75, 8·22]**

Heterogeneity: Tau² = 175·09; Chi² = 11·02, df = 3 (P = 0·01); I² = 73%
Test for overall effect: Z = 0·95 (P = 0·34)

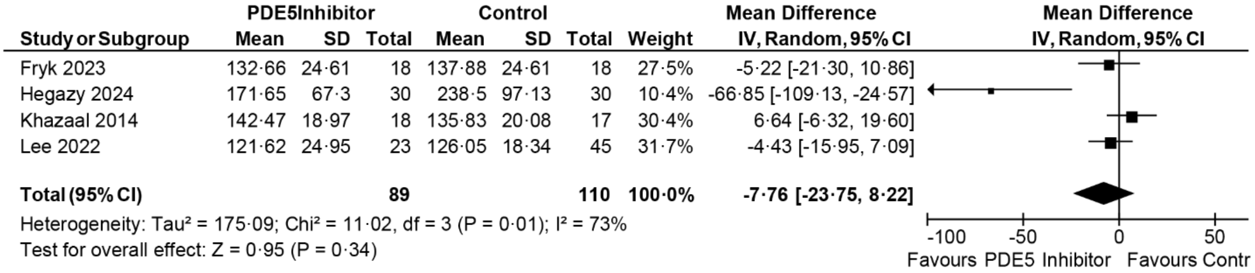

b)

	PDE5Inhibitor			Control				Mean Difference
**Study or Subgroup**	**Mean**	**SD**	**Total**	**Mean**	**SD**	**Total**	**Weight**	**IV, Random, 95% CI**
Derosa 2022	139·2	18·5	30	141·1	19·4	30	45·4%	-1·90 [-11·49, 7·69]
Liu 2016	102·6	55·71	26	108	46·79	26	28·2%	-5·40 [-33·36, 22·56]
Morano 2007	165·4	38·6	8	127·8	19·5	8	26·5%	37·60 [7·63, 67·57]
**Total (95% CI)**			**64**			**64**	**100·0%**	**7·57 [-15·05, 30·19]**

Heterogeneity: Tau² = 269·50; Chi² = 6·28, df = 2 (P = 0·04); I² = 68%
Test for overall effect: Z = 0·66 (P = 0·51)

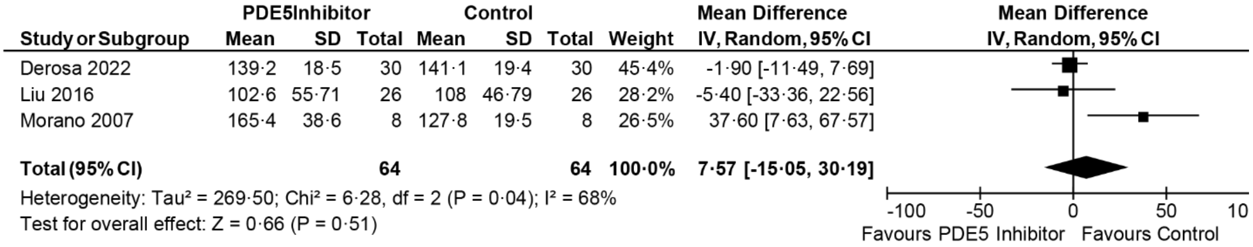

Supplementary Fig. 29: Secondary analysis on 2 hour post prandial glucose

Secondary analysis on 2 hour post prandial glucose among trials reporting HbA1c was conducted to investigate the effects of other metabolic parameters. Units are in mg/dL. The following conditions were used for each meta-analysis: a) long half-life PDE5 inhibitor; b) short half-life PDE5 inhibitor. No trials with short half-life PDE5 inhibitors reported 2 hour post prandial glucose, but the forest plot is included for consistency.

a)

	PDE5Inhibitor			Control				Mean Difference
**Study or Subgroup**	**Mean**	**SD**	**Total**	**Mean**	**SD**	**Total**	**Weight**	**IV, Random, 95% CI**
Hegazy 2024	245·63	98·23	30	367·36	100·41	30	100·0%	-121·73 [-171·99, -71·47]
**Total (95% CI)**			**30**			**30**	**100·0%**	**-121·73 [-171·99, -71·47]**

Heterogeneity: Not applicable

Test for overall effect: Z = 4·75 (P < 0·00001)

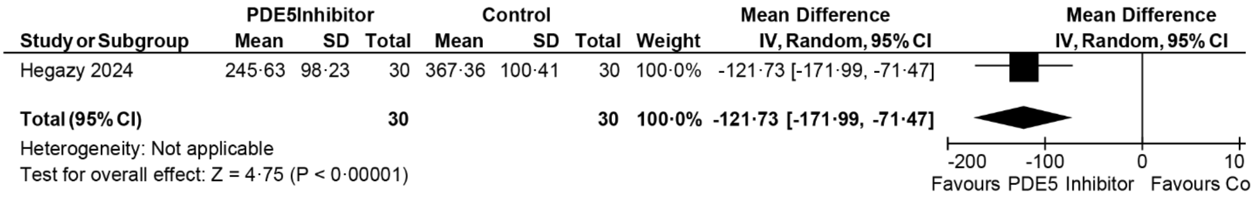

b)

	PDE5Inhibitor			Control				Mean Difference
**Study or Subgroup**	**Mean**	**SD**	**Total**	**Mean**	**SD**	**Total**	**Weight**	**IV, Random, 95% CI**
**Total (95% CI)**			**0**			**0**		**Not estimable**

Heterogeneity: Not applicable

Test for overall effect: Not applicable

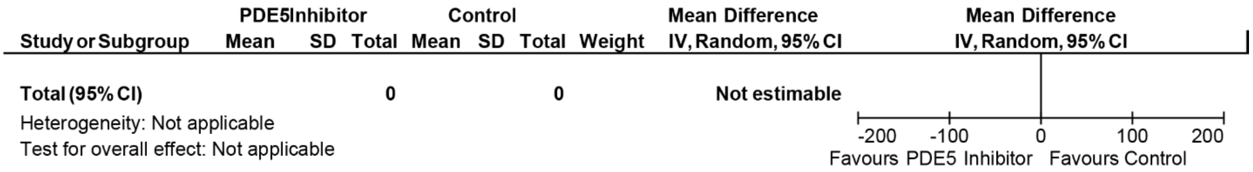

Supplementary Fig. 30: Secondary analysis on fasting insulin

Secondary analysis on fasting insulin among trials reporting HbA1c was conducted to investigate the effects of other metabolic parameters. Units are in μU/mL. The following conditions were used for each meta-analysis: a) long half-life PDE5 inhibitor; b) short half-life PDE5 inhibitor.

a)

	PDE5Inhibitor			Control				Mean Difference
**Study or Subgroup**	**Mean**	**SD**	**Total**	**Mean**	**SD**	**Total**	**Weight**	**IV, Random, 95% CI**
Fryk 2023	16·5	6·64	18	17·2	6·13	18	53·9%	-0·70 [-4·87, 3·47]
Lee 2022	8·35	9·14	23	10·71	8·66	45	46·1%	-2·36 [-6·87, 2·15]
**Total (95% CI)**			**41**			**63**	**100·0%**	**-1·47 [-4·53, 1·60]**

Heterogeneity: Tau² = 0·00; Chi² = 0·28, df = 1 (P = 0·60); I² = 0%

Test for overall effect: Z = 0·94 (P = 0·35)

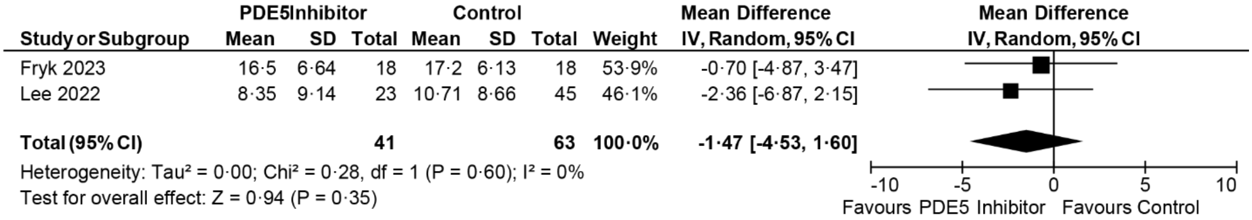

b)

	PDE5Inhibitor			Control				Mean Difference
**Study or Subgroup**	**Mean**	**SD**	**Total**	**Mean**	**SD**	**Total**	**Weight**	**IV, Random, 95% CI**
Derosa 2022	15·3	3·8	30	15·2	3·7	30	100·0%	0·10 [-1·80, 2·00]
**Total (95% CI)**			**30**			**30**	**100·0%**	**0·10 [-1·80, 2·00]**

Heterogeneity: Not applicable

Test for overall effect: Z = 0·10 (P = 0·92)

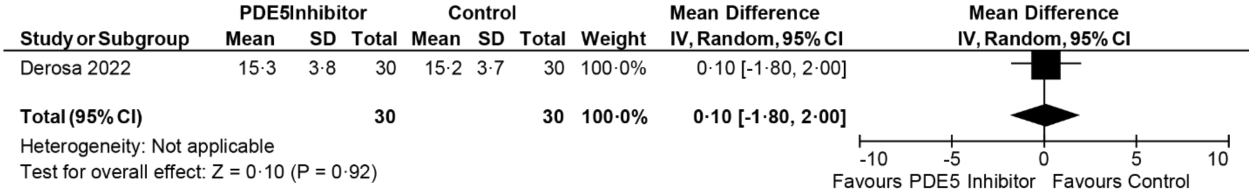

Supplementary Fig. 31: Secondary analysis on cholesterol

Secondary analysis on cholesterol among trials reporting HbA1c was conducted to investigate the effects of other metabolic parameters. Units are in mg/dL. The following conditions were used for each meta-analysis: a) long half-life PDE5 inhibitor; b) short half-life PDE5 inhibitor.

a)

	PDE5Inhibitor			Control				Mean Difference
**Study or Subgroup**	**Mean**	**SD**	**Total**	**Mean**	**SD**	**Total**	**Weight**	**IV, Random, 95% CI**
Fryk 2023	186·78	45·49	18	186·39	47·74	18	11·6%	0·39 [-30·07, 30·85]
Khazaal 2014	198·47	19·15	18	205·28	29·41	17	39·3%	-6·81 [-23·35, 9·73]
Lee 2022	135·84	28·05	23	142·26	31·98	45	49·1%	-6·42 [-21·21, 8·37]
**Total (95% CI)**			**59**			**80**	**100·0%**	**-5·78 [-16·15, 4·58]**

Heterogeneity: Tau² = 0·00; Chi² = 0·18, df = 2 (P = 0·91); I² = 0%
Test for overall effect: Z = 1·09 (P = 0·27)

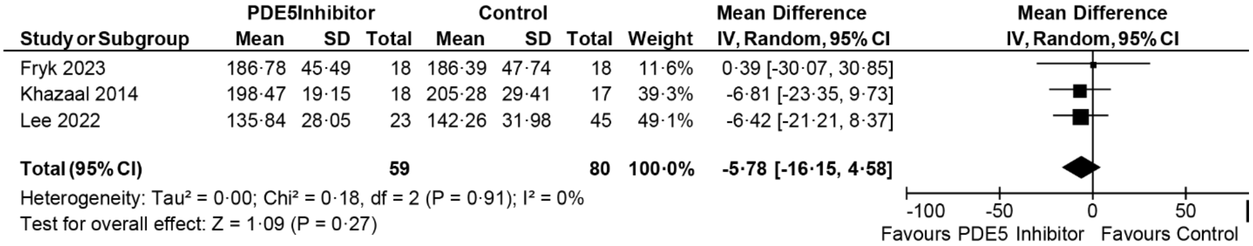

b)

	PDE5Inhibitor			Control				Mean Difference
**Study or Subgroup**	**Mean**	**SD**	**Total**	**Mean**	**SD**	**Total**	**Weight**	**IV, Random, 95% CI**
Derosa 2022	173·1	32·8	30	175·4	33·1	30	94·6%	-2·30 [-18·97, 14·37]
Morano 2007	198·6	81·5	8	205·4	58·4	8	5·4%	-6·80 [-76·28, 62·68]
**Total (95% CI)**			**38**			**38**	**100·0%**	**-2·55 [-18·76, 13·67]**

Heterogeneity: Tau² = 0·00; Chi² = 0·02, df = 1 (P = 0·90); I² = 0%
Test for overall effect: Z = 0·31 (P = 0·76)

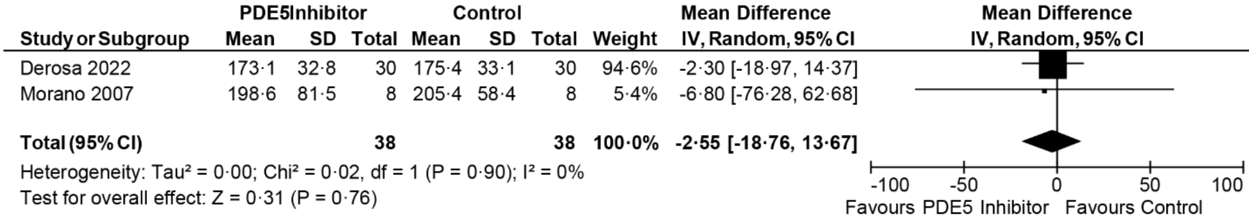

Supplementary Fig. 32: Secondary analysis on triglycerides

Secondary analysis on triglycerides among trials reporting HbA1c was conducted to investigate the effects of other metabolic parameters. Units are in mg/dL. The following conditions were used for each meta-analysis: a) long half-life PDE5 inhibitor; b) short half-life PDE5 inhibitor.

a)

	PDE5Inhibitor			Control				Mean Difference
**Study or Subgroup**	**Mean**	**SD**	**Total**	**Mean**	**SD**	**Total**	**Weight**	**IV, Random, 95% CI**
Fryk 2023	146·14	12·47	18	162·97	68·57	18	25·0%	-16·83 [-49·03, 15·37]
Hegazy 2024	215·03	85·08	30	268·68	52·07	30	22·7%	-53·65 [-89·34, -17·96]
Khazaal 2014	169·74	23·35	18	170·61	21·66	17	38·1%	-0·87 [-15·78, 14·04]
Lee 2022	127·58	123·55	23	137·48	54·57	45	14·3%	-9·90 [-62·85, 43·05]
**Total (95% CI)**			**89**			**110**	**100·0%**	**-18·11 [-42·39, 6·17]**

Heterogeneity: Tau² = 345·09; Chi² = 7·36, df = 3 (P = 0·06); I² = 59%

Test for overall effect: Z = 1·46 (P = 0·14)

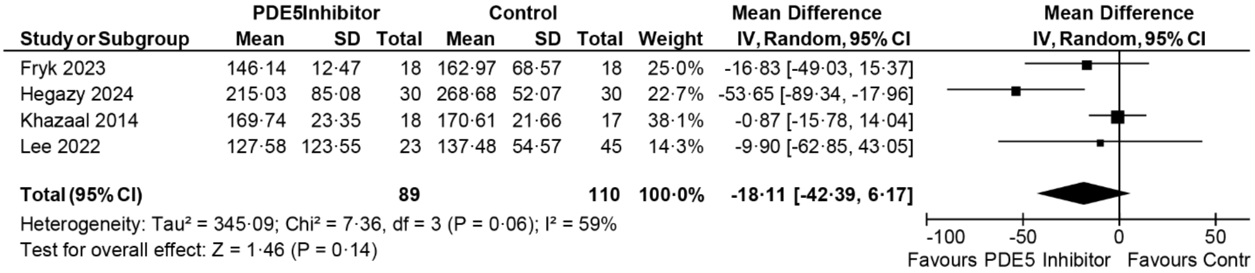

b)

	PDE5Inhibitor			Control				Mean Difference
**Study or Subgroup**	**Mean**	**SD**	**Total**	**Mean**	**SD**	**Total**	**Weight**	**IV, Random, 95% CI**
Derosa 2022	135·3	22·1	30	128·4	21·7	30	85·6%	6·90 [-4·18, 17·98]
Giannetta 2012	134·63	64·66	25	110·71	42·51	29	11·9%	23·92 [-5·78, 53·62]
Morano 2007	121·6	42·1	8	139·2	83·5	8	2·5%	-17·60 [-82·40, 47·20]
**Total (95% CI)**			**63**			**67**	**100·0%**	**8·32 [-1·94, 18·57]**

Heterogeneity: Tau² = 0·00; Chi² = 1·74, df = 2 (P = 0·42); I² = 0%

Test for overall effect: Z = 1·59 (P = 0·11)

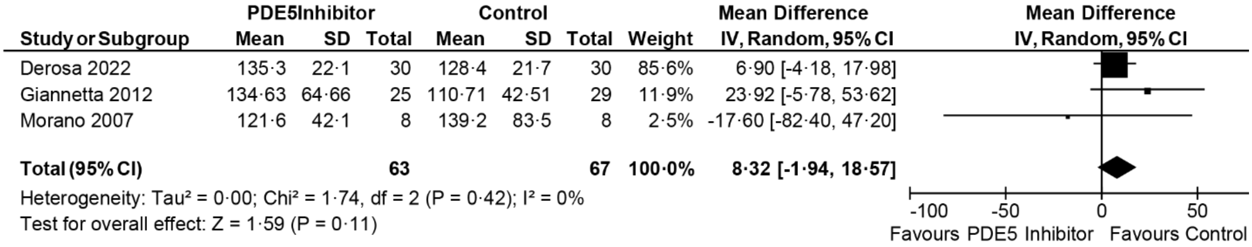

Supplementary Fig. 33: Secondary analysis on HDL

Secondary analysis on HDL among trials reporting HbA1c was conducted to investigate the effects of other metabolic parameters. Units are in mg/dL. The following conditions were used for each meta-analysis: a) long half-life PDE5 inhibitor; b) short half-life PDE5 inhibitor.

a)

	PDE5Inhibitor			Control				Mean Difference
**Study or Subgroup**	**Mean**	**SD**	**Total**	**Mean**	**SD**	**Total**	**Weight**	**IV, Random, 95% CI**
Fryk 2023	45·63	12·05	18	45·63	11·66	18	23·3%	0·00 [-7·75, 7·75]
Hegazy 2024	56	16·34	30	29·46	6·3	30	24·4%	26·54 [20·27, 32·81]
Khazaal 2014	40·35	3·7	18	39·16	3·05	17	26·6%	1·19 [-1·05, 3·43]
Lee 2022	44·66	8·13	23	44·87	9·5	45	25·7%	-0·21 [-4·54, 4·12]
**Total (95% CI)**			**89**			**110**	**100·0%**	**6·75 [-3·41, 16·91]**

Heterogeneity: Tau² = 99·73; Chi² = 59·39, df = 3 (P < 0·00001); I² = 95%

Test for overall effect: Z = 1·30 (P = 0·19)

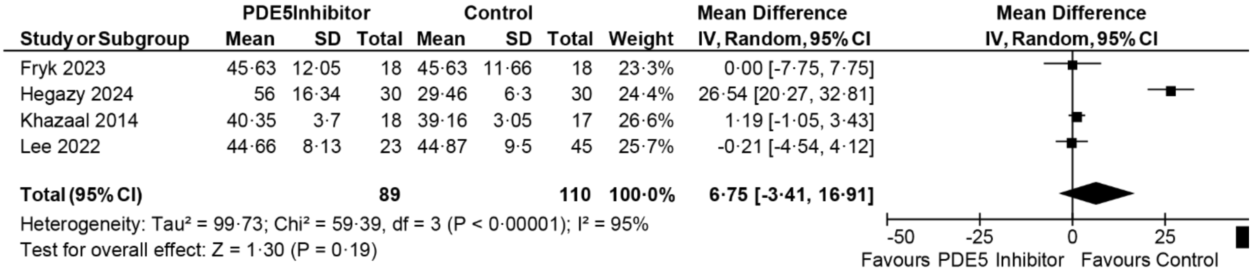

b)

	PDE5Inhibitor			Control				Mean Difference
**Study or Subgroup**	**Mean**	**SD**	**Total**	**Mean**	**SD**	**Total**	**Weight**	**IV, Random, 95% CI**
Derosa 2022	40·6	5·5	30	41·4	6·2	30	34·3%	-0·80 [-3·77, 2·17]
Giannetta 2012	43·31	8·51	25	46·4	11·99	29	31·5%	-3·09 [-8·58, 2·40]
Morano 2007	42·5	3·9	8	56·5	2·1	8	34·2%	-14·00 [-17·07, -10·93]
**Total (95% CI)**			**63**			**67**	**100·0%**	**-6·03 [-15·25, 3·18]**

Heterogeneity: Tau² = 62·25; Chi² = 38·59, df = 2 (P < 0·00001); I² = 95%

Test for overall effect: Z = 1·28 (P = 0·20)

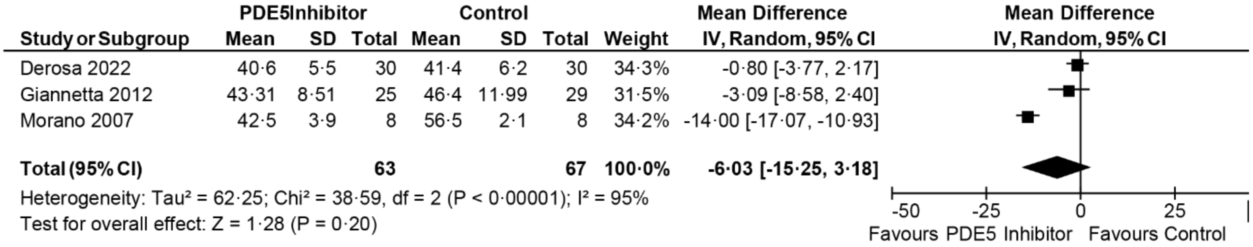

Supplementary Fig. 34: Secondary analysis on LDL

Secondary analysis on LDL among trials reporting HbA1c was conducted to investigate the effects of other metabolic parameters. Units are in mg/dL. The following conditions were used for each meta-analysis: a) long half-life PDE5 inhibitor; b) short half-life PDE5 inhibitor.

a)

	PDE5Inhibitor			Control				Mean Difference
**Study or Subgroup**	**Mean**	**SD**	**Total**	**Mean**	**SD**	**Total**	**Weight**	**IV, Random, 95% CI**
Fryk 2023	45·63	12·05	18	45·63	11·66	18	30·1%	0·00 [-7·75, 7·75]
Hegazy 2024	164·88	46·9	30	204·43	26·95	30	20·5%	-39·55 [-58·91, -20·19]
Khazaal 2014	124·35	21·08	18	132·44	28·01	17	22·8%	-8·09 [-24·59, 8·41]
Lee 2022	74·22	23·32	23	78·35	26·29	45	26·6%	-4·13 [-16·37, 8·11]
**Total (95% CI)**			**89**			**110**	**100·0%**	**-11·04 [-25·19, 3·12]**

Heterogeneity: Tau² = 157·46; Chi² = 13·97, df = 3 (P = 0·003); I² = 79%

Test for overall effect: Z = 1·53 (P = 0·13)

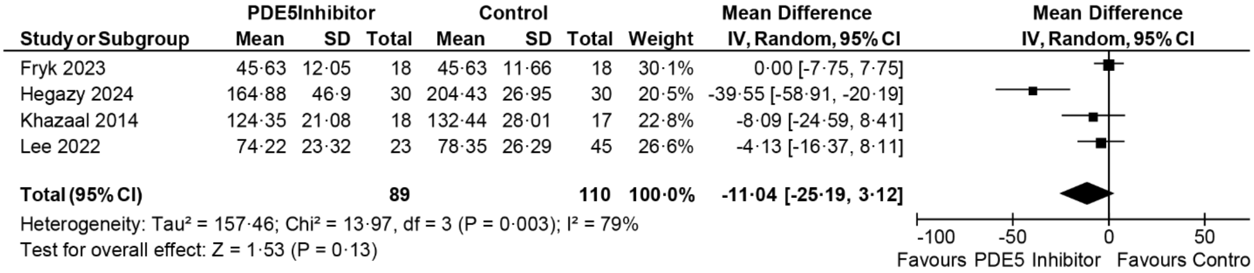

b)

	PDE5Inhibitor			Control				Mean Difference
**Study or Subgroup**	**Mean**	**SD**	**Total**	**Mean**	**SD**	**Total**	**Weight**	**IV, Random, 95% CI**
Derosa 2022	106	14·3	30	108·4	15·5	30	96·1%	-2·40 [-9·95, 5·15]
Morano 2007	120·9	36·6	8	120·6	39·6	8	3·9%	0·30 [-37·07, 37·67]
**Total (95% CI)**			**38**			**38**	**100·0%**	**-2·29 [-9·69, 5·10]**

Heterogeneity: Tau² = 0·00; Chi² = 0·02, df = 1 (P = 0·89); I² = 0%

Test for overall effect: Z = 0·61 (P = 0·54)

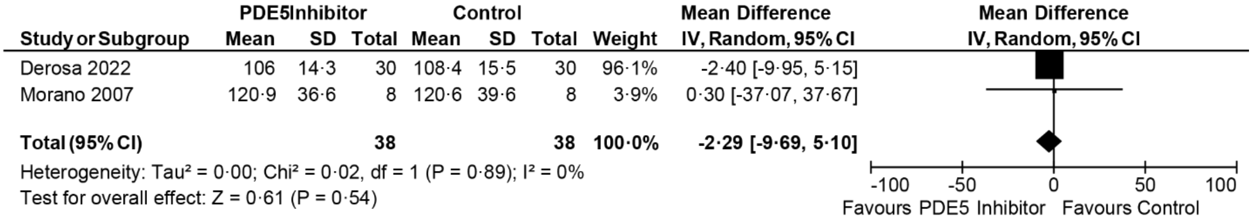

Supplementary Fig. 35: Funnel plots

While the small sample size of less than 10 studies prevents meaningful interpretation of funnel plots, these are included for reference for future meta-analyses. Funnel plots appear mostly symmetrical, with many data points close to the mean line. Funnel plots were generated for a) all long half-life PDE5 inhibitor trials b) all short half-life PDE5 inhibitor trials c) long half-life PDE5 inhibitor trials among participants with type 2 diabetes and of duration of at least 8 weeks d) short half-life PDE5 inhibitor trials among participants with type 2 diabetes and of duration of at least 8 weeks.

a)

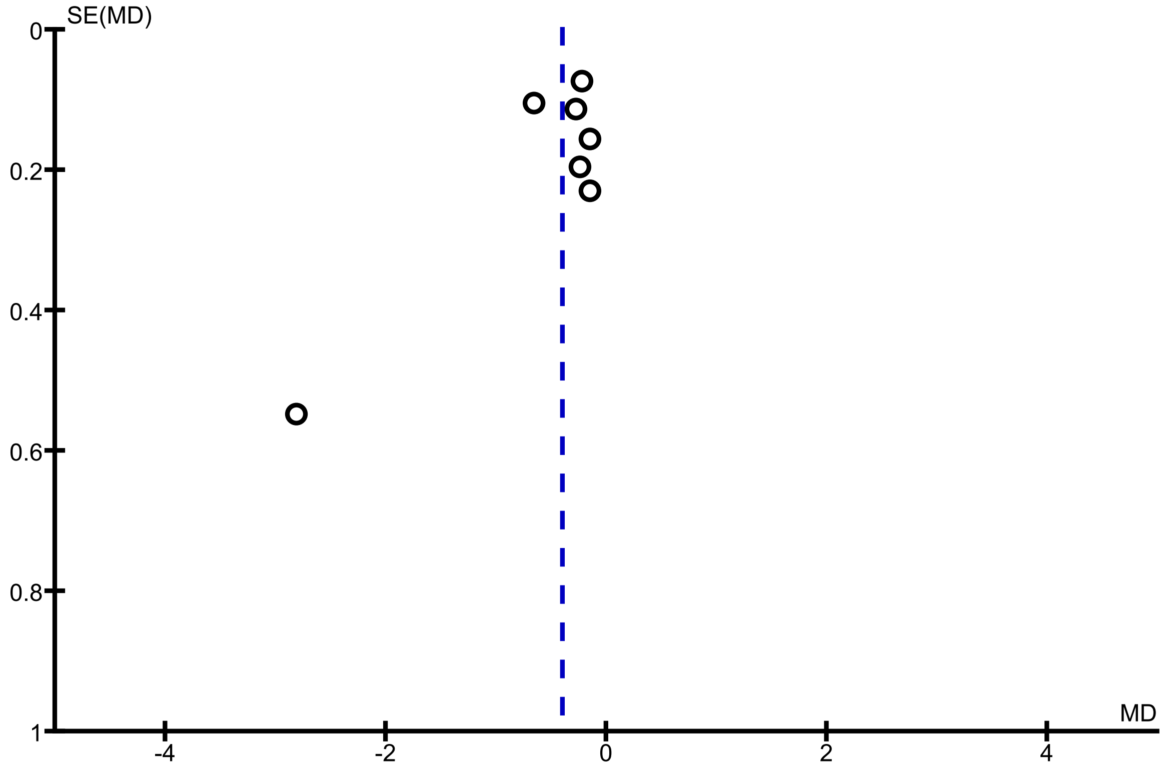

b)

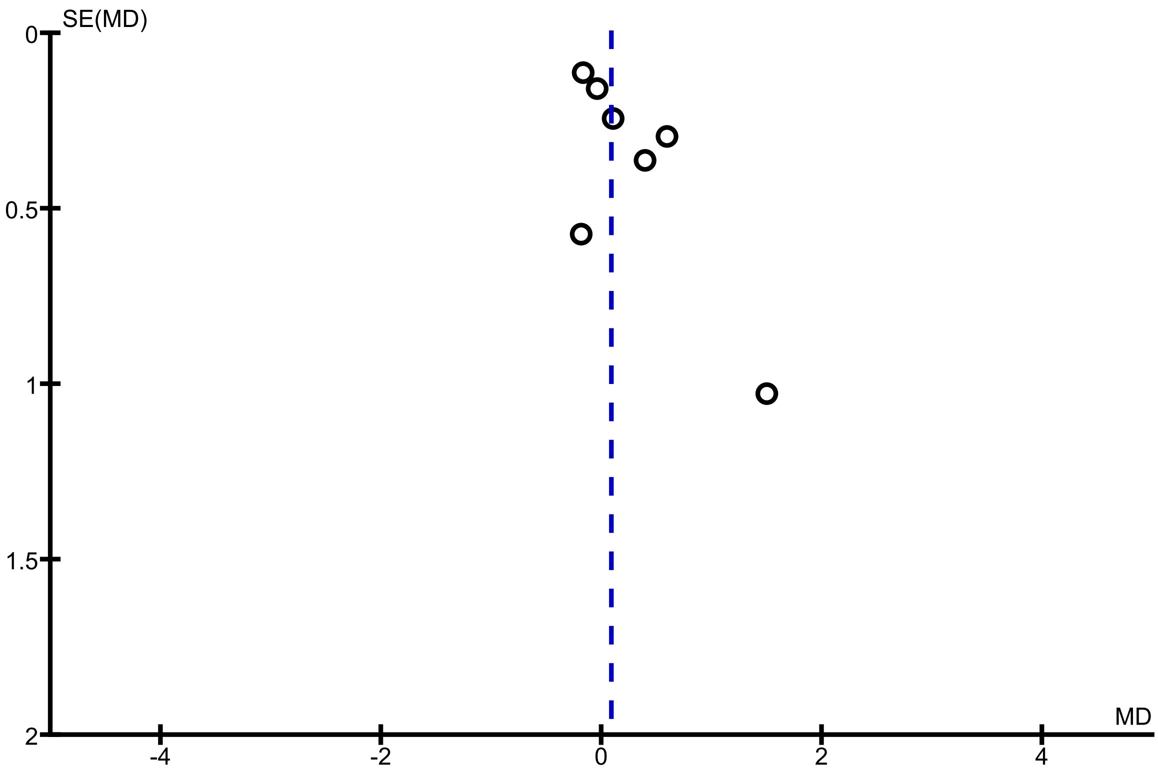

c)

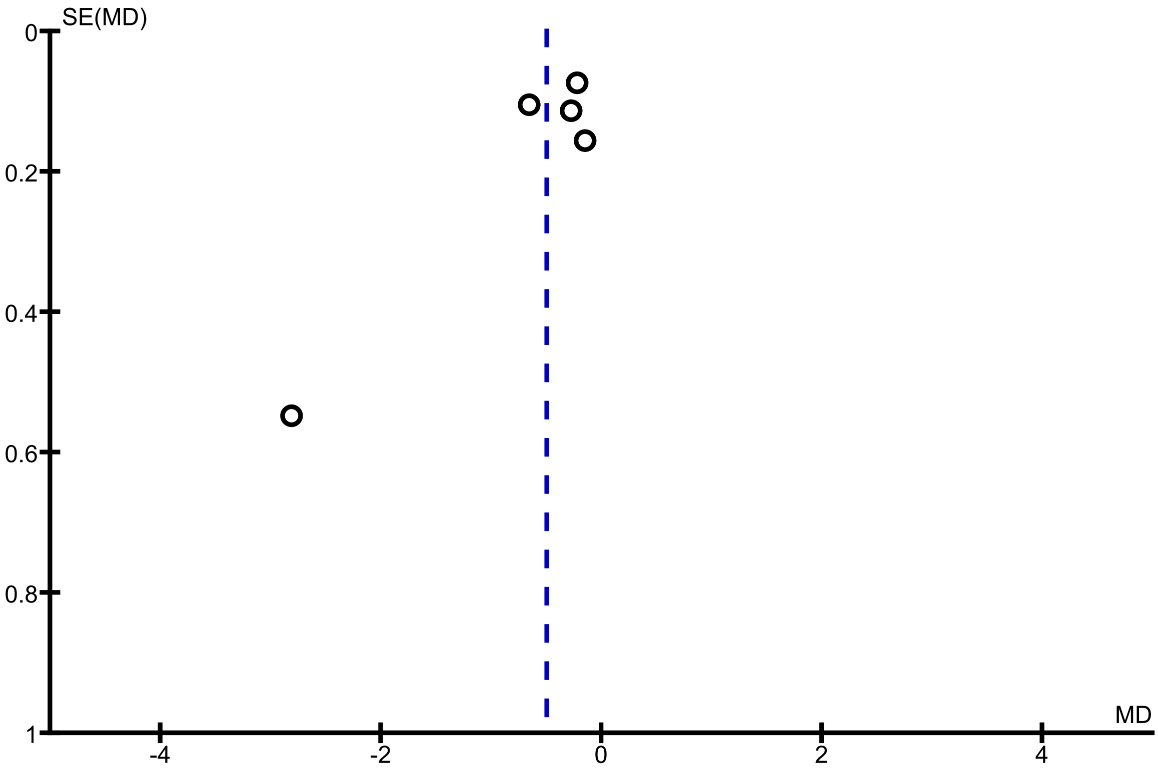

d)

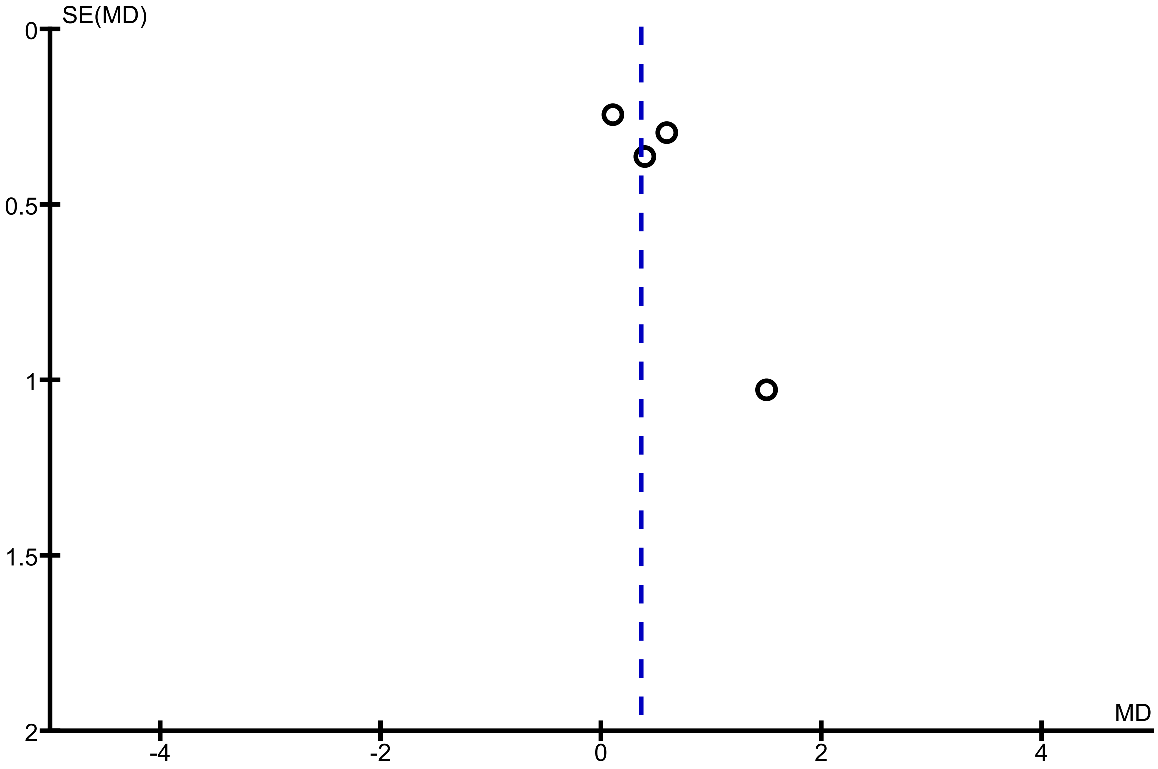

Supplementary Fig. 36: Risk of Bias Analysis Overview

Study	D1	D2	D3	D4	D5	Overall
Hegazy et al. (2024)	+	+	+	+	+	+
Fryk et al. (2023)	+	+	-	+	+	-
Pofi et al. (2022)	+	+	+	+	+	+
Lee et al. (2022)	+	+	+	+	+	+
Khazaal (2014)	-	-	+	+	-	-
Sáenz de Tejada et al. (2002)	+	+	+	+	-	-
Liu et al. (2016)	+	+	+	+	+	+
Morano et al. (2007)	+	+	+	+	-	-
Grover-Páez et al. (2007)	+	+	+	+	-	-
Derosa et al. (2022)	+	-	+	+	+	-
Scheele et al. (2016)	+	-	+	+	+	-
Giannetta et al. (2012)	+	+	+	+	+	+
Kirilmaz et al. (2015)	+	+	-	+	-	-

Risk of bias domains

Domains :
D1: Bias due to randomisation.
D2: Bias due to deviations from intended intervention.
D3: Bias due to missing data.
D4: Bias due to outcome measurement.
D5: Bias due to selection of reported result.

Judgement
+ Low
- Some concerns

Supplementary Fig. 37: Risk of Bias Baseline Characteristics Analysis

As outlined in Domain 1.3 of the Risk of Bias guidelines, baseline differences were analyzed and compared to reasonable baseline differences.[6] In statistical analyses, p-values are roughly uniformly distributed from 0 to 1, regardless of p-value measurement methodology. Consequently, it is also expected that the empirical cumulative distribution function (represented in red) coarsely follows a linear increase from 0 to 1 (indicated in blue) as the p-value upper limit ranges from 0 to 1. That is, for instance, it is expected that approximately 30% of p-values are less than 0.3. All clinical trials were analyzed in this aspect: **a)** Hegazy 2024 **b)** Fryk 2023 **c)** Pofi 2022 **d)** Lee 2022 **e)** Derosa 2022 **f)** Liu 2016 **g)** Scheele 2016 **h)** Kirilmaz 2015 **i)** Khazaal 2014 **j)** Giannetta 2012 **k)** Morano 2007 **l)** Grover-Páez 2007 **m)** Sáenz de Tejada 2002.

a)

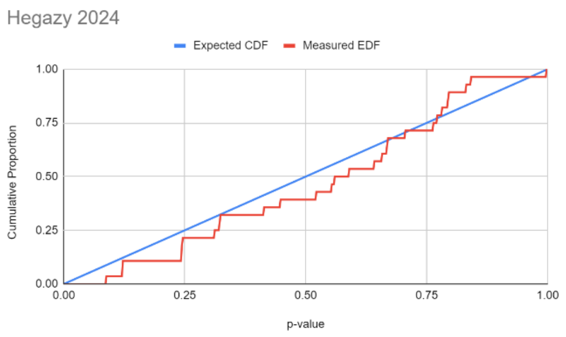

b)

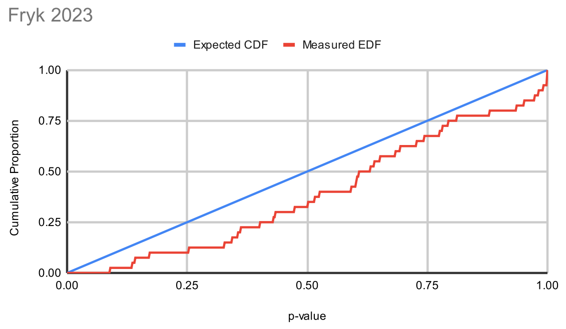

c)

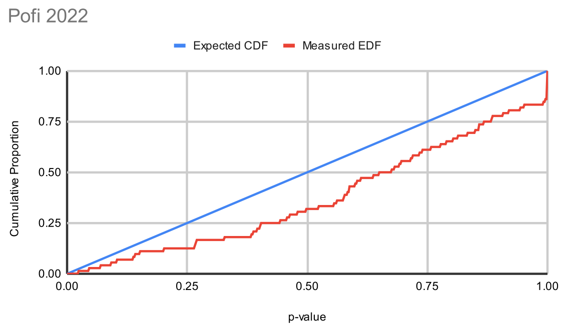

d)

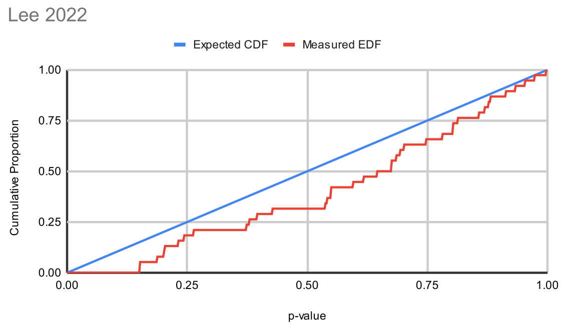
Lee 2022
Expected CDF
Measured EDF
Cumulative Proportion
p-value
0.00
0.25
0.50
0.75
1.00

e)

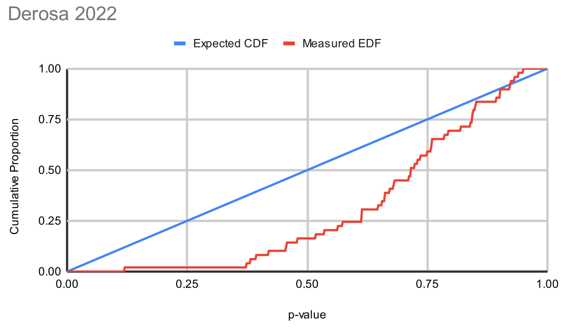
Derosa 2022
Expected CDF
Measured EDF
Cumulative Proportion
p-value
0.00
0.25
0.50
0.75
1.00

f)

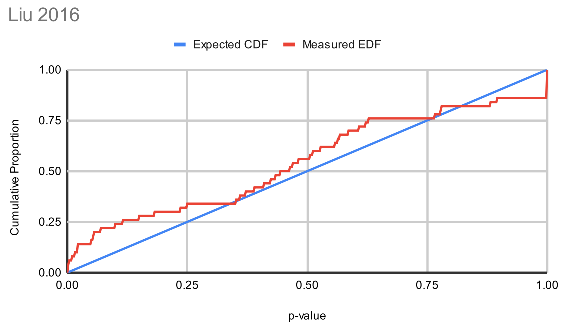
Liu 2016
Expected CDF
Measured EDF
Cumulative Proportion
p-value
0.00
0.25
0.50
0.75
1.00

g)

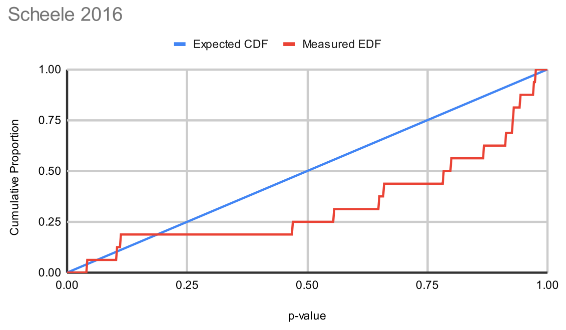
Scheele 2016
Expected CDF
Measured EDF
Cumulative Proportion
p-value
0.00
0.25
0.50
0.75
1.00

h)

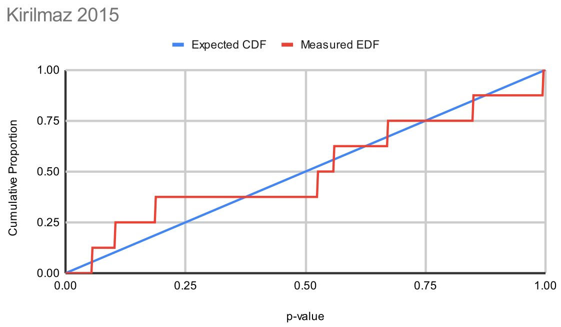
Kirilmaz 2015
Expected CDF
Measured EDF
Cumulative Proportion
1.00
0.75
0.50
0.25
0.00
0.00
0.25
0.50
0.75
1.00
p-value

i)

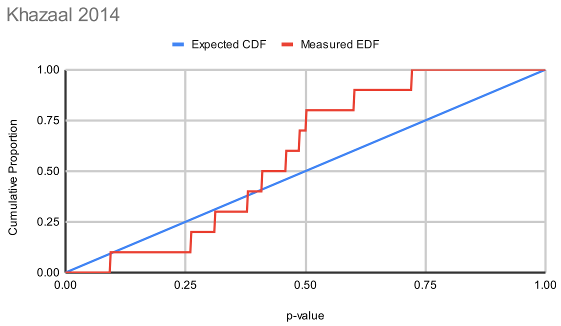
Khazaal 2014
Expected CDF
Measured EDF
Cumulative Proportion
1.00
0.75
0.50
0.25
0.00
0.00
0.25
0.50
0.75
1.00
p-value

j)

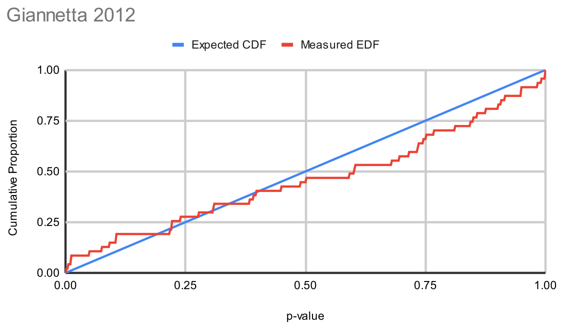
Giannetta 2012
Expected CDF
Measured EDF
Cumulative Proportion
1.00
0.75
0.50
0.25
0.00
0.00
0.25
0.50
0.75
1.00
p-value

k)

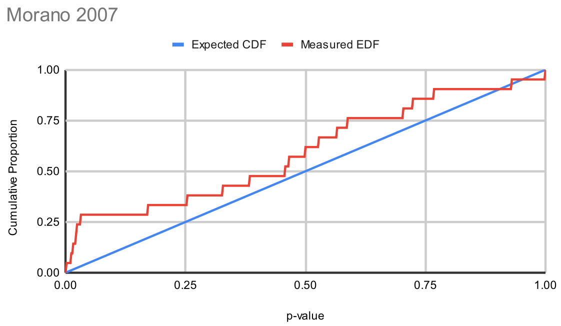
Morano 2007
Expected CDF
Measured EDF
Cumulative Proportion
1.00
0.75
0.50
0.25
0.00
0.00
0.25
0.50
0.75
1.00
p-value

l)

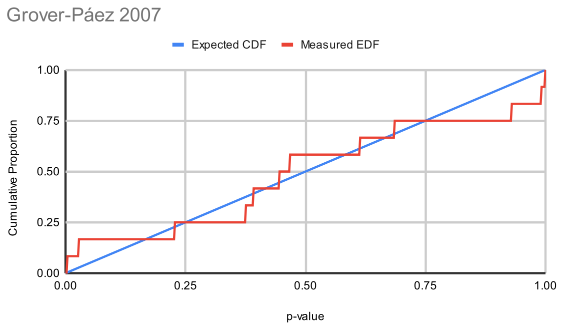
Grover-Páez 2007
Expected CDF
Measured EDF
Cumulative Proportion
1.00
0.75
0.50
0.25
0.00
0.00
0.25
0.50
0.75
1.00
p-value

m)

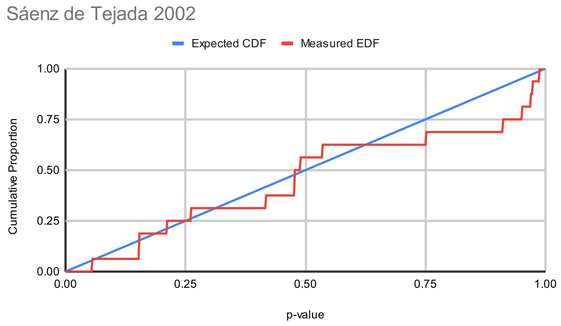
Sáenz de Tejada 2002
Expected CDF
Measured EDF
Cumulative Proportion
1.00
0.75
0.50
0.25
0.00
0.00
0.25
0.50
0.75
1.00
p-value

Supplementary Fig. 38: Risk of Bias Analysis (Hegazy 2024)

Study details

**Reference**	Hegazy 2024[7]

Study design

X Individually-randomized parallel-group trial
☐ Cluster-randomized parallel-group trial
☐ Individually randomized cross-over (or other matched) trial

For the purposes of this assessment, the interventions being compared are defined as

Experimental: Tadalafil treatment Comparator: Control treatment

**Specify which outcome is being assessed for risk of bias**	HbA1c% at baseline and at 6 months
**Specify the numerical result being assessed.** In case of multiple alternative analyses being presented, specify the numeric result (e.g. RR = 1.52 (95% CI 0.83 to 2.77) and/or a reference (e.g. to a table, figure or paragraph) that uniquely defines the result being assessed.	Table 2

Is the review team's aim for this result…?

X to assess the effect of *assignment to intervention* (the 'intention-to-treat' effect)
☐ to assess the effect of *adhering to intervention* (the 'per-protocol' effect)

If the aim is to assess the effect of *adhering to intervention*, select the deviations from intended intervention that should be addressed (at least one must be checked):

☐ occurrence of non-protocol interventions
☐ failures in implementing the intervention that could have affected the outcome
☐ non-adherence to their assigned intervention by trial participants

Which of the following sources were <u>obtained</u> to help inform the risk-of-bias assessment? (tick as many as apply)

X Journal article(s) with results of the trial
X Trial protocol
☐ Statistical analysis plan (SAP)
☐ Non-commercial trial registry record (e.g. ClinicalTrials.gov record)
☐ Company-owned trial registry record (e.g. GSK Clinical Study Register record)

- ☐ “Grey literature” (e.g. unpublished thesis)
- ☐ Conference abstract(s) about the trial
- ☐ Regulatory document (e.g. Clinical Study Report, Drug Approval Package)
- ☐ Research ethics application
- ☐ Grant database summary (e.g. NIH RePORTER or Research Councils UK Gateway to Research)
- ☐ Personal communication with trialist
- ☐ Personal communication with the sponsor

Risk of bias assessment

Responses underlined in green are potential markers for low risk of bias, and responses in red are potential markers for a risk of bias. Where questions relate only to sign posts to other questions, no formatting is used.

Domain 1: Risk of bias arising from the randomization process

Signalling questions	Comments	Response options
**1.1 Was the allocation sequence random?**	"randomly divided into three groups with a ratio of 1:1:1 using computer-generated code into three groups"	Y
**1.2 Was the allocation sequence concealed until participants were enrolled and assigned to interventions?**		PY
**1.3 Did baseline differences between intervention groups suggest a problem with the randomization process?**	Baseline p-values appear uniformly distributed.	N
**Risk-of-bias judgement**		Low
Optional: What is the predicted direction of bias arising from the randomization process?		NA

Domain 2: Risk of bias due to deviations from the intended interventions (*effect of assignment to intervention*)

Signalling questions	Comments	Response options
**2.1. Were participants aware of their assigned intervention during the trial?**	The study was an "open-labeled" study.	Y
**2.2. Were carers and people delivering the interventions aware of participants' assigned intervention during the trial?**		Y
**2.3. If Y/PY/NI to 2.1 or 2.2: Were there deviations from the intended intervention that arose because of the trial context?**	Participants were not allowed to change their (medications, usual diet, or physical activity) during the study period."	N
**2.4 If Y/PY to 2.3: Were these deviations likely to have affected the outcome?**		NA
**2.5. If Y/PY/NI to 2.4: Were these deviations from intended intervention balanced between groups?**		NA
**2.6 Was an appropriate analysis used to estimate the effect of assignment to intervention?**	"All participants submitted to the following analysis at the baseline and 6 months after the assigned treatment" and all 30 participants assigned to each treatment group were analyzed (Figure 1), making this a full ITT analysis.	Y
**2.7 If N/PN/NI to 2.6: Was there potential for a substantial impact (on the result) of the failure to analyse participants in the group to which they were randomized?**		NA
**Risk-of-bias judgement**		Low
Optional: What is the predicted direction of bias due to deviations from intended interventions?		NA

Domain 3: Missing outcome data

Signalling questions	Comments	Response options
**3.1 Were data for this outcome available for all, or nearly all, participants randomized?**	As in Domain 2.6, "All participants submitted to the following analysis at the baseline and 6 months after the assigned treatment" and all 30 participants assigned to each treatment group were analyzed (Figure 1), making this a full ITT analysis.	Y
**3.2 If N/PN/NI to 3.1: Is there evidence that the result was not biased by missing outcome data?**		NA
**3.3 If N/PN to 3.2: Could missingness in the outcome depend on its true value?**		NA
**3.4 If Y/PY/NI to 3.3: Is it likely that missingness in the outcome depended on its true value?**		NA
**Risk-of-bias judgement**		Low
Optional: What is the predicted direction of bias due to missing outcome data?		NA

Domain 4: Risk of bias in measurement of the outcome

Signalling questions	Comments	Response options
**4.1 Was the method of measuring the outcome inappropriate?**	HbA1c was measured with standardized HPLC technique.	N
**4.2 Could measurement or ascertainment of the outcome have differed between intervention groups?**	No differences in A1c measurement were specified, and it is very likely that both received the same laboratory measurement methods.	PN
**4.3 If N/PN/NI to 4.1 and 4.2: Were outcome assessors aware of the intervention received by study participants?**	HbA1c is likely to be measured with the same instrumentation at a laboratory that is likely unaware of the intervention received.	PN
**4.4 If Y/PY/NI to 4.3: Could assessment of the outcome have been influenced by knowledge of intervention received?**		NA
**4.5 If Y/PY/NI to 4.4: Is it likely that assessment of the outcome was influenced by knowledge of intervention received?**		NA
**Risk-of-bias judgement**		Low
Optional: What is the predicted direction of bias in measurement of the outcome?		NA

Domain 5: Risk of bias in selection of the reported result

Signalling questions	Comments	Response options
**5.1 Were the data that produced this result analysed in accordance with a pre-specified analysis plan that was finalized before unblinded outcome data were available for analysis?**	Data analysis plan provided in ClinicalTrails.gov registration.	Y
**Is the numerical result being assessed likely to have been selected, on the basis of the results, from...**		
**5.2. ... multiple eligible outcome measurements (e.g. scales, definitions, time points) within the outcome domain?**	Outcome measurements are specified in the preregistration.	N
**5.3 ... multiple eligible analyses of the data?**	Outcome analysis methods are specified in the preregistration.	Y / PY / PN / N / NI
**Risk-of-bias judgement**		Lo
Optional: What is the predicted direction of bias due to selection of the reported result?		NA

Overall risk of bias

**Risk-of-bias judgement**		Lo
Optional: What is the overall predicted direction of bias for this outcome?		N

This work is licensed under a Creative Commons Attribution-NonCommercial-NoDerivatives 4.0 International License.

Supplementary Fig. 39: Risk of Bias Analysis (Fryk 2023)

Study details

**Reference**	Fryk 2023[8]

Study design

☐ Individually-randomized parallel-group trial
☐ Cluster-randomized parallel-group trial
X Individually randomized cross-over (or other matched) trial

For the purposes of this assessment, the interventions being compared are defined as

Experimental:	Tadalafil treatment	Comparator:	Placebo treatment

**Specify which outcome is being assessed for risk of bias**	Endpoint HbA1c concentration
**Specify the numerical result being assessed.** In case of multiple alternative analyses being presented, specify the numeric result (e.g. RR = 1.52 (95% CI 0.83 to 2.77) and/or a reference (e.g. to a table, figure or paragraph) that uniquely defines the result being assessed.	Figure 3a (Table 2, 3 for Domain 1.3)

Is the review team's aim for this result…?

X to assess the effect of *assignment to intervention* (the 'intention-to-treat' effect)
☐ to assess the effect of *adhering to intervention* (the 'per-protocol' effect)

If the aim is to assess the effect of *adhering to intervention*, select the deviations from intended intervention that should be addressed (at least one must be checked):

☐ occurrence of non-protocol interventions
☐ failures in implementing the intervention that could have affected the outcome
☐ non-adherence to their assigned intervention by trial participants

Which of the following sources were obtained to help inform the risk-of-bias assessment? (tick as many as apply)

X Journal article(s) with results of the trial
X Trial protocol

X	Statistical analysis plan (SAP)
X	Non-commercial trial registry record (e.g. ClinicalTrials.gov record)
☐	Company-owned trial registry record (e.g. GSK Clinical Study Register record)
☐	“Grey literature” (e.g. unpublished thesis)
☐	Conference abstract(s) about the trial
☐	Regulatory document (e.g. Clinical Study Report, Drug Approval Package)
☐	Research ethics application
☐	Grant database summary (e.g. NIH RePORTER or Research Councils UK Gateway to Research)
☐	Personal communication with trialist
☐	Personal communication with the sponsor

Risk of bias assessment

Responses underlined in green are potential markers for low risk of bias, and responses in red are potential markers for a risk of bias. Where questions relate only to sign posts to other questions, no formatting is used.

Domain 1a: Risk of bias arising from the randomization process

Signalling questions	Comments	Response options
**1.1 Was the allocation sequence random?**	"The randomisation was conducted by Apoteket Produktion & Laboratorier AB (APL, Stockholm, Sweden)."	PY
**1.2 Was the allocation sequence concealed until participants were enrolled and assigned to interventions?**	"All study personnel, medical staff, and data analysts were blinded to given treatment throughout the study." "Both Cialis (tadalafil) and the placebo are manufactured by Eli Lilly, and the study drug and the placebo are visually indistinguishable"	PY
**1.3 Did baseline differences between intervention groups at the start of the first period suggest a problem with the randomization process?**	Only baseline information of population in full analysis reported. No baseline information on the subgroup assigned to placebo treatment first and the subgroup assigned to tadalafil treatment first was given. Nevertheless, collated baseline information appears uniformly distributed.	PN
**Risk-of-bias judgement**		Low
Optional: What is the predicted direction of bias arising from the randomization process?		NA

Domain S: Risk of bias arising from period and carryover effects

Signalling questions	Comments	Response options
**S.1 Was the number of participants allocated to each of the two sequences equal or nearly equal?**	Randomization of 12 to initial tadalafil treatment and 11 to initial placebo treatment as in Figure 2	Y
**S.2 If N/PN/NI to S.1: Were period effects accounted for in the analysis?**		NA
**S.3 Was there sufficient time for any carryover effects to have disappeared before outcome assessment in the second period?**	"the elimination half-life of tadalafil is 17.5 h and the wash-out period was eight weeks"	Y
**Risk-of-bias judgement**		Low
Optional: What is the predicted direction of bias arising from period and carryover effects?		NA

Domain 2: Risk of bias due to deviations from the intended interventions (effect of assignment to intervention)

Signalling questions	Comments	Response options
**2.1. Were participants aware of their assigned intervention during each period of the trial?**	"All study personnel, medical staff, and data analysts were blinded to given treatment throughout the study." "Both Cialis (tadalafil) and the placebo are manufactured by Eli Lilly, and the study drug and the placebo are visually indistinguishable"	N
**2.2. Were carers and people delivering the interventions aware of participants' assigned intervention during each period of the trial?**		N
**2.3. If Y/PY/NI to 2.1 or 2.2: Were there deviations from the intended intervention that arose because of the trial context?**		NA
**2.4 If Y/PY to 2.3: Were these deviations likely to have affected the outcome?**		NA
**2.5. If Y/PY/NI to 2.4: Were these deviations from intended intervention balanced between groups?**		NA
**2.6 Was an appropriate analysis used to estimate the effect of assignment to intervention?**	A modified intention-to-treat analysis was done in this study. While a per-protocol analysis was also done, the results of the per-protocol analysis were not included in this meta-analysis.	Y
**2.7 If N/PN/NI to 2.6: Was there potential for a substantial impact (on the result) of the failure to analyse participants in the group to which they were randomized?**		NA
**Risk-of-bias judgement**		Low
Optional: What is the predicted direction of bias due to deviations from intended interventions?		NA

Domain 3: Risk of bias due to missing outcome data

<table>
<tr><th>Signalling questions</th><th>Comments</th><th>Response options</th></tr>
<tr><td>3.1 Were data for this outcome available for all, or nearly all, participants randomized?</td><td>Data available for only 18/23 participants as in Figure 2</td><td>N</td></tr>
<tr><td>3.2 If N/PN/NI to 3.1: Is there evidence that the result was not biased by missing outcome data?</td><td>No analysis methods for bias or sensitivity analyses reported</td><td>N</td></tr>
<tr><td>3.3 If N/PN to 3.2 Could missingness in the outcome depend on its true value?</td><td rowspan="2">3 participants excluded due to adverse events on tadalafil or placebo).
All adverse events (pyelonephritis, back pain, persistent cold) do not appear to depend on HbA1c levels. One participant withdrew consent due to illness in the family, which likely does not depend on HbA1c levels. The last excluded participant was done so due to hyperglycemia, but they had only taken placebo treatment by that time and cited lifestyle changes, which suggests that the event did not depend on a drug’s effect on HbA1c levels.</td><td>Y</td></tr>
<tr><td>3.4 If Y/PY/NI to 3.3: Is it likely that missingness in the outcome depended on its true value?</td><td>PN</td></tr>
<tr><td>Risk-of-bias judgement</td><td></td><td>Some concerns</td></tr>
<tr><td>Optional: What is the predicted direction of bias due to missing outcome data?</td><td></td><td>Unpredictable</td></tr>
</table>

Domain 4: Risk of bias in measurement of the outcome

Signalling questions	Comments	Response options
**4.1 Was the method of measuring the outcome inappropriate?**	"Besides safety analyses at the screening visit, we measured HbA1c… All samples were analysed using accredited methods at the Clinical Chemistry Laboratory at Sahlgrenska University Hospital in Gothenburg.".	N
**4.2 Could measurement or ascertainment of the outcome have differed between interventions within each sequence?**	See 4.1	N
**4.3 If N/PN/NI to 4.1 and 4.2: Were outcome assessors aware of the intervention received by study participants?**	See 2.1, 2.2	N
**4.4 If Y/PY/NI to 4.3: Could assessment of the outcome have been influenced by knowledge of intervention received?**		NA
**4.5 If Y/PY/NI to 4.4: Is it likely that assessment of the outcome was influenced by knowledge of intervention received?**		NA
**Risk-of-bias judgement**		Low
Optional: What is the predicted direction of bias in measurement of the outcome?		NA

Domain 5: Risk of bias in selection of the reported result

Signalling questions	Comments	Response options
**5.1 Were the data that produced this result analysed in accordance with a pre-specified analysis plan that was finalized before unblinded outcome data were available for analysis?**	Clinical Study Protocol is included (Fryk 2023, Appendix A, 8-34) as well as a Statistical Analysis Plan.	Y
**Is the numerical result being assessed likely to have been selected, on the basis of the results, from...**		
**5.2. ... multiple eligible outcome measurements (e.g. scales, definitions, time points) within the outcome domain?**	Measurement reported in Clinical Trial Protocol: "Blood sampling for routine check of blood-… status at the accredited Central Laboratory at Sahlgrenska University Hospital is performed."	N
**5.3 ... multiple eligible analyses of the data?**	Only one eligible analysis of the data given. While period-adjusted p-values are given, they are not included in the meta-analysis.	N
**5.4 Is a result based on data from both periods sought, but unavailable on the basis of carryover having been identified?**	Data is not censored on the basis of carryover.	N
**Risk-of-bias judgement**		Low
Optional: What is the predicted direction of bias due to selection of the reported result?		NA

Overall risk of bias

**Risk-of-bias judgement**		Some concerns
Optional: What is the overall predicted direction of bias for this outcome?		Unpredictable

This work is licensed under a Creative Commons Attribution-NonCommercial-NoDerivatives 4.0 International License.

Supplementary Fig. 40: Risk of Bias Analysis (Pofi 2022)

Study details

**Reference**	Pofi 2022[9]

Study design

X Individually-randomized parallel-group trial
☐ Cluster-randomized parallel-group trial
☐ Individually randomized cross-over (or other matched) trial

For the purposes of this assessment, the interventions being compared are defined as

Experimental: Tadalafil treatment Comparator: Placebo treatment

**Specify which outcome is being assessed for risk of bias**	Endpoint HbA1c concentration
**Specify the numerical result being assessed.** In case of multiple alternative analyses being presented, specify the numeric result (e.g. RR = 1.52 (95% CI 0.83 to 2.77) and/or a reference (e.g. to a table, figure or paragraph) that uniquely defines the result being assessed.	Table 3

Is the review team's aim for this result…?

X to assess the effect of *assignment to intervention* (the 'intention-to-treat' effect)
☐ to assess the effect of *adhering to intervention* (the 'per-protocol' effect)

If the aim is to assess the effect of *adhering to intervention*, select the deviations from intended intervention that should be addressed (at least one must be checked):

☐ occurrence of non-protocol interventions
☐ failures in implementing the intervention that could have affected the outcome
☐ non-adherence to their assigned intervention by trial participants

Which of the following sources were <u>obtained</u> to help inform the risk-of-bias assessment? (tick as many as apply)

X Journal article(s) with results of the trial
X Trial protocol

X	Statistical analysis plan (SAP)
X	Non-commercial trial registry record (e.g. ClinicalTrials.gov record)
☐	Company-owned trial registry record (e.g. GSK Clinical Study Register record)
☐	“Grey literature” (e.g. unpublished thesis)
☐	Conference abstract(s) about the trial
☐	Regulatory document (e.g. Clinical Study Report, Drug Approval Package)
☐	Research ethics application
☐	Grant database summary (e.g. NIH RePORTER or Research Councils UK Gateway to Research)
☐	Personal communication with trialist
☐	Personal communication with the sponsor

Risk of bias assessment

Responses underlined in green are potential markers for low risk of bias, and responses in red are potential markers for a risk of bias. Where questions relate only to sign posts to other questions, no formatting is used.

Domain 1: Risk of bias arising from the randomization process

**Signalling questions**	**Comments**	**Response options**
**1.1 Was the allocation sequence random?**	"A computer-generated random sequence stratified randomized participants by diabetes duration (years) and HbA1c. The trial was double-blind for participants, medical team, and magnetic resonance imaging (MRI) assessors"	Y
**1.2 Was the allocation sequence concealed until participants were enrolled and assigned to interventions?**		PY
**1.3 Did baseline differences between intervention groups suggest a problem with the randomization process?**	Although there are many p-values of exactly 1 due to the inherently low number of significant figures of many baseline measurements, the baseline characteristics as in Table 1 appear compatible with random chance and do not suggest excessive similarity or dissimilarity.	N
**Risk-of-bias judgement**		Low
Optional: What is the predicted direction of bias arising from the randomization process?		NA

Domain 2: Risk of bias due to deviations from the intended interventions (*effect of assignment to intervention*)

Signalling questions	Comments	Response options
**2.1. Were participants aware of their assigned intervention during the trial?**	Placebo treatment is included. “The trial was double-blind for participants, medical team, and magnetic resonance imaging (MRI) assessors”	N
**2.2. Were carers and people delivering the interventions aware of participants' assigned intervention during the trial?**		N
**2.3. If Y/PY/NI to 2.1 or 2.2: Were there deviations from the intended intervention that arose because of the trial context?**		NA
**2.4 If Y/PY to 2.3: Were these deviations likely to have affected the outcome?**		NA
**2.5. If Y/PY/NI to 2.4: Were these deviations from intended intervention balanced between groups?**		NA
**2.6 Was an appropriate analysis used to estimate the effect of assignment to intervention?**	Modified intention-to-treat analysis done: “At baseline, two male participants (one in each group) were excluded after myocardial ischemia diagnosis, giving a final intention-to-treat (ITT) population of 122”	Y
**2.7 If N/PN/NI to 2.6: Was there potential for a substantial impact (on the result) of the failure to analyse participants in the group to which they were randomized?**		NA
**Risk-of-bias judgement**		Low
Optional: What is the predicted direction of bias due to deviations from intended interventions?		NA

Domain 3: Missing outcome data

Signalling questions	Comments	Response options
**3.1 Were data for this outcome available for all, or nearly all, participants randomized?**	Outcome data is available for 122/124 randomized participants: "At baseline, two male participants (one in each group) were excluded after myocardial ischemia diagnosis, giving a final intention-to-treat (ITT) population of 122"	Y
**3.2 If N/PN/NI to 3.1: Is there evidence that the result was not biased by missing outcome data?**		NA
**3.3 If N/PN to 3.2: Could missingness in the outcome depend on its true value?**		NA
**3.4 If Y/PY/NI to 3.3: Is it likely that missingness in the outcome depended on its true value?**		NA
**Risk-of-bias judgement**		Low
Optional: What is the predicted direction of bias due to missing outcome data?		NA

Domain 4: Risk of bias in measurement of the outcome

Signalling questions	Comments	Response options
**4.1 Was the method of measuring the outcome inappropriate?**	The exact method of "assessment of biochemical parameters" is not specified in either the journal article or the clinical trial protocol but is likely done using routine, accurate measurements.	PN
**4.2 Could measurement or ascertainment of the outcome have differed between intervention groups?**	All measurements are identical regardless of the intervention group as outlined in the study design of the clinical trial protocol.	N
**4.3 If N/PN/NI to 4.1 and 4.2: Were outcome assessors aware of the intervention received by study participants?**	Placebo treatment is included. "The trial was double-blind for participants, medical team, and magnetic resonance imaging (MRI) assessors"	PN
**4.4 If Y/PY/NI to 4.3: Could assessment of the outcome have been influenced by knowledge of intervention received?**		NA
**4.5 If Y/PY/NI to 4.4: Is it likely that assessment of the outcome was influenced by knowledge of intervention received?**		NA
**Risk-of-bias judgement**		Low
Optional: What is the predicted direction of bias in measurement of the outcome?		NA

Domain 5: Risk of bias in selection of the reported result

Signalling questions	Comments	Response options
**5.1 Were the data that produced this result analysed in accordance with a pre-specified analysis plan that was finalized before unblinded outcome data were available for analysis?**	Statistical analysis included in study protocol.	Y
**Is the numerical result being assessed likely to have been selected, on the basis of the results, from...**		
**5.2. ... multiple eligible outcome measurements (e.g. scales, definitions, time points) within the outcome domain?**	All eligible outcome measurements are reported, as outlined in the study design of the clinical trial protocol.	N
**5.3 ... multiple eligible analyses of the data?**	Only one method of statistical analysis was performed as outlined in the statistical analysis section of the clinical trial protocol.	N
**Risk-of-bias judgement**		Low
Optional: What is the predicted direction of bias due to selection of the reported result?		NA

Overall risk of bias

**Risk-of-bias judgement**		Low
Optional: What is the overall predicted direction of bias for this outcome?		NA

This work is licensed under a Creative Commons Attribution-NonCommercial-NoDerivatives 4.0 International License.

Supplementary Fig. 41: Risk of Bias Analysis (Lee 2022)

Study details

**Reference**	Lee 2022[10]

Study design

X Individually-randomized parallel-group trial
☐ Cluster-randomized parallel-group trial
☐ Individually randomized cross-over (or other matched) trial

For the purposes of this assessment, the interventions being compared are defined as

Experimental: Tadalafil treatment | Comparator: Placebo treatment

**Specify which outcome is being assessed for risk of bias**	Endpoint HbA1c concentration
**Specify the numerical result being assessed.** In case of multiple alternative analyses being presented, specify the numeric result (e.g. RR = 1.52 (95% CI 0.83 to 2.77) and/or a reference (e.g. to a table, figure or paragraph) that uniquely defines the result being assessed.	Table 2 (Table 1 for Domain 1.3)

Is the review team's aim for this result…?

X to assess the effect of *assignment to intervention* (the 'intention-to-treat' effect)
☐ to assess the effect of *adhering to intervention* (the 'per-protocol' effect)

If the aim is to assess the effect of *adhering to intervention*, select the deviations from intended intervention that should be addressed (at least one must be checked):

☐ occurrence of non-protocol interventions
☐ failures in implementing the intervention that could have affected the outcome
☐ non-adherence to their assigned intervention by trial participants

Which of the following sources were obtained to help inform the risk-of-bias assessment? (tick as many as apply)

X Journal article(s) with results of the trial
☐ Trial protocol

- ☐ Statistical analysis plan (SAP)
- ☐ Non-commercial trial registry record (e.g. ClinicalTrials.gov record)
- ☐ Company-owned trial registry record (e.g. GSK Clinical Study Register record)
- ☐ “Grey literature” (e.g. unpublished thesis)
- ☐ Conference abstract(s) about the trial
- ☐ Regulatory document (e.g. Clinical Study Report, Drug Approval Package)
- ☐ Research ethics application
- ☐ Grant database summary (e.g. NIH RePORTER or Research Councils UK Gateway to Research)
- ☐ Personal communication with trialist
- ☐ Personal communication with the sponsor

Risk of bias assessment

Responses underlined in green are potential markers for low risk of bias, and responses in red are potential markers for a risk of bias. Where questions relate only to sign posts to other questions, no formatting is used.

Domain 1: Risk of bias arising from the randomization process

Signalling questions	Comments	Response options
**1.1 Was the allocation sequence random?**	"The allocation list was produced using dedicated software (ID-net™) via permuted-block randomization with 2:1 allocation and randomly sized blocks." "The allocation details were blinded until statistical analysis was completed."	Y
**1.2 Was the allocation sequence concealed until participants were enrolled and assigned to interventions?**		PY
**1.3 Did baseline differences between intervention groups suggest a problem with the randomization process?**	The baseline characteristics in Table 1 do not suggest problematic differences between intervention groups.	N
**Risk-of-bias judgement**		Low
Optional: What is the predicted direction of bias arising from the randomization process?		NA

Domain 2: Risk of bias due to deviations from the intended interventions (*effect of assignment to intervention*)

Signalling questions	Comments	Response options
**2.1. Were participants aware of their assigned intervention during the trial?**	The trial uses a "double-blind, placebo-controlled" design; moreover, "The allocation details were blinded until statistical analysis was completed"	PN
**2.2. Were carers and people delivering the interventions aware of participants' assigned intervention during the trial?**		PN
**2.3. If Y/PY/NI to 2.1 or 2.2: Were there deviations from the intended intervention that arose because of the trial context?**		NA
**2.4 If Y/PY to 2.3: Were these deviations likely to have affected the outcome?**		NA
**2.5. If Y/PY/NI to 2.4: Were these deviations from intended intervention balanced between groups?**		NA
**2.6 Was an appropriate analysis used to estimate the effect of assignment to intervention?**	A modified intention-to-treat analysis likely has been done based on Figure 1	PY
**2.7 If N/PN/NI to 2.6: Was there potential for a substantial impact (on the result) of the failure to analyse participants in the group to which they were randomized?**		NA
**Risk-of-bias judgement**		Low
Optional: What is the predicted direction of bias due to deviations from intended interventions?		NA

Domain 3: Missing outcome data

Signalling questions	Comments	Response options
**3.1 Were data for this outcome available for all, or nearly all, participants randomized?**	Data on 68/75 participants were able to be collected based on Figure 1, which is close to the desired 95% availability	PY
**3.2 If N/PN/NI to 3.1: Is there evidence that the result was not biased by missing outcome data?**		NA
**3.3 If N/PN to 3.2: Could missingness in the outcome depend on its true value?**		NA
**3.4 If Y/PY/NI to 3.3: Is it likely that missingness in the outcome depended on its true value?**		NA
**Risk-of-bias judgement**		Low
Optional: What is the predicted direction of bias due to missing outcome data?		NA

Domain 4: Risk of bias in measurement of the outcome

Signalling questions	Comments	Response options
**4.1 Was the method of measuring the outcome inappropriate?**	"HbA1c levels were measured using turbidimetric inhibition immunoassay".	N
**4.2 Could measurement or ascertainment of the outcome have differed between intervention groups?**	"These variables were checked at baseline and 6 months… HbA1c levels were measured at 3 months".	N
**4.3 If N/PN/NI to 4.1 and 4.2: Were outcome assessors aware of the intervention received by study participants?**	"HbA1c levels were measured using turbidimetric inhibition immunoassay". The trial uses a "double-blind, placebo-controlled" design; moreover, "The allocation details were blinded until statistical analysis was completed"	N
**4.4 If Y/PY/NI to 4.3: Could assessment of the outcome have been influenced by knowledge of intervention received?**		NA
**4.5 If Y/PY/NI to 4.4: Is it likely that assessment of the outcome was influenced by knowledge of intervention received?**		NA
**Risk-of-bias judgement**		Low
Optional: What is the predicted direction of bias in measurement of the outcome?		NA

Domain 5: Risk of bias in selection of the reported result

Signalling questions	Comments	Response options
**5.1 Were the data that produced this result analysed in accordance with a pre-specified analysis plan that was finalized before unblinded outcome data were available for analysis?**	No study protocol or data analysis plan provided, but "The present study protocol was reviewed and approved by the institutional review board of Myongji hospital (approval no. MJH-16-038). The study was conducted following the protocol"	PY
**Is the numerical result being assessed likely to have been selected, on the basis of the results, from...**		
**5.2. ... multiple eligible outcome measurements (e.g. scales, definitions, time points) within the outcome domain?**	"HbA1c levels were measured using turbidimetric inhibition immunoassay". No other methods of HbA1c measurement are specified, but other methods are unlikely to be done.	PN
**5.3 ... multiple eligible analyses of the data?**	There is only one way to analyze change from baseline.	N
**Risk-of-bias judgement**		Low
Optional: What is the predicted direction of bias due to selection of the reported result?		NA

Overall risk of bias

**Risk-of-bias judgement**		Low
Optional: What is the overall predicted direction of bias for this outcome?		NA

This work is licensed under a Creative Commons Attribution-NonCommercial-NoDerivatives 4.0 International License.

Supplementary Fig. 42: Risk of Bias Analysis (Derosa 2022)

Study details

**Reference**	Derosa 2022[11]

Study design

X Individually-randomized parallel-group trial
☐ Cluster-randomized parallel-group trial
☐ Individually randomized cross-over (or other matched) trial

For the purposes of this assessment, the interventions being compared are defined as

Experimental: Avanafil only   Comparator: Placebo

Specify which outcome is being assessed for risk of bias: HbA1c level after medication administration

Specify the numerical result being assessed. In case of multiple alternative analyses being presented, specify the numeric result (e.g. RR = 1.52 (95% CI 0.83 to 2.77) and/or a reference (e.g. to a table, figure or paragraph) that uniquely defines the result being assessed.

HbA1c at baseline and after 3 months for both placebo and sole avanafil treatment (Table 3) 7.5 ± 1.1 7.3 ± 0.9 7.4 ± 1.0 7.4 ± 1.0

Is the review team's aim for this result…?

X to assess the effect of *assignment to intervention* (the 'intention-to-treat' effect)
☐ to assess the effect of *adhering to intervention* (the 'per-protocol' effect)

If the aim is to assess the effect of *adhering to intervention*, select the deviations from intended intervention that should be addressed (at least one must be checked):

☐ occurrence of non-protocol interventions
☐ failures in implementing the intervention that could have affected the outcome
☐ non-adherence to their assigned intervention by trial participants

Which of the following sources were <u>obtained</u> to help inform the risk-of-bias assessment? (tick as many as apply)

X Journal article(s) with results of the trial
☐ Trial protocol

☐ Statistical analysis plan (SAP)
☐ Non-commercial trial registry record (e.g. ClinicalTrials.gov record)
☐ Company-owned trial registry record (e.g. GSK Clinical Study Register record)
☐ “Grey literature” (e.g. unpublished thesis)
☐ Conference abstract(s) about the trial
☐ Regulatory document (e.g. Clinical Study Report, Drug Approval Package)
☐ Research ethics application
☐ Grant database summary (e.g. NIH RePORTER or Research Councils UK Gateway to Research)
☐ Personal communication with trialist
☐ Personal communication with the sponsor

Risk of bias assessment

Responses underlined in green are potential markers for low risk of bias, and responses in red are potential markers for a risk of bias. Where questions relate only to sign posts to other questions, no formatting is used.

Domain 1: Risk of bias arising from the randomization process

Signalling questions	Comments	Response options
**1.1 Was the allocation sequence random?**	The randomization method is not specified.	NI
**1.2 Was the allocation sequence concealed until participants were enrolled and assigned to interventions?**	The clinical trial is described as “double-blind”.	PY
**1.3 Did baseline differences between intervention groups suggest a problem with the randomization process?**	Baseline values appear to be uniformly distributed, although it seems to exhibit a high degree of collinearity.	N
**Risk-of-bias judgement**		Low
Optional: What is the predicted direction of bias arising from the randomization process?		NA

Domain 2: Risk of bias due to deviations from the intended interventions (*effect of assignment to intervention*)

Signalling questions	Comments	Response options
**2.1. Were participants aware of their assigned intervention during the trial?**	The clinical trial is described as “double-blind”.	PN
**2.2. Were carers and people delivering the interventions aware of participants' assigned intervention during the trial?**		PN
**2.3. If Y/PY/NI to 2.1 or 2.2: Were there deviations from the intended intervention that arose because of the trial context?**		NA
**2.4 If Y/PY to 2.3: Were these deviations likely to have affected the outcome?**		NA
**2.5. If Y/PY/NI to 2.4: Were these deviations from intended intervention balanced between groups?**		NA
**2.6 Was an appropriate analysis used to estimate the effect of assignment to intervention?**	While the trial says to have conducted a “intent-to-treat analysis”, it only analysed participants that received at least one dose of treatment, which is not an appropriate form of modified intention to treat analysis for this purpose.	N
**2.7 If N/PN/NI to 2.6: Was there potential for a substantial impact (on the result) of the failure to analyse participants in the group to which they were randomized?**	0/30 (0%) of participants on placebo and 1/31 (3%) of participants on treatment were not analysed, which is very small (<5%) and likely not substantial due to HbA1c being a continuous outcome and due to exclusions for this reason likely not being related to prognostic factors.	N
**Risk-of-bias judgement**		Some concerns
Optional: What is the predicted direction of bias due to deviations from intended interventions?		Unpredictable

Domain 3: Missing outcome data

Signalling questions	Comments	Response options
**3.1 Were data for this outcome available for all, or nearly all, participants randomized?**	It is not specified if data is available for the 1/31 (3%) participants on treatment (with the 0/30 participants on placebo with missing data), but data is available for at least 95% of participants. There is low risk for missing data to affect the estimated effect of intervention, as detailed in Domain 2.7.	Y
**3.2 If N/PN/NI to 3.1: Is there evidence that the result was not biased by missing outcome data?**		NA
**3.3 If N/PN to 3.2: Could missingness in the outcome depend on its true value?**		NA
**3.4 If Y/PY/NI to 3.3: Is it likely that missingness in the outcome depended on its true value?**		NA
**Risk-of-bias judgement**		Low
Optional: What is the predicted direction of bias due to missing outcome data?		NA

Domain 4: Risk of bias in measurement of the outcome

Signalling questions	Comments	Response options
**4.1 Was the method of measuring the outcome inappropriate?**	Though referral to a previous paper, HbA1c was measured with standard HPLC methods, which is a well-established method.[12]	N
**4.2 Could measurement or ascertainment of the outcome have differed between intervention groups?**	Through referral to a previous paper, HbA1c was always measured with the same method, as detailed in Domain 4.1.[12]	N
**4.3 If N/PN/NI to 4.1 and 4.2: Were outcome assessors aware of the intervention received by study participants?**	"All measurements were performed in a central laboratory", which is very likely to not know the intervention received by study participants.	PN
**4.4 If Y/PY/NI to 4.3: Could assessment of the outcome have been influenced by knowledge of intervention received?**		NA
**4.5 If Y/PY/NI to 4.4: Is it likely that assessment of the outcome was influenced by knowledge of intervention received?**		NA
**Risk-of-bias judgement**		Low
Optional: What is the predicted direction of bias in measurement of the outcome?		NA

Domain 5: Risk of bias in selection of the reported result

Signalling questions	Comments	Response options
**5.1 Were the data that produced this result analysed in accordance with a pre-specified analysis plan that was finalized before unblinded outcome data were available for analysis?**	A study analysis plan was not specified to have been prepared.	NI
**Is the numerical result being assessed likely to have been selected, on the basis of the results, from...**		
**5.2. ... multiple eligible outcome measurements (e.g. scales, definitions, time points) within the outcome domain?**	Only one type of measurement for HbA1c was specified to have been taken, as detailed in in Domain 4.1.	N
**5.3 ... multiple eligible analyses of the data?**	While several types of analyses are possible, a quick inspection of recent clinical trials conducted by the same authors suggests that the analysis chosen was not selected from multiple eligible analysis: only analysing data for participants with at least one dose and one measurement as well as only reporting endpoint values without data imputation.[13,14]	PN
**Risk-of-bias judgement**		Low
Optional: What is the predicted direction of bias due to selection of the reported result?		NA

Overall risk of bias

**Risk-of-bias judgement**		Some concerns
Optional: What is the overall predicted direction of bias for this outcome?		Unpredictable

This work is licensed under a Creative Commons Attribution-NonCommercial-NoDerivatives 4.0 International License.

Supplementary Fig. 43: Risk of Bias Analysis (Liu 2016)

Study details

**Reference**	Liu 2016[15]

Study design

X Individually-randomized parallel-group trial
☐ Cluster-randomized parallel-group trial
☐ Individually randomized cross-over (or other matched) trial

For the purposes of this assessment, the interventions being compared are defined as

Experimental: Sildenafil treatment | Comparator: Placebo treatment

**Specify which outcome is being assessed for risk of bias**	HbA1c levels post-intervention
**Specify the numerical result being assessed.** In case of multiple alternative analyses being presented, specify the numeric result (e.g. RR = 1.52 (95% CI 0.83 to 2.77) and/or a reference (e.g. to a table, figure or paragraph) that uniquely defines the result being assessed.	Table 4: Week 12 HbA1c for sildenafil, placebo treatment groups (Table 1, 2, 3b, 4 for Domain 1.3)

Is the review team's aim for this result…?

X to assess the effect of *assignment to intervention* (the 'intention-to-treat' effect)
☐ to assess the effect of *adhering to intervention* (the 'per-protocol' effect)

If the aim is to assess the effect of *adhering to intervention*, select the deviations from intended intervention that should be addressed (at least one must be checked):

☐ occurrence of non-protocol interventions
☐ failures in implementing the intervention that could have affected the outcome
☐ non-adherence to their assigned intervention by trial participants

Which of the following sources were <u>obtained</u> to help inform the risk-of-bias assessment? (tick as many as apply)

X Journal article(s) with results of the trial
☐ Trial protocol
☐ Statistical analysis plan (SAP)

X	Non-commercial trial registry record (e.g. ClinicalTrials.gov record)
☐	Company-owned trial registry record (e.g. GSK Clinical Study Register record)
☐	“Grey literature” (e.g. unpublished thesis)
☐	Conference abstract(s) about the trial
☐	Regulatory document (e.g. Clinical Study Report, Drug Approval Package)
☐	Research ethics application
☐	Grant database summary (e.g. NIH RePORTER or Research Councils UK Gateway to Research)
☐	Personal communication with trialist
☐	Personal communication with the sponsor

Risk of bias assessment

Responses underlined in green are potential markers for low risk of bias, and responses in red are potential markers for a risk of bias. Where questions relate only to sign posts to other questions, no formatting is used.

Domain 1: Risk of bias arising from the randomization process

Signalling questions	Comments	Response options
**1.1 Was the allocation sequence random?**	Allocation was randomized via computer program, as specified in the methods.[16]	Y
**1.2 Was the allocation sequence concealed until participants were enrolled and assigned to interventions?**	The trial is described as double-blind, as specified in the methods.[16]	PY
**1.3 Did baseline differences between intervention groups suggest a problem with the randomization process?**	Baseline p-values appear to be approximately uniformly distributed.	N
**Risk-of-bias judgement**		Low
Optional: What is the predicted direction of bias arising from the randomization process?		NA

Domain 2: Risk of bias due to deviations from the intended interventions (*effect of assignment to intervention*)

Signalling questions	Comments	Response options
**2.1. Were participants aware of their assigned intervention during the trial?**	The trial is described as double-blind and participants were given sildenafil or a identical placebo provided in identically masked kits by a third party, as specified in the methods.[16]	N
**2.2. Were carers and people delivering the interventions aware of participants' assigned intervention during the trial?**		N
**2.3. If Y/PY/NI to 2.1 or 2.2: Were there deviations from the intended intervention that arose because of the trial context?**		NA
**2.4 If Y/PY to 2.3: Were these deviations likely to have affected the outcome?**		NA
**2.5. If Y/PY/NI to 2.4: Were these deviations from intended intervention balanced between groups?**		NA
**2.6 Was an appropriate analysis used to estimate the effect of assignment to intervention?**	An intention-to-treat analysis was correctly done. The effects of data imputation are examined in Domain 3.1.	Y
**2.7 If N/PN/NI to 2.6: Was there potential for a substantial impact (on the result) of the failure to analyse participants in the group to which they were randomized?**		NA
**Risk-of-bias judgement**		Low
Optional: What is the predicted direction of bias due to deviations from intended interventions?		NA

Domain 3: Missing outcome data

Signalling questions	Comments	Response options
**3.1 Were data for this outcome available for all, or nearly all, participants randomized?**	"Missing" data supplemental figure S1 could not be located. "Complete haemodynamics" are available for 21/26 sildenafil participants and 22/26 placebo participants with other data imputed, as specified in the methods,[16] with a significant portion of data missing.	PN
**3.2 If N/PN/NI to 3.1: Is there evidence that the result was not biased by missing outcome data?**	Corrections for bias (other than data imputation) are not reported.	N
**3.3 If N/PN to 3.2: Could missingness in the outcome depend on its true value?**	Both groups had missing data for similar reasons. Missing data due to deaths were very small (<5%) and are unlikely to be affected by changes HbA1c over this short time period.	PN
**3.4 If Y/PY/NI to 3.3: Is it likely that missingness in the outcome depended on its true value?**		NA
**Risk-of-bias judgement**		Low
Optional: What is the predicted direction of bias due to missing outcome data?		NA

Domain 4: Risk of bias in measurement of the outcome

Signalling questions	Comments	Response options
**4.1 Was the method of measuring the outcome inappropriate?**	Blood sampling was performed in a private laboratory that are very likely performed with standard, accurate techniques.	PN
**4.2 Could measurement or ascertainment of the outcome have differed between intervention groups?**	All blood sampling was done at the same laboratory.	N
**4.3 If N/PN/NI to 4.1 and 4.2: Were outcome assessors aware of the intervention received by study participants?**	Blood sampling was performed by a third party laboratory that is very likely unaware of the intervention received.	PN
**4.4 If Y/PY/NI to 4.3: Could assessment of the outcome have been influenced by knowledge of intervention received?**		NA
**4.5 If Y/PY/NI to 4.4: Is it likely that assessment of the outcome was influenced by knowledge of intervention received?**		NA
**Risk-of-bias judgement**		Low
Optional: What is the predicted direction of bias in measurement of the outcome?		NA

Domain 5: Risk of bias in selection of the reported result

Signalling questions	Comments	Response options
**5.1 Were the data that produced this result analysed in accordance with a pre-specified analysis plan that was finalized before unblinded outcome data were available for analysis?**	Although the analysis plan is not shared, methods such as the "substitution/imputation approach was determined prior to unblinding during the determination of the study analysis sets."	PY
**Is the numerical result being assessed likely to have been selected, on the basis of the results, from...**		
**5.2. ... multiple eligible outcome measurements (e.g. scales, definitions, time points) within the outcome domain?**	Outcome measurements were specified before trial completion in the corresponding clinical registry.[17]	N
**5.3 ... multiple eligible analyses of the data?**	Although the analysis plan is not shared, methods were likely determined before the trial; see Domain 5.1.	PN
**Risk-of-bias judgement**		Low
Optional: What is the predicted direction of bias due to selection of the reported result?		NA

Overall risk of bias

**Risk-of-bias judgement**		Low
Optional: What is the overall predicted direction of bias for this outcome?		NA

This work is licensed under a Creative Commons Attribution-NonCommercial-NoDerivatives 4.0 International License.

Supplementary Fig. 44: Risk of Bias Analysis (Scheele 2016)

Study details

**Reference**	Scheele 2016[18]

Study design

X Individually-randomized parallel-group trial
☐ Cluster-randomized parallel-group trial
☐ Individually randomized cross-over (or other matched) trial

For the purposes of this assessment, the interventions being compared are defined as

Experimental:	PF-00489791 treatment	Comparator:	Placebo treatment

**Specify which outcome is being assessed for risk of bias**	Endpoint HbA1c
**Specify the numerical result being assessed.** In case of multiple alternative analyses being presented, specify the numeric result (e.g. RR = 1.52 (95% CI 0.83 to 2.77) and/or a reference (e.g. to a table, figure or paragraph) that uniquely defines the result being assessed.	"Analysis of change from baseline in glycosylated hemoglobin (HbA1c) at week 12 revealed a statistically significant mean decrease of 0.3% in the PF-00489791 group compared with a mean increase of 0.1% in the placebo group." as well as Supplementary Material. For Domain 1.3, Table 1.

Is the review team's aim for this result…?

X to assess the effect of *assignment to intervention* (the 'intention-to-treat' effect)
☐ to assess the effect of *adhering to intervention* (the 'per-protocol' effect)

If the aim is to assess the effect of *adhering to intervention*, select the deviations from intended intervention that should be addressed (at least one must be checked):

☐ occurrence of non-protocol interventions
☐ failures in implementing the intervention that could have affected the outcome
☐ non-adherence to their assigned intervention by trial participants

Which of the following sources were <u>obtained</u> to help inform the risk-of-bias assessment? (tick as many as apply)

X Journal article(s) with results of the trial

☐ Trial protocol
☐ Statistical analysis plan (SAP)
☐ Non-commercial trial registry record (e.g. ClinicalTrials.gov record)
☐ Company-owned trial registry record (e.g. GSK Clinical Study Register record)
☐ "Grey literature" (e.g. unpublished thesis)
☐ Conference abstract(s) about the trial
☐ Regulatory document (e.g. Clinical Study Report, Drug Approval Package)
☐ Research ethics application
☐ Grant database summary (e.g. NIH RePORTER or Research Councils UK Gateway to Research)
☐ Personal communication with trialist
☐ Personal communication with the sponsor

Risk of bias assessment

Responses underlined in green are potential markers for low risk of bias, and responses in red are potential markers for a risk of bias. Where questions relate only to sign posts to other questions, no formatting is used.

Domain 1: Risk of bias arising from the randomization process

Signalling questions	Comments	Response options
**1.1 Was the allocation sequence random?**	Allocation was randomized by computer.	Y
**1.2 Was the allocation sequence concealed until participants were enrolled and assigned to interventions?**	The study is double-blind, and treatment was masked during randomization.	Y
**1.3 Did baseline differences between intervention groups suggest a problem with the randomization process?**	Baseline p-values appear to be approximately uniformly distributed.	N
**Risk-of-bias judgement**		Low
Optional: What is the predicted direction of bias arising from the randomization process?		NA

Domain 2: Risk of bias due to deviations from the intended interventions (*effect of assignment to intervention*)

Signalling questions	Comments	Response options
**2.1. Were participants aware of their assigned intervention during the trial?**	The study is double-blind and the treatments are specified to be masked while interventions were given.	N
**2.2. Were carers and people delivering the interventions aware of participants' assigned intervention during the trial?**		N
**2.3. If Y/PY/NI to 2.1 or 2.2: Were there deviations from the intended intervention that arose because of the trial context?**		NA
**2.4 If Y/PY to 2.3: Were these deviations likely to have affected the outcome?**		NA
**2.5. If Y/PY/NI to 2.4: Were these deviations from intended intervention balanced between groups?**		NA
**2.6 Was an appropriate analysis used to estimate the effect of assignment to intervention?**	A per protocol analysis was completed.	N
**2.7 If N/PN/NI to 2.6: Was there potential for a substantial impact (on the result) of the failure to analyse participants in the group to which they were randomized?**	Only 3/256 (1.2%) of participants were excluded from a potential intention-to-treat analysis, which is very small especially for a continuous outcome. Missing data is analysed in Domain 3.1.	N
**Risk-of-bias judgement**		Some concerns
Optional: What is the predicted direction of bias due to deviations from intended interventions?	Exclusion due to violation protocol likely causes deviation away from null compared to intention-to-treat analysis (which is itself biased towards null).	Away from null

Domain 3: Missing outcome data

Signalling questions	Comments	Response options
**3.1 Were data for this outcome available for all, or nearly all, participants randomized?**	Apart from subjects excluded on the basis of protocol violation (examined in Domain 2.7), 28/256 (10.9%) of participants had data that was likely missing.	N
**3.2 If N/PN/NI to 3.1: Is there evidence that the result was not biased by missing outcome data?**	20 participants had missing data due to not meeting entrance criteria, which cannot depend on the true value of HbA1c, or adverse event likely unrelated to HbA1c for this drug. Up to 5 participants could have withdrawn due to hypoglycemia or hyperglycemia. Excluding these participants, 13/256 (5.1%) participants with missing data could be affected by the true value of HbA1c, which is sufficiently small for this continuous outcome.	Y
**3.3 If N/PN to 3.2: Could missingness in the outcome depend on its true value?**		NA
**3.4 If Y/PY/NI to 3.3: Is it likely that missingness in the outcome depended on its true value?**		NA
**Risk-of-bias judgement**		Low
Optional: What is the predicted direction of bias due to missing outcome data?		NA

Domain 4: Risk of bias in measurement of the outcome

Signalling questions	Comments	Response options
**4.1 Was the method of measuring the outcome inappropriate?**	Blood sampling was performed in a laboratory that are very likely performed with standard, accurate techniques.	PN
**4.2 Could measurement or ascertainment of the outcome have differed between intervention groups?**	All blood sampling was very likely to have been done at the same laboratory with the same methods of measurement.	PN
**4.3 If N/PN/NI to 4.1 and 4.2: Were outcome assessors aware of the intervention received by study participants?**	Laboratory values were measured by a laboratory that is very likely unaware of the intervention received.	PN
**4.4 If Y/PY/NI to 4.3: Could assessment of the outcome have been influenced by knowledge of intervention received?**		NA
**4.5 If Y/PY/NI to 4.4: Is it likely that assessment of the outcome was influenced by knowledge of intervention received?**		NA
**Risk-of-bias judgement**		Low
Optional: What is the predicted direction of bias in measurement of the outcome?		NA

Domain 5: Risk of bias in selection of the reported result

Signalling questions	Comments	Response options
**5.1 Were the data that produced this result analysed in accordance with a pre-specified analysis plan that was finalized before unblinded outcome data were available for analysis?**	Analysis methods are specified to be "predetermined", although the analysis plan is not available.	Y
**Is the numerical result being assessed likely to have been selected, on the basis of the results, from...**		
**5.2. ... multiple eligible outcome measurements (e.g. scales, definitions, time points) within the outcome domain?**	Endpoint HbA1c outcomes were very likely only measured once at the end of the study, and variation of outcome measurement is unlikely.	N
**5.3 ... multiple eligible analyses of the data?**	Analysis methods are specified to be predetermined.	N
**Risk-of-bias judgement**		Low
Optional: What is the predicted direction of bias due to selection of the reported result?		NA

Overall risk of bias

**Risk-of-bias judgement**		Some concerns
Optional: What is the overall predicted direction of bias for this outcome?		Away from null

This work is licensed under a Creative Commons Attribution-NonCommercial-NoDerivatives 4.0 International License.

Supplementary Fig. 45: Risk of Bias Analysis (Kirilmaz 2015)

Study details

**Reference**	Kirilmaz 2015[19]

Study design

X Individually-randomized parallel-group trial
☐ Cluster-randomized parallel-group trial
☐ Individually randomized cross-over (or other matched) trial

For the purposes of this assessment, the interventions being compared are defined as

Experimental: Sildenafil treatment | Comparator: Placebo Treatment

Specify which outcome is being assessed for risk of bias: Endpoint HbA1c after treatment

Specify the numerical result being assessed. In case of multiple alternative analyses being presented, specify the numeric result (e.g. RR = 1.52 (95% CI 0.83 to 2.77) and/or a reference (e.g. to a table, figure or paragraph) that uniquely defines the result being assessed.: Figure 1

Is the review team's aim for this result…?

X to assess the effect of *assignment to intervention* (the 'intention-to-treat' effect)
☐ to assess the effect of *adhering to intervention* (the 'per-protocol' effect)

If the aim is to assess the effect of *adhering to intervention*, select the deviations from intended intervention that should be addressed (at least one must be checked):

☐ occurrence of non-protocol interventions
☐ failures in implementing the intervention that could have affected the outcome
☐ non-adherence to their assigned intervention by trial participants

Which of the following sources were <u>obtained</u> to help inform the risk-of-bias assessment? (tick as many as apply)

X Journal article(s) with results of the trial
☐ Trial protocol
☐ Statistical analysis plan (SAP)

- ☐ Non-commercial trial registry record (e.g. ClinicalTrials.gov record)
- ☐ Company-owned trial registry record (e.g. GSK Clinical Study Register record)
- ☐ “Grey literature” (e.g. unpublished thesis)
- ☐ Conference abstract(s) about the trial
- ☐ Regulatory document (e.g. Clinical Study Report, Drug Approval Package)
- ☐ Research ethics application
- ☐ Grant database summary (e.g. NIH RePORTER or Research Councils UK Gateway to Research)
- ☐ Personal communication with trialist
- ☐ Personal communication with the sponsor

Risk of bias assessment

Responses underlined in green are potential markers for low risk of bias, and responses in red are potential markers for a risk of bias. Where questions relate only to sign posts to other questions, no formatting is used.

Domain 1: Risk of bias arising from the randomization process

Signalling questions	Comments	Response options
**1.1 Was the allocation sequence random?**	Allocation is “randomized”, but randomization tools used are not specified.	NI
**1.2 Was the allocation sequence concealed until participants were enrolled and assigned to interventions?**	While not specified in this study, other trials by several of the authors suggest that blinding was done but not explicitly stated.[20]	PY
**1.3 Did baseline differences between intervention groups suggest a problem with the randomization process?**	Baseline p-values appear to be uniformly distributed.	N
**Risk-of-bias judgement**		Low
Optional: What is the predicted direction of bias arising from the randomization process?		NA

Domain 2: Risk of bias due to deviations from the intended interventions (*effect of assignment to intervention*)

Signalling questions	Comments	Response options
**2.1. Were participants aware of their assigned intervention during the trial?**	A placebo is not specified to have been given.	PY
**2.2. Were carers and people delivering the interventions aware of participants' assigned intervention during the trial?**		PY
**2.3. If Y/PY/NI to 2.1 or 2.2: Were there deviations from the intended intervention that arose because of the trial context?**	While not specified in this article, other trials by several of the authors reported in more detail suggest that deviations from intended interventions were not allowed in general.[21]	PN
**2.4 If Y/PY to 2.3: Were these deviations likely to have affected the outcome?**		NA
**2.5. If Y/PY/NI to 2.4: Were these deviations from intended intervention balanced between groups?**		NA
**2.6 Was an appropriate analysis used to estimate the effect of assignment to intervention?**	Participants were only excluded from analysis if they discontinued due to medication complications and inadequate compliance with treatment is cited as a limitation of the study, which suggest a proper modified intention-to-treat analysis.	PY
**2.7 If N/PN/NI to 2.6: Was there potential for a substantial impact (on the result) of the failure to analyse participants in the group to which they were randomized?**		NA
**Risk-of-bias judgement**		Low
Optional: What is the predicted direction of bias due to deviations from intended interventions?		NA

Domain 3: Missing outcome data

Signalling questions	Comments	Response options
**3.1 Were data for this outcome available for all, or nearly all, participants randomized?**	The number of participants excluded due to medication side effects is not specified.	NI
**3.2 If N/PN/NI to 3.1: Is there evidence that the result was not biased by missing outcome data?**	Sensitivity analysis is not performed.	N
**3.3 If N/PN to 3.2: Could missingness in the outcome depend on its true value?**	Missingness of outcome is due to side effects of headache, dizziness, and dyspepsia are likely unrelated to HbA1c.	NI
**3.4 If Y/PY/NI to 3.3: Is it likely that missingness in the outcome depended on its true value?**		N
**Risk-of-bias judgement**		Some concerns
Optional: What is the predicted direction of bias due to missing outcome data?		Unpredictable

Domain 4: Risk of bias in measurement of the outcome

Signalling questions	Comments	Response options
**4.1 Was the method of measuring the outcome inappropriate?**	Serum sampling was very likely performed in a laboratory using standard, accurate techniques.	PN
**4.2 Could measurement or ascertainment of the outcome have differed between intervention groups?**	All blood sampling was very likely to have been done at the same laboratory with the same methods of measurement.	PN
**4.3 If N/PN/NI to 4.1 and 4.2: Were outcome assessors aware of the intervention received by study participants?**	Laboratory values were very likely measured by a laboratory that was unaware of the intervention received.	PN
**4.4 If Y/PY/NI to 4.3: Could assessment of the outcome have been influenced by knowledge of intervention received?**		NA
**4.5 If Y/PY/NI to 4.4: Is it likely that assessment of the outcome was influenced by knowledge of intervention received?**		NA
**Risk-of-bias judgement**		Low
Optional: What is the predicted direction of bias in measurement of the outcome?		NA

Domain 5: Risk of bias in selection of the reported result

Signalling questions	Comments	Response options
**5.1 Were the data that produced this result analysed in accordance with a pre-specified analysis plan that was finalized before unblinded outcome data were available for analysis?**	An analysis plan was not specified to have been prepared before data analysis.	NI
**Is the numerical result being assessed likely to have been selected, on the basis of the results, from...**		
**5.2. ... multiple eligible outcome measurements (e.g. scales, definitions, time points) within the outcome domain?**	Endpoint HbA1c outcomes were very likely only measured once at the end of the study, and variation of outcome measurement is unlikely.	PN
**5.3 ... multiple eligible analyses of the data?**	Other trials by several of the authors reported in more detail suggest that the analysis performed is standard.[21]	PN
**Risk-of-bias judgement**		Some concerns
Optional: What is the predicted direction of bias due to selection of the reported result?		Unpredictable

Overall risk of bias

**Risk-of-bias judgement**		Some concerns
Optional: What is the overall predicted direction of bias for this outcome?		Unpredictable

This work is licensed under a Creative Commons Attribution-NonCommercial-NoDerivatives 4.0 International License.

Supplementary Fig. 46: Risk of Bias Analysis (Khazaal 2014)

Study details

**Reference**	Khazaal et al. (2014)[22]

Study design

X Individually-randomized parallel-group trial
☐ Cluster-randomized parallel-group trial
☐ Individually randomized cross-over (or other matched) trial

For the purposes of this assessment, the interventions being compared are defined as

Experimental: PDE5 inhibitor treatment   Comparator: Placebo treatment

**Specify which outcome is being assessed for risk of bias**	Endpoint HbA1c concentration
**Specify the numerical result being assessed.** In case of multiple alternative analyses being presented, specify the numeric result (e.g. RR = 1.52 (95% CI 0.83 to 2.77) and/or a reference (e.g. to a table, figure or paragraph) that uniquely defines the result being assessed.	Table 2, Table 3

Is the review team's aim for this result…?

X to assess the effect of *assignment to intervention* (the 'intention-to-treat' effect)
☐ to assess the effect of *adhering to intervention* (the 'per-protocol' effect)

If the aim is to assess the effect of *adhering to intervention*, select the deviations from intended intervention that should be addressed (at least one must be checked):

☐ occurrence of non-protocol interventions
☐ failures in implementing the intervention that could have affected the outcome
☐ non-adherence to their assigned intervention by trial participants

Which of the following sources were obtained to help inform the risk-of-bias assessment? (tick as many as apply)

X Journal article(s) with results of the trial
☐ Trial protocol

- ☐ Statistical analysis plan (SAP)
- ☐ Non-commercial trial registry record (e.g. ClinicalTrials.gov record)
- ☐ Company-owned trial registry record (e.g. GSK Clinical Study Register record)
- ☐ “Grey literature” (e.g. unpublished thesis)
- ☐ Conference abstract(s) about the trial
- ☐ Regulatory document (e.g. Clinical Study Report, Drug Approval Package)
- ☐ Research ethics application
- ☐ Grant database summary (e.g. NIH RePORTER or Research Councils UK Gateway to Research)
- ☐ Personal communication with trialist
- ☐ Personal communication with the sponsor

Risk of bias assessment

Responses underlined in green are potential markers for low risk of bias, and responses in red are potential markers for a risk of bias. Where questions relate only to sign posts to other questions, no formatting is used.

Domain 1: Risk of bias arising from the randomization process

Signalling questions	Comments	Response options
**1.1 Was the allocation sequence random?**	Study randomization method is not specified. Though the study authors describe the study as a "case control comparative study," the prospective nature and randomization performed suggest that this is better classified as an RCT.	NI
**1.2 Was the allocation sequence concealed until participants were enrolled and assigned to interventions?**	Study blinding not specified, but no suspicion of lack of allocation concealment.	NI
**1.3 Did baseline differences between intervention groups suggest a problem with the randomization process?**	1/10 (10%) baseline p values are significant at the 95% confidence level, which is close to the 5% proportion expected.	N
**Risk-of-bias judgement**		Some concerns
Optional: What is the predicted direction of bias arising from the randomization process?		Unpredictable

Domain 2: Risk of bias due to deviations from the intended interventions (*effect of assignment to intervention*)

Signalling questions	Comments	Response options
**2.1. Were participants aware of their assigned intervention during the trial?**	The control group was not specified to have been given a placebo.	PY
**2.2. Were carers and people delivering the interventions aware of participants' assigned intervention during the trial?**		PY
**2.3. If Y/PY/NI to 2.1 or 2.2: Were there deviations from the intended intervention that arose because of the trial context?**	Lifestyle medications and prescribed diabetic medications were continued. No change of therapy was allowed during treatment. All participants who did not successfully complete the program were due to not attending the clinic on time, which is likely irrelevant to the trial context.	PN
**2.4 If Y/PY to 2.3: Were these deviations likely to have affected the outcome?**		NA
**2.5. If Y/PY/NI to 2.4: Were these deviations from intended intervention balanced between groups?**		NA
**2.6 Was an appropriate analysis used to estimate the effect of assignment to intervention?**	Although "after 8 weeks all 40 participants reexamined and investigated as before starting the therapy", figures only showing a subset of the population and tables with data that suggest division by 17 or 18 suggest that a per-protocol analysis was performed.	PN
**2.7 If N/PN/NI to 2.6: Was there potential for a substantial impact (on the result) of the failure to analyse participants in the group to which they were randomized?**	The amount of missing data is moderate (10% - 15%). A1c is a quantitative result and the exclusion reason (not attending the clinic on time) is likely not strongly correlated with a change in either direction of A1c.	PN
**Risk-of-bias judgement**		Some concerns
Optional: What is the predicted direction of bias due to deviations from intended interventions?		Unpredictable

Domain 3: Missing outcome data

Signalling questions	Comments	Response options
**3.1 Were data for this outcome available for all, or nearly all, participants randomized?**	The study specifies that "after 8 weeks all 40 participants reexamined and investigated as before starting the therapy", suggesting that data is available for all participants that were randomized, though not reported (as analysed in Domain 2)	PY
**3.2 If N/PN/NI to 3.1: Is there evidence that the result was not biased by missing outcome data?**		NA
**3.3 If N/PN to 3.2: Could missingness in the outcome depend on its true value?**		NA
**3.4 If Y/PY/NI to 3.3: Is it likely that missingness in the outcome depended on its true value?**		NA
**Risk-of-bias judgement**		Low
Optional: What is the predicted direction of bias due to missing outcome data?	There is no risk of bias due to missing outcome data because there is no missing outcome data.	NA

Domain 4: Risk of bias in measurement of the outcome

Signalling questions	Comments	Response options
**4.1 Was the method of measuring the outcome inappropriate?**	HbA1c measurement methods were not specified, but were likely performed using well-established methods.	PN
**4.2 Could measurement or ascertainment of the outcome have differed between intervention groups?**	HbA1c data was collected at the same prespecified intervals regardless of intervention.	N
**4.3 If N/PN/NI to 4.1 and 4.2: Were outcome assessors aware of the intervention received by study participants?**	The control group was not specified to have been given a placebo.	PY
**4.4 If Y/PY/NI to 4.3: Could assessment of the outcome have been influenced by knowledge of intervention received?**	HbA1c measurement data does not involve human judgement.	N
**4.5 If Y/PY/NI to 4.4: Is it likely that assessment of the outcome was influenced by knowledge of intervention received?**		NA
**Risk-of-bias judgement**		Low
Optional: What is the predicted direction of bias in measurement of the outcome?		NA

Domain 5: Risk of bias in selection of the reported result

Signalling questions	Comments	Response options
**5.1 Were the data that produced this result analysed in accordance with a pre-specified analysis plan that was finalized before unblinded outcome data were available for analysis?**	This study does not report if a pre-specific analysis plan was prepared.	NI
**Is the numerical result being assessed likely to have been selected, on the basis of the results, from...**		
**5.2. ... multiple eligible outcome measurements (e.g. scales, definitions, time points) within the outcome domain?**	Although there are multiple ways to measure HbA1c, they are unlikely to change the measured outcome. It is also unlikely for multiple different types of measurement of HbA1c to have been done as it is routine clinical data.	N
**5.3 ... multiple eligible analyses of the data?**	An intention-to-treat analysis could have been performed, but whether the intention-to-treat analysis was actually performed is not stated. Other studies by the author does not perform intention-to-treat analysis.	NI
**Risk-of-bias judgement**		Some concerns
Optional: What is the predicted direction of bias due to selection of the reported result?		Unpredictable

Overall risk of bias

**Risk-of-bias judgement**		Some concerns
Optional: What is the overall predicted direction of bias for this outcome?		Unpredictable

This work is licensed under a Creative Commons Attribution-NonCommercial-NoDerivatives 4.0 International License.

Supplementary Fig. 47: Risk of Bias Analysis (Giannetta 2012)

Study details

**Reference**	Giannetta 2012[23]

Study design

X Individually-randomized parallel-group trial
☐ Cluster-randomized parallel-group trial
☐ Individually randomized cross-over (or other matched) trial

For the purposes of this assessment, the interventions being compared are defined as

Experimental: Sildenafil treatment
Comparator: Placebo treatment

**Specify which outcome is being assessed for risk of bias**	Endpoint HbA1c levels
**Specify the numerical result being assessed.** In case of multiple alternative analyses being presented, specify the numeric result (e.g. RR = 1.52 (95% CI 0.83 to 2.77) and/or a reference (e.g. to a table, figure or paragraph) that uniquely defines the result being assessed.	Table 4; Domain 1.3: Table 1, 3, 4

Is the review team's aim for this result…?

X to assess the effect of *assignment to intervention* (the 'intention-to-treat' effect)
☐ to assess the effect of *adhering to intervention* (the 'per-protocol' effect)

If the aim is to assess the effect of *adhering to intervention*, select the deviations from intended intervention that should be addressed (at least one must be checked):

☐ occurrence of non-protocol interventions
☐ failures in implementing the intervention that could have affected the outcome
☐ non-adherence to their assigned intervention by trial participants

Which of the following sources were <u>obtained</u> to help inform the risk-of-bias assessment? (tick as many as apply)

X Journal article(s) with results of the trial
☐ Trial protocol
☐ Statistical analysis plan (SAP)

X	Non-commercial trial registry record (e.g. ClinicalTrials.gov record)
☐	Company-owned trial registry record (e.g. GSK Clinical Study Register record)
☐	“Grey literature” (e.g. unpublished thesis)
☐	Conference abstract(s) about the trial
☐	Regulatory document (e.g. Clinical Study Report, Drug Approval Package)
☐	Research ethics application
☐	Grant database summary (e.g. NIH RePORTER or Research Councils UK Gateway to Research)
☐	Personal communication with trialist
☐	Personal communication with the sponsor

Risk of bias assessment

Responses underlined in green are potential markers for low risk of bias, and responses in red are potential markers for a risk of bias. Where questions relate only to sign posts to other questions, no formatting is used.

Domain 1: Risk of bias arising from the randomization process

Signalling questions	Comments	Response options
**1.1 Was the allocation sequence random?**	Randomization was performed by software.	Y
**1.2 Was the allocation sequence concealed until participants were enrolled and assigned to interventions?**	The allocation sequence was concealed using anonymous drug vials and kept in sequentially numbered, sealed, and likely opaque envelope managed by a third party physician	Y
**1.3 Did baseline differences between intervention groups suggest a problem with the randomization process?**	Baseline values appear to be uniformly distributed.	N
**Risk-of-bias judgement**		Low
Optional: What is the predicted direction of bias arising from the randomization process?		NA

Domain 2: Risk of bias due to deviations from the intended interventions (*effect of assignment to intervention*)

Signalling questions	Comments	Response options
**2.1. Were participants aware of their assigned intervention during the trial?**	Participants were blinded to their treatments, and treatments were masked as described in Domain 1.2.	N
**2.2. Were carers and people delivering the interventions aware of participants' assigned intervention during the trial?**		N
**2.3. If Y/PY/NI to 2.1 or 2.2: Were there deviations from the intended intervention that arose because of the trial context?**		NA
**2.4 If Y/PY to 2.3: Were these deviations likely to have affected the outcome?**		NA
**2.5. If Y/PY/NI to 2.4: Were these deviations from intended intervention balanced between groups?**		NA
**2.6 Was an appropriate analysis used to estimate the effect of assignment to intervention?**	All participants were analyzed as long as they had relevant data available, making this a modified intention-to-treat analysis (Figure 1).	Y
**2.7 If N/PN/NI to 2.6: Was there potential for a substantial impact (on the result) of the failure to analyse participants in the group to which they were randomized?**		NA
**Risk-of-bias judgement**		Low
Optional: What is the predicted direction of bias due to deviations from intended interventions?		NA

Domain 3: Missing outcome data

Signalling questions	Comments	Response options
**3.1 Were data for this outcome available for all, or nearly all, participants randomized?**	Data was available for 55/59 (93%) of participants, which is likely sufficient for this continuous outcome.	Y
**3.2 If N/PN/NI to 3.1: Is there evidence that the result was not biased by missing outcome data?**		NA
**3.3 If N/PN to 3.2: Could missingness in the outcome depend on its true value?**		NA
**3.4 If Y/PY/NI to 3.3: Is it likely that missingness in the outcome depended on its true value?**		NA
**Risk-of-bias judgement**		Low
Optional: What is the predicted direction of bias due to missing outcome data?		NA

Domain 4: Risk of bias in measurement of the outcome

Signalling questions	Comments	Response options
**4.1 Was the method of measuring the outcome inappropriate?**	HbA1c measuring was very likely performed in a laboratory using standard, accurate techniques.	PN
**4.2 Could measurement or ascertainment of the outcome have differed between intervention groups?**	All HbA1c measuring was very likely to have been done at the same laboratory with the same methods of measurement.	PN
**4.3 If N/PN/NI to 4.1 and 4.2: Were outcome assessors aware of the intervention received by study participants?**	Laboratory values were very likely measured by a laboratory that was unaware of the intervention received.	PN
**4.4 If Y/PY/NI to 4.3: Could assessment of the outcome have been influenced by knowledge of intervention received?**		NA
**4.5 If Y/PY/NI to 4.4: Is it likely that assessment of the outcome was influenced by knowledge of intervention received?**		NA
**Risk-of-bias judgement**		Low
Optional: What is the predicted direction of bias in measurement of the outcome?		NA

Domain 5: Risk of bias in selection of the reported result

Signalling questions	Comments	Response options
**5.1 Were the data that produced this result analysed in accordance with a pre-specified analysis plan that was finalized before unblinded outcome data were available for analysis?**	Trial registry records created before outcome data shows analysis was preplanned. Though HbA1c measurements are not explicitly listed as outcome data, other aspects of records strongly suggest this was omitted in error.	PY
**Is the numerical result being assessed likely to have been selected, on the basis of the results, from...**		
**5.2. ... multiple eligible outcome measurements (e.g. scales, definitions, time points) within the outcome domain?**	Based on trial registry records, endpoint HbA1c outcomes were very likely only measured once at the end of the study, and variation of outcome measurement is unlikely.	PN
**5.3 ... multiple eligible analyses of the data?**	Both final value and change-from-baseline values are reported, and other eligible analyses are unlikely as data imputation was not used and data conversion was not performed, as usual.	PN
**Risk-of-bias judgement**		Some concerns
Optional: What is the predicted direction of bias due to selection of the reported result?		Unpredictable

Overall risk of bias

**Risk-of-bias judgement**		Some concerns
Optional: What is the overall predicted direction of bias for this outcome?		Unpredictable

This work is licensed under a Creative Commons Attribution-NonCommercial-NoDerivatives 4.0 International License.

Supplementary Fig. 48: Risk of Bias Analysis (Morano 2007)

Study details

**Reference**	Morano 2007[24]

Study design

X Individually-randomized parallel-group trial
☐ Cluster-randomized parallel-group trial
☐ Individually randomized cross-over (or other matched) trial

For the purposes of this assessment, the interventions being compared are defined as

Experimental: Sildenafil treatment   Comparator: Placebo treatment

Specify which outcome is being assessed for risk of bias: Endpoint HbA1c level

Specify the numerical result being assessed. In case of multiple alternative analyses being presented, specify the numeric result (e.g. RR = 1.52 (95% CI 0.83 to 2.77) and/or a reference (e.g. to a table, figure or paragraph) that uniquely defines the result being assessed.: Domain 1.3: Table 1, 2

Is the review team's aim for this result…?

X to assess the effect of *assignment to intervention* (the 'intention-to-treat' effect)
☐ to assess the effect of *adhering to intervention* (the 'per-protocol' effect)

If the aim is to assess the effect of *adhering to intervention*, select the deviations from intended intervention that should be addressed (at least one must be checked):

☐ occurrence of non-protocol interventions
☐ failures in implementing the intervention that could have affected the outcome
☐ non-adherence to their assigned intervention by trial participants

Which of the following sources were <u>obtained</u> to help inform the risk-of-bias assessment? (tick as many as apply)

X Journal article(s) with results of the trial
☐ Trial protocol

- ☐ Statistical analysis plan (SAP)
- ☐ Non-commercial trial registry record (e.g. ClinicalTrials.gov record)
- ☐ Company-owned trial registry record (e.g. GSK Clinical Study Register record)
- ☐ “Grey literature” (e.g. unpublished thesis)
- ☐ Conference abstract(s) about the trial
- ☐ Regulatory document (e.g. Clinical Study Report, Drug Approval Package)
- ☐ Research ethics application
- ☐ Grant database summary (e.g. NIH RePORTER or Research Councils UK Gateway to Research)
- ☐ Personal communication with trialist
- ☐ Personal communication with the sponsor

Risk of bias assessment

Responses underlined in green are potential markers for low risk of bias, and responses in red are potential markers for a risk of bias. Where questions relate only to sign posts to other questions, no formatting is used.

Domain 1: Risk of bias arising from the randomization process

Signalling questions	Comments	Response options
**1.1 Was the allocation sequence random?**	Participants were randomized via software.	Y
**1.2 Was the allocation sequence concealed until participants were enrolled and assigned to interventions?**	Although not directly specified, the trial is "double-blind" and complies with GCP/ICH guidelines, which ensures appropriate blinding.[25]	PY
**1.3 Did baseline differences between intervention groups suggest a problem with the randomization process?**	Baseline p-values appear to be approximately uniform when considering both standard deviation (SD) and standard error (SEM) statistics, since the trial does not specify when SD or SEM statistics are provided. One anomalous baseline value, "stimulation index", may be the result of a writing error when taking into account other data elsewhere in the paper.	PN
**Risk-of-bias judgement**		Low
Optional: What is the predicted direction of bias arising from the randomization process?		NA

Domain 2: Risk of bias due to deviations from the intended interventions (*effect of assignment to intervention*)

Signalling questions	Comments	Response options
**2.1. Were participants aware of their assigned intervention during the trial?**	Although not directly specified, the trial is "double-blind" and complies with GCP/ICH guidelines, which ensures appropriate blinding.	PN
**2.2. Were carers and people delivering the interventions aware of participants' assigned intervention during the trial?**		PN
**2.3. If Y/PY/NI to 2.1 or 2.2: Were there deviations from the intended intervention that arose because of the trial context?**		NA
**2.4 If Y/PY to 2.3: Were these deviations likely to have affected the outcome?**		NA
**2.5. If Y/PY/NI to 2.4: Were these deviations from intended intervention balanced between groups?**		NA
**2.6 Was an appropriate analysis used to estimate the effect of assignment to intervention?**	The number of people not included in analysis is not specified, but the small number of participants and the statement that all 32 participants were randomized (which other articles by the authors suggest is a number that includes all participants before any potential drop outs)[26,27] suggests that no participants dropped out and all were included in analysis, making this an intention-to-treat analysis.	PY
**2.7 If N/PN/NI to 2.6: Was there potential for a substantial impact (on the result) of the failure to analyse participants in the group to which they were randomized?**		NA
**Risk-of-bias judgement**		Low
Optional: What is the predicted direction of bias due to deviations from intended interventions?		NA

Domain 3: Missing outcome data

Signalling questions	Comments	Response options
**3.1 Were data for this outcome available for all, or nearly all, participants randomized?**	As in Domain 2.6, evidence suggests that data was available for all participants randomized.	PY
**3.2 If N/PN/NI to 3.1: Is there evidence that the result was not biased by missing outcome data?**		NA
**3.3 If N/PN to 3.2: Could missingness in the outcome depend on its true value?**		NA
**3.4 If Y/PY/NI to 3.3: Is it likely that missingness in the outcome depended on its true value?**		NA
**Risk-of-bias judgement**		Low
Optional: What is the predicted direction of bias due to missing outcome data?		NA

Domain 4: Risk of bias in measurement of the outcome

Signalling questions	Comments	Response options
**4.1 Was the method of measuring the outcome inappropriate?**	HPLC measurement of HbA1c was very likely performed in a laboratory using standard, accurate techniques.	PN
**4.2 Could measurement or ascertainment of the outcome have differed between intervention groups?**	All HbA1c measurement was very likely to have been done at the same laboratory with the same methods of measurement.	PN
**4.3 If N/PN/NI to 4.1 and 4.2: Were outcome assessors aware of the intervention received by study participants?**	The HPLC measurement of HbA1c references a city outside the location of the trial authors, suggesting a third party laboratory was used that is very likely unaware of the intervention received.	PN
**4.4 If Y/PY/NI to 4.3: Could assessment of the outcome have been influenced by knowledge of intervention received?**		NA
**4.5 If Y/PY/NI to 4.4: Is it likely that assessment of the outcome was influenced by knowledge of intervention received?**		NA
**Risk-of-bias judgement**		Low
Optional: What is the predicted direction of bias in measurement of the outcome?		NA

Domain 5: Risk of bias in selection of the reported result

Signalling questions	Comments	Response options
**5.1 Were the data that produced this result analysed in accordance with a pre-specified analysis plan that was finalized before unblinded outcome data were available for analysis?**	An analysis plan was not specified to have been prepared before data analysis.	NI
**Is the numerical result being assessed likely to have been selected, on the basis of the results, from...**		
**5.2. ... multiple eligible outcome measurements (e.g. scales, definitions, time points) within the outcome domain?**	All possible outcome measurements are reported, with explicit lack of data at other time points. Other ways to vary outcome measurements do not apply for this type of data.	PN
**5.3 ... multiple eligible analyses of the data?**	Standard analysis techniques are used.	PN
**Risk-of-bias judgement**		Some concerns
Optional: What is the predicted direction of bias due to selection of the reported result?		Unpredictable

Overall risk of bias

**Risk-of-bias judgement**		Some concerns
Optional: What is the overall predicted direction of bias for this outcome?		Unpredictable

This work is licensed under a Creative Commons Attribution-NonCommercial-NoDerivatives 4.0 International License.

Supplementary Fig. 49: Risk of Bias Analysis (Grover-Páez 2007)

Study details

**Reference**	Grover-Páez 2007[28]

Study design

X Individually-randomized parallel-group trial
☐ Cluster-randomized parallel-group trial
☐ Individually randomized cross-over (or other matched) trial

For the purposes of this assessment, the interventions being compared are defined as

Experimental: Sildenafil treatment Comparator: Placebo treatment

**Specify which outcome is being assessed for risk of bias**	Endpoint HbA1c level
**Specify the numerical result being assessed.** In case of multiple alternative analyses being presented, specify the numeric result (e.g. RR = 1.52 (95% CI 0.83 to 2.77) and/or a reference (e.g. to a table, figure or paragraph) that uniquely defines the result being assessed.	Figure 2; Domain 1.3: Table 1

Is the review team's aim for this result…?

X to assess the effect of *assignment to intervention* (the 'intention-to-treat' effect)
☐ to assess the effect of *adhering to intervention* (the 'per-protocol' effect)

If the aim is to assess the effect of *adhering to intervention*, select the deviations from intended intervention that should be addressed (at least one must be checked):

☐ occurrence of non-protocol interventions
☐ failures in implementing the intervention that could have affected the outcome
☐ non-adherence to their assigned intervention by trial participants

Which of the following sources were <u>obtained</u> to help inform the risk-of-bias assessment? (tick as many as apply)

X Journal article(s) with results of the trial
☐ Trial protocol

- ☐ Statistical analysis plan (SAP)
- ☐ Non-commercial trial registry record (e.g. ClinicalTrials.gov record)
- ☐ Company-owned trial registry record (e.g. GSK Clinical Study Register record)
- ☐ “Grey literature” (e.g. unpublished thesis)
- ☐ Conference abstract(s) about the trial
- ☐ Regulatory document (e.g. Clinical Study Report, Drug Approval Package)
- ☐ Research ethics application
- ☐ Grant database summary (e.g. NIH RePORTER or Research Councils UK Gateway to Research)
- ☐ Personal communication with trialist
- ☐ Personal communication with the sponsor

Risk of bias assessment

Responses underlined in green are potential markers for low risk of bias, and responses in red are potential markers for a risk of bias. Where questions relate only to sign posts to other questions, no formatting is used.

Domain 1: Risk of bias arising from the randomization process

Signalling questions	Comments	Response options
**1.1 Was the allocation sequence random?**	Subjects were assigned at random and with a concealed allocation sequence using closed-envelope selection.	Y
**1.2 Was the allocation sequence concealed until participants were enrolled and assigned to interventions?**		Y
**1.3 Did baseline differences between intervention groups suggest a problem with the randomization process?**	Baseline p-values appear uniformly distributed.	N
**Risk-of-bias judgement**		Low
Optional: What is the predicted direction of bias arising from the randomization process?		NA

Domain 2: Risk of bias due to deviations from the intended interventions (*effect of assignment to intervention*)

Signalling questions	Comments	Response options
**2.1. Were participants aware of their assigned intervention during the trial?**	The trial is specified to be "double-blind".	PN
**2.2. Were carers and people delivering the interventions aware of participants' assigned intervention during the trial?**		PN
**2.3. If Y/PY/NI to 2.1 or 2.2: Were there deviations from the intended intervention that arose because of the trial context?**		NA
**2.4 If Y/PY to 2.3: Were these deviations likely to have affected the outcome?**		NA
**2.5. If Y/PY/NI to 2.4: Were these deviations from intended intervention balanced between groups?**		NA
**2.6 Was an appropriate analysis used to estimate the effect of assignment to intervention?**	The sample size was precalculated as 20 participants per group, which was the number of participants in each group that were enrolled and had analysis reported, suggesting that all participants were reported, making this an intention-to-treat analysis.	PY
**2.7 If N/PN/NI to 2.6: Was there potential for a substantial impact (on the result) of the failure to analyse participants in the group to which they were randomized?**		NA
**Risk-of-bias judgement**		Low
Optional: What is the predicted direction of bias due to deviations from intended interventions?		NA

Domain 3: Missing outcome data

Signalling questions	Comments	Response options
**3.1 Were data for this outcome available for all, or nearly all, participants randomized?**	As in Domain 2.6, evidence in the trial suggests data is available for all participants randomized.	PY
**3.2 If N/PN/NI to 3.1: Is there evidence that the result was not biased by missing outcome data?**		NA
**3.3 If N/PN to 3.2: Could missingness in the outcome depend on its true value?**		NA
**3.4 If Y/PY/NI to 3.3: Is it likely that missingness in the outcome depended on its true value?**		NA
**Risk-of-bias judgement**		Low
Optional: What is the predicted direction of bias due to missing outcome data?		NA

Domain 4: Risk of bias in measurement of the outcome

Signalling questions	Comments	Response options
**4.1 Was the method of measuring the outcome inappropriate?**	A1c is specified to have been measured via venous blood sample, which was likely analyzed using standard techniques.	PN
**4.2 Could measurement or ascertainment of the outcome have differed between intervention groups?**	No differences in A1c measurement were specified, and it is very likely that both received the same laboratory measurement methods.	PN
**4.3 If N/PN/NI to 4.1 and 4.2: Were outcome assessors aware of the intervention received by study participants?**	HbA1c is likely to be measured with the same instrumentation at a laboratory that is likely unaware of the intervention received.	PN
**4.4 If Y/PY/NI to 4.3: Could assessment of the outcome have been influenced by knowledge of intervention received?**		NA
**4.5 If Y/PY/NI to 4.4: Is it likely that assessment of the outcome was influenced by knowledge of intervention received?**		NA
**Risk-of-bias judgement**		Low
Optional: What is the predicted direction of bias in measurement of the outcome?		NA

Domain 5: Risk of bias in selection of the reported result

Signalling questions	Comments	Response options
**5.1 Were the data that produced this result analysed in accordance with a pre-specified analysis plan that was finalized before unblinded outcome data were available for analysis?**	It is not stated whether a pre-specified analysis was prepared.	NI
**Is the numerical result being assessed likely to have been selected, on the basis of the results, from...**		
**5.2. ... multiple eligible outcome measurements (e.g. scales, definitions, time points) within the outcome domain?**	All participants were likely only measured at the beginning and end of the study, with other outcome measurement variation unlikely.	PN
**5.3 ... multiple eligible analyses of the data?**	Data is reported with standard forms of analyses.	PN
**Risk-of-bias judgement**		Some concerns
Optional: What is the predicted direction of bias due to selection of the reported result?		Unpredictable

Overall risk of bias

**Risk-of-bias judgement**		Some concerns
Optional: What is the overall predicted direction of bias for this outcome?		Unpredictable

This work is licensed under a Creative Commons Attribution-NonCommercial-NoDerivatives 4.0 International License.

Supplementary Fig. 50: Risk of Bias Analysis (Sáenz de Tejada 2002)

Study details

**Reference**	Sáenz de Tejada 2002[29]

Study design

X Individually-randomized parallel-group trial
☐ Cluster-randomized parallel-group trial
☐ Individually randomized cross-over (or other matched) trial

For the purposes of this assessment, the interventions being compared are defined as

Experimental: Tadalafil treatment Comparator: Placebo treatment

**Specify which outcome is being assessed for risk of bias**	Change of HbA1c concentration over period of treatment
**Specify the numerical result being assessed.** In case of multiple alternative analyses being presented, specify the numeric result (e.g. RR = 1.52 (95% CI 0.83 to 2.77) and/or a reference (e.g. to a table, figure or paragraph) that uniquely defines the result being assessed.	"Tadalafil treatment did not significantly change HbA1c levels from baseline to end point (0.2% both in the tadalafil 10- and 20-mg groups vs. 0% in the placebo group; P 0.083)" Sáenz de Tejada 2002, 2162)

Is the review team's aim for this result…?

X to assess the effect of *assignment to intervention* (the 'intention-to-treat' effect)
☐ to assess the effect of *adhering to intervention* (the 'per-protocol' effect)

If the aim is to assess the effect of *adhering to intervention*, select the deviations from intended intervention that should be addressed (at least one must be checked):

☐ occurrence of non-protocol interventions
☐ failures in implementing the intervention that could have affected the outcome
☐ non-adherence to their assigned intervention by trial participants

Which of the following sources were <u>obtained</u> to help inform the risk-of-bias assessment? (tick as many as apply)

X Journal article(s) with results of the trial
☐ Trial protocol

☐ Statistical analysis plan (SAP)
☐ Non-commercial trial registry record (e.g. ClinicalTrials.gov record)
☐ Company-owned trial registry record (e.g. GSK Clinical Study Register record)
☐ “Grey literature” (e.g. unpublished thesis)
☐ Conference abstract(s) about the trial
☐ Regulatory document (e.g. Clinical Study Report, Drug Approval Package)
☐ Research ethics application
☐ Grant database summary (e.g. NIH RePORTER or Research Councils UK Gateway to Research)
☐ Personal communication with trialist
☐ Personal communication with the sponsor

Risk of bias assessment

Responses underlined in green are potential markers for low risk of bias, and responses in red are potential markers for a risk of bias. Where questions relate only to sign posts to other questions, no formatting is used.

Domain 1: Risk of bias arising from the randomization process

Signalling questions	Comments	Response options
**1.1 Was the allocation sequence random?**	The study is called "randomized" (Sáenz de Tejada 2002, 2159). No further details on the method of randomization are given, but the corresponding author (Dr. Jeffrey T. Emmick) outlines in another paper (10.1111/j.1743-6109.2005.02043.x) that a "computer-generated randomization table" is used for randomization. Allocation sequence concealment is likely as the study is "double-blind".	PY
**1.2 Was the allocation sequence concealed until participants were enrolled and assigned to interventions?**		PY
**1.3 Did baseline differences between intervention groups suggest a problem with the randomization process?**	Baseline characteristics as in Table 1 appear compatible with random chance and do not suggest excessive similarity or dissimilarity	NI
**Risk-of-bias judgement**		Low
Optional: What is the predicted direction of bias arising from the randomization process?		NA

Domain 2: Risk of bias due to deviations from the intended interventions (*effect of assignment to intervention*)

Signalling questions	Comments	Response options
**2.1. Were participants aware of their assigned intervention during the trial?**	The study is "randomized, double-blind, placebo-controlled," so both carers and participants are likely unaware of the intervention.	PN
**2.2. Were carers and people delivering the interventions aware of participants' assigned intervention during the trial?**		PN
**2.3. If Y/PY/NI to 2.1 or 2.2: Were there deviations from the intended intervention that arose because of the trial context?**		NA
**2.4 If Y/PY to 2.3: Were these deviations likely to have affected the outcome?**		NA
**2.5. If Y/PY/NI to 2.4: Were these deviations from intended intervention balanced between groups?**		NA
**2.6 Was an appropriate analysis used to estimate the effect of assignment to intervention?**	An intention-to-treat analysis was done: "All analyses were performed with the participants included in the groups to which they were assigned by random allocation, even if the participant did not take the assigned treatment, did not receive the correct treatment, or otherwise did not follow the protocol."	Y
**2.7 If N/PN/NI to 2.6: Was there potential for a substantial impact (on the result) of the failure to analyse participants in the group to which they were randomized?**		NA
**Risk-of-bias judgement**		Low
Optional: What is the predicted direction of bias due to deviations from intended interventions?		NA

Domain 3: Missing outcome data

Signalling questions	Comments	Response options
**3.1 Were data for this outcome available for all, or nearly all, participants randomized?**	"All analyses were performed with the participants included in the groups to which they were assigned by random allocation, even if the participant did not take the assigned treatment, did not receive the correct treatment, or otherwise did not follow the protocol."	Y
**3.2 If N/PN/NI to 3.1: Is there evidence that the result was not biased by missing outcome data?**		NA
**3.3 If N/PN to 3.2: Could missingness in the outcome depend on its true value?**		NA
**3.4 If Y/PY/NI to 3.3: Is it likely that missingness in the outcome depended on its true value?**		NA
**Risk-of-bias judgement**		Low
Optional: What is the predicted direction of bias due to missing outcome data?		NA

Domain 4: Risk of bias in measurement of the outcome

Signalling questions	Comments	Response options
**4.1 Was the method of measuring the outcome inappropriate?**	The exact method of HbA1c level is not specified in the journal article but is likely done using routine, accurate measurements.	PN
**4.2 Could measurement or ascertainment of the outcome have differed between intervention groups?**	The exact method of HbA1c level is not specified in the journal article but is likely done in the same, routine manner for all intervention groups.	PN
**4.3 If N/PN/NI to 4.1 and 4.2: Were outcome assessors aware of the intervention received by study participants?**	The study is called "double-blind."	PN
**4.4 If Y/PY/NI to 4.3: Could assessment of the outcome have been influenced by knowledge of intervention received?**		NA
**4.5 If Y/PY/NI to 4.4: Is it likely that assessment of the outcome was influenced by knowledge of intervention received?**		NA
**Risk-of-bias judgement**		Low
Optional: What is the predicted direction of bias in measurement of the outcome?		NA

Domain 5: Risk of bias in selection of the reported result

Signalling questions	Comments	Response options
**5.1 Were the data that produced this result analysed in accordance with a pre-specified analysis plan that was finalized before unblinded outcome data were available for analysis?**	Though a statistical analysis plan is not provided, it is likely included in the study protocol: "The study protocol and informed consent form were reviewed and approved by ethical review boards."	PY
**Is the numerical result being assessed likely to have been selected, on the basis of the results, from...**		
**5.2. ... multiple eligible outcome measurements (e.g. scales, definitions, time points) within the outcome domain?**	There is only one way to measure HbA1c concentration, and there is only one time point at which it could be measured.	PN
**5.3 ... multiple eligible analyses of the data?**	No information on how the p-value was obtained, which is a value specifically used in this meta-analysis and may not have been specified in a protocol from this time period.	NI
**Risk-of-bias judgement**		Some concerns
Optional: What is the predicted direction of bias due to selection of the reported result?		Unpredictable

Overall risk of bias

**Risk-of-bias judgement**		Some concerns
Optional: What is the overall predicted direction of bias for this outcome?		Unpredictable

This work is licensed under a Creative Commons Attribution-NonCommercial-NoDerivatives 4.0 International License.

Supplementary Table 1: List of Study Search Results

The following tables return search results and the resulting fate of each search result. The database and date of search for each table were: **a)** PubMed on 2024-01-11 **b)** PMC Medline on 2024-01-13 **c)** PubMed on 2024-06-06 **d)** PMC Medline on 2024-06-06 **e)** Embase on 2024-09-01 **f)** Cochrane CENTRAL on 2024-09-28 **g)** CinicalTrials.gov on 2024-09-28 **h)** Embase on 2024-09-28 **i)** WHO ICTRP on 2024-09-28 **j)** PMC Medline on 2024-09-28 **k)** PubMed on 2024-09-28. Abbreviations: Haemoglobin A1c (HbA1c), Phosphodiesterase 5 (PDE5), Cochrane Central Register of Controlled Trials (Cochrane CENTRAL), World Health Organization International Clinical Trials Registry Platform (WHO ICTRP), PubMed Central (PMC), PubMed ID (PMID), Digital Object Identifier (DOI), Embase identification number (PUI), Uniform Resource Locator (URL).

a)

	DOI	PMID	STATUS
**1**	10.1016/j.eclinm.2023.101985		INCLUDED IN TRIAL
**2**		36305253	Not a clinical trial
**3**	10.1126/scitranslmed.abl8503		INCLUDED IN TRIAL
**4**	10.1186/s13098-022-00825-w		INCLUDED IN TRIAL
**5**	10.1152/ajpcell.00595.2020		drug but no a1c
**6**	10.1097/MJT.0000000000001303		Does not solely vary PDE5 inhibitor treatment
**7**	10.1111/andr.12919		PDE5 inhibitor and baseline HbA1c measured but endpoint HbA1c not measured
**8**	10.4103/aja.aja_121_19		PDE5 inhibitor and baseline HbA1c measured but endpoint HbA1c not measured
**9**	10.1016/j.jsxm.2019.01.003		PDE5 inhibitor and baseline HbA1c measured but endpoint HbA1c not measured
**10**	10.1016/j.jcte.2016.11.003		Not a clinical trial
**11**	10.1080/13685538.2016.1260107		Clinical trial but no PDE5 inhibitor
**12**	10.1002/ejhf.662		INCLUDED IN TRIAL
**13**	10.1016/j.jsxm.2016.04.075		PDE5 inhibitor and baseline HbA1c measured but endpoint HbA1c not measured
**14**	10.1517/14728222.2015.1066337		INCLUDED IN TRIAL
**15**	10.1038/ijir.2010.21		PDE5 inhibitor and baseline HbA1c measured but endpoint HbA1c not measured
**16**		20565373	Does not solely vary PDE5 inhibitor treatment
**17**	10.1038/ijir.2009.51		PDE5 inhibitor and baseline HbA1c measured but endpoint HbA1c not measured
**18**	10.2298/vsp0706399p		INCLUDED IN TRIAL
**19**	10.1016/j.eururo.2007.04.042		INCLUDED IN TRIAL
**20**	10.1016/j.diabres.2007.02.006		INCLUDED IN TRIAL
**21**	10.1111/j.1743-6109.2006.00295.x		PDE5 inhibitor and baseline HbA1c measured but endpoint HbA1c not measured
**22**	10.1111/j.1442-2042.2006.01480.x		PDE5 inhibitor and baseline HbA1c measured but endpoint HbA1c not measured
**23**	10.1016/j.fertnstert.2005.10.043		data PDE5 inhibitor and baseline HbA1c measured but endpoint HbA1c not measured
**24**	10.1007/s00125-004-1549-6		PDE5 inhibitor and baseline HbA1c measured but endpoint HbA1c not measured
**25**	10.13263/j.cnki.nja.2004.10.022	15562798	PDE5 inhibitor and baseline HbA1c measured but endpoint HbA1c not measured
**26**		14971333	abstract not found
**27**	10.2337/diacare.26.3.777		PDE5 inhibitor and baseline HbA1c measured but endpoint HbA1c not measured
**28**	10.2337/diacare.25.12.2159		INCLUDED IN TRIAL
**29**	10.1016/s0212-6567(02)79030-6		data PDE5 inhibitor and baseline HbA1c measured but endpoint HbA1c not measured
**30**	10.1007/s001250100656		PDE5 inhibitor and baseline HbA1c measured but endpoint HbA1c not measured

b)

	DOI	PMCID	STATUS
**1**	10.7759/cureus.50515		Not a clinical trial

**2**	10.1021/acsptsci.2c00082	9926524	Not a clinical trial
**3**	10.1016/j.jdiacomp.2022.108288	10783766	Not a clinical trial
**4**	10.21037/tau-23-71	10772644	Not a clinical trial
**5**	10.1210/endrev/bnad026	10765166	Not a clinical trial
**6**	10.3389/fendo.2023.1301093	10766371	Not a clinical trial
**7**	10.3390/diagnostics13243650	10743125	Not a clinical trial
**8**	10.3390/medicina59122190	10744870	Not a clinical trial
**9**	10.1038/s42255-023-00931-7	10730394	Not a clinical trial
**10**	10.1186/s12882-023-03427-4	10731818	Not a clinical trial
**11**	10.1016/j.heliyon.2023.e22482	10700708	Not a clinical trial
**12**	10.1152/japplphysiol.00478.2022	9762960	Clinical trial but no PDE5 inhibitor
**13**	10.1097/MD.0000000000035939	10637505	Clinical trial but no PDE5 inhibitor
**14**	10.1016/j.jceh.2022.03.012	9630008	Not a clinical trial
**15**	10.1002/jmri.28133	9411265	Clinical trial but no PDE5 inhibitor
**16**	10.2967/jnumed.122.264795	10152130	Clinical trial but no PDE5 inhibitor
**17**	10.1016/j.heliyon.2023.e21844	10661066	Not a clinical trial
**18**	10.3390/ijms242015078	10606418	Not a clinical trial
**19**	10.3389/fendo.2023.1238090	10600375	Clinical trial but no PDE5 inhibitor
**20**	10.1186/s12944-023-01950-9	10601238	Clinical trial but no PDE5 inhibitor
**21**	10.1186/s12969-023-00879-8	10580657	Clinical trial but no PDE5 inhibitor
**22**	10.1186/s40001-023-01413-y	10566198	Not a clinical trial
**23**	10.1097/CLD.0000000000000066	10550044	Not a clinical trial
**24**	10.7759/cureus.44576	10545003	Clinical trial but no PDE5 inhibitor
**25**	10.5114/aoms/135634	10508044	Clinical trial but no PDE5 inhibitor
**26**	10.1016/j.jacbts.2023.02.017	10504399	Clinical trial but no PDE5 inhibitor
**27**	10.7573/dic.2023-5-4	10499368	Clinical trial but no PDE5 inhibitor
**28**	10.2147/PPA.S412969	10493135	Clinical trial but no PDE5 inhibitor
**29**	10.3390/ijms241713541	10488183	Clinical trial but no PDE5 inhibitor
**30**	10.3390/ijms241713226	10488129	Not a clinical trial
**31**	10.25122/jml-2023-0068	10478652	PDE5 inhibitor and baseline HbA1c measured but endpoint HbA1c not measured
**32**	10.1016/j.jceh.2022.05.001	9499842	Clinical trial but no PDE5 inhibitor
**33**	10.1186/s12933-023-01956-8	10436534	Clinical trial but no PDE5 inhibitor
**34**	10.1210/clinem/dgad180	10438885	Clinical trial but no PDE5 inhibitor
**35**	10.1042/CS20220795	10415166	Not a clinical trial
**36**	10.1080/19585969.2022.2134739	10408697	Not a clinical trial
**37**	10.36628/ijhf.2023.0016	10406556	Not a clinical trial
**38**	10.4070/kcj.2023.0114	10406530	Not a clinical trial
**39**	10.18553/jmcp.2016.22.5.449	10398103	Clinical trial but no PDE5 inhibitor
**40**	10.1016/j.jsxm.2022.04.010	9329230	Clinical trial but no PDE5 inhibitor
**41**	10.1055/s-0041-1740936	9948071	Not a clinical trial
**42**	10.3389/fphar.2023.1184572	10367013	Not a clinical trial
**43**	10.1136/bcr-2020-241439	8311326	Clinical trial but no PDE5 inhibitor
**44**	10.1038/s41420-023-01553-4	10362058	Not a clinical trial
**45**	10.1038/s41419-023-05935-5	10336063	Not a clinical trial
**46**	10.2147/CIA.S405121	10337775	Clinical trial but no PDE5 inhibitor
**47**	10.1016/j.mad.2023.111818	10330534	Clinical trial but no PDE5 inhibitor
**48**	10.1016/j.ebiom.2023.104674	10328805	Not a clinical trial
**49**	10.1177/14791641231183634	10328035	Clinical trial but no PDE5 inhibitor

**50**	10.5534/wjmh.220057	10307658	PDE5 inhibitor and baseline HbA1c measured but endpoint HbA1c not measured
**51**	10.5534/wjmh.221027	10307648	Not a clinical trial
**52**	10.3390/life13061305	10301499	Not a clinical trial
**53**	10.3390/medicina59061119	10304508	Not a clinical trial
**54**	10.3390/biom13060930	10295993	Not a clinical trial
**55**	10.1038/s41419-023-05904-y	10293205	Not a clinical trial
**56**	10.3390/genes14061233	10297911	Clinical trial but no PDE5 inhibitor
**57**	10.1007/s11255-023-03602-4	10293434	Clinical trial but no PDE5 inhibitor
**58**	10.3389/fcvm.2023.1220000	10291612	Not a clinical trial
**59**	10.1186/s13098-023-01117-7	10288670	Clinical trial but no PDE5 inhibitor
**60**	10.1186/s12877-023-04100-z	10286414	Clinical trial but no PDE5 inhibitor
**61**	10.1002/pul2.12249	10271598	Clinical trial but no PDE5 inhibitor
**62**	10.1093/hropen/hoad023	10270320	Not a clinical trial
**63**	10.1016/j.metop.2023.100247	10267599	Not a clinical trial
**64**	10.1016/j.eclinm.2023.101985	10225663	Duplicate
**65**	10.1007/s11101-023-09869-w	10205037	Not a clinical trial
**66**	10.3390/jox13020017	10204391	Not a clinical trial
**67**	10.1007/s10741-023-10321-6	10185959	Not a clinical trial
**68**	10.1016/j.heliyon.2023.e15778	10176068	Not a clinical trial
**69**	10.5688/ajpe9171	10159487	Clinical trial but no PDE5 inhibitor
**70**	10.3389/fendo.2023.1135530	10151816	Not a clinical trial
**71**	10.1038/s41433-022-02007-4	9046155	Not a clinical trial
**72**	10.1161/CIRCRESAHA.122.319967	9060387	Not a clinical trial
**73**	10.3390/biology12040558	10135985	Not a clinical trial
**74**	10.3390/cancers15082316	10136828	Not a clinical trial
**75**	10.1007/s00508-023-02167-7	10133034	Not a clinical trial
**76**	10.1186/s12991-023-00447-0	10122283	Clinical trial but no PDE5 inhibitor
**77**	10.1111/andr.13328	10107754	Not a clinical trial
**78**	10.1002/hsr2.1167	10090802	Not a clinical trial
**79**	10.5588/ijtld.22.0514	10094053	Not a clinical trial
**80**	10.3389/fendo.2023.1149239	10086443	Not a clinical trial
**81**	10.1111/andr.13192	10084359	Not a clinical trial
**82**	10.2147/JPR.S397777	10069439	Clinical trial but no PDE5 inhibitor
**83**	10.1093/pcmedi/pbad005	10068425	Not a clinical trial
**84**	10.3390/ijms24065478	10058074	Not a clinical trial
**85**	10.1016/j.ebiom.2023.104506	10043778	Clinical trial but no PDE5 inhibitor
**86**	10.1016/j.jbc.2023.103059	10033317	Not a clinical trial
**87**	10.3389/fendo.2023.1134325	10028207	Not a clinical trial
**88**	10.5334/gh.1165	10000335	Clinical trial but no PDE5 inhibitor
**89**		9997681	Clinical trial but no PDE5 inhibitor
**90**	10.1001/jamacardio.2023.0065	9996460	Clinical trial but no PDE5 inhibitor
**91**	10.3389/fmed.2023.1110532	9971232	Not a clinical trial
**92**	10.3389/fonc.2023.1113462	9939513	Not a clinical trial
**93**	10.3389/fphar.2023.1033492	9939646	Not a clinical trial
**94**	10.21203/rs.3.rs-2475555/v1	9934781	Not a clinical trial
**95**	10.3390/healthcare11030433	9914730	Not a clinical trial
**96**	10.3390/jcm12030846	9917599	Not a clinical trial
**97**	10.1016/j.jacbts.2022.05.010	9911324	Not a clinical trial
**98**	10.21203/rs.3.rs-2515453/v1	9915771	Not a clinical trial
**99**	10.3389/fendo.2022.1036243	9902700	Clinical trial but no PDE5 inhibitor

**100**	10.1101/2023.01.25.525428	9900832	Not a clinical trial
**101**	10.1186/s12894-023-01180-2	9901095	Clinical trial but no PDE5 inhibitor
**102**	10.1002/pul2.12153	9748629	Does not solely vary PDE5 inhibitor treatment
**103**	10.3389/fendo.2023.1017886	9889556	Not a clinical trial
**104**	10.20517/jca.2022.42	9894375	Not a clinical trial
**105**	10.1186/s12967-022-03800-1	9887734	Clinical trial but no PDE5 inhibitor
**106**	10.34172/apb.2023.039	9871268	Clinical trial but no PDE5 inhibitor
**107**	10.1007/s40618-023-02015-5	9876440	Not a clinical trial
**108**	10.3390/jpm13010108	9862349	Clinical trial but no PDE5 inhibitor
**109**	10.6515/ACS.202301_39(1).20221103A	9829849	Not a clinical trial
**110**	10.1055/s-0041-1735209	9272316	Clinical trial but no PDE5 inhibitor
**111**	10.3389/fnagi.2022.899389	9831269	Clinical trial but no PDE5 inhibitor
**112**	10.14744/bej.2022.71676	9794510	Not a clinical trial
**113**	10.1038/s41392-022-01257-8	9797940	Not a clinical trial
**114**	10.1111/1753-0407.13334	9789395	Not a clinical trial
**115**	10.3389/fendo.2022.1032268	9767955	Not a clinical trial
**116**	10.1016/j.prnil.2022.06.004	9747574	Not a clinical trial
**117**	10.1007/s12026-022-09352-2	9760530	Not a clinical trial
**118**	10.1155/2022/5243594	9757934	Clinical trial but no PDE5 inhibitor
**119**	10.3389/fneur.2022.988825	9756760	Not a clinical trial
**120**	10.3390/jcm11236891	9737178	Not a clinical trial
**121**	10.3390/jcm11237214	9740756	Not a clinical trial
**122**	10.3389/fphar.2022.1057083	9731127	Not a clinical trial
**123**	10.1111/jdi.13903	9720202	Not a clinical trial
**124**	10.1111/jvim.16541	9708457	Not a clinical trial
**125**	10.3389/fnut.2022.930272	9691656	Clinical trial but no PDE5 inhibitor
**126**	10.1371/journal.pone.0276963	9668147	Clinical trial but no PDE5 inhibitor
**127**	10.21037/atm-22-3678	9652507	Not a clinical trial
**128**	10.4103/jfmpc.jfmpc_1130_21	9648286	Not a clinical trial
**129**	10.1007/s11910-022-01240-4	9663281	Not a clinical trial
**130**	10.7759/cureus.29699	9616173	Not a clinical trial
**131**	10.3390/jpm12101738	9604792	Not a clinical trial
**132**	10.1002/cpt.2627	9398938	Not a clinical trial
**133**	10.4183/aeb.2022.174	9512378	Not a clinical trial
**134**	10.1038/s41598-022-19364-5	9515177	Not a clinical trial
**135**	10.3390/nu14183737	9504067	Not a clinical trial
**136**	10.22038/IJBMS.2022.63378.13985	9464341	Not a clinical trial
**137**	10.4103/aja2021105	9491027	Clinical trial but no PDE5 inhibitor
**138**	10.1016/j.bjorl.2017.12.002	9449167	Not a clinical trial
**139**	10.1038/s41401-020-00557-5	8379181	Not a clinical trial
**140**	10.1111/1753-0407.13300	9426278	Clinical trial but no PDE5 inhibitor
**141**	10.1007/s11010-022-04520-2	9421626	Not a clinical trial
**142**	10.7759/cureus.27337	9414788	Not a clinical trial
**143**	10.3390/biomedicines10081848	9405076	Not a clinical trial
**144**	10.3390/life12081222	9410036	Not a clinical trial
**145**	10.1161/CIRCULATIONAHA.121.053889	8384699	Not a clinical trial
**146**	10.1186/s12967-022-03581-7	9389664	Clinical trial but no PDE5 inhibitor
**147**	10.1007/s00059-022-05123-9	9355932	Not a clinical trial
**148**	10.1111/andr.13169	9310719	Clinical trial but no PDE5 inhibitor
**149**	10.1038/s42003-022-03716-y	9314386	Not a clinical trial
**150**	10.3390/jcm11144027	9320223	Not a clinical trial

**151**	10.3390/jpm12071139	9319005	Clinical trial but no PDE5 inhibitor
**152**	10.1016/j.nerep.2022.100121	9314262	Not a clinical trial
**153**	10.3389/fphys.2022.906272	9304560	Not a clinical trial
**154**	10.1002/dmrr.3494	9286480	Not a clinical trial
**155**	10.1016/j.amsu.2022.104137	9283796	PDE5 inhibitor and baseline HbA1c measured but endpoint HbA1c not measured
**156**	10.1007/s00210-022-02249-9	9276575	Not a clinical trial
**157**	10.1038/s41392-022-01073-0	9259665	Not a clinical trial
**158**	10.1016/j.expneurol.2021.113694	8169562	Not a clinical trial
**159**	10.1007/s12325-022-02168-4	9239965	Clinical trial but no PDE5 inhibitor
**160**	10.1093/function/zqac029	9228651	Not a clinical trial
**161**	10.3390/jcm11123382	9224931	Clinical trial but no PDE5 inhibitor
**162**	10.1038/s41746-022-00617-6	9198008	Not a clinical trial
**163**	10.3390/jcm11113118	9181610	Not a clinical trial
**164**	10.1186/s13063-022-06296-8	9186476	Clinical trial but no PDE5 inhibitor
**165**	10.6515/ACS.202205_38(3).20220321A	9121756	Not a clinical trial
**166**	10.1016/j.jsxm.2021.03.077	8253516	Not a clinical trial
**167**	10.3390/diagnostics12051249	9141739	Not a clinical trial
**168**	10.4103/indianjpsychiatry.indianjpsychiatry_1014_21	9122155	Not a clinical trial
**169**	10.1186/s13014-022-02060-z	9115982	Not a clinical trial
**170**		9028527	Clinical trial but no PDE5 inhibitor
**171**	10.1001/jama.2021.18463	8596197	Clinical trial but no PDE5 inhibitor
**172**	10.3389/fimmu.2022.882032	9082262	Not a clinical trial
**173**	10.3389/fendo.2022.821113	9065269	Not a clinical trial
**174**	10.1111/dme.14276	9065795	Clinical trial but no PDE5 inhibitor
**175**		9045589	Clinical trial but no PDE5 inhibitor
**176**	10.18549/PharmPract.2022.1.2487	9014904	Clinical trial but no PDE5 inhibitor
**177**	10.1155/2022/9404025	9038412	Not a clinical trial
**178**	10.1371/journal.pone.0267047	9038205	Not a clinical trial
**179**	10.1097/MJT.0000000000001303	9035320	Duplicate
**180**	10.1186/s13098-022-00825-w	9022238	Duplicate
**181**	10.3389/fendo.2022.847240	9022207	INCLUDED IN TRIAL
**182**	10.23750/abm.v93i1.12910	8972891	Not a clinical trial
**183**	10.5534/wjmh.210021	8987149	Not a clinical trial
**184**	10.5534/wjmh.200157	8987134	PDE5 inhibitor and baseline HbA1c measured but endpoint HbA1c not measured
**185**	10.3390/ijms23073535	8998588	Not a clinical trial
**186**	10.21037/tau-22-58	8984969	Not a clinical trial
**187**	10.1177/2397198319898367	8922672	Clinical trial but no PDE5 inhibitor
**188**	10.1007/s40200-021-00782-7	8212202	Not a clinical trial
**189**	10.1016/j.jdiacomp.2020.107841	8007279	Not a clinical trial
**190**	10.1249/MSS.0000000000002521	7969358	Clinical trial but no PDE5 inhibitor
**191**	10.3390/biom12020278	8961612	Not a clinical trial
**192**	10.3390/jcm11061632	8956033	Not a clinical trial
**193**	10.3390/antiox11030580	8945168	Not a clinical trial
**194**	10.1016/j.esxm.2021.100477	8847829	Not a clinical trial
**195**	10.1155/2022/5583298	8791751	Not a clinical trial
**196**	10.5534/wjmh.200184	8761237	Not a clinical trial
**197**	10.5534/wjmh.200176	8761244	Not a clinical trial
**198**	10.1016/j.esxm.2021.100438	8766268	Not a clinical trial

**199**	10.1016/j.clnu.2021.11.030	8757535	Clinical trial but no PDE5 inhibitor
**200**	10.2215/CJN.08410520	7792638	Clinical trial but no PDE5 inhibitor
**201**	10.1002/ehf2.13590	8712918	Not a clinical trial
**202**	10.7759/cureus.19408	8654114	Not a clinical trial
**203**	10.1111/j.1751-7176.2010.00416.x	8108786	Clinical trial but no PDE5 inhibitor
**204**	10.12669/pjms.37.7.4257	8613049	Clinical trial but no PDE5 inhibitor
**205**	10.17245/jdapm.2021.21.6.479	8637910	Not a clinical trial
**206**	10.1186/s40001-021-00618-3	8655085	Clinical trial but no PDE5 inhibitor
**207**	10.1136/bcr-2019-232433	6904175	Not a clinical trial
**208**	10.1093/eurheartj/ehab389	8599003	Not a clinical trial
**209**	10.3389/fmed.2021.665023	8595206	Not a clinical trial
**210**	10.1007/s40618-021-01598-1	8572206	Clinical trial but no PDE5 inhibitor
**211**	10.4252/wjsc.v13.i10.1549	8567456	Not a clinical trial
**212**	10.1177/23969873211026698	8564163	PDE5 inhibitor and baseline HbA1c measured but endpoint HbA1c not measured
**213**	10.1186/s12913-021-07158-w	8540874	Not a clinical trial
**214**	10.3390/nu13103529	8541559	Not a clinical trial
**215**	10.3390/medicina57090868	8467670	Not a clinical trial
**216**	10.1177/2050640619854671	8454868	
**217**	10.21037/tau-21-441	8421832	PDE5 inhibitor and baseline HbA1c measured but endpoint HbA1c not measured
**218**	10.3390/ijms22179296	8431217	Clinical trial but no PDE5 inhibitor
**219**	10.1161/HYPERTENSIONAHA.120.14930	7429358	Clinical trial but no PDE5 inhibitor
**220**	10.1016/j.amsu.2021.102748	8387920	Clinical trial but no PDE5 inhibitor
**221**	10.1007/s42000-021-00295-1	8357658	Clinical trial but no PDE5 inhibitor
**222**	10.3390/ijms22168880	8396298	Clinical trial but no PDE5 inhibitor
**223**	10.3389/fphar.2021.518345	8381854	Clinical trial but no PDE5 inhibitor
**224**	10.1177/20458940211037274	8381443	Clinical trial but no PDE5 inhibitor
**225**	10.2147/JEP.S236743	8380049	Not a clinical trial
**226**	10.1177/20420188211034297	8365016	Clinical trial but no PDE5 inhibitor
**227**	10.3389/fcvm.2021.715400	8329089	Not a clinical trial
**228**	10.4239/wjd.v12.i7.954	8311479	Not a clinical trial
**229**	10.1186/s12931-021-01797-7	8314029	Not a clinical trial
**230**	10.36660/abc.20210180	8294740	Not a clinical trial
**231**	10.1007/s10067-020-05551-0	8289755	Clinical trial but no PDE5 inhibitor
**232**	10.1007/s11255-021-02867-x	8280019	Clinical trial but no PDE5 inhibitor
**233**	10.21037/atm-21-2479	8267313	Clinical trial but no PDE5 inhibitor
**234**	10.1007/s10822-021-00409-2	8273033	Not a clinical trial
**235**	10.1186/s12872-021-02146-8	8256614	Clinical trial but no PDE5 inhibitor
**236**	10.1186/s12902-021-00807-5	8252293	Clinical trial but no PDE5 inhibitor
**237**	10.1186/s12967-021-02935-x	8259336	Clinical trial but no PDE5 inhibitor
**238**	10.5534/wjmh.200109	8255402	Clinical trial but no PDE5 inhibitor
**239**	10.1111/dom.14322	8248154	Clinical trial but no PDE5 inhibitor
**240**	10.2196/25409	8218212	Clinical trial but no PDE5 inhibitor
**241**	10.1002/14651858.CD012787.pub2	8130994	Clinical trial but no PDE5 inhibitor
**242**	10.1038/s41443-020-0242-8	7483362	Clinical trial but no PDE5 inhibitor
**243**	10.1155/2021/6656406	8205584	Clinical trial but no PDE5 inhibitor
**244**	10.3390/biology10060540	8235660	Clinical trial but no PDE5 inhibitor
**245**	10.3390/nu13061784	8225153	Clinical trial but no PDE5 inhibitor
**246**		8221238	Clinical trial but no PDE5 inhibitor

**247**	10.3390/jcm10112501	8201035	Clinical trial but no PDE5 inhibitor
**248**	10.3390/ijms22115973	8198766	Clinical trial but no PDE5 inhibitor
**249**	10.7150/ijms.58147	8176183	Clinical trial but no PDE5 inhibitor
**250**	10.4103/aja.aja_71_20	8152419	Clinical trial but no PDE5 inhibitor
**251**	10.1152/ajpheart.00024.2020	7311696	Clinical trial but no PDE5 inhibitor
**252**	10.1161/ATVBAHA.119.313883	7255946	Clinical trial but no PDE5 inhibitor
**253**	10.3390/jcm10102221	8161068	Clinical trial but no PDE5 inhibitor
**254**	10.1136/bcr-2018-228872	6536206	Clinical trial but no PDE5 inhibitor
**255**	10.1007/s40618-020-01453-9	8124039	Clinical trial but no PDE5 inhibitor
**256**	10.1002/ehf2.13327	8120363	Clinical trial but no PDE5 inhibitor
**257**	10.3390/ijms22094666	8125634	Clinical trial but no PDE5 inhibitor
**258**	10.1002/ccr3.3946	8077334	Clinical trial but no PDE5 inhibitor
**259**	10.3389/fphar.2021.654489	8076853	Clinical trial but no PDE5 inhibitor
**260**	10.3390/ph14040365	8071249	drug but no a1c
**261**	10.2337/db19-0432	7085247	Clinical trial but no PDE5 inhibitor
**262**	10.1016/j.oret.2019.11.008	7150646	Clinical trial but no PDE5 inhibitor
**263**	10.3390/healthcare9030257	8000828	Not a clinical trial
**264**	10.1007/s40618-020-01381-8	7946690	Clinical trial but no PDE5 inhibitor
**265**	10.1007/s12664-020-01133-9	7972945	Not a clinical trial
**266**	10.1186/s12894-020-00730-2	7945372	Clinical trial but no PDE5 inhibitor
**267**	10.1172/jci.insight.146175	7934844	Clinical trial but no PDE5 inhibitor
**268**	10.1165/rcmb.2019-0226PS	6993553	Clinical trial but no PDE5 inhibitor
**269**	10.1038/s41569-020-0339-2	7849055	drug but no a1c
**270**	10.1167/tvst.10.1.13	7804520	Clinical trial but no PDE5 inhibitor
**271**	10.2147/CEOR.S285434	7814241	Clinical trial but no PDE5 inhibitor
**272**	10.1038/s41467-020-20500-w	7801461	Clinical trial but no PDE5 inhibitor
**273**	10.21037/tau-20-999	7807359	Clinical trial but no PDE5 inhibitor
**274**	10.1007/s13679-020-00422-w	7787121	Clinical trial but no PDE5 inhibitor
**275**	10.1007/s11845-020-02450-w	7788179	Not a clinical trial
**276**	10.1080/13543784.2020.1705277	6990416	Not a clinical trial
**277**	10.1111/bph.14920	7707100	Clinical trial but no PDE5 inhibitor
**278**	10.1016/S0212-6567(02)79030-6	7684223	Not a clinical trial
**279**	10.2478/jomb-2019-0043	7682853	Clinical trial but no PDE5 inhibitor
**280**	10.3390/molecules25225481	7700551	Clinical trial but no PDE5 inhibitor
**281**	10.3390/molecules25225318	7696151	Clinical trial but no PDE5 inhibitor
**282**	10.1177/2050640617725676	7672678	Not a clinical trial
**283**	10.3390/ijms21218244	7662747	Clinical trial but no PDE5 inhibitor
**284**	10.3390/pharmaceutics12090882	7558015	Clinical trial but no PDE5 inhibitor
**285**	10.1093/braincomms/fcaa020	7530832	Clinical trial but no PDE5 inhibitor
**286**	10.1002/jia2.25616	7533869	Not a clinical trial
**287**	10.1002/ehf2.12772	7524060	Clinical trial but no PDE5 inhibitor
**288**	10.1007/s12028-019-00710-x	6759381	Not a clinical trial
**289**	10.1016/j.neures.2018.10.005	6462258	Not a clinical trial
**290**	10.1016/j.ijcha.2020.100632	7502339	Clinical trial but no PDE5 inhibitor
**291**	10.1177/1179546820953415	7466888	Not a clinical trial
**292**	10.1038/s41598-020-71946-3	7484782	Clinical trial but no PDE5 inhibitor
**293**	10.3390/biom10081162	7464753	Clinical trial but no PDE5 inhibitor
**294**	10.3390/jcm9082513	7463896	Clinical trial but no PDE5 inhibitor
**295**	10.3390/ijms21155338	7432892	drug but no a1c
**296**	10.1007/s12551-020-00742-0	7429613	Not a clinical trial
**297**	10.4103/aja.aja_121_19	7406094	drug but no a1c
**298**	10.4103/aja.aja_106_19	7406095	Clinical trial but no PDE5 inhibitor

**299**	10.1155/2020/7078108	7407035	Clinical trial but no PDE5 inhibitor
**300**	10.3892/etm.2020.8934	7401651	drug but no a1c
**301**	10.18632/aging.103571	7377835	Clinical trial but no PDE5 inhibitor
**302**	10.1007/s00246-020-02408-w	7393337	Not a clinical trial
**303**	10.1016/j.ijcha.2020.100584	7385446	Clinical trial but no PDE5 inhibitor
**304**	10.1155/2020/9175676	7364200	Clinical trial but no PDE5 inhibitor
**305**	10.1186/s12874-020-01078-9	7382082	Clinical trial but no PDE5 inhibitor
**306**	10.3390/jcm9061995	7355625	Clinical trial but no PDE5 inhibitor
**307**	10.4103/ijem.IJEM_225_20	7328526	Not a clinical trial
**308**	10.1002/trc2.12050	7364858	Not a clinical trial
**309**	10.1155/2020/3293065	7345966	Not a clinical trial
**310**	10.5688/ajpe8220	7334344	Not a clinical trial
**311**	10.1152/japplphysiol.00947.2018	6692740	PDE5 inhibitor and baseline HbA1c measured but endpoint HbA1c not measured
**312**	10.3390/ijms21114020	7312670	Clinical trial but no PDE5 inhibitor
**313**	10.4103/aja.aja_15_19	7275806	Not a clinical trial
**314**	10.4103/ijmr.IJMR_502_20	7288773	Clinical trial but no PDE5 inhibitor
**315**	10.3390/biom10050752	7277861	Clinical trial but no PDE5 inhibitor
**316**	10.1038/s41366-019-0517-7	7260126	Clinical trial but no PDE5 inhibitor
**317**	10.1016/B978-3-437-21203-1.10002-0	7271212	Not a clinical trial
**318**	10.1016/B978-343721332-8.50004-1	7271219	Not a clinical trial
**319**	10.1016/B978-3-437-23246-6.10004-3	7271201	Not a clinical trial
**320**	10.1002/ehf2.12694	7261527	Clinical trial but no PDE5 inhibitor
**321**	10.1016/j.esxm.2020.02.006	7261691	Clinical trial but no PDE5 inhibitor
**322**	10.1016/j.esxm.2020.01.010	7261672	Clinical trial but no PDE5 inhibitor
**323**	10.1016/j.esxm.2020.01.006	7261708	Clinical trial but no PDE5 inhibitor
**324**	10.1002/14651858.CD012625.pub2	6535156	Clinical trial but no PDE5 inhibitor
**325**	10.3390/ijms21082703	7216146	Not a clinical trial
**326**	10.21037/tau.2020.02.07	7214985	Clinical trial but no PDE5 inhibitor
**327**	10.1097/HJH.0000000000001988	7223638	Clinical trial but no PDE5 inhibitor
**328**	10.15420/ecr.2019.14	7199190	Not a clinical trial
**329**	10.1210/jc.2019-00683	6735730	Not a clinical trial
**330**	10.1038/s41387-020-0116-7	7186220	Clinical trial but no PDE5 inhibitor
**331**	10.1007/978-3-642-33108-4_1	7176304	Not a clinical trial
**332**	10.1111/hiv.12513	7165664	Not a clinical trial
**333**	10.1111/jns.12225	7166380	Not a clinical trial
**334**	10.1002/ppul.23576	7159391	Not a clinical trial
**335**	10.1016/B978-0-323-52993-8.00048-5	7161392	Not a clinical trial
**336**	10.3390/foods9030340	7143620	Clinical trial but no PDE5 inhibitor
**337**	10.1016/B978-0-12-803206-0.00001-8	7149322	Not a clinical trial
**338**	10.1007/978-1-4419-6505-9_2	7139436	Not a clinical trial
**339**	10.1007/978-3-319-11821-5_2	7123375	Not a clinical trial
**340**	10.1007/978-1-60761-685-6_5	7123449	Not a clinical trial
**341**	10.1007/s12055-018-0649-8	7102261	Not a clinical trial
**342**	10.1007/s11096-008-9226-3	7102062	Not a clinical trial
**343**	10.1002/agm2.12097	7099759	Not a clinical trial
**344**	10.1038/s41409-019-0559-4	7091813	Not a clinical trial
**345**	10.1177/1753466620910092	7074506	Clinical trial but no PDE5 inhibitor
**346**	10.1155/2020/5214751	7064866	Clinical trial but no PDE5 inhibitor
**347**	10.7717/peerj.8653	7050549	Clinical trial but no PDE5 inhibitor
**348**	10.3389/fnins.2020.00140	7046549	Clinical trial but no PDE5 inhibitor

**349**	10.1177/0300060519859134	7045648	Clinical trial but no PDE5 inhibitor
**350**	10.1016/j.esxm.2019.08.012	7042165	drug but no a1c
**351**	10.1186/s13613-020-0623-7	7013036	Not a clinical trial
**352**	10.2169/internalmedicine.3372-19	7028403	Not a clinical trial
**353**	10.1093/gerona/glz056	6909887	Not a clinical trial
**354**	10.2169/internalmedicine.3625-19	7008044	Not a clinical trial
**355**	10.3390/ijms21010116	6982327	Not a clinical trial
**356**	10.1177/2042018819897527	6977225	Not a clinical trial
**357**	10.4103/aja.aja_108_19	6958978	Not a clinical trial
**358**	10.4103/aja.aja_92_19	6958986	Clinical trial but no PDE5 inhibitor
**359**	10.1111/jdi.13089	6944840	Clinical trial but no PDE5 inhibitor
**360**	10.1016/j.jacbts.2019.08.008	6939015	Clinical trial but no PDE5 inhibitor
**361**	10.1038/s41572-019-0087-y	6944317	Clinical trial but no PDE5 inhibitor
**362**	10.14744/nci.2018.23230	6936940	Clinical trial but no PDE5 inhibitor
**363**	10.5534/wjmh.180052M	6920065	Clinical trial but no PDE5 inhibitor
**364**	10.1016/j.heliyon.2019.e03035	6928307	drug but no a1c
**365**	10.3390/s19235311	6928990	Clinical trial but no PDE5 inhibitor
**366**	10.1136/bmjopen-2019-029098	6887087	Clinical trial but no PDE5 inhibitor
**367**	10.1002/14651858.CD010060.pub2	6517182	drug but no a1c
**368**	10.1186/s12882-019-1584-7	6820937	Clinical trial but no PDE5 inhibitor
**369**	10.1007/s11892-017-0917-9	6826336	Clinical trial but no PDE5 inhibitor
**370**	10.1177/2050640618792819	6796246	Not a clinical trial
**371**	10.17179/excli2019-1447	6785772	Clinical trial but no PDE5 inhibitor
**372**	10.5489/cuaj.6197	6752998	Clinical trial but no PDE5 inhibitor
**373**	10.2337/dc18-0255	6150428	Clinical trial but no PDE5 inhibitor
**374**	10.1152/physrev.00033.2017	6170978	Clinical trial but no PDE5 inhibitor
**375**	10.1371/journal.pone.0221992	6738611	Clinical trial but no PDE5 inhibitor
**376**	10.1002/14651858.CD002187.pub3	6718223	INCLUDED IN TRIAL
**377**	10.1016/j.esxm.2019.04.001	6728771	drug but no a1c
**378**	10.1186/s13104-019-4576-6	6720088	Not a clinical trial
**379**	10.1177/2040622319868376	6709440	Clinical trial but no PDE5 inhibitor
**380**	10.2337/dc17-2285	6105327	Clinical trial but no PDE5 inhibitor
**381**	10.1002/clc.21993	6652630	Clinical trial but no PDE5 inhibitor
**382**	10.1002/clc.4960271305	6654274	drug but no a1c
**383**	10.3390/jcm8071017	6678562	Clinical trial but no PDE5 inhibitor
**384**	10.3390/ijms20133299	6651183	Clinical trial but no PDE5 inhibitor
**385**	10.1177/1751143719835452	6661815	Not a clinical trial
**386**	10.15420/ecr.2019.13.1	6659039	Not a clinical trial
**387**	10.5114/ada.2019.83656	6640017	Not a clinical trial
**388**		6614614	Not a clinical trial
**389**	10.1002/edm2.64	6613223	Not a clinical trial
**390**	10.4111/icu.2019.60.4.275	6607068	Not a clinical trial
**391**	10.1186/s13063-019-3474-5	6588901	drug but no a1c
**392**	10.1007/s11302-018-9637-0	6339618	Clinical trial but no PDE5 inhibitor
**393**	10.2337/dc17-2510	6014549	Not a clinical trial
**394**	10.1016/j.ihj.2018.05.003	6097178	Clinical trial but no PDE5 inhibitor
**395**	10.1016/j.ihj.2018.01.002	6097164	Clinical trial but no PDE5 inhibitor
**396**	10.1002/cpt.979	6590078	Clinical trial but no PDE5 inhibitor
**397**	10.1371/journal.pone.0217690	6563988	Clinical trial but no PDE5 inhibitor
**398**	10.1002/14651858.CD009183.pub2	6540387	drug but no a1c
**399**	10.1007/s40618-018-0977-y	6531405	INCLUDED IN TRIAL
**400**	10.1136/ejhpharm-2016-001008	6451457	Clinical trial but no PDE5 inhibitor

**401**	10.1172/jci.insight.123618	6538324	Clinical trial but no PDE5 inhibitor
**402**	10.1016/j.esxm.2019.01.004	6522934	drug but no a1c
**403**	10.2196/12459	6483060	Clinical trial but no PDE5 inhibitor
**404**	10.5534/wjmh.180057	6479089	Not a clinical trial
**405**	10.1172/jci.insight.123611	6485674	Clinical trial but no PDE5 inhibitor
**406**	10.4103/aam.aam_3_18	6330780	Clinical trial but no PDE5 inhibitor
**407**	10.1002/14651858.CD008226.pub3	6464917	Not a clinical trial
**408**	10.1161/CIRCRESAHA.118.311912	5901903	Not a clinical trial
**409**	10.12688/f1000research.16561.1	6436191	Clinical trial but no PDE5 inhibitor
**410**	10.1186/s12933-019-0847-8	6432760	Clinical trial but no PDE5 inhibitor
**411**	10.1111/jvim.15372	6430903	Not a clinical trial
**412**	10.7555/JBR.31.20160164	6352876	Clinical trial but no PDE5 inhibitor
**413**	10.3399/bjgp18X695261	5819978	Not a clinical trial
**414**	10.12688/f1000research.17118.1	6381801	Clinical trial but no PDE5 inhibitor
**415**	10.1177/1559827617695219	6378503	Clinical trial but no PDE5 inhibitor
**416**	10.7861/clinmedicine.13-2-136	4952627	Clinical trial but no PDE5 inhibitor
**417**	10.7861/clinmedicine.13-1-63	5873712	Not a clinical trial
**418**	10.7861/clinmedicine.1-6-505	4953880	Not a clinical trial
**419**		6306027	Not a clinical trial
**420**		6306026	Not a clinical trial
**421**	10.3390/ijms19123942	6320923	Clinical trial but no PDE5 inhibitor
**422**	10.1038/aps.2017.77	5758669	Clinical trial but no PDE5 inhibitor
**423**	10.1111/bjh.15011	5847561	Clinical trial but no PDE5 inhibitor
**424**	10.5534/wjmh.180038	6305867	Clinical trial but no PDE5 inhibitor
**425**	10.5534/wjmh.180027	6305869	Clinical trial but no PDE5 inhibitor
**426**	10.2337/db16-1182	5697943	Not a clinical trial
**427**	10.21037/jtd.2018.09.74	6236195	Clinical trial but no PDE5 inhibitor
**428**	10.1097/MD.0000000000012559	6200524	drug but no a1c
**429**	10.4111/icu.2018.59.6.399	6215782	Clinical trial but no PDE5 inhibitor
**430**	10.1111/bph.13743	5660004	drug but no a1c
**431**	10.1016/j.jbi.2017.08.009	5705492	Not a clinical trial
**432**	10.1073/pnas.1809872115	6205494	Not a clinical trial
**433**	10.2147/DMSO.S172057	6181110	Clinical trial but no PDE5 inhibitor
**434**	10.1242/bio.036830	6176942	Not a clinical trial
**435**	10.1002/ehf2.12306	6165933	Not a clinical trial
**436**	10.1016/j.jsxm.2017.07.012	5624836	Clinical trial but no PDE5 inhibitor
**437**	10.1016/j.visres.2017.03.002	5660664	Clinical trial but no PDE5 inhibitor
**438**	10.1016/j.imr.2018.04.002	6160498	Clinical trial but no PDE5 inhibitor
**439**	10.1371/journal.pone.0202725	6108484	Not a clinical trial
**440**	10.5534/wjmh.180005	6119841	Not a clinical trial
**441**	10.1186/s12933-018-0763-3	6117983	Clinical trial but no PDE5 inhibitor
**442**	10.3390/nu10081126	6115838	Clinical trial but no PDE5 inhibitor
**443**	10.1152/ajprenal.00489.2016	6109798	Not a clinical trial
**444**	10.1002/jia2.25148	6062436	Not a clinical trial
**445**	10.1002/rth2.12125	6032109	Not a clinical trial
**446**	10.1007/s13300-018-0454-9	6028327	Clinical trial but no PDE5 inhibitor
**447**	10.1371/journal.pone.0199299	6023114	PDE5 inhibitor and baseline HbA1c measured but endpoint HbA1c not measured
**448**	10.1371/journal.pone.0199194	6014638	Not a clinical trial
**449**	10.1186/s12958-018-0378-2	6015465	Clinical trial but no PDE5 inhibitor
**450**	10.1111/apt.14674	6001629	Clinical trial but no PDE5 inhibitor

**451**	10.1007/s13300-018-0415-3	5984918	Clinical trial but no PDE5 inhibitor
**452**	10.1177/1557988315592026	5987948	Clinical trial but no PDE5 inhibitor
**453**	10.1038/s41598-018-24147-y	5940896	Not a clinical trial
**454**	10.1186/s12933-018-0704-1	5907287	Clinical trial but no PDE5 inhibitor
**455**	10.1038/s41598-018-24347-6	5897450	Clinical trial but no PDE5 inhibitor
**456**	10.1371/journal.pone.0194494	5882124	Clinical trial but no PDE5 inhibitor
**457**	10.1155/2018/9389784	5831709	Clinical trial but no PDE5 inhibitor
**458**	10.17925/EE.2015.11.02.81	5819072	Clinical trial but no PDE5 inhibitor
**459**	10.1007/s11606-017-4028-8	5391321	Clinical trial but no PDE5 inhibitor
**460**	10.1186/s12933-018-0688-x	5866526	Clinical trial but no PDE5 inhibitor
**461**	10.1093/cvr/cvx008	5852638	Not a clinical trial
**462**	10.1186/s12610-018-0068-0	5838858	Clinical trial but no PDE5 inhibitor
**463**	10.1007/s13410-018-0604-7	5838201	Clinical trial but no PDE5 inhibitor
**464**	10.1177/1557988316639050	5818109	Not a clinical trial
**465**	10.1111/jvim.14858	5787188	Not a clinical trial
**466**	10.3389/fcvm.2018.00001	5780411	Not a clinical trial
**467**	10.1513/AnnalsATS.201608-605OC	5427733	Clinical trial but no PDE5 inhibitor
**468**	10.1590/1414-431X20176601	5769756	drug but no a1c
**469**	10.4239/wjd.v9.i1.1	5763036	Not a clinical trial
**470**	10.18632/oncotarget.22389	5739671	Not a clinical trial
**471**	10.1177/2045893217743966	5731727	Clinical trial but no PDE5 inhibitor
**472**	10.1177/2045893217743616	5731720	drug but no a1c
**473**	10.1136/bmjopen-2016-015599	5729999	Clinical trial but no PDE5 inhibitor
**474**	10.21037/tau.2017.07.04	5715186	Not a clinical trial
**475**	10.1021/acs.jmedchem.6b00669	5564430	Not a clinical trial
**476**	10.1186/s10194-017-0817-z	5709272	Not a clinical trial
**477**	10.3390/nu9111273	5707745	Clinical trial but no PDE5 inhibitor
**478**	10.1259/bjr.20160366	5604913	Clinical trial but no PDE5 inhibitor
**479**	10.1111/ijcp.12995	5698762	Clinical trial but no PDE5 inhibitor
**480**	10.1016/j.esxm.2017.06.006	5693398	Not a clinical trial
**481**	10.1155/2017/4375253	5646336	Clinical trial but no PDE5 inhibitor
**482**	10.1681/ASN.2015050473	5084877	INCLUDED IN TRIAL
**483**	10.4103/jpp.JPP_42_17	5642133	Not a clinical trial
**484**	10.7759/cureus.1598	5652893	Not a clinical trial
**485**	10.1016/j.jcte.2016.11.003	5644434	INCLUDED IN TRIAL
**486**	10.1007/s13300-017-0313-0	5630567	Not a clinical trial
**487**	10.1111/1440-1681.12796	5601287	Clinical trial but no PDE5 inhibitor
**488**	10.1016/j.bbrep.2017.09.002	5613235	Not a clinical trial
**489**	10.4070/kcj.2017.0009	5614939	Clinical trial but no PDE5 inhibitor
**490**	10.1186/s12933-017-0595-6	5598064	Clinical trial but no PDE5 inhibitor
**491**	10.21037/tau.2017.07.19	5583047	Not a clinical trial
**492**	10.1155/2017/1253425	5574229	Clinical trial but no PDE5 inhibitor
**493**	10.1016/j.atherosclerosis.2016.07.921	5035618	Clinical trial but no PDE5 inhibitor
**494**	10.1186/s12933-017-0590-y	5577843	Clinical trial but no PDE5 inhibitor
**495**	10.1016/j.jsxm.2016.06.004	5333763	Not a clinical trial
**496**	10.1016/j.cgh.2015.11.021	4912904	Not a clinical trial
**497**	10.1210/jc.2016-1294	4929841	INCLUDED IN TRIAL
**498**	10.3389/fphar.2017.00363	5468794	Clinical trial but no PDE5 inhibitor
**499**	10.22038/IJBMS.2017.8690	5478785	Clinical trial but no PDE5 inhibitor
**500**	10.1016/j.jcmgh.2017.04.001	5472192	Not a clinical trial
**501**	10.1900/RDS.2015.12.63	5397984	Clinical trial but no PDE5 inhibitor
**502**	10.1186/s12969-017-0141-9	5461530	Not a clinical trial

**503**	10.1186/s12882-017-0553-2	5455080	Not a clinical trial
**504**	10.1016/j.jsxm.2016.04.064	5317031	Clinical trial but no PDE5 inhibitor
**505**	10.1007/s00125-017-4245-z	5423985	Clinical trial but no PDE5 inhibitor
**506**	10.1111/cts.12047	5350776	Not a clinical trial
**507**	10.3904/kjim.2016.208	5432803	Not a clinical trial
**508**	10.1016/j.jsxm.2016.02.168	4886867	Clinical trial but no PDE5 inhibitor
**509**	10.1186/s12890-017-0407-5	5405506	Clinical trial but no PDE5 inhibitor
**510**	10.3390/healthcare5010015	5371921	Clinical trial but no PDE5 inhibitor
**511**	10.7717/peerj.3020	5346286	Clinical trial but no PDE5 inhibitor
**512**	10.1371/journal.pone.0172751	5330475	drug but no a1c
**513**	10.7860/JCDR/2016/19971.8996	5296448	Clinical trial but no PDE5 inhibitor
**514**	10.3109/01658107.2013.817593	5291059	Clinical trial but no PDE5 inhibitor
**515**	10.1038/nrdp.2016.3	5027992	Not a clinical trial
**516**	10.1503/cmaj.151208	4674398	Not a clinical trial
**517**	10.1093/eurheartj/suv054	4700909	Clinical trial but no PDE5 inhibitor
**518**	10.1210/jc.2015-3415	4667163	drug but no a1c
**519**	10.6515/ACS20160611A	5126440	Not a clinical trial
**520**	10.1016/j.aju.2016.07.002	5122751	Clinical trial but no PDE5 inhibitor
**521**	10.1371/journal.pone.0165982	5089726	Not a clinical trial
**522**	10.1136/heartjnl-2015-309223	5099221	drug but no a1c
**523**	10.1371/journal.pone.0157915	5051725	PDE5 inhibitor and baseline HbA1c measured but endpoint HbA1c not measured
**524**	10.1093/eurheartj/ehv720	5074060	Not a clinical trial
**525**	10.1177/1535370214547155	4935182	drug but no a1c
**526**	10.1186/s40635-016-0099-9	5042923	Not a clinical trial
**527**		5010262	Not a clinical trial
**528**	10.1152/japplphysiol.00316.2015	4687862	Clinical trial but no PDE5 inhibitor
**529**	10.1016/j.esxm.2016.03.027	5005302	PDE5 inhibitor and baseline HbA1c measured but endpoint HbA1c not measured
**530**	10.1155/2016/9364861	4993942	Clinical trial but no PDE5 inhibitor
**531**	10.3390/ijms17081273	5000671	Not a clinical trial
**532**	10.5534/wjmh.2016.34.2.89	4999494	Not a clinical trial
**533**	10.1136/bcr-2014-205278	4154006	Not a clinical trial
**534**	10.1186/s13098-016-0159-z	4964290	Clinical trial but no PDE5 inhibitor
**535**	10.1007/s40256-016-0165-4	4947116	Not a clinical trial
**536**	10.1111/j.1743-6109.2008.01209.x	4951185	drug but no a1c
**537**	10.1007/s10753-016-0359-6	4883282	drug but no a1c
**538**	10.1186/s13020-016-0096-7	4864906	Not a clinical trial
**539**	10.7603/s40602-016-0003-6	4833805	Not a clinical trial
**540**	10.1177/1479164115621667	4834510	Clinical trial but no PDE5 inhibitor
**541**	10.1002/psp4.12049	4809625	Not a clinical trial
**542**	10.1161/CIRCULATIONAHA.114.013215	4390480	Clinical trial but no PDE5 inhibitor
**543**	10.1007/s11606-015-3271-0	4405523	Does not solely vary PDE5 inhibitor treatment
**544**	10.1111/jsm.12848	4390459	Clinical trial but no PDE5 inhibitor
**545**	10.1177/1756287215617648	4772354	Clinical trial but no PDE5 inhibitor
**546**	10.3389/fphar.2016.00050	4782109	Not a clinical trial
**547**	10.1177/2054270415622602	4776250	Clinical trial but no PDE5 inhibitor
**548**		4768436	Not a clinical trial
**549**		4755073	Clinical trial but no PDE5 inhibitor

**550**	10.1016/j.pharmthera.2014.10.003	4494657	Not a clinical trial
**551**	10.1002/14651858.CD006127.pub2	4439213	Clinical trial but no PDE5 inhibitor
**552**	10.2174/1573403X09666131117174414	4347210	Clinical trial but no PDE5 inhibitor
**553**	10.3978/j.issn.2223-4683.2012.07.03	4708248	Clinical trial but no PDE5 inhibitor
**554**	10.1002/sm2.91	4721034	Clinical trial but no PDE5 inhibitor
**555**	10.5114/aoms.2015.56342	4697050	Clinical trial but no PDE5 inhibitor
**556**	10.1111/odi.12275	4275405	drug but no a1c
**557**	10.1177/2050640615601623	4669991	Not a clinical trial
**558**	10.3109/13685538.2015.1004049	4648196	Not a clinical trial
**559**	10.4103/1008-682X.140966	4650459	Not a clinical trial
**560**	10.1186/s40200-015-0217-3	4665823	Clinical trial but no PDE5 inhibitor
**561**		4634342	Not a clinical trial
**562**	10.1155/2015/918069	4609427	Clinical trial but no PDE5 inhibitor
**563**	10.1093/eurheartj/ehu204	4204003	drug but no a1c
**564**	10.1007/s00439-015-1572-3	4607040	Clinical trial but no PDE5 inhibitor
**565**	10.1210/me.2014-1120	4179632	drug but no a1c
**566**	10.14814/phy2.12508	4562591	drug but no a1c
**567**	10.5152/tud.2013.97752	4548387	Clinical trial but no PDE5 inhibitor
**568**		4525223	Clinical trial but no PDE5 inhibitor
**569**	10.1186/s13098-015-0060-1	4546105	Clinical trial but no PDE5 inhibitor
**570**	10.2147/DDDT.S85676	4535549	Clinical trial but no PDE5 inhibitor
**571**	10.17795/ijpbs911	4525453	Not a clinical trial
**572**	10.4137/CMED.S27700	4509465	Clinical trial but no PDE5 inhibitor
**573**	10.1016/j.ophtha.2014.03.005	4122609	Clinical trial but no PDE5 inhibitor
**574**	10.1371/journal.pone.0133121	4505907	Clinical trial but no PDE5 inhibitor
**575**	10.3109/14767058.2012.717126	4511475	Not a clinical trial
**576**	10.1177/2051415813491862	4467226	Not a clinical trial
**577**	10.1111/j.1743-6109.2009.01458.x	4461030	Clinical trial but no PDE5 inhibitor
**578**	10.1007/s40620-014-0140-6	4439441	Clinical trial but no PDE5 inhibitor
**579**		4430881	Clinical trial but no PDE5 inhibitor
**580**	10.1161/CIRCULATIONAHA.113.001805	4053195	Not a clinical trial
**581**	10.1086/679705	4405710	Clinical trial but no PDE5 inhibitor
**582**	10.1007/s11606-014-2834-9	4429500	Not a clinical trial
**583**	10.1155/2015/548951	4415735	Clinical trial but no PDE5 inhibitor
**584**	10.1038/ijir.2014.14	4216643	Clinical trial but no PDE5 inhibitor
**585**	10.1186/s13063-015-0631-3	4411711	Not a clinical trial
**586**	10.1111/j.1582-4934.2008.00639.x	3822508	Not a clinical trial
**587**		4395914	Not a clinical trial
**588**	10.4111/kju.2015.56.4.310	4392031	Clinical trial but no PDE5 inhibitor
**589**	10.1155/2015/259592	4385644	Clinical trial but no PDE5 inhibitor
**590**	10.1007/s40268-015-0085-9	4359185	Not a clinical trial
**591**		4350889	Clinical trial but no PDE5 inhibitor
**592**	10.5489/cuaj.2731	4336025	Not a clinical trial
**593**	10.5489/cuaj.2699	4336024	Not a clinical trial
**594**	10.2147/DMSO.S71376	4334308	Clinical trial but no PDE5 inhibitor
**595**	10.1136/bcr-2012-007819	3604290	Not a clinical trial
**596**	10.1001/jamaophthalmol.2013.6326	4050640	Clinical trial but no PDE5 inhibitor
**597**	10.4239/wjd.v5.i6.905	4265879	Not a clinical trial
**598**	10.1136/bmjdrc-2013-000004	4212558	Clinical trial but no PDE5 inhibitor
**599**	10.1093/ehjci/jeu142	4240406	drug but no a1c
**600**	10.1371/journal.pone.0112394	4234367	Clinical trial but no PDE5 inhibitor
**601**	10.1177/2050640614548980	4212306	Not a clinical trial

**602**	10.1093/ajh/hpt098	3773573	Clinical trial but no PDE5 inhibitor
**603**	10.2337/dc13-0315	3781524	Clinical trial but no PDE5 inhibitor
**604**	10.2337/dc13-0294	3781490	Clinical trial but no PDE5 inhibitor
**605**	10.2147/TCRM.S57610	4155803	drug but no a1c
**606**	10.1111/bjh.12245	4129543	Not a clinical trial
**607**	10.1177/2042098611428486	4110829	Clinical trial but no PDE5 inhibitor
**608**	10.1177/2050640613502899	4070608	Not a clinical trial
**609**	10.1177/2050640613502900	4070603	Not a clinical trial
**610**	10.1093/eurheartj/eht497	3992428	Clinical trial but no PDE5 inhibitor
**611**	10.5489/cuaj.1962	4039601	Not a clinical trial
**612**	10.3892/etm.2014.1582	3991500	Clinical trial but no PDE5 inhibitor
**613**	10.4103/2230-8210.123552	4046605	Not a clinical trial
**614**		4021780	Not a clinical trial
**615**	10.1155/2014/653587	4009334	Not a clinical trial
**616**	10.1155/2014/143763	4000629	Not a clinical trial
**617**	10.1016/j.pharmthera.2008.05.005	4007052	Not a clinical trial
**618**	10.1111/bph.12143	3651669	drug but no a1c
**619**	10.1155/2014/878670	3976909	Not a clinical trial
**620**	10.3390/cells2020224	3972685	Not a clinical trial
**621**	10.5489/cuaj.1608	3956835	Not a clinical trial
**622**	10.2147/DMSO.S36455	3949699	Not a clinical trial
**623**	10.2147/PPA.S48357	3920925	Clinical trial but no PDE5 inhibitor
**624**	10.1097/MBP.0b013e328344c713	3915515	Clinical trial but no PDE5 inhibitor
**625**	10.1016/j.jash.2011.02.007	3915530	Not a clinical trial
**626**	10.4103/0975-7406.124317	3895294	Not a clinical trial
**627**	10.1155/2013/504915	3884863	Not a clinical trial
**628**	10.3205/cto000094	3884537	Not a clinical trial
**629**	10.1371/journal.pone.0083951	3877124	Clinical trial but no PDE5 inhibitor
**630**	10.1371/journal.pone.0085071	3873469	drug but no a1c
**631**	10.4111/kju.2013.54.12.858	3866290	PDE5 inhibitor and baseline HbA1c measured but endpoint HbA1c not measured
**632**	10.1089/wound.2012.0422	3817001	Not a clinical trial
**633**	10.4239/wjd.v4.i5.177	3797883	Not a clinical trial
**634**		3776489	Not a clinical trial
**635**	10.5534/wjmh.2013.31.2.83	3770856	Not a clinical trial
**636**	10.5534/wjmh.2013.31.2.103	3770846	Not a clinical trial
**637**	10.1038/aja.2010.123	3739608	Not a clinical trial
**638**	10.1186/1471-244X-13-214	3766216	Clinical trial but no PDE5 inhibitor
**639**	10.1016/j.juro.2012.04.001	3764461	Clinical trial but no PDE5 inhibitor
**640**	10.1155/2013/323574	3728554	Not a clinical trial
**641**	10.1007/s11606-011-1966-4	3378740	drug but no a1c
**642**	10.1097/HJH.0b013e32834000a7	3682653	drug but no a1c
**643**	10.1155/2013/763125	3665238	Not a clinical trial
**644**	10.1164/rccm.201111-2082CI	3373067	Not a clinical trial
**645**		3641729	Not a clinical trial
**646**	10.5534/wjmh.2012.30.2.114	3623519	Not a clinical trial
**647**	10.1007/s00125-011-2402-3	3329963	Clinical trial but no PDE5 inhibitor
**648**	10.1186/1758-5996-4-43	3568051	Not a clinical trial
**649**	10.1089/scd.2011.0303	3272247	Not a clinical trial
**650**		3565859	Clinical trial but no PDE5 inhibitor
**651**	10.1016/j.mayocp.2012.06.015	3498391	Not a clinical trial

**652**	10.1155/2012/836893	3521631	Not a clinical trial
**653**	10.1186/1471-2377-12-108	3517486	Not a clinical trial
**654**		3410114	Clinical trial but no PDE5 inhibitor
**655**	10.2147/VHRM.S26712	3433322	Not a clinical trial
**656**	10.1210/jc.2010-2724	3167667	Clinical trial but no PDE5 inhibitor
**657**		3413650	Not a clinical trial
**658**	10.2337/dc10-2339	3120209	Clinical trial but no PDE5 inhibitor
**659**		3351876	Not a clinical trial
**660**	10.1136/amiajnl-2011-000113	3078666	Clinical trial but no PDE5 inhibitor
**661**	10.1155/2012/569654	3303762	Not a clinical trial
**662**	10.1038/nutd.2011.6	3302138	Clinical trial but no PDE5 inhibitor
**663**	10.1186/1472-6904-12-5	3296596	Clinical trial but no PDE5 inhibitor
**664**	10.4111/kju.2011.52.11.725	3242984	drug but no a1c
**665**	10.1016/j.juro.2010.12.098	3220602	drug but no a1c
**666**		3206546	Not a clinical trial
**667**	10.1530/EJE-11-0221	3188848	Clinical trial but no PDE5 inhibitor
**668**	10.2119/molmed.2011.00100	3188861	Clinical trial but no PDE5 inhibitor
**669**	10.1186/1471-2261-11-36	3157429	Clinical trial but no PDE5 inhibitor
**670**	10.2147/JPR.S21751	3141833	Clinical trial but no PDE5 inhibitor
**671**		3048017	Not a clinical trial
**672**	10.1124/pr.109.002014	2835398	Not a clinical trial
**673**	10.2174/1874192401004010240	3026340	Not a clinical trial
**674**		3020279	Not a clinical trial
**675**	10.4065/mcp.2010.0164	2894722	Not a clinical trial
**676**		2094729	Not a clinical trial
**677**	10.1186/1475-2840-9-55	2954908	Not a clinical trial
**678**	10.1152/ajpendo.90996.2008	2763792	Not a clinical trial
**679**		2941787	Clinical trial but no PDE5 inhibitor
**680**	10.1007/s00125-010-1819-4	2931646	Clinical trial but no PDE5 inhibitor
**681**		2854169	Not a clinical trial
**682**		2911822	Clinical trial but no PDE5 inhibitor
**683**	10.1186/1472-6963-10-128	2893175	drug but no a1c
**684**	10.1186/1477-7827-8-50	2887879	Clinical trial but no PDE5 inhibitor
**685**		1913720	drug but no a1c
**686**		2849981	Clinical trial but no PDE5 inhibitor
**687**	10.2337/dc07-2375	2660483	Not a clinical trial
**688**	10.1007/s11606-009-0963-3	2670986	Not a clinical trial
**689**		2809989	Not a clinical trial
**690**		2801586	Not a clinical trial
**691**	10.1007/s00431-006-0349-z	2799065	Not a clinical trial
**692**	10.2174/157340308785160589	2780822	Not a clinical trial
**693**		2730071	Not a clinical trial
**694**	10.4103/0970-1591.52907	2710058	Not a clinical trial
**695**		2664585	Not a clinical trial
**696**		2699638	PDE5 inhibitor and baseline HbA1c measured but endpoint HbA1c not measured
**697**	10.3748/wjg.14.1564	2693753	PDE5 inhibitor and baseline HbA1c measured but endpoint HbA1c not measured
**698**		2686330	Not a clinical trial
**699**	10.4103/0970-1591.42612	2684375	Not a clinical trial

**700**		2544367	Not a clinical trial
**701**		1860735	PDE5 inhibitor and baseline HbA1c measured but endpoint HbA1c not measured
**702**	10.1186/1475-2840-8-19	2667490	Not a clinical trial
**703**	10.1186/1476-4598-7-82	2615789	Not a clinical trial
**704**		1853342	Not a clinical trial
**705**	10.1007/s00592-008-0030-2	2335289	Not a clinical trial
**706**		1994005	Not a clinical trial
**707**	10.1016/j.blre.2006.07.001	2048670	Not a clinical trial
**708**	10.1111/j.1525-1497.2006.00469.x	1484878	Not a clinical trial
**709**		1716227	Not a clinical trial
**710**		1502384	Not a clinical trial
**711**	10.1111/j.1525-1497.2005.020S1.x	1490295	Not a clinical trial
**712**		1495721	Not a clinical trial
**713**	10.1172/JCI27758	1359065	Not a clinical trial
**714**		1120492	Not a clinical trial
**715**		1117946	Not a clinical trial
**716**	10.1186/1475-2840-2-8	194431	PDE5 inhibitor and baseline HbA1c measured but endpoint HbA1c not measured
**717**	10.1177/00185787221115368	9445541	Not a clinical trial

c)

	**DOI**	**PMID**	**STATUS**
**1**	10.1016/j.eclinm.2023.101985	37256099	Duplicate
**2**		36305253	Duplicate
**3**	10.1111/and.14421	35301742	Not a clinical trial
**4**	10.1126/scitranslmed.abl8503	35704597	Duplicate
**5**	10.1186/s13098-022-00825-w	35449082	Duplicate
**6**	10.1152/ajpcell.00595.2020	33788629	Duplicate
**7**	10.1097/MJT.0000000000001303	33369909	Duplicate
**8**	10.1111/andr.12919	33022887	Duplicate
**9**	10.4103/aja.aja_121_19	31696836	Duplicate
**10**	10.1016/j.jsxm.2019.01.003	30773497	Duplicate
**11**	10.1080/13685538.2016.1260107	28084147	Duplicate
**12**	10.1002/ejhf.662	27873388	Duplicate
**13**	10.1016/j.jcte.2016.11.003	29067241	Duplicate
**14**	10.1016/j.jsxm.2016.04.075	27235284	Duplicate
**15**	10.1517/14728222.2015.1066337	26178526	Duplicate
**16**	10.1038/ijir.2010.21	20811390	Duplicate
**17**		20565373	Duplicate
**18**	10.1038/ijir.2009.51	19907424	Duplicate
**19**	10.1016/j.eururo.2007.04.042	17478034	Duplicate
**20**	10.1016/j.diabres.2007.02.006	17374416	Duplicate
**21**	10.2298/vsp0706399p	17687944	Duplicate
**22**	10.1111/j.1743-6109.2006.00295.x	16942532	Duplicate
**23**	10.1111/j.1442-2042.2006.01480.x	16903931	Duplicate
**24**	10.1016/j.fertnstert.2005.10.043	16579999	Duplicate
**25**	10.1007/s00125-004-1549-6	15599697	Duplicate

**26**		15562798	Duplicate
**27**		14971333	Duplicate
**28**	10.2337/diacare.26.3.777	12610037	Duplicate
**29**	10.2337/diacare.25.12.2159	12453954	Duplicate
**30**	10.1016/s0212-6567(02)79030-6	12372210	Duplicate
**31**	10.1007/s001250100656	11692178	Duplicate

d)

	**DOI**	**PMC ID**	**STATUS**
**1**	10.1007/s40618-023-02237-7	11142995	Not a clinical trial
**2**	10.1038/s41598-024-61287-w	11087471	Not a clinical trial
**3**	10.2147/JMDH.S455089	11075683	Not a clinical trial
**4**	10.5114/pm.2024.136327	11056728	Not a clinical trial
**5**	10.1007/s40259-024-00650-9	11055746	Not a clinical trial
**6**	10.3390/antiox13040455	11047699	Not a clinical trial
**7**	10.1016/S2213-8587(19)30405-X	11044807	Not a clinical trial
**8**	10.1080/14712598.2023.2203811	10330142	Not a clinical trial
**9**	10.1007/s10456-023-09903-7	11021332	Not a clinical trial
**10**	10.1002/mco2.516	11014467	Not a clinical trial
**11**	10.1093/europace/euae043	11000153	Not a clinical trial
**12**	10.3748/wjg.v30.i9.1073	10989500	Not a clinical trial
**13**	10.1159/000537829	10987180	Not a clinical trial
**14**		10775241	Not a clinical trial
**15**	10.1002/14651858.CD006127.pub3	10049880	Not a clinical trial
**16**	10.1016/j.heliyon.2024.e27206	10955197	Not a clinical trial
**17**	10.11817/j.issn.1672-7347.2023.220267	10930255	Not a clinical trial
**18**	10.4103/jhrs.JHRS_ISAR_IFS	10942128	Not a clinical trial
**19**	10.5935/1518-0557.20230071	10936909	Not a clinical trial
**20**	10.5213/inj.2346250.125	10932574	Not a clinical trial
**21**	10.1177/20420188241229540	10929063	Not a clinical trial
**22**	10.1148/radiol.220743	9968769	Not a clinical trial
**23**	10.22038/IJBMS.2023.73410.15950	10897555	Not a clinical trial
**24**	10.25122/jml-2023-0290	10893579	Not a clinical trial
**25**	10.1210/clinem/dgad100	10438886	Not a clinical trial
**26**	10.1210/clinem/dgad572	10876414	Not a clinical trial
**27**	10.1093/sexmed/qfae001	10849181	Not a clinical trial
**28**	10.4102/safp.v66i1.5822	10839213	Not a clinical trial
**29**	10.3390/ijms25020821	10815857	Not a clinical trial
**30**	10.14744/hf.2022.2022.0052	10809338	Not a clinical trial
**31**	10.1007/s11845-023-03412-8	10808673	Clinical trial but no PDE5 inhibitor
**32**	10.1136/bmjopen-2023-078325	10806598	Not a clinical trial
**33**	10.5114/pm.2023.133280	10793608	Not a clinical trial
**34**	10.5114/pm.2023.133883	10793607	Not a clinical trial
**35**	10.1016/j.cccb.2023.100199	10792690	Not a clinical trial
**36**	10.7759/cureus.50515	10787576	Duplicate
**37**	10.1021/acsptsci.2c00082	9926524	Duplicate
**38**	10.1016/j.jdiacomp.2022.108288	10783766	Duplicate
**39**	10.21037/tau-23-71	10772644	Duplicate
**40**	10.1210/endrev/bnad026	10765166	Duplicate

**41**	10.3389/fendo.2023.1301093	10766371	Duplicate
**42**	10.3390/diagnostics13243650	10743125	Duplicate
**43**	10.3390/medicina59122190	10744870	Duplicate
**44**	10.1038/s42255-023-00931-7	10730394	Duplicate
**45**	10.1186/s12882-023-03427-4	10731818	Duplicate
**46**	10.1016/j.heliyon.2023.e22482	10700708	Duplicate
**47**	10.1152/japplphysiol.00478.2022	9762960	Duplicate
**48**	10.1097/MD.0000000000035939	10637505	Duplicate
**49**	10.1016/j.jceh.2022.03.012	9630008	Duplicate
**50**	10.1002/jmri.28133	9411265	Duplicate
**51**	10.2967/jnumed.122.264795	10152130	Duplicate
**52**	10.1016/j.heliyon.2023.e21844	10661066	Duplicate
**53**	10.3390/ijms242015078	10606418	Duplicate
**54**	10.3389/fendo.2023.1238090	10600375	Duplicate
**55**	10.1186/s12944-023-01950-9	10601238	Duplicate
**56**	10.1186/s12969-023-00879-8	10580657	Duplicate
**57**	10.1186/s40001-023-01413-y	10566198	Duplicate
**58**	10.1097/CLD.0000000000000066	10550044	Duplicate
**59**	10.7759/cureus.44576	10545003	Duplicate
**60**	10.5114/aoms/135634	10508044	Duplicate
**61**	10.1016/j.jacbts.2023.02.017	10504399	Duplicate
**62**	10.7573/dic.2023-5-4	10499368	Duplicate
**63**	10.2147/PPA.S412969	10493135	Duplicate
**64**	10.3390/ijms241713541	10488183	Duplicate
**65**	10.3390/ijms241713226	10488129	Duplicate
**66**	10.25122/jml-2023-0068	10478652	Duplicate
**67**	10.1016/j.jceh.2022.05.001	9499842	Duplicate
**68**	10.1186/s12933-023-01956-8	10436534	Duplicate
**69**	10.1210/clinem/dgad180	10438885	Duplicate
**70**	10.1042/CS20220795	10415166	Duplicate
**71**	10.1080/19585969.2022.2134739	10408697	Duplicate
**72**	10.36628/ijhf.2023.0016	10406556	Duplicate
**73**	10.4070/kcj.2023.0114	10406530	Duplicate
**74**	10.18553/jmcp.2016.22.5.449	10398103	Duplicate
**75**	10.1016/j.jsxm.2022.04.010	9329230	Duplicate
**76**	10.1055/s-0041-1740936	9948071	Duplicate
**77**	10.3389/fphar.2023.1184572	10367013	Duplicate
**78**	10.1136/bcr-2020-241439	8311326	Duplicate
**79**	10.1038/s41420-023-01553-4	10362058	Duplicate
**80**	10.1038/s41419-023-05935-5	10336063	Duplicate
**81**	10.2147/CIA.S405121	10337775	Duplicate
**82**	10.1016/j.mad.2023.111818	10330534	Duplicate
**83**	10.1016/j.ebiom.2023.104674	10328805	Duplicate
**84**	10.1177/14791641231183634	10328035	Duplicate
**85**	10.5534/wjmh.220057	10307658	Duplicate
**86**	10.5534/wjmh.221027	10307648	Duplicate
**87**	10.3390/life13061305	10301499	Duplicate
**88**	10.3390/medicina59061119	10304508	Duplicate
**89**	10.3390/biom13060930	10295993	Duplicate
**90**	10.1038/s41419-023-05904-y	10293205	Duplicate
**91**	10.3390/genes14061233	10297911	Duplicate
**92**	10.1007/s11255-023-03602-4	10293434	Duplicate

**93**	10.3389/fcvm.2023.1220000	10291612	Duplicate
**94**	10.1186/s13098-023-01117-7	10288670	Duplicate
**95**	10.1186/s12877-023-04100-z	10286414	Duplicate
**96**	10.1002/pul2.12249	10271598	Duplicate
**97**	10.1093/hropen/hoad023	10270320	Duplicate
**98**	10.1016/j.metop.2023.100247	10267599	Duplicate
**99**	10.1016/j.eclinm.2023.101985	10225663	Duplicate
**100**	10.1007/s11101-023-09869-w	10205037	Duplicate
**101**	10.3390/jox13020017	10204391	Duplicate
**102**	10.1007/s10741-023-10321-6	10185959	Duplicate
**103**	10.1016/j.heliyon.2023.e15778	10176068	Duplicate
**104**	10.5688/ajpe9171	10159487	Duplicate
**105**	10.3389/fendo.2023.1135530	10151816	Duplicate
**106**	10.1038/s41433-022-02007-4	9046155	Duplicate
**107**	10.1161/CIRCRESAHA.122.319967	9060387	Duplicate
**108**	10.3390/biology12040558	10135985	Duplicate
**109**	10.3390/cancers15082316	10136828	Duplicate
**110**	10.1007/s00508-023-02167-7	10133034	Duplicate
**111**	10.1186/s12991-023-00447-0	10122283	Duplicate
**112**	10.4103/0970-1591.368611	10121074	Not a clinical trial
**113**	10.1111/andr.13328	10107754	Duplicate
**114**	10.1002/hsr2.1167	10090802	Duplicate
**115**	10.5588/ijtld.22.0514	10094053	Duplicate
**116**	10.3389/fendo.2023.1149239	10086443	Duplicate
**117**	10.1111/andr.13192	10084359	Duplicate
**118**	10.2147/JPR.S397777	10069439	Duplicate
**119**	10.1093/pcmedi/pbad005	10068425	Duplicate
**120**	10.3390/ijms24065478	10058074	Duplicate
**121**	10.1016/j.ebiom.2023.104506	10043778	Duplicate
**122**	10.1016/j.jbc.2023.103059	10033317	Duplicate
**123**	10.3389/fendo.2023.1134325	10028207	Duplicate
**124**	10.5334/gh.1165	10000335	Duplicate
**125**		9997681	Duplicate
**126**	10.1001/jamacardio.2023.0065	9996460	Duplicate
**127**	10.3389/fmed.2023.1110532	9971232	Duplicate
**128**	10.3389/fonc.2023.1113462	9939513	Duplicate
**129**	10.3389/fphar.2023.1033492	9939646	Duplicate
**130**	10.21203/rs.3.rs-2475555/v1	9934781	Duplicate
**131**	10.3390/healthcare11030433	9914730	Duplicate
**132**	10.3390/jcm12030846	9917599	Duplicate
**133**	10.1016/j.jacbts.2022.05.010	9911324	Duplicate
**134**	10.21203/rs.3.rs-2515453/v1	9915771	Duplicate
**135**	10.3389/fendo.2022.1036243	9902700	Duplicate
**136**	10.1101/2023.01.25.525428	9900832	Duplicate
**137**	10.1186/s12894-023-01180-2	9901095	Duplicate
**138**	10.1002/pul2.12153	9748629	Duplicate
**139**	10.3389/fendo.2023.1017886	9889556	Duplicate
**140**	10.20517/jca.2022.42	9894375	Duplicate
**141**	10.1186/s12967-022-03800-1	9887734	Duplicate
**142**	10.34172/apb.2023.039	9871268	Duplicate
**143**	10.1007/s40618-023-02015-5	9876440	Duplicate
**144**	10.3390/jpm13010108	9862349	Duplicate

**145**	10.6515/ACS.202301_39(1).20221103A	9829849	Duplicate
**146**	10.1055/s-0041-1735209	9272316	Duplicate
**147**	10.3389/fnagi.2022.899389	9831269	Duplicate
**148**	10.14744/bej.2022.71676	9794510	Duplicate
**149**	10.1038/s41392-022-01257-8	9797940	Duplicate
**150**	10.1111/1753-0407.13334	9789395	Duplicate
**151**	10.3389/fendo.2022.1032268	9767955	Duplicate
**152**	10.1016/j.prnil.2022.06.004	9747574	Duplicate
**153**	10.1007/s12026-022-09352-2	9760530	Duplicate
**154**	10.1155/2022/5243594	9757934	Duplicate
**155**	10.3389/fneur.2022.988825	9756760	Duplicate
**156**	10.3390/jcm11236891	9737178	Duplicate
**157**	10.3390/jcm11237214	9740756	Duplicate
**158**	10.3389/fphar.2022.1057083	9731127	Duplicate
**159**	10.1111/jdi.13903	9720202	Duplicate
**160**	10.1111/jvim.16541	9708457	Duplicate
**161**	10.3389/fnut.2022.930272	9691656	Duplicate
**162**	10.1371/journal.pone.0276963	9668147	Duplicate
**163**	10.21037/atm-22-3678	9652507	Duplicate
**164**	10.4103/jfmpc.jfmpc_1130_21	9648286	Duplicate
**165**	10.1007/s11910-022-01240-4	9663281	Duplicate
**166**	10.7759/cureus.29699	9616173	Duplicate
**167**	10.3390/jpm12101738	9604792	Duplicate
**168**	10.1002/cpt.2627	9398938	Duplicate
**169**	10.4183/aeb.2022.174	9512378	Duplicate
**170**	10.1038/s41598-022-19364-5	9515177	Duplicate
**171**	10.3390/nu14183737	9504067	Duplicate
**172**	10.22038/IJBMS.2022.63378.13985	9464341	Duplicate
**173**	10.4103/aja2021105	9491027	Duplicate
**174**	10.1016/j.bjorl.2017.12.002	9449167	Duplicate
**175**	10.1038/s41401-020-00557-5	8379181	Duplicate
**176**	10.1111/1753-0407.13300	9426278	Duplicate
**177**	10.1007/s11010-022-04520-2	9421626	Duplicate
**178**	10.7759/cureus.27337	9414788	Duplicate
**179**	10.3390/biomedicines10081848	9405076	Duplicate
**180**	10.3390/life12081222	9410036	Duplicate
**181**	10.1161/CIRCULATIONAHA.121.053889	8384699	Duplicate
**182**	10.1186/s12967-022-03581-7	9389664	Duplicate
**183**	10.1007/s00059-022-05123-9	9355932	Duplicate
**184**	10.1111/andr.13169	9310719	Duplicate
**185**	10.1038/s42003-022-03716-y	9314386	Duplicate
**186**	10.3390/jcm11144027	9320223	Duplicate
**187**	10.3390/jpm12071139	9319005	Duplicate
**188**	10.1016/j.nerep.2022.100121	9314262	Duplicate
**189**	10.3389/fphys.2022.906272	9304560	Duplicate
**190**	10.1002/dmrr.3494	9286480	Duplicate
**191**	10.1016/j.amsu.2022.104137	9283796	Duplicate
**192**	10.1007/s00210-022-02249-9	9276575	Duplicate
**193**	10.1038/s41392-022-01073-0	9259665	Duplicate
**194**	10.1016/j.expneurol.2021.113694	8169562	Duplicate
**195**	10.1007/s12325-022-02168-4	9239965	Duplicate
**196**	10.1093/function/zqac029	9228651	Duplicate

**197**	10.3390/jcm11123382	9224931	Duplicate
**198**	10.1038/s41746-022-00617-6	9198008	Duplicate
**199**	10.3390/jcm11113118	9181610	Duplicate
**200**	10.1186/s13063-022-06296-8	9186476	Duplicate
**201**	10.6515/ACS.202205_38(3).20220321A	9121756	Duplicate
**202**	10.1016/j.jsxm.2021.03.077	8253516	Duplicate
**203**	10.3390/diagnostics12051249	9141739	Duplicate
**204**	10.4103/indianjpsychiatry.indianjpsychiatry_1014_21	9122155	Duplicate
**205**	10.1186/s13014-022-02060-z	9115982	Duplicate
**206**		9028527	Duplicate
**207**	10.1001/jama.2021.18463	8596197	Duplicate
**208**	10.3389/fimmu.2022.882032	9082262	Duplicate
**209**	10.3389/fendo.2022.821113	9065269	Duplicate
**210**	10.1111/dme.14276	9065795	Duplicate
**211**		9045589	Duplicate
**212**	10.18549/PharmPract.2022.1.2487	9014904	Duplicate
**213**	10.1155/2022/9404025	9038412	Duplicate
**214**	10.1371/journal.pone.0267047	9038205	Duplicate
**215**	10.1097/MJT.0000000000001303	9035320	Duplicate
**216**	10.1186/s13098-022-00825-w	9022238	Duplicate
**217**	10.3389/fendo.2022.847240	9022207	Duplicate
**218**	10.23750/abm.v93i1.12910	8972891	Duplicate
**219**	10.5534/wjmh.210021	8987149	Duplicate
**220**	10.5534/wjmh.200157	8987134	Duplicate
**221**	10.3390/ijms23073535	8998588	Duplicate
**222**	10.21037/tau-22-58	8984969	Duplicate
**223**	10.1177/2397198319898367	8922672	Duplicate
**224**	10.1007/s40200-021-00782-7	8212202	Duplicate
**225**	10.1016/j.jdiacomp.2020.107841	8007279	Duplicate
**226**	10.1249/MSS.0000000000002521	7969358	Duplicate
**227**	10.3390/biom12020278	8961612	Duplicate
**228**	10.3390/jcm11061632	8956033	Duplicate
**229**	10.3390/antiox11030580	8945168	Duplicate
**230**	10.1016/j.esxm.2021.100477	8847829	Duplicate
**231**	10.1155/2022/5583298	8791751	Duplicate
**232**	10.5534/wjmh.200184	8761237	Duplicate
**233**	10.5534/wjmh.200176	8761244	Duplicate
**234**	10.1016/j.esxm.2021.100438	8766268	Duplicate
**235**	10.1016/j.clnu.2021.11.030	8757535	Duplicate
**236**	10.2215/CJN.08410520	7792638	Duplicate
**237**	10.1002/ehf2.13590	8712918	Duplicate
**238**	10.7759/cureus.19408	8654114	Duplicate
**239**	10.1111/j.1751-7176.2010.00416.x	8108786	Duplicate
**240**	10.12669/pjms.37.7.4257	8613049	Duplicate
**241**	10.17245/jdapm.2021.21.6.479	8637910	Duplicate
**242**	10.1186/s40001-021-00618-3	8655085	Duplicate
**243**	10.1136/bcr-2019-232433	6904175	Duplicate
**244**	10.1093/eurheartj/ehab389	8599003	Duplicate
**245**	10.3389/fmed.2021.665023	8595206	Duplicate
**246**	10.1007/s40618-021-01598-1	8572206	Duplicate
**247**	10.4252/wjsc.v13.i10.1549	8567456	Duplicate
**248**	10.1177/23969873211026698	8564163	Duplicate

**249**	10.1186/s12913-021-07158-w	8540874	Duplicate
**250**	10.3390/nu13103529	8541559	Duplicate
**251**	10.3390/medicina57090868	8467670	Duplicate
**252**	10.1177/2050640619854671	8454868	Duplicate
**253**	10.21037/tau-21-441	8421832	Duplicate
**254**	10.3390/ijms22179296	8431217	Duplicate
**255**	10.1161/HYPERTENSIONAHA.120.14930	7429358	Duplicate
**256**	10.1016/j.amsu.2021.102748	8387920	Duplicate
**257**	10.1007/s42000-021-00295-1	8357658	Duplicate
**258**	10.3390/ijms22168880	8396298	Duplicate
**259**	10.3389/fphar.2021.518345	8381854	Duplicate
**260**	10.1177/20458940211037274	8381443	Duplicate
**261**	10.2147/JEP.S236743	8380049	Duplicate
**262**	10.1177/20420188211034297	8365016	Duplicate
**263**	10.3389/fcvm.2021.715400	8329089	Duplicate
**264**	10.4239/wjd.v12.i7.954	8311479	Duplicate
**265**	10.1186/s12931-021-01797-7	8314029	Duplicate
**266**	10.36660/abc.20210180	8294740	Duplicate
**267**	10.1007/s10067-020-05551-0	8289755	Duplicate
**268**	10.1007/s11255-021-02867-x	8280019	Duplicate
**269**	10.21037/atm-21-2479	8267313	Duplicate
**270**	10.1007/s10822-021-00409-2	8273033	Duplicate
**271**	10.1186/s12872-021-02146-8	8256614	Duplicate
**272**	10.1186/s12902-021-00807-5	8252293	Duplicate
**273**	10.1186/s12967-021-02935-x	8259336	Duplicate
**274**	10.5534/wjmh.200109	8255402	Duplicate
**275**	10.1111/dom.14322	8248154	Duplicate
**276**	10.2196/25409	8218212	Duplicate
**277**	10.1002/14651858.CD012787.pub2	8130994	Duplicate
**278**	10.1038/s41443-020-0242-8	7483362	Duplicate
**279**	10.1155/2021/6656406	8205584	Duplicate
**280**	10.3390/biology10060540	8235660	Duplicate
**281**	10.3390/nu13061784	8225153	Duplicate
**282**		8221238	Duplicate
**283**	10.3390/jcm10112501	8201035	Duplicate
**284**	10.3390/ijms22115973	8198766	Duplicate
**285**	10.7150/ijms.58147	8176183	Duplicate
**286**	10.4103/aja.aja_71_20	8152419	Duplicate
**287**	10.1152/ajpheart.00024.2020	7311696	Duplicate
**288**	10.1161/ATVBAHA.119.313883	7255946	Duplicate
**289**	10.3390/jcm10102221	8161068	Duplicate
**290**	10.1136/bcr-2018-228872	6536206	Duplicate
**291**	10.1007/s40618-020-01453-9	8124039	Duplicate
**292**	10.1002/ehf2.13327	8120363	Duplicate
**293**	10.3390/ijms22094666	8125634	Duplicate
**294**	10.1002/ccr3.3946	8077334	Duplicate
**295**	10.3389/fphar.2021.654489	8076853	Duplicate
**296**	10.3390/ph14040365	8071249	Duplicate
**297**	10.2337/db19-0432	7085247	Duplicate
**298**	10.1016/j.oret.2019.11.008	7150646	Duplicate
**299**	10.3390/healthcare9030257	8000828	Duplicate
**300**	10.1007/s40618-020-01381-8	7946690	Duplicate

**301**	10.1007/s12664-020-01133-9	7972945	Duplicate
**302**	10.1186/s12894-020-00730-2	7945372	Duplicate
**303**	10.1172/jci.insight.146175	7934844	Duplicate
**304**	10.1165/rcmb.2019-0226PS	6993553	Duplicate
**305**	10.1038/s41569-020-0339-2	7849055	Duplicate
**306**	10.1167/tvst.10.1.13	7804520	Duplicate
**307**	10.2147/CEOR.S285434	7814241	Duplicate
**308**	10.1038/s41467-020-20500-w	7801461	Duplicate
**309**	10.21037/tau-20-999	7807359	Duplicate
**310**	10.1007/s13679-020-00422-w	7787121	Duplicate
**311**	10.1007/s11845-020-02450-w	7788179	Duplicate
**312**	10.1080/13543784.2020.1705277	6990416	Duplicate
**313**	10.1111/bph.14920	7707100	Duplicate
**314**	10.1016/S0212-6567(02)79030-6	7684223	Duplicate
**315**	10.2478/jomb-2019-0043	7682853	Duplicate
**316**	10.3390/molecules25225481	7700551	Duplicate
**317**	10.3390/molecules25225318	7696151	Duplicate
**318**	10.1177/2050640617725676	7672678	Duplicate
**319**	10.3390/ijms21218244	7662747	Duplicate
**320**	10.3390/pharmaceutics12090882	7558015	Duplicate
**321**	10.1093/braincomms/fcaa020	7530832	Duplicate
**322**	10.1002/jia2.25616	7533869	Duplicate
**323**	10.1002/ehf2.12772	7524060	Duplicate
**324**	10.1007/s12028-019-00710-x	6759381	Duplicate
**325**	10.1016/j.neures.2018.10.005	6462258	Duplicate
**326**	10.1016/j.ijcha.2020.100632	7502339	Duplicate
**327**	10.1177/1179546820953415	7466888	Duplicate
**328**	10.1038/s41598-020-71946-3	7484782	Duplicate
**329**	10.3390/biom10081162	7464753	Duplicate
**330**	10.3390/jcm9082513	7463896	Duplicate
**331**	10.3390/ijms21155338	7432892	Duplicate
**332**	10.1007/s12551-020-00742-0	7429613	Duplicate
**333**	10.4103/aja.aja_121_19	7406094	Duplicate
**334**	10.4103/aja.aja_106_19	7406095	Duplicate
**335**	10.1155/2020/7078108	7407035	Duplicate
**336**	10.3892/etm.2020.8934	7401651	Duplicate
**337**	10.18632/aging.103571	7377835	Duplicate
**338**	10.1007/s00246-020-02408-w	7393337	Duplicate
**339**	10.1016/j.ijcha.2020.100584	7385446	Duplicate
**340**	10.1155/2020/9175676	7364200	Duplicate
**341**	10.1186/s12874-020-01078-9	7382082	Duplicate
**342**	10.3390/jcm9061995	7355625	Duplicate
**343**	10.4103/ijem.IJEM_225_20	7328526	Duplicate
**344**	10.1002/trc2.12050	7364858	Duplicate
**345**	10.1155/2020/3293065	7345966	Duplicate
**346**	10.5688/ajpe8220	7334344	Duplicate
**347**	10.1152/japplphysiol.00947.2018	6692740	Duplicate
**348**	10.3390/ijms21114020	7312670	Duplicate
**349**	10.4103/aja.aja_15_19	7275806	Duplicate
**350**	10.4103/ijmr.IJMR_502_20	7288773	Duplicate
**351**	10.3390/biom10050752	7277861	Duplicate
**352**	10.1038/s41366-019-0517-7	7260126	Duplicate

**353**	10.1016/B978-3-437-21203-1.10002-0	7271212	Duplicate
**354**	10.1016/B978-343721332-8.50004-1	7271219	Duplicate
**355**	10.1016/B978-3-437-23246-6.10004-3	7271201	Duplicate
**356**	10.1002/ehf2.12694	7261527	Duplicate
**357**	10.1016/j.esxm.2020.02.006	7261691	Duplicate
**358**	10.1016/j.esxm.2020.01.010	7261672	Duplicate
**359**	10.1016/j.esxm.2020.01.006	7261708	Duplicate
**360**	10.1002/14651858.CD012625.pub2	6535156	Duplicate
**361**	10.3390/ijms21082703	7216146	Duplicate
**362**	10.21037/tau.2020.02.07	7214985	Duplicate
**363**	10.1097/HJH.0000000000001988	7223638	Duplicate
**364**	10.15420/ecr.2019.14	7199190	Duplicate
**365**	10.1210/jc.2019-00683	6735730	Duplicate
**366**	10.1038/s41387-020-0116-7	7186220	Duplicate
**367**	10.1007/978-3-642-33108-4_1	7176304	Duplicate
**368**	10.1111/hiv.12513	7165664	Duplicate
**369**	10.1111/jns.12225	7166380	Duplicate
**370**	10.1002/ppul.23576	7159391	Duplicate
**371**	10.1016/B978-0-323-52993-8.00048-5	7161392	Duplicate
**372**	10.1016/B978-0-12-803206-0.00001-8	7149322	Duplicate
**373**	10.1007/978-1-4419-6505-9_2	7139436	Duplicate
**374**	10.1007/978-3-319-11821-5_2	7123375	Duplicate
**375**	10.1007/978-1-60761-685-6_5	7123449	Duplicate
**376**	10.1007/s12055-018-0649-8	7102261	Duplicate
**377**	10.1007/s11096-008-9226-3	7102062	Duplicate
**378**	10.1002/agm2.12097	7099759	Duplicate
**379**	10.1038/s41409-019-0559-4	7091813	Duplicate
**380**	10.1177/1753466620910092	7074506	Duplicate
**381**	10.1155/2020/5214751	7064866	Duplicate
**382**	10.7717/peerj.8653	7050549	Duplicate
**383**	10.3389/fnins.2020.00140	7046549	Duplicate
**384**	10.1177/0300060519859134	7045648	Duplicate
**385**	10.1016/j.esxm.2019.08.012	7042165	Duplicate
**386**	10.1186/s13613-020-0623-7	7013036	Duplicate
**387**	10.2169/internalmedicine.3372-19	7028403	Duplicate
**388**	10.1093/gerona/glz056	6909887	Duplicate
**389**	10.2169/internalmedicine.3625-19	7008044	Duplicate
**390**	10.3390/ijms21010116	6982327	Duplicate
**391**	10.1177/2042018819897527	6977225	Duplicate
**392**	10.4103/aja.aja_108_19	6958978	Duplicate
**393**	10.4103/aja.aja_92_19	6958986	Duplicate
**394**	10.1111/jdi.13089	6944840	Duplicate
**395**	10.1016/j.jacbts.2019.08.008	6939015	Duplicate
**396**	10.1038/s41572-019-0087-y	6944317	Duplicate
**397**	10.14744/nci.2018.23230	6936940	Duplicate
**398**	10.5534/wjmh.180052M	6920065	Duplicate
**399**	10.1016/j.heliyon.2019.e03035	6928307	Duplicate
**400**	10.3390/s19235311	6928990	Duplicate
**401**	10.1136/bmjopen-2019-029098	6887087	Duplicate
**402**	10.1002/14651858.CD010060.pub2	6517182	Duplicate
**403**	10.1186/s12882-019-1584-7	6820937	Duplicate
**404**	10.1007/s11892-017-0917-9	6826336	Duplicate

**405**	10.1177/2050640618792819	6796246	Duplicate
**406**	10.17179/excli2019-1447	6785772	Duplicate
**407**	10.5489/cuaj.6197	6752998	Duplicate
**408**	10.2337/dc18-0255	6150428	Duplicate
**409**	10.1152/physrev.00033.2017	6170978	Duplicate
**410**	10.1371/journal.pone.0221992	6738611	Duplicate
**411**	10.1002/14651858.CD002187.pub3	6718223	Duplicate
**412**	10.1016/j.esxm.2019.04.001	6728771	Duplicate
**413**	10.1186/s13104-019-4576-6	6720088	Duplicate
**414**	10.1177/2040622319868376	6709440	Duplicate
**415**	10.2337/dc17-2285	6105327	Duplicate
**416**	10.1002/clc.21993	6652630	Duplicate
**417**	10.1002/clc.4960271305	6654274	Duplicate
**418**	10.3390/jcm8071017	6678562	Duplicate
**419**	10.3390/ijms20133299	6651183	Duplicate
**420**	10.1177/1751143719835452	6661815	Duplicate
**421**	10.15420/ecr.2019.13.1	6659039	Duplicate
**422**	10.5114/ada.2019.83656	6640017	Duplicate
**423**		6614614	Duplicate
**424**	10.1002/edm2.64	6613223	Duplicate
**425**	10.4111/icu.2019.60.4.275	6607068	Duplicate
**426**	10.1186/s13063-019-3474-5	6588901	Duplicate
**427**	10.1007/s11302-018-9637-0	6339618	Duplicate
**428**	10.2337/dc17-2510	6014549	Duplicate
**429**	10.1016/j.ihj.2018.05.003	6097178	Duplicate
**430**	10.1016/j.ihj.2018.01.002	6097164	Duplicate
**431**	10.1002/cpt.979	6590078	Duplicate
**432**	10.1371/journal.pone.0217690	6563988	Duplicate
**433**	10.1002/14651858.CD009183.pub2	6540387	Duplicate
**434**	10.1007/s40618-018-0977-y	6531405	Duplicate
**435**	10.1136/ejhpharm-2016-001008	6451457	Duplicate
**436**	10.1172/jci.insight.123618	6538324	Duplicate
**437**	10.1016/j.esxm.2019.01.004	6522934	Duplicate
**438**	10.2196/12459	6483060	Duplicate
**439**	10.5534/wjmh.180057	6479089	Duplicate
**440**	10.1172/jci.insight.123611	6485674	Duplicate
**441**	10.4103/aam.aam_3_18	6330780	Duplicate
**442**	10.1002/14651858.CD008226.pub3	6464917	Duplicate
**443**	10.1161/CIRCRESAHA.118.311912	5901903	Duplicate
**444**	10.12688/f1000research.16561.1	6436191	Duplicate
**445**	10.1186/s12933-019-0847-8	6432760	Duplicate
**446**	10.1111/jvim.15372	6430903	Duplicate
**447**	10.7555/JBR.31.20160164	6352876	Duplicate
**448**	10.3399/bjgp18X695261	5819978	Duplicate
**449**	10.12688/f1000research.17118.1	6381801	Duplicate
**450**	10.1177/1559827617695219	6378503	Duplicate
**451**	10.7861/clinmedicine.13-2-136	4952627	Duplicate
**452**	10.7861/clinmedicine.13-1-63	5873712	Duplicate
**453**	10.7861/clinmedicine.1-6-505	4953880	Duplicate
**454**		6306027	Duplicate
**455**		6306026	Duplicate
**456**	10.3390/ijms19123942	6320923	Duplicate

**457**	10.1038/aps.2017.77	5758669	Duplicate
**458**	10.1111/bjh.15011	5847561	Duplicate
**459**	10.5534/wjmh.180038	6305867	Duplicate
**460**	10.5534/wjmh.180027	6305869	Duplicate
**461**	10.2337/db16-1182	5697943	Duplicate
**462**	10.21037/jtd.2018.09.74	6236195	Duplicate
**463**	10.1097/MD.0000000000012559	6200524	Duplicate
**464**	10.4111/icu.2018.59.6.399	6215782	Duplicate
**465**	10.1111/bph.13743	5660004	Duplicate
**466**	10.1016/j.jbi.2017.08.009	5705492	Duplicate
**467**	10.1073/pnas.1809872115	6205494	Duplicate
**468**	10.2147/DMSO.S172057	6181110	Duplicate
**469**	10.1242/bio.036830	6176942	Duplicate
**470**	10.1002/ehf2.12306	6165933	Duplicate
**471**	10.1016/j.jsxm.2017.07.012	5624836	Duplicate
**472**	10.1016/j.visres.2017.03.002	5660664	Duplicate
**473**	10.1016/j.imr.2018.04.002	6160498	Duplicate
**474**	10.1371/journal.pone.0202725	6108484	Duplicate
**475**	10.5534/wjmh.180005	6119841	Duplicate
**476**	10.1186/s12933-018-0763-3	6117983	Duplicate
**477**	10.3390/nu10081126	6115838	Duplicate
**478**	10.1152/ajprenal.00489.2016	6109798	Duplicate
**479**	10.1002/jia2.25148	6062436	Duplicate
**480**	10.1002/rth2.12125	6032109	Duplicate
**481**	10.1007/s13300-018-0454-9	6028327	Duplicate
**482**	10.1371/journal.pone.0199299	6023114	Duplicate
**483**	10.1371/journal.pone.0199194	6014638	Duplicate
**484**	10.1186/s12958-018-0378-2	6015465	Duplicate
**485**	10.1111/apt.14674	6001629	Duplicate
**486**	10.1007/s13300-018-0415-3	5984918	Duplicate
**487**	10.1177/1557988315592026	5987948	Duplicate
**488**	10.1038/s41598-018-24147-y	5940896	Duplicate
**489**	10.1186/s12933-018-0704-1	5907287	Duplicate
**490**	10.1038/s41598-018-24347-6	5897450	Duplicate
**491**	10.1371/journal.pone.0194494	5882124	Duplicate
**492**	10.1155/2018/9389784	5831709	Duplicate
**493**	10.17925/EE.2015.11.02.81	5819072	Duplicate
**494**	10.1007/s11606-017-4028-8	5391321	Duplicate
**495**	10.1186/s12933-018-0688-x	5866526	Duplicate
**496**	10.1093/cvr/cvx008	5852638	Duplicate
**497**	10.1186/s12610-018-0068-0	5838858	Duplicate
**498**	10.1007/s13410-018-0604-7	5838201	Duplicate
**499**	10.1177/1557988316639050	5818109	Duplicate
**500**	10.1111/jvim.14858	5787188	Duplicate
**501**	10.3389/fcvm.2018.00001	5780411	Duplicate
**502**	10.1513/AnnalsATS.201608-605OC	5427733	Duplicate
**503**	10.1590/1414-431X20176601	5769756	Duplicate
**504**	10.4239/wjd.v9.i1.1	5763036	Duplicate
**505**	10.18632/oncotarget.22389	5739671	Duplicate
**506**	10.1177/2045893217743966	5731727	Duplicate
**507**	10.1177/2045893217743616	5731720	Duplicate
**508**	10.1136/bmjopen-2016-015599	5729999	Duplicate

**509**	10.21037/tau.2017.07.04	5715186	Duplicate
**510**	10.1021/acs.jmedchem.6b00669	5564430	Duplicate
**511**	10.1186/s10194-017-0817-z	5709272	Duplicate
**512**	10.3390/nu9111273	5707745	Duplicate
**513**	10.1259/bjr.20160366	5604913	Duplicate
**514**	10.1111/ijcp.12995	5698762	Duplicate
**515**	10.1016/j.esxm.2017.06.006	5693398	Duplicate
**516**	10.1155/2017/4375253	5646336	Duplicate
**517**	10.1681/ASN.2015050473	5084877	Duplicate
**518**	10.4103/jpp.JPP_42_17	5642133	Duplicate
**519**	10.7759/cureus.1598	5652893	Duplicate
**520**	10.1016/j.jcte.2016.11.003	5644434	Duplicate
**521**	10.1007/s13300-017-0313-0	5630567	Duplicate
**522**	10.1111/1440-1681.12796	5601287	Duplicate
**523**	10.1016/j.bbrep.2017.09.002	5613235	Duplicate
**524**	10.4070/kcj.2017.0009	5614939	Duplicate
**525**	10.1186/s12933-017-0595-6	5598064	Duplicate
**526**	10.21037/tau.2017.07.19	5583047	Duplicate
**527**	10.1155/2017/1253425	5574229	Duplicate
**528**	10.1016/j.atherosclerosis.2016.07.921	5035618	Duplicate
**529**	10.1186/s12933-017-0590-y	5577843	Duplicate
**530**	10.1016/j.jsxm.2016.06.004	5333763	Duplicate
**531**	10.1016/j.cgh.2015.11.021	4912904	Duplicate
**532**	10.1210/jc.2016-1294	4929841	Duplicate
**533**	10.3389/fphar.2017.00363	5468794	Duplicate
**534**	10.22038/IJBMS.2017.8690	5478785	Duplicate
**535**	10.1016/j.jcmgh.2017.04.001	5472192	Duplicate
**536**	10.1900/RDS.2015.12.63	5397984	Duplicate
**537**	10.1186/s12969-017-0141-9	5461530	Duplicate
**538**	10.1186/s12882-017-0553-2	5455080	Duplicate
**539**	10.1016/j.jsxm.2016.04.064	5317031	Duplicate
**540**	10.1007/s00125-017-4245-z	5423985	Duplicate
**541**	10.1111/cts.12047	5350776	Duplicate
**542**	10.3904/kjim.2016.208	5432803	Duplicate
**543**	10.1016/j.jsxm.2016.02.168	4886867	Duplicate
**544**	10.1186/s12890-017-0407-5	5405506	Duplicate
**545**	10.3390/healthcare5010015	5371921	Duplicate
**546**	10.7717/peerj.3020	5346286	Duplicate
**547**	10.1371/journal.pone.0172751	5330475	Duplicate
**548**	10.7860/JCDR/2016/19971.8996	5296448	Duplicate
**549**	10.3109/01658107.2013.817593	5291059	Duplicate
**550**	10.1038/nrdp.2016.3	5027992	Duplicate
**551**	10.1503/cmaj.151208	4674398	Duplicate
**552**	10.1093/eurheartj/suv054	4700909	Duplicate
**553**	10.1210/jc.2015-3415	4667163	Duplicate
**554**	10.6515/ACS20160611A	5126440	Duplicate
**555**	10.1016/j.aju.2016.07.002	5122751	Duplicate
**556**	10.1371/journal.pone.0165982	5089726	Duplicate
**557**	10.1136/heartjnl-2015-309223	5099221	Duplicate
**558**	10.1371/journal.pone.0157915	5051725	Duplicate
**559**	10.1093/eurheartj/ehv720	5074060	Duplicate
**560**	10.1177/1535370214547155	4935182	Duplicate

**561**	10.1186/s40635-016-0099-9	5042923	Duplicate
**562**		5010262	Duplicate
**563**	10.1152/japplphysiol.00316.2015	4687862	Duplicate
**564**	10.1016/j.esxm.2016.03.027	5005302	Duplicate
**565**	10.1155/2016/9364861	4993942	Duplicate
**566**	10.3390/ijms17081273	5000671	Duplicate
**567**	10.5534/wjmh.2016.34.2.89	4999494	Duplicate
**568**	10.1136/bcr-2014-205278	4154006	Duplicate
**569**	10.1186/s13098-016-0159-z	4964290	Duplicate
**570**	10.1007/s40256-016-0165-4	4947116	Duplicate
**571**	10.1111/j.1743-6109.2008.01209.x	4951185	Duplicate
**572**	10.1007/s10753-016-0359-6	4883282	Duplicate
**573**	10.1186/s13020-016-0096-7	4864906	Duplicate
**574**	10.7603/s40602-016-0003-6	4833805	Duplicate
**575**	10.1177/1479164115621667	4834510	Duplicate
**576**	10.1002/psp4.12049	4809625	Duplicate
**577**	10.1161/CIRCULATIONAHA.114.013215	4390480	Duplicate
**578**	10.1007/s11606-015-3271-0	4405523	Duplicate
**579**	10.1111/jsm.12848	4390459	Duplicate
**580**	10.1177/1756287215617648	4772354	Duplicate
**581**	10.3389/fphar.2016.00050	4782109	Duplicate
**582**	10.1177/2054270415622602	4776250	Duplicate
**583**		4768436	Duplicate
**584**		4755073	Duplicate
**585**	10.1016/j.pharmthera.2014.10.003	4494657	Duplicate
**586**	10.1002/14651858.CD006127.pub2	4439213	Duplicate
**587**	10.2174/1573403X09666131117174414	4347210	Duplicate
**588**	10.3978/j.issn.2223-4683.2012.07.03	4708248	Duplicate
**589**	10.1002/sm2.91	4721034	Duplicate
**590**	10.5114/aoms.2015.56342	4697050	Duplicate
**591**	10.1111/odi.12275	4275405	Duplicate
**592**	10.1177/2050640615601623	4669991	Duplicate
**593**	10.3109/13685538.2015.1004049	4648196	Duplicate
**594**	10.4103/1008-682X.140966	4650459	Duplicate
**595**	10.1186/s40200-015-0217-3	4665823	Duplicate
**596**		4634342	Duplicate
**597**	10.1155/2015/918069	4609427	Duplicate
**598**	10.1093/eurheartj/ehu204	4204003	Duplicate
**599**	10.1007/s00439-015-1572-3	4607040	Duplicate
**600**	10.1210/me.2014-1120	4179632	Duplicate
**601**	10.14814/phy2.12508	4562591	Duplicate
**602**	10.5152/tud.2013.97752	4548387	Duplicate
**603**		4525223	Duplicate
**604**	10.1186/s13098-015-0060-1	4546105	Duplicate
**605**	10.2147/DDDT.S85676	4535549	Duplicate
**606**	10.17795/ijpbs911	4525453	Duplicate
**607**	10.4137/CMED.S27700	4509465	Duplicate
**608**	10.1016/j.ophtha.2014.03.005	4122609	Duplicate
**609**	10.1371/journal.pone.0133121	4505907	Duplicate
**610**	10.3109/14767058.2012.717126	4511475	Duplicate
**611**	10.1177/2051415813491862	4467226	Duplicate
**612**	10.1111/j.1743-6109.2009.01458.x	4461030	Duplicate

**613**	10.1007/s40620-014-0140-6	4439441	Duplicate
**614**		4430881	Duplicate
**615**	10.1161/CIRCULATIONAHA.113.001805	4053195	Duplicate
**616**	10.1086/679705	4405710	Duplicate
**617**	10.1007/s11606-014-2834-9	4429500	Duplicate
**618**	10.1155/2015/548951	4415735	Duplicate
**619**	10.1038/ijir.2014.14	4216643	Duplicate
**620**	10.1186/s13063-015-0631-3	4411711	Duplicate
**621**	10.1111/j.1582-4934.2008.00639.x	3822508	Duplicate
**622**		4395914	Duplicate
**623**	10.4111/kju.2015.56.4.310	4392031	Duplicate
**624**	10.1155/2015/259592	4385644	Duplicate
**625**	10.1007/s40268-015-0085-9	4359185	Duplicate
**626**		4350889	Duplicate
**627**	10.5489/cuaj.2731	4336025	Duplicate
**628**	10.5489/cuaj.2699	4336024	Duplicate
**629**	10.2147/DMSO.S71376	4334308	Duplicate
**630**	10.1136/bcr-2012-007819	3604290	Duplicate
**631**	10.1001/jamaophthalmol.2013.6326	4050640	Duplicate
**632**	10.4239/wjd.v5.i6.905	4265879	Duplicate
**633**	10.1136/bmjdrc-2013-000004	4212558	Duplicate
**634**	10.1093/ehjci/jeu142	4240406	Duplicate
**635**	10.1371/journal.pone.0112394	4234367	Duplicate
**636**	10.1177/2050640614548980	4212306	Duplicate
**637**	10.1093/ajh/hpt098	3773573	Duplicate
**638**	10.2337/dc13-0315	3781524	Duplicate
**639**	10.2337/dc13-0294	3781490	Duplicate
**640**	10.2147/TCRM.S57610	4155803	Duplicate
**641**	10.1111/bjh.12245	4129543	Duplicate
**642**	10.1177/2042098611428486	4110829	Duplicate
**643**	10.1177/2050640613502899	4070608	Duplicate
**644**	10.1177/2050640613502900	4070603	Duplicate
**645**	10.1093/eurheartj/eht497	3992428	Duplicate
**646**	10.5489/cuaj.1962	4039601	Duplicate
**647**	10.3892/etm.2014.1582	3991500	Duplicate
**648**	10.4103/2230-8210.123552	4046605	Duplicate
**649**		4021780	Duplicate
**650**	10.1155/2014/653587	4009334	Duplicate
**651**	10.1155/2014/143763	4000629	Duplicate
**652**	10.1016/j.pharmthera.2008.05.005	4007052	Duplicate
**653**	10.1111/bph.12143	3651669	Duplicate
**654**	10.1155/2014/878670	3976909	Duplicate
**655**	10.3390/cells2020224	3972685	Duplicate
**656**	10.5489/cuaj.1608	3956835	Duplicate
**657**	10.2147/DMSO.S36455	3949699	Duplicate
**658**	10.2147/PPA.S48357	3920925	Duplicate
**659**	10.1097/MBP.0b013e328344c713	3915515	Duplicate
**660**	10.1016/j.jash.2011.02.007	3915530	Duplicate
**661**	10.4103/0975-7406.124317	3895294	Duplicate
**662**	10.1155/2013/504915	3884863	Duplicate
**663**	10.3205/cto000094	3884537	Duplicate
**664**	10.1371/journal.pone.0083951	3877124	Duplicate

**665**	10.1371/journal.pone.0085071	3873469	Duplicate
**666**	10.4111/kju.2013.54.12.858	3866290	Duplicate
**667**	10.1089/wound.2012.0422	3817001	Duplicate
**668**	10.4239/wjd.v4.i5.177	3797883	Duplicate
**669**		3776489	Duplicate
**670**	10.5534/wjmh.2013.31.2.83	3770856	Duplicate
**671**	10.5534/wjmh.2013.31.2.103	3770846	Duplicate
**672**	10.1038/aja.2010.123	3739608	Duplicate
**673**	10.1186/1471-244X-13-214	3766216	Duplicate
**674**	10.1016/j.juro.2012.04.001	3764461	Duplicate
**675**	10.1155/2013/323574	3728554	Duplicate
**676**	10.1007/s11606-011-1966-4	3378740	Duplicate
**677**	10.1097/HJH.0b013e32834000a7	3682653	Duplicate
**678**	10.1155/2013/763125	3665238	Duplicate
**679**	10.1164/rccm.201111-2082CI	3373067	Duplicate
**680**		3641729	Duplicate
**681**	10.5534/wjmh.2012.30.2.114	3623519	Duplicate
**682**	10.1007/s00125-011-2402-3	3329963	Duplicate
**683**	10.1186/1758-5996-4-43	3568051	Duplicate
**684**	10.1089/scd.2011.0303	3272247	Duplicate
**685**		3565859	Duplicate
**686**	10.1016/j.mayocp.2012.06.015	3498391	Duplicate
**687**	10.1155/2012/836893	3521631	Duplicate
**688**	10.1186/1471-2377-12-108	3517486	Duplicate
**689**		3410114	Duplicate
**690**	10.2147/VHRM.S26712	3433322	Duplicate
**691**	10.1210/jc.2010-2724	3167667	Duplicate
**692**		3413650	Duplicate
**693**	10.2337/dc10-2339	3120209	Duplicate
**694**		3351876	Duplicate
**695**	10.1136/amiajnl-2011-000113	3078666	Duplicate
**696**	10.1155/2012/569654	3303762	Duplicate
**697**	10.1038/nutd.2011.6	3302138	Duplicate
**698**	10.1186/1472-6904-12-5	3296596	Duplicate
**699**	10.4111/kju.2011.52.11.725	3242984	Duplicate
**700**	10.1016/j.juro.2010.12.098	3220602	Duplicate
**701**		3206546	Duplicate
**702**	10.1530/EJE-11-0221	3188848	Duplicate
**703**	10.2119/molmed.2011.00100	3188861	Duplicate
**704**	10.1186/1471-2261-11-36	3157429	Duplicate
**705**	10.2147/JPR.S21751	3141833	Duplicate
**706**		3048017	Duplicate
**707**	10.1124/pr.109.002014	2835398	Duplicate
**708**	10.2174/1874192401004010240	3026340	Duplicate
**709**		3020279	Duplicate
**710**	10.4065/mcp.2010.0164	2894722	Duplicate
**711**		2094729	Duplicate
**712**	10.1186/1475-2840-9-55	2954908	Duplicate
**713**	10.1152/ajpendo.90996.2008	2763792	Duplicate
**714**		2941787	Duplicate
**715**	10.1007/s00125-010-1819-4	2931646	Duplicate
**716**		2854169	Duplicate

**717**		2911822	Duplicate
**718**	10.1186/1472-6963-10-128	2893175	Duplicate
**719**	10.1186/1477-7827-8-50	2887879	Duplicate
**720**		1913720	Duplicate
**721**		2849981	Duplicate
**722**	10.2337/dc07-2375	2660483	Duplicate
**723**	10.1007/s11606-009-0963-3	2670986	Duplicate
**724**		2809989	Duplicate
**725**		2801586	Duplicate
**726**	10.1007/s00431-006-0349-z	2799065	Duplicate
**727**	10.2174/157340308785160589	2780822	Duplicate
**728**		2730071	Duplicate
**729**	10.4103/0970-1591.52907	2710058	Duplicate
**730**		2664585	Duplicate
**731**		2699638	Duplicate
**732**	10.3748/wjg.14.1564	2693753	Duplicate
**733**		2686330	Duplicate
**734**	10.4103/0970-1591.42612	2684375	Duplicate
**735**		2544367	Duplicate
**736**		1860735	Duplicate
**737**	10.1186/1475-2840-8-19	2667490	Duplicate
**738**	10.1186/1476-4598-7-82	2615789	Duplicate
**739**		1853342	Duplicate
**740**	10.1007/s00592-008-0030-2	2335289	Duplicate
**741**		1994005	Duplicate
**742**	10.1016/j.blre.2006.07.001	2048670	Duplicate
**743**	10.1111/j.1525-1497.2006.00469.x	1484878	Duplicate
**744**		1716227	Duplicate
**745**		1502384	Duplicate
**746**	10.1111/j.1525-1497.2005.020S1.x	1490295	Duplicate
**747**		1495721	Duplicate
**748**	10.1172/JCI27758	1359065	Duplicate
**749**		1120492	Duplicate
**750**		1117946	Duplicate
**751**	10.1186/1475-2840-2-8	194431	Duplicate
**752**	10.1177/00185787221115368	9445541	Duplicate

e)

**#**	**DOI**	**EMBASE PUI**	**STATUS**
**1**	10.1186/s13098-024-01363-3	L2030334235	INCLUDED IN TRIAL
**2**	10.1093/jsxmed/qdae064	L2033712496	PDE5 inhibitor and baseline HbA1c measured but endpoint HbA1c not measured
**3**	10.1056/NEJMcps2311302	L2033144729	Not a clinical trial
**4**	10.1093/jsxmed/qdae015	L2032111244	Not a clinical trial
**5**		L2028928778	Not a clinical trial
**6**	10.1016/j.fhj.2024.100097	L2033327698	Not a clinical trial
**7**	10.1016/j.jdiacomp.2023.108669	L2029777737	Not a clinical trial
**8**	10.1093/jsxmed/qdae001.148	L643787257	Not a clinical trial

**9**	10.1111/dme.15217	L2025449169	Does not solely vary PDE5 inhibitor treatment
**10**	10.1038/s41443-024-00895-6	L2029890876	Not a clinical trial
**11**	10.7196/SAMJ.2024.v114i4.1670	L2032392563	Not a clinical trial
**12**	10.21037/tau-23-71	L2029658898	Duplicate
**13**	10.1002/14651858.CD013166.pub2	L642306915	Not a clinical trial
**14**	10.1038/s41443-022-00601-4	L2018385817	Not a clinical trial
**15**	10.1111/andr.13372	L2021036706	Not a clinical trial
**16**	10.1016/j.ebiom.2023.104674	L2025446764	Duplicate
**17**	10.4111/icu.20230013	L2024656801	Not a clinical trial
**18**	10.23736/S2724-6507.21.03698-8	L2025149872	Not a clinical trial
**19**	10.23736/S2724-5683.22.06039-2	L2026095446	Not a clinical trial
**20**	10.1016/j.eclinm.2023.101985	L2024595623	Duplicate
**21**	10.1007/s12072-021-10264-w	L2014241830	data PDE5 inhibitor and baseline HbA1c measured but endpoint HbA1c not measured
**22**	10.1002/hsr2.1167	L2022841060	Duplicate
**23**	10.2337/dc23-S012	L2018812246	Not a clinical trial
**24**	10.1080/13685538.2023.2176484	L2021596294	Not a clinical trial
**25**	10.1620/tjem.2023.J020	L2023967606	Not a clinical trial
**26**	10.3389/fcvm.2023.1220000	L2024011396	Duplicate
**27**	10.2174/0113816128270340231121043038	L2027175135	Not a clinical trial
**28**		L2029041240	Not a clinical trial
**29**		L640366091	Not a clinical trial
**30**	10.1007/978-3-031-19443-6_24	L641032551	Not a clinical trial
**31**	10.1186/s13098-022-00825-w	L2015842954	Duplicate
**32**	10.1111/and.14629	L2020010202	Not a clinical trial
**33**	10.3390/jcm11237214	L2020508983	Duplicate
**34**	10.1016/S2666-1683(22)02069-9	L2020633648	Not a clinical trial
**35**	10.4103/aja2021105	L638986432	Duplicate
**36**	10.3389/fcvm.2022.985020	L2018863633	Not a clinical trial
**37**	10.1139/cjpp-2022-0132	L2018035538	Not a clinical trial
**38**	10.1016/j.niox.2022.05.005	L2018678744	Not a clinical trial
**39**	10.22038/IJBMS.2022.63378.13985	L2020333658	Duplicate
**40**	10.1111/and.14421	L2015354154	Duplicate
**41**	10.1126/scitranslmed.abl8503	L2019775456	Duplicate
**42**	10.3389/fendo.2022.847240	L2015670844	Duplicate
**43**	10.1016/j.jsxm.2022.01.213	L2017655104	Not a clinical trial
**44**	10.1016/j.jsxm.2022.01.213	L640967523	Not a clinical trial
**45**	10.3390/jpm12030454	L2016103060	Not a clinical trial
**46**	10.1016/j.metabol.2021.155005	L2017086854	Not a clinical trial
**47**	10.1111/aos.14853	L2010856874	Not a clinical trial
**48**	10.1002/dmrr.3494	L2013747123	Duplicate
**49**	10.1016/j.esxm.2021.100477	L2016372443	Duplicate
**50**	10.5114/pq.2022.121151	L2022753603	Clinical trial but no PDE5 inhibitor
**51**	10.1016/j.eprac.2021.07.004	L2015266949	Not a clinical trial
**52**	10.1007/s10389-020-01224-z	L2004227611	Not a clinical trial
**53**	10.1007/s10741-020-09955-7	L2005019727	Not a clinical trial
**54**	10.1016/j.esxm.2021.100400	L2013585478	Not a clinical trial
**55**	10.1024/0301-1526/a000944	L634291511	Not a clinical trial
**56**	10.3390/ijms22115973	L2007385183	Duplicate
**57**	10.1152/ajpcell.00595.2020	L2013002466	Duplicate

**58**	10.1111/bcp.14632	L2007395987	PDE5 inhibitor and baseline HbA1c measured but endpoint HbA1c not measured
**59**	10.1111/cen.14412	L2010710179	Not a clinical trial
**60**	10.1002/ccr3.3946	L2010525408	Duplicate
**61**	10.1016/j.metabol.2020.154469	L2010826713	Not a clinical trial
**62**	10.1016/j.jsxm.2021.01.122	L2011395670	Not a clinical trial
**63**	10.1007/s11695-020-04852-2	L2005597049	Not a clinical trial
**64**	10.1007/s11695-020-04855-z	L2005632980	Not a clinical trial
**65**	10.1111/andr.12919	L2007081987	Duplicate
**66**	10.1080/17512433.2021.1917381	L2011424187	Not a clinical trial
**67**	10.1097/MJT.0000000000001303	L2018427498	Duplicate
**68**	10.20452/pamw.15532	L2008457771	Not a clinical trial
**69**	10.3390/ijms21155338	L2004800914	Duplicate
**70**	10.1038/s41443-019-0149-4	L627556832	Not a clinical trial
**71**	10.4103/aja.aja_121_19	L632195743	Duplicate
**72**	10.1007/s13679-020-00378-x	L2004886209	Not a clinical trial
**73**		L633719033	Not a clinical trial
**74**	10.4103/ijnpnd.ijnpnd_83_19	L631671785	Not a clinical trial
**75**		L632060891	Not a clinical trial
**76**	10.1016/j.jsxm.2019.11.057	L2004351943	Not a clinical trial
**77**	10.1016/j.jsxm.2019.11.056	L2004351956	Not a clinical trial
**78**	10.1016/j.jsxm.2019.11.057	L640578771	Not a clinical trial
**79**	10.1161/ATVBAHA.119.313267	L630975212	Not a clinical trial
**80**	10.1016/j.esxm.2019.04.001	L2002092073	Duplicate
**81**	10.1016/j.jsxm.2019.01.202	L2001749233	Not a clinical trial
**82**	10.1016/j.jsxm.2019.01.003	L2001573372	Duplicate
**83**	10.2174/1389450118666170315110902	L2001599839	Not a clinical trial
**84**	10.1590/1806-9282.65.9.1133	L2003907399	Not a clinical trial
**85**	10.1038/s41443-018-0060-4	L623699542	Not a clinical trial
**86**	10.1002/oby.22346	L625593658	Does not solely vary PDE5 inhibitor treatment
**87**	10.1111/andr.12541	L624285952	Not a clinical trial
**88**		L624641450	Not a clinical trial
**89**		L624207682	Not a clinical trial
**90**	10.1016/j.pcad.2018.07.002	L2000943085	Not a clinical trial
**91**	10.2337/dc17-2510	L624998334	Duplicate
**92**	10.2337/ds16-0075	L620836068	Not a clinical trial
**93**		L622964657	Not a clinical trial
**94**	10.1007/s41782-017-0020-y	L625818755	Not a clinical trial
**95**	10.1111/ijcp.13027	L619243798	Not a clinical trial
**96**	10.1177/1479164117714397	L618419550	Not a clinical trial
**97**		L619899069	Not a clinical trial
**98**		L619900102	Not a clinical trial
**99**	10.1016/j.jsxm.2017.03.154	L640792358	Not a clinical trial
**100**	10.1371/journal.pone.0172751	L614604381	Duplicate
**101**	10.1080/13685538.2016.1230601	L612477467	Not a clinical trial
**102**	10.1080/13685538.2016.1260107	L614212785	Duplicate
**103**	10.1002/ejhf.662	L613729415	Duplicate
**104**	10.7717/peerj.3020	L614768856	Duplicate
**105**	10.1136/ejhpharm-2016-001008	L615121913	Duplicate
**106**		L619587426	Not a clinical trial

**107**	10.2174/1574886311666160426141851	L619707566	Not a clinical trial
**108**	10.1016/j.jcte.2016.11.003	L613510825	Duplicate
**109**		L613300918	Not a clinical trial
**110**		L619219560	Not a clinical trial
**111**	10.1093/eurheartj/ehw433	L612283448	Not a clinical trial
**112**	10.1186/s13098-016-0159-z	L611379959	Duplicate
**113**	10.1016/j.jsxm.2016.04.075	L610813372	Duplicate
**114**	10.1038/nrdp.2016.31	L610266688	Not a clinical trial
**115**	10.1016/j.jsxm.2016.03.171	L72325477	Not a clinical trial
**116**	10.1016/j.jsxm.2016.03.223	L72325540	Not a clinical trial
**117**	10.1016/j.jsxm.2016.03.258	L72325575	Not a clinical trial
**118**	10.1016/j.jsxm.2016.03.261	L72325578	Not a clinical trial
**119**	10.1002/psb.1450	L610447291	Not a clinical trial
**120**	10.1007/978-3-319-31587-4_5	L627404817	Not a clinical trial
**121**	10.1007/978-3-319-31587-4_9	L627405025	Not a clinical trial
**122**	10.1503/cmaj.150033	L607225058	Not a clinical trial
**123**	10.1517/14728222.2015.1066337	L607416084	Duplicate
**124**	10.3109/13685538.2015.1072154	L605633108	Duplicate
**125**		L619608786	Not a clinical trial
**126**	10.1111/jsm.12752	L602690965	Not a clinical trial
**127**	10.1371/journal.pone.0118134	L602337895	Not a clinical trial
**128**	10.4103/1008-682X.143250	L601132044	Not a clinical trial
**129**	10.1179/1607845414Y.0000000164	L601828650	Not a clinical trial
**130**	10.1016/j.pharmthera.2014.10.003	L602169070	Duplicate
**131**	10.1152/ajpregu.00420.2014	L602686580	Not a clinical trial
**132**		L604591822	Not a clinical trial
**133**	10.7417/T.2015.1885	L612738994	Not a clinical trial
**134**		L613817899	Not a clinical trial
**135**	10.1111/jsm.12826/abstract	L71788838	Not a clinical trial
**136**		L72038155	Not a clinical trial
**137**	10.1007/s00125-014-3355-0	L71594766	PDE5 inhibitor and baseline HbA1c measured but endpoint HbA1c not measured
**138**	10.1136/bcr-2014-205278	L373801852	Duplicate
**139**		L72336951	Not a clinical trial
**140**	10.2147/DMSO.S36455	L372594502	Duplicate
**141**	10.1111/dme.12378_2	L71554598	Not a clinical trial
**142**	10.1517/14656566.2014.934809	L373745358	Not a clinical trial
**143**	10.1111/jsm.12404	L52909579	Clinical trial but no PDE5 inhibitor
**144**	10.1111/jsm.12483	L53018851	Not a clinical trial
**145**	10.1373/clinchem.2013.214676	L604235027	Not a clinical trial
**146**	10.1186/1478-7547-11-31	L602650303	Not a clinical trial
**147**	10.4111/kju.2013.54.12.858	L370492298	Duplicate
**148**	10.1016/j.ecl.2013.07.003	L52793222	Not a clinical trial
**149**	10.1007/s00125-013-3012-z	L71439724	PDE5 inhibitor and baseline and endpoint HbA1c measured but too short
**150**	10.3109/10715762.2013.821701	L369512671	Not a clinical trial
**151**	10.1016/j.ejim.2013.01.001	L52411456	Not a clinical trial
**152**	10.1111/bjh.12245	L52448284	Duplicate
**153**		L71785319	Not a clinical trial
**154**	10.1111/j.1743-6109.2012.02783.x	L52209907	Not a clinical trial
**155**	10.2146/ajhp110221	L364346508	Not a clinical trial

**156**	10.1136/postgradmedj-2011-130069	L51835528	Not a clinical trial
**157**	10.1089/scd.2011.0303	L364212416	Duplicate
**158**		L364166557	Not a clinical trial
**159**	10.1016/j.mayocp.2012.06.015	L365658899	Duplicate
**160**	10.1016/j.diabet.2011.09.003	L51699200	Not a clinical trial
**161**	10.1111/j.1743-6109.2010.02546_3.x	L70612341	Not a clinical trial
**162**	10.1111/j.1365-2605.2011.01196.x	L51546014	Not a clinical trial
**163**	10.1159/000334326	L70570540	Not a clinical trial
**164**	10.1001/jama.2011.1203	L362549033	Not a clinical trial
**165**	10.1016/j.amjmed.2010.12.027	L361978639	Not a clinical trial
**166**	10.1038/ijir.2010.21	L51055257	Duplicate
**167**		L70334400	Not a clinical trial
**168**	10.1007/s10557-010-6254-8	L51006902	Not a clinical trial
**169**		L359095529	Not a clinical trial
**170**		L70167503	Duplicate
**171**	10.1111/j.1742-1241.2010.02392.x	L358606631	Not a clinical trial
**172**		L359794109	Not a clinical trial
**173**	10.1093/jat/34.4.169	L361616506	Not a clinical trial
**174**		L71008010	Not a clinical trial
**175**		L70149116	Not a clinical trial
**176**	10.1111/j.1743-6109.2009.01608-3.x	L70208029	Not a clinical trial
**177**	10.1016/S1557-0843(09)80023-7	L354529401	Not a clinical trial
**178**	10.2164/jandrol.108.005751	L354045496	Not a clinical trial
**179**	10.1111/j.1743-6109.2008.01209.x	L354559442	Duplicate
**180**	10.1111/j.1743-6109.2008.01125-3.x	L70207730	Not a clinical trial
**181**	10.2174/156652408786733658	L354400565	Not a clinical trial
**182**	10.1016/j.jomh.2008.03.014	L50222442	Not a clinical trial
**183**	10.1038/ijir.2008.36	L50224647	Clinical trial but no PDE5 inhibitor
**184**		L351955259	Not a clinical trial
**185**		L352579538	Not a clinical trial
**186**	10.1185/030079908X02498440	L354797258	Not a clinical trial
**187**	10.1016/j.diabres.2007.02.006	L47351245	Duplicate
**188**	10.2298/VSP0706399P	L47406342	Duplicate
**189**	10.2217/1745509X.2.6.1025	L46017491	Not a clinical trial
**190**	10.1080/01658100600981063	L44674048	Not a clinical trial
**191**	10.1111/j.1442-2042.2006.01480.x	L44125678	Duplicate
**192**	10.1016/j.fertnstert.2005.10.043	L43627672	Duplicate
**193**	10.1038/ncpuro0406	L43239265	Not a clinical trial
**194**	10.4088/jcp.v67n0617	L44049099	Not a clinical trial
**195**	10.1111/j.1743-6109.2006.00295.x	L44269544	Duplicate
**196**		L41583746	Not a clinical trial
**197**	10.1371/journal.pmed.0020040	L40418899	Not a clinical trial
**198**	10.1007/s00125-004-1549-6	L40110198	Duplicate
**199**		L39720668	Not a clinical trial
**200**	10.1002/pdi.658	L39144932	Not a clinical trial
**201**		L39095151	Not a clinical trial
**202**		L38195854	Not a clinical trial
**203**		L137582412	Not a clinical trial
**204**	10.1007/s00120-003-0420-6	L37383838	Not a clinical trial
**205**		L36859549	Not a clinical trial
**206**		L36998671	Not a clinical trial
**207**	10.2337/diacare.26.3.777	L36929343	Duplicate

**208**	10.1002/j.1939-4640.2003.tb02748.x	L37370529	Not a clinical trial
**209**	10.1016/s0212-6567(02)79030-6	L35264930	Duplicate
**210**	10.1007/s001250100656	L33010506	Duplicate
**211**		L32591590	Not a clinical trial
**212**		L32066366	Not a clinical trial
**213**	10.1016/S0140-6736(05)72164-4	L30107642	Not a clinical trial
**214**	10.1038/eye.2000.205	L30838331	Not a clinical trial
**215**		L29381637	Not a clinical trial
**216**	10.2337/diacare.22.6.989	L29241056	Not a clinical trial
**217**	10.2337/diacare.21.1.183	L28030406	Not a clinical trial

f)

#	DOI	CENTRAL ID	STATUS
**1**	10.1007/s001250100656	CN-00375093	Duplicate
**2**	10.2337/diacare.25.12.2159	CN-00434667	Duplicate
**3**	10.1007/s00125-004-1549-6	CN-00511440	Duplicate
**4**	10.1111/j.1442-2042.2006.01480.x	CN-00571622	Duplicate
**5**	10.1016/j.diabres.2007.02.006	CN-00609382	Duplicate
**6**	10.1016/j.eururo.2007.04.042	CN-00617985	Duplicate
**7**	10.1007/s00125-014-3355-0	CN-01009248	Duplicate
**8**		CN-01011295	drug but no a1c
**9**	10.1002/ejhf.662	CN-01332777	Duplicate
**10**		CN-01439200	Clinical trial but no PDE5 inhibitor
**11**		CN-01429761	Not a clinical trial
**12**		CN-01439629	Duplicate
**13**		CN-01548114	data PDE5 inhibitor and baseline HbA1c measured but endpoint HbA1c not measured
**14**		CN-01566434	Does not solely vary PDE5 inhibitor treatment
**15**		CN-01492149	Does not solely vary PDE5 inhibitor treatment
**16**		CN-01850624	Clinical trial but no PDE5 inhibitor
**17**		CN-01866343	drug but no a1c
**18**		CN-01808084	Does not solely vary PDE5 inhibitor treatment

**19**		CN-01856982	Clinical trial but no PDE5 inhibitor
**20**		CN-01895853	Study in progress or withdrawn
**21**	10.1093/eurheartj/ehw433	CN-01732650	Duplicate
**22**	10.1016/j.jsxm.2019.01.003	CN-01915911	Duplicate
**23**	10.4103/aja.aja_121_19	CN-02183638	Duplicate
**24**		CN-02213341	Clinical trial but no PDE5 inhibitor
**25**	10.1097/MJT.0000000000001303	CN-02232352	Duplicate
**26**		CN-02238054	Duplicate
**27**		CN-02296901	Does not solely vary PDE5 inhibitor treatment
**28**	10.1186/s13098-022-00825-w	CN-02394276	Duplicate
**29**	10.1126/scitranslmed.abl8503	CN-02413977	Duplicate
**30**		CN-02433656	Does not solely vary PDE5 inhibitor treatment
**31**		CN-02431595	Duplicate
**32**	10.1016/j.eclinm.2023.101985	CN-02567183	Duplicate
**33**	10.1186/s13098-022-00825-w	CN-02639324	Duplicate
**34**		CN-02704861	Does not solely vary PDE5 inhibitor treatment
**35**		CN-02722639	Does not solely vary PDE5 inhibitor treatment
**36**	10.1186/s13098-024-01363-3	CN-02721013	Duplicate
**37**	DOI	CN-02725687	Duplicate

g)

#	URL	CTGOV ID	STATUS
**1**	https://clinicaltrials.gov/study/NCT00199563	NCT00199563	data PDE5 inhibitor and baseline HbA1c measured but endpoint HbA1c not measured
**2**	https://clinicaltrials.gov/study/NCT00056433	NCT00056433	Does not solely vary PDE5 inhibitor treatment
**3**	https://clinicaltrials.gov/study/NCT05051436	NCT05051436	Study in progress or withdrawn
**4**	https://clinicaltrials.gov/study/NCT03364335	NCT03364335	Duplicate
**5**	https://clinicaltrials.gov/study/NCT05487755	NCT05487755	Duplicate

**6**	https://clinicaltrials.gov/study/NCT02252367	NCT02252367	data PDE5 inhibitor and baseline HbA1c measured but endpoint HbA1c not measured
**7**	https://clinicaltrials.gov/study/NCT01326117	NCT01326117	Study in progress or withdrawn
**8**	https://clinicaltrials.gov/study/NCT02546609	NCT02546609	Duplicate
**9**	https://clinicaltrials.gov/study/NCT02601989	NCT02601989	Duplicate
**10**	https://clinicaltrials.gov/study/NCT03834610	NCT03834610	data PDE5 inhibitor and baseline HbA1c measured but endpoint HbA1c not measured
**11**	https://clinicaltrials.gov/study/NCT01200394	NCT01200394	Duplicate
**12**	https://clinicaltrials.gov/study/NCT01084369	NCT01084369	Study in progress or withdrawn

h)

#	DOI	EMBASE PUI	STATUS
**1**	10.1111/joim.20012	L2031403463	Not a clinical trial
**2**	10.1093/jsxmed/qdae076	L2034464022	Not a clinical trial
**3**	10.1371/journal.pone.0304485	L2034401116	Not a clinical trial
**4**	10.1080/03007995.2024.2386047	L2030829373	Not a clinical trial
**5**	10.1093/jsxmed/qdae064	L2033712496	Duplicate
**6**	10.1016/j.fhj.2024.100097	L2033327698	Duplicate
**7**	10.1056/NEJMcps2311302	L2033144729	Duplicate
**8**	10.1186/s13098-024-01363-3	L2030334235	Duplicate
**9**	10.7196/SAMJ.2024.v114i4.1670	L2032392563	Duplicate
**10**	10.1038/s41443-024-00895-6	L2029890876	Duplicate
**11**		L2029041240	Duplicate
**12**	10.1093/jsxmed/qdae015	L2032111244	Duplicate
**13**	10.1093/jsxmed/qdae001.148	L643787257	Duplicate
**14**		L2028928778	Duplicate
**15**	10.1016/j.jdiacomp.2023.108669	L2029777737	Duplicate
**16**	10.21037/tau-23-71	L2029658898	Duplicate
**17**	10.2174/0113816128270340231121043038	L2027175135	Duplicate
**18**	10.1111/dme.15217	L2025449169	Duplicate
**19**	10.1002/14651858.CD013166.pub2	L642306915	Duplicate
**20**	10.1038/s41443-022-00601-4	L2018385817	Duplicate
**21**	10.1111/andr.13372	L2021036706	Duplicate
**22**	10.23736/S2724-5683.22.06039-2	L2026095446	Duplicate
**23**	10.4111/icu.20230013	L2024656801	Duplicate
**24**	10.1007/s12072-021-10264-w	L2014241830	Duplicate
**25**	10.1016/j.ebiom.2023.104674	L2025446764	Duplicate
**26**	10.3389/fcvm.2023.1220000	L2024011396	Duplicate
**27**	10.23736/S2724-6507.21.03698-8	L2025149872	Duplicate
**28**	10.1620/tjem.2023.J020	L2023967606	Duplicate
**29**	10.1016/j.eclinm.2023.101985	L2024595623	Duplicate
**30**	10.1007/978-3-031-19443-6_24	L641032551	Duplicate
**31**	10.1002/hsr2.1167	L2022841060	Duplicate
**32**	10.1016/S2666-1683(22)02069-9	L2020633648	Duplicate
**33**	10.1016/j.jsxm.2022.01.213	L640967523	Duplicate
**34**	10.1016/j.jsxm.2017.03.154	L640792358	Duplicate
**35**	10.1016/j.jsxm.2019.11.057	L640578771	Duplicate
**36**	10.1080/13685538.2023.2176484	L2021596294	Duplicate

**37**	10.5114/pq.2022.121151	L2022753603	Duplicate
**38**		L640366091	Duplicate
**39**	10.3390/jcm11237214	L2020508983	Duplicate
**40**	10.2337/dc23-S012	L2018812246	Duplicate
**41**	10.1111/and.14629	L2020010202	Duplicate
**42**	10.1097/MJT.0000000000001303	L2018427498	Duplicate
**43**	10.1139/cjpp-2022-0132	L2018035538	Duplicate
**44**	10.1016/j.niox.2022.05.005	L2018678744	Duplicate
**45**	10.22038/IJBMS.2022.63378.13985	L2020333658	Duplicate
**46**	10.4103/aja2021105	L638986432	Duplicate
**47**	10.3389/fcvm.2022.985020	L2018863633	Duplicate
**48**	10.1126/scitranslmed.abl8503	L2019775456	Duplicate
**49**	10.1001/jama.2011.1203	L362549033	Duplicate
**50**	10.1111/and.14421	L2015354154	Duplicate
**51**	10.3389/fendo.2022.847240	L2015670844	Duplicate
**52**	10.1186/s13098-022-00825-w	L2015842954	Duplicate
**53**	10.3390/jpm12030454	L2016103060	Duplicate
**54**	10.1016/j.jsxm.2022.01.213	L2017655104	Duplicate
**55**	10.1016/j.esxm.2021.100477	L2016372443	Duplicate
**56**	10.1080/17512433.2021.1917381	L2011424187	Duplicate
**57**	10.1016/j.metabol.2021.155005	L2017086854	Duplicate
**58**	10.1152/ajpcell.00595.2020	L2013002466	Duplicate
**59**	10.1016/j.esxm.2021.100400	L2013585478	Duplicate
**60**	10.1007/s10389-020-01224-z	L2004227611	Duplicate
**61**	10.1016/j.eprac.2021.07.004	L2015266949	Duplicate
**62**	10.1002/dmrr.3494	L2013747123	Duplicate
**63**	10.3390/ijms22115973	L2007385183	Duplicate
**64**	10.1024/0301-1526/a000944	L634291511	Duplicate
**65**	10.1007/s11695-020-04852-2	L2005597049	Duplicate
**66**	10.1002/ccr3.3946	L2010525408	Duplicate
**67**	10.1111/cen.14412	L2010710179	Duplicate
**68**	10.1111/aos.14853	L2010856874	Duplicate
**69**	10.1016/j.jsxm.2021.01.122	L2011395670	Duplicate
**70**	10.20452/pamw.15532	L2008457771	Duplicate
**71**	10.1111/andr.12919	L2007081987	Duplicate
**72**	10.1016/j.metabol.2020.154469	L2010826713	Duplicate
**73**		L633719033	Duplicate
**74**	10.1111/bcp.14632	L2007395987	Duplicate
**75**	10.1111/j.1743-6109.2012.02783.x	L52209907	Duplicate
**76**	10.1111/jsm.12404	L52909579	Duplicate
**77**	10.1111/jsm.12483	L53018851	Duplicate
**78**	10.1016/j.jsxm.2019.01.003	L2001573372	Duplicate
**79**	10.1111/jsm.12752	L602690965	Duplicate
**80**	10.1016/j.jsxm.2016.04.075	L610813372	Duplicate
**81**	10.4103/aja.aja_121_19	L632195743	Duplicate
**82**	10.3390/ijms21155338	L2004800914	Duplicate
**83**	10.1038/s41443-019-0149-4	L627556832	Duplicate
**84**	10.1007/s11695-020-04855-z	L2005632980	Duplicate
**85**		L632060891	Duplicate
**86**	10.4103/ijnpnd.ijnpnd_83_19	L631671785	Duplicate
**87**	10.1007/s13679-020-00378-x	L2004886209	Duplicate
**88**	10.1007/s10741-020-09955-7	L2005019727	Duplicate

**89**	10.1161/ATVBAHA.119.313267	L630975212	Duplicate
**90**	10.2146/ajhp110221	L364346508	Duplicate
**91**	10.1016/j.jsxm.2019.11.057	L2004351943	Duplicate
**92**	10.1016/j.jsxm.2019.11.056	L2004351956	Duplicate
**93**	10.1590/1806-9282.65.9.1133	L2003907399	Duplicate
**94**	10.1016/j.esxm.2019.04.001	L2002092073	Duplicate
**95**	10.1111/bjh.12245	L52448284	Duplicate
**96**	10.1038/s41443-018-0060-4	L623699542	Duplicate
**97**	10.1007/978-3-319-31587-4_5	L627404817	Duplicate
**98**	10.1007/978-3-319-31587-4_9	L627405025	Duplicate
**99**	10.1016/j.jsxm.2019.01.202	L2001749233	Duplicate
**100**	10.2174/1389450118666170315110902	L2001599839	Duplicate
**101**	10.1007/s41782-017-0020-y	L625818755	Duplicate
**102**	10.1002/oby.22346	L625593658	Duplicate
**103**	10.2337/dc17-2510	L624998334	Duplicate
**104**		L624641450	Duplicate
**105**		L624207682	Duplicate
**106**	10.1111/andr.12541	L624285952	Duplicate
**107**	10.1016/j.pcad.2018.07.002	L2000943085	Duplicate
**108**		L622964657	Duplicate
**109**	10.2174/1574886311666160426141851	L619707566	Duplicate
**110**	10.2337/ds16-0075	L620836068	Duplicate
**111**		L619899069	Duplicate
**112**		L619900102	Duplicate
**113**		L619608786	Duplicate
**114**		L619587426	Duplicate
**115**	10.1111/ijcp.13027	L619243798	Duplicate
**116**		L619219560	Duplicate
**117**	10.1177/1479164117714397	L618419550	Duplicate
**118**	10.1080/13685538.2016.1230601	L612477467	Duplicate
**119**	10.1080/13685538.2016.1260107	L614212785	Duplicate
**120**	10.1136/ejhpharm-2016-001008	L615121913	Duplicate
**121**	10.1371/journal.pone.0172751	L614604381	Duplicate
**122**	10.7717/peerj.3020	L614768856	Duplicate
**123**	10.1002/ejhf.662	L613729415	Duplicate
**124**		L613817899	Duplicate
**125**	10.1016/j.jcte.2016.11.003	L613510825	Duplicate
**126**		L613300918	Duplicate
**127**	10.7417/T.2015.1885	L612738994	Duplicate
**128**	10.1093/eurheartj/ehw433	L612283448	Duplicate
**129**	10.1186/s13098-016-0159-z	L611379959	Duplicate
**130**	10.1016/j.jsxm.2016.03.171	L72325477	Duplicate
**131**	10.1016/j.jsxm.2016.03.223	L72325540	Duplicate
**132**	10.1016/j.jsxm.2016.03.258	L72325575	Duplicate
**133**	10.1016/j.jsxm.2016.03.261	L72325578	Duplicate
**134**		L72336951	Duplicate
**135**	10.1038/nrdp.2016.31	L610266688	Duplicate
**136**	10.1002/psb.1450	L610447291	Duplicate
**137**	10.1517/14728222.2015.1066337	L607416084	Duplicate
**138**	10.1503/cmaj.150033	L607225058	Duplicate
**139**	10.3109/13685538.2015.1072154	L605633108	Duplicate
**140**		L72038155	Duplicate

**141**		L604591822	Duplicate
**142**	10.1373/clinchem.2013.214676	L604235027	Duplicate
**143**	10.1186/1478-7547-11-31	L602650303	Duplicate
**144**	10.1152/ajpregu.00420.2014	L602686580	Duplicate
**145**	10.1371/journal.pone.0118134	L602337895	Duplicate
**146**	10.1016/j.pharmthera.2014.10.003	L602169070	Duplicate
**147**	10.1111/jsm.12826/abstract	L71788838	Duplicate
**148**		L71785319	Duplicate
**149**	10.1179/1607845414Y.0000000164	L601828650	Duplicate
**150**	10.4103/1008-682X.143250	L601132044	Duplicate
**151**	10.1136/bcr-2014-205278	L373801852	Duplicate
**152**	10.1517/14656566.2014.934809	L373745358	Duplicate
**153**	10.1007/s00125-014-3355-0	L71594766	Duplicate
**154**	10.1111/dme.12378_2	L71554598	Duplicate
**155**	10.1007/s00125-013-3012-z	L71439724	Duplicate
**156**	10.2147/DMSO.S36455	L372594502	Duplicate
**157**	10.4111/kju.2013.54.12.858	L370492298	Duplicate
**158**	10.1016/j.ecl.2013.07.003	L52793222	Duplicate
**159**	10.3109/10715762.2013.821701	L369512671	Duplicate
**160**	10.1016/j.ejim.2013.01.001	L52411456	Duplicate
**161**		L71008010	Duplicate
**162**	10.1016/j.mayocp.2012.06.015	L365658899	Duplicate
**163**	10.1136/postgradmedj-2011-130069	L51835528	Duplicate
**164**	10.1016/j.diabet.2011.09.003	L51699200	Duplicate
**165**	10.1089/scd.2011.0303	L364212416	Duplicate
**166**		L364166557	Duplicate
**167**	10.1111/j.1743-6109.2010.02546_3.x	L70612341	Duplicate
**168**	10.1159/000334326	L70570540	Duplicate
**169**	10.1111/j.1365-2605.2011.01196.x	L51546014	Duplicate
**170**	10.1016/j.amjmed.2010.12.027	L361978639	Duplicate
**171**	10.1093/jat/34.4.169	L361616506	Duplicate
**172**		L70334400	Duplicate
**173**	10.1007/s10557-010-6254-8	L51006902	Duplicate
**174**	10.1038/ijir.2010.21	L51055257	Duplicate
**175**		L359794109	Duplicate
**176**		L359095529	Duplicate
**177**	10.1111/j.1743-6109.2008.01125-3.x	L70207730	Duplicate
**178**	10.1111/j.1743-6109.2009.01608-3.x	L70208029	Duplicate
**179**		L70167503	Duplicate
**180**		L70149116	Duplicate
**181**	10.1111/j.1742-1241.2010.02392.x	L358606631	Duplicate
**182**	10.1185/03007990802498440	L354797258	Duplicate
**183**	10.1111/j.1743-6109.2008.01209.x	L354559442	Duplicate
**184**	10.1016/S1557-0843(09)80023-7	L354529401	Duplicate
**185**	10.2174/156652408786733658	L354400565	Duplicate
**186**	10.2164/jandrol.108.005751	L354045496	Duplicate
**187**		L352579538	Duplicate
**188**	10.1038/ijir.2008.36	L50224647	Duplicate
**189**	10.1016/j.jomh.2008.03.014	L50222442	Duplicate
**190**		L351955259	Duplicate
**191**	10.1016/j.diabres.2007.02.006	L47351245	Duplicate
**192**	10.2298/VSP0706399P	L47406342	Duplicate

**193**	10.2217/1745509X.2.6.1025	L46017491	Duplicate
**194**	10.1080/01658100600981063	L44674048	Duplicate
**195**	10.1111/j.1743-6109.2006.00295.x	L44269544	Duplicate
**196**	10.4088/jcp.v67n0617	L44049099	Duplicate
**197**	10.1111/j.1442-2042.2006.01480.x	L44125678	Duplicate
**198**	10.1016/j.fertnstert.2005.10.043	L43627672	Duplicate
**199**	10.1038/ncpuro0406	L43239265	Duplicate
**200**		L41583746	Duplicate
**201**	10.1371/journal.pmed.0020040	L40418899	Duplicate
**202**	10.1007/s00125-004-1549-6	L40110198	Duplicate
**203**		L39720668	Duplicate
**204**	10.1002/pdi.658	L39144932	Duplicate
**205**		L39095151	Duplicate
**206**		L137582412	Duplicate
**207**		L38195854	Duplicate
**208**	10.1007/s00120-003-0420-6	L37383838	Duplicate
**209**	10.1002/j.1939-4640.2003.tb02748.x	L37370529	Duplicate
**210**		L36998671	Duplicate
**211**	10.2337/diacare.26.3.777	L36929343	Duplicate
**212**		L36859549	Duplicate
**213**	10.1016/s0212-6567(02)79030-6	L35264930	Duplicate
**214**	10.1007/s001250100656	L33010506	Duplicate
**215**		L32591590	Duplicate
**216**		L32066366	Duplicate
**217**	10.1038/eye.2000.205	L30838331	Duplicate
**218**	10.1016/S0140-6736(05)72164-4	L30107642	Duplicate
**219**		L29381637	Duplicate
**220**	10.2337/diacare.22.6.989	L29241056	Duplicate
**221**	10.2337/diacare.21.1.183	L28030406	Duplicate

i)

#	TITLE	ICTRP ID	STATUS
**1**	Daily Oral L-arginine Plus Tadalafil in Diabetic Patients With Erectile Dysfunction: A Double-blinded Randomized Controlled Clinical Trial	NCT03834610	Duplicate

j)

#	DOI	PMC ID	STATUS
**1**	10.1503/cmaj.240399	11426347	Not a clinical trial
**2**	10.6026/973206300200705	11414330	Not a clinical trial
**3**	10.3390/ijms25179260	11395528	Not a clinical trial
**4**	10.1177/23969873231174267	11391896	drug but no a1c
**5**	10.1177/23969873231169660	11391898	Not a clinical trial
**6**	10.1186/s12969-024-01005-y	11384717	Not a clinical trial
**7**	10.3233/JPD-240149	11380238	Not a clinical trial
**8**	10.1371/journal.pone.0304485	11371211	Duplicate
**9**	10.1002/ccr3.9408	11358210	Not a clinical trial
**10**	10.1177/11786329241266648	11359442	Not a clinical trial
**11**	10.1007/s40261-024-01379-7	11338981	Not a clinical trial
**12**	10.2174/1570159X22666240212141602	11337684	Not a clinical trial

**13**	10.1007/s00395-024-01045-1	11319409	Not a clinical trial
**14**	10.3389/fendo.2024.1451100	11319149	Not a clinical trial
**15**	10.3390/ijms25158202	11311839	Not a clinical trial
**16**	10.4103/indianjpsychiatry.indianjpsychiatry_784_23	11293283	Not a clinical trial
**17**	10.1161/JAHA.124.035264	11292752	Not a clinical trial
**18**	10.1038/s41598-024-68208-x	11291728	Not a clinical trial
**19**	10.31083/j.rcm2304115	11273979	Not a clinical trial
**20**	10.1111/ene.16339	11235923	Not a clinical trial
**21**	10.1007/s11255-024-04003-x	11266281	PDE5 inhibitor and baseline HbA1c measured but endpoint HbA1c not measured
**22**	10.1016/j.heliyon.2024.e32524	467047	Not a clinical trial
**23**	10.7759/cureus.62537	11254138	Not a clinical trial
**24**	10.3390/ijms25137295	11242737	Not a clinical trial
**25**	10.1186/s12610-024-00229-y	11225209	Clinical trial but no PDE5 inhibitor
**26**	10.1111/jdi.14192	11215682	Not a clinical trial
**27**	10.1002/ajh.26923	10272107	Not a clinical trial
**28**	10.1007/s11606-023-08226-z	10323075	Not a clinical trial
**29**	10.1097/JU.0000000000003481	10330773	Clinical trial but no PDE5 inhibitor
**30**	10.5493/wjem.v14.i2.93689	11212738	Not a clinical trial
**31**	10.3389/fmolb.2024.1390814	11200040	Not a clinical trial
**32**	10.1007/s40618-023-02285-z	11196288	Not a clinical trial
**33**	10.1186/s13098-024-01363-3	11194930	Duplicate
**34**	10.1152/ajpendo.00267.2023	11193530	Clinical trial but no PDE5 inhibitor
**35**		11192546	Clinical trial but no PDE5 inhibitor
**36**	10.3390/diagnostics14111125	11172383	Not a clinical trial
**37**	10.1177/0271678X231176482	10265373	Not a clinical trial
**38**	10.1007/s00125-024-06146-z	11153285	Not a clinical trial
**39**	10.1007/s40618-023-02237-7	11142995	Duplicate
**40**	10.1038/s41598-024-61287-w	11087471	Duplicate
**41**	10.2147/JMDH.S455089	11075683	Duplicate
**42**	10.5114/pm.2024.136327	11056728	Duplicate
**43**	10.1007/s40259-024-00650-9	11055746	Duplicate
**44**	10.3390/antiox13040455	11047699	Duplicate
**45**	10.1016/S2213-8587(19)30405-X	11044807	Duplicate
**46**	10.1080/14712598.2023.2203811	10330142	Duplicate
**47**	10.1007/s10456-023-09903-7	11021332	Duplicate
**48**	10.1002/mco2.516	11014467	Duplicate
**49**	10.1093/europace/euae043	11000153	Duplicate
**50**	10.3748/wjg.v30.i9.1073	10989500	Duplicate
**51**	10.1159/000537829	10987180	Duplicate
**52**		10775241	Duplicate
**53**	10.1002/14651858.CD006127.pub3	10049880	Duplicate
**54**	10.1016/j.heliyon.2024.e27206	10955197	Duplicate
**55**	10.11817/j.issn.1672-7347.2023.220267	10930255	Duplicate
**56**	10.4103/jhrs.JHRS_ISAR_IFS	10942128	Duplicate
**57**	10.5935/1518-0557.20230071	10936909	Duplicate
**58**	10.5213/inj.2346250.125	10932574	Duplicate
**59**	10.1177/20420188241229540	10929063	Duplicate
**60**	10.1148/radiol.220743	9968769	Duplicate
**61**	10.22038/IJBMS.2023.73410.15950	10897555	Duplicate
**62**	10.25122/jml-2023-0290	10893579	Duplicate

**63**	10.1210/clinem/dgad100	10438886	Duplicate
**64**	10.1210/clinem/dgad572	10876414	Duplicate
**65**	10.1093/sexmed/qfae001	10849181	Duplicate
**66**	10.4102/safp.v66i1.5822	10839213	Duplicate
**67**	10.3390/ijms25020821	10815857	Duplicate
**68**	10.14744/hf.2022.2022.0052	10809338	Duplicate
**69**	10.1007/s11845-023-03412-8	10808673	Duplicate
**70**	10.1136/bmjopen-2023-078325	10806598	Duplicate
**71**	10.5114/pm.2023.133883	10793607	Duplicate
**72**	10.5114/pm.2023.133280	10793608	Duplicate
**73**	10.1016/j.cccb.2023.100199	10792690	Duplicate
**74**	10.7759/cureus.50515	10787576	Duplicate
**75**	10.1021/acsptsci.2c00082	9926524	Duplicate
**76**	10.1016/j.jdiacomp.2022.108288	10783766	Duplicate
**77**	10.21037/tau-23-71	10772644	Duplicate
**78**	10.1210/endrev/bnad026	10765166	Duplicate
**79**	10.3389/fendo.2023.1301093	10766371	Duplicate
**80**	10.3390/diagnostics13243650	10743125	Duplicate
**81**	10.3390/medicina59122190	10744870	Duplicate
**82**	10.1038/s42255-023-00931-7	10730394	Duplicate
**83**	10.1186/s12882-023-03427-4	10731818	Duplicate
**84**	10.1016/j.heliyon.2023.e22482	10700708	Duplicate
**85**	10.1152/japplphysiol.00478.2022	9762960	Duplicate
**86**	10.1097/MD.0000000000035939	10637505	Duplicate
**87**	10.1016/j.jceh.2022.03.012	9630008	Duplicate
**88**	10.1002/jmri.28133	9411265	Duplicate
**89**	10.2967/jnumed.122.264795	10152130	Duplicate
**90**	10.1016/j.heliyon.2023.e21844	10661066	Duplicate
**91**	10.3390/ijms242015078	10606418	Duplicate
**92**	10.3389/fendo.2023.1238090	10600375	Duplicate
**93**	10.1186/s12944-023-01950-9	10601238	Duplicate
**94**	10.1186/s12969-023-00879-8	10580657	Duplicate
**95**	10.1186/s40001-023-01413-y	10566198	Duplicate
**96**	10.1097/CLD.0000000000000066	10550044	Duplicate
**97**	10.7759/cureus.44576	10545003	Duplicate
**98**	10.1177/00185787221115368	9445541	Duplicate
**99**	10.5114/aoms/135634	10508044	Duplicate
**100**	10.1016/j.jacbts.2023.02.017	10504399	Duplicate
**101**	10.7573/dic.2023-5-4	10499368	Duplicate
**102**	10.2147/PPA.S412969	10493135	Duplicate
**103**	10.3390/ijms241713541	10488183	Duplicate
**104**	10.3390/ijms241713226	10488129	Duplicate
**105**	10.25122/jml-2023-0068	10478652	Duplicate
**106**	10.1016/j.jceh.2022.05.001	9499842	Duplicate
**107**	10.1186/s12933-023-01956-8	10436534	Duplicate
**108**	10.1210/clinem/dgad180	10438885	Duplicate
**109**	10.1042/CS20220795	10415166	Duplicate
**110**	10.1080/19585969.2022.2134739	10408697	Duplicate
**111**	10.36628/ijhf.2023.0016	10406556	Duplicate
**112**	10.4070/kcj.2023.0114	10406530	Duplicate
**113**	10.18553/jmcp.2016.22.5.449	10398103	Duplicate
**114**	10.1016/j.jsxm.2022.04.010	9329230	Duplicate

**115**	10.1055/s-0041-1740936	9948071	Duplicate
**116**	10.3389/fphar.2023.1184572	10367013	Duplicate
**117**	10.1136/bcr-2020-241439	8311326	Duplicate
**118**	10.1038/s41420-023-01553-4	10362058	Duplicate
**119**	10.1038/s41419-023-05935-5	10336063	Duplicate
**120**	10.2147/CIA.S405121	10337775	Duplicate
**121**	10.1016/j.mad.2023.111818	10330534	Duplicate
**122**	10.1016/j.ebiom.2023.104674	10328805	Duplicate
**123**	10.1177/14791641231183634	10328035	Duplicate
**124**	10.5534/wjmh.221027	10307648	Duplicate
**125**	10.5534/wjmh.220057	10307658	Duplicate
**126**	10.3390/life13061305	10301499	Duplicate
**127**	10.3390/medicina59061119	10304508	Duplicate
**128**	10.3390/biom13060930	10295993	Duplicate
**129**	10.1038/s41419-023-05904-y	10293205	Duplicate
**130**	10.3390/genes14061233	10297911	Duplicate
**131**	10.1007/s11255-023-03602-4	10293434	Duplicate
**132**	10.3389/fcvm.2023.1220000	10291612	Duplicate
**133**	10.1186/s13098-023-01117-7	10288670	Duplicate
**134**	10.1186/s12877-023-04100-z	10286414	Duplicate
**135**	10.1002/pul2.12249	10271598	Duplicate
**136**	10.1093/hropen/hoad023	10270320	Duplicate
**137**	10.1016/j.metop.2023.100247	10267599	Duplicate
**138**	10.1016/j.eclinm.2023.101985	10225663	Duplicate
**139**	10.1007/s11101-023-09869-w	10205037	Duplicate
**140**	10.3390/jox13020017	10204391	Duplicate
**141**	10.5005/jp-journals-10071-23712A	10202389	Not a clinical trial
**142**	10.1007/s10741-023-10321-6	10185959	Duplicate
**143**	10.1016/j.heliyon.2023.e15778	10176068	Duplicate
**144**	10.5688/ajpe9171	10159487	Duplicate
**145**	10.3389/fendo.2023.1135530	10151816	Duplicate
**146**	10.1038/s41433-022-02007-4	9046155	Duplicate
**147**	10.1161/CIRCRESAHA.122.319967	9060387	Duplicate
**148**	10.3390/biology12040558	10135985	Duplicate
**149**	10.3390/cancers15082316	10136828	Duplicate
**150**	10.1007/s00508-023-02167-7	10133034	Duplicate
**151**	10.1186/s12991-023-00447-0	10122283	Duplicate
**152**	10.4103/0970-1591.368611	10121074	Duplicate
**153**	10.1111/andr.13328	10107754	Duplicate
**154**	10.1002/hsr2.1167	10090802	Duplicate
**155**	10.5588/ijtld.22.0514	10094053	Duplicate
**156**	10.3389/fendo.2023.1149239	10086443	Duplicate
**157**	10.1111/andr.13192	10084359	Duplicate
**158**	10.2147/JPR.S397777	10069439	Duplicate
**159**	10.1093/pcmedi/pbad005	10068425	Duplicate
**160**	10.3390/ijms24065478	10058074	Duplicate
**161**	10.1016/j.ebiom.2023.104506	10043778	Duplicate
**162**	10.1016/j.jbc.2023.103059	10033317	Duplicate
**163**	10.3389/fendo.2023.1134325	10028207	Duplicate
**164**	10.5334/gh.1165	10000335	Duplicate
**165**		9997681	Duplicate
**166**	10.1001/jamacardio.2023.0065	9996460	Duplicate

**167**	10.3389/fmed.2023.1110532	9971232	Duplicate
**168**	10.3389/fonc.2023.1113462	9939513	Duplicate
**169**	10.3389/fphar.2023.1033492	9939646	Duplicate
**170**	10.21203/rs.3.rs-2475555/v1	9934781	Duplicate
**171**	10.3390/healthcare11030433	9914730	Duplicate
**172**	10.3390/jcm12030846	9917599	Duplicate
**173**	10.1016/j.jacbts.2022.05.010	9911324	Duplicate
**174**	10.21203/rs.3.rs-2515453/v1	9915771	Duplicate
**175**	10.3389/fendo.2022.1036243	9902700	Duplicate
**176**	10.1101/2023.01.25.525428	9900832	Duplicate
**177**	10.1186/s12894-023-01180-2	9901095	Duplicate
**178**	10.1002/pul2.12153	9748629	Duplicate
**179**	10.3389/fendo.2023.1017886	9889556	Duplicate
**180**	10.20517/jca.2022.42	9894375	Duplicate
**181**	10.1186/s12967-022-03800-1	9887734	Duplicate
**182**	10.34172/apb.2023.039	9871268	Duplicate
**183**	10.1007/s40618-023-02015-5	9876440	Duplicate
**184**	10.3390/jpm13010108	9862349	Duplicate
**185**	10.6515/ACS.202301_39(1).20221103A	9829849	Duplicate
**186**	10.1055/s-0041-1735209	9272316	Duplicate
**187**	10.3389/fnagi.2022.899389	9831269	Duplicate
**188**	10.14744/bej.2022.71676	9794510	Duplicate
**189**	10.1038/s41392-022-01257-8	9797940	Duplicate
**190**	10.1111/1753-0407.13334	9789395	Duplicate
**191**	10.3389/fendo.2022.1032268	9767955	Duplicate
**192**	10.1016/j.prnil.2022.06.004	9747574	Duplicate
**193**	10.1007/s12026-022-09352-2	9760530	Duplicate
**194**	10.1155/2022/5243594	9757934	Duplicate
**195**	10.3389/fneur.2022.988825	9756760	Duplicate
**196**	10.3390/jcm11237214	9740756	Duplicate
**197**	10.3390/jcm11236891	9737178	Duplicate
**198**	10.3389/fphar.2022.1057083	9731127	Duplicate
**199**	10.1111/jdi.13903	9720202	Duplicate
**200**	10.1111/jvim.16541	9708457	Duplicate
**201**	10.3389/fnut.2022.930272	9691656	Duplicate
**202**	10.1371/journal.pone.0276963	9668147	Duplicate
**203**	10.21037/atm-22-3678	9652507	Duplicate
**204**	10.4103/jfmpc.jfmpc_1130_21	9648286	Duplicate
**205**	10.1007/s11910-022-01240-4	9663281	Duplicate
**206**	10.7759/cureus.29699	9616173	Duplicate
**207**	10.3390/jpm12101738	9604792	Duplicate
**208**	10.1002/cpt.2627	9398938	Duplicate
**209**	10.4183/aeb.2022.174	9512378	Duplicate
**210**	10.1038/s41598-022-19364-5	9515177	Duplicate
**211**	10.3390/nu14183737	9504067	Duplicate
**212**	10.22038/IJBMS.2022.63378.13985	9464341	Duplicate
**213**	10.4103/aja2021105	9491027	Duplicate
**214**	10.1016/j.bjorl.2017.12.002	9449167	Duplicate
**215**	10.1038/s41401-020-00557-5	8379181	Duplicate
**216**	10.1111/1753-0407.13300	9426278	Duplicate
**217**	10.1007/s11010-022-04520-2	9421626	Duplicate
**218**	10.7759/cureus.27337	9414788	Duplicate

**219**	10.3390/biomedicines10081848	9405076	Duplicate
**220**	10.3390/life12081222	9410036	Duplicate
**221**	10.1161/CIRCULATIONAHA.121.053889	8384699	Duplicate
**222**	10.1186/s12967-022-03581-7	9389664	Duplicate
**223**	10.1007/s00059-022-05123-9	9355932	Duplicate
**224**	10.1111/andr.13169	9310719	Duplicate
**225**	10.1038/s42003-022-03716-y	9314386	Duplicate
**226**	10.3390/jcm11144027	9320223	Duplicate
**227**	10.3390/jpm12071139	9319005	Duplicate
**228**	10.1016/j.nerep.2022.100121	9314262	Duplicate
**229**	10.3389/fphys.2022.906272	9304560	Duplicate
**230**	10.1002/dmrr.3494	9286480	Duplicate
**231**	10.1016/j.amsu.2022.104137	9283796	Duplicate
**232**	10.1007/s00210-022-02249-9	9276575	Duplicate
**233**	10.1038/s41392-022-01073-0	9259665	Duplicate
**234**	10.1016/j.expneurol.2021.113694	8169562	Duplicate
**235**	10.1007/s12325-022-02168-4	9239965	Duplicate
**236**	10.1093/function/zqac029	9228651	Duplicate
**237**	10.3390/jcm11123382	9224931	Duplicate
**238**	10.1038/s41746-022-00617-6	9198008	Duplicate
**239**	10.3390/jcm11113118	9181610	Duplicate
**240**	10.1186/s13063-022-06296-8	9186476	Duplicate
**241**	10.6515/ACS.202205_38(3).20220321A	9121756	Duplicate
**242**	10.1016/j.jsxm.2021.03.077	8253516	Duplicate
**243**	10.3390/diagnostics12051249	9141739	Duplicate
**244**	10.4103/indianjpsychiatry.indianjpsychiatry_1014_21	9122155	Duplicate
**245**	10.1186/s13014-022-02060-z	9115982	Duplicate
**246**		9028527	Duplicate
**247**	10.1001/jama.2021.18463	8596197	Duplicate
**248**	10.3389/fimmu.2022.882032	9082262	Duplicate
**249**	10.3389/fendo.2022.821113	9065269	Duplicate
**250**	10.1111/dme.14276	9065795	Duplicate
**251**		9045589	Duplicate
**252**	10.18549/PharmPract.2022.1.2487	9014904	Duplicate
**253**	10.1155/2022/9404025	9038412	Duplicate
**254**	10.1371/journal.pone.0267047	9038205	Duplicate
**255**	10.1097/MJT.0000000000001303	9035320	Duplicate
**256**	10.1186/s13098-022-00825-w	9022238	Duplicate
**257**	10.3389/fendo.2022.847240	9022207	Duplicate
**258**	10.23750/abm.v93i1.12910	8972891	Duplicate
**259**	10.5534/wjmh.210021	8987149	Duplicate
**260**	10.5534/wjmh.200157	8987134	Duplicate
**261**	10.3390/ijms23073535	8998588	Duplicate
**262**	10.21037/tau-22-58	8984969	Duplicate
**263**	10.1177/2397198319898367	8922672	Duplicate
**264**	10.1007/s40200-021-00782-7	8212202	Duplicate
**265**	10.1016/j.jdiacomp.2020.107841	8007279	Duplicate
**266**	10.1249/MSS.0000000000002521	7969358	Duplicate
**267**	10.3390/biom12020278	8961612	Duplicate
**268**	10.3390/jcm11061632	8956033	Duplicate
**269**	10.3390/antiox11030580	8945168	Duplicate
**270**	10.1016/j.esxm.2021.100477	8847829	Duplicate

**271**	10.1155/2022/5583298	8791751	Duplicate
**272**	10.5534/wjmh.200184	8761237	Duplicate
**273**	10.5534/wjmh.200176	8761244	Duplicate
**274**	10.1016/j.esxm.2021.100438	8766268	Duplicate
**275**	10.1016/j.clnu.2021.11.030	8757535	Duplicate
**276**	10.2215/CJN.08410520	7792638	Duplicate
**277**	10.1002/ehf2.13590	8712918	Duplicate
**278**	10.7759/cureus.19408	8654114	Duplicate
**279**	10.1111/j.1751-7176.2010.00416.x	8108786	Duplicate
**280**	10.12669/pjms.37.7.4257	8613049	Duplicate
**281**	10.17245/jdapm.2021.21.6.479	8637910	Duplicate
**282**	10.1186/s40001-021-00618-3	8655085	Duplicate
**283**	10.1136/bcr-2019-232433	6904175	Duplicate
**284**	10.1093/eurheartj/ehab389	8599003	Duplicate
**285**	10.3389/fmed.2021.665023	8595206	Duplicate
**286**	10.1007/s40618-021-01598-1	8572206	Duplicate
**287**	10.4252/wjsc.v13.i10.1549	8567456	Duplicate
**288**	10.1177/23969873211026698	8564163	Duplicate
**289**	10.1186/s12913-021-07158-w	8540874	Duplicate
**290**	10.3390/nu13103529	8541559	Duplicate
**291**	10.3390/medicina57090868	8467670	Duplicate
**292**	10.1177/2050640619854671	8454868	Duplicate
**293**	10.21037/tau-21-441	8421832	Duplicate
**294**	10.3390/ijms22179296	8431217	Duplicate
**295**	10.1161/HYPERTENSIONAHA.120.14930	7429358	Duplicate
**296**	10.1016/j.amsu.2021.102748	8387920	Duplicate
**297**	10.1007/s42000-021-00295-1	8357658	Duplicate
**298**	10.3390/ijms22168880	8396298	Duplicate
**299**	10.3389/fphar.2021.518345	8381854	Duplicate
**300**	10.1177/20458940211037274	8381443	Duplicate
**301**	10.2147/JEP.S236743	8380049	Duplicate
**302**	10.1177/20420188211034297	8365016	Duplicate
**303**	10.3389/fcvm.2021.715400	8329089	Duplicate
**304**	10.4239/wjd.v12.i7.954	8311479	Duplicate
**305**	10.1186/s12931-021-01797-7	8314029	Duplicate
**306**	10.36660/abc.20210180	8294740	Duplicate
**307**	10.1007/s10067-020-05551-0	8289755	Duplicate
**308**	10.1007/s11255-021-02867-x	8280019	Duplicate
**309**	10.21037/atm-21-2479	8267313	Duplicate
**310**	10.1007/s10822-021-00409-2	8273033	Duplicate
**311**	10.1186/s12872-021-02146-8	8256614	Duplicate
**312**	10.1186/s12902-021-00807-5	8252293	Duplicate
**313**	10.1186/s12967-021-02935-x	8259336	Duplicate
**314**	10.5534/wjmh.200109	8255402	Duplicate
**315**	10.1111/dom.14322	8248154	Duplicate
**316**	10.2196/25409	8218212	Duplicate
**317**	10.1002/14651858.CD012787.pub2	8130994	Duplicate
**318**	10.1038/s41443-020-0242-8	7483362	Duplicate
**319**	10.1155/2021/6656406	8205584	Duplicate
**320**	10.3390/biology10060540	8235660	Duplicate
**321**	10.3390/nu13061784	8225153	Duplicate
**322**		8221238	Duplicate

**323**	10.3390/jcm10112501	8201035	Duplicate
**324**	10.3390/ijms22115973	8198766	Duplicate
**325**	10.7150/ijms.58147	8176183	Duplicate
**326**	10.4103/aja.aja_71_20	8152419	Duplicate
**327**	10.1152/ajpheart.00024.2020	7311696	Duplicate
**328**	10.1161/ATVBAHA.119.313883	7255946	Duplicate
**329**	10.3390/jcm10102221	8161068	Duplicate
**330**	10.1136/bcr-2018-228872	6536206	Duplicate
**331**	10.1007/s40618-020-01453-9	8124039	Duplicate
**332**	10.1002/ehf2.13327	8120363	Duplicate
**333**	10.3390/ijms22094666	8125634	Duplicate
**334**	10.1002/ccr3.3946	8077334	Duplicate
**335**	10.3389/fphar.2021.654489	8076853	Duplicate
**336**	10.3390/ph14040365	8071249	Duplicate
**337**	10.2337/db19-0432	7085247	Duplicate
**338**	10.1016/j.oret.2019.11.008	7150646	Duplicate
**339**	10.3390/healthcare9030257	8000828	Duplicate
**340**	10.1007/s40618-020-01381-8	7946690	Duplicate
**341**	10.1007/s12664-020-01133-9	7972945	Duplicate
**342**	10.1186/s12894-020-00730-2	7945372	Duplicate
**343**	10.1172/jci.insight.146175	7934844	Duplicate
**344**	10.1165/rcmb.2019-0226PS	6993553	Duplicate
**345**	10.1038/s41569-020-0339-2	7849055	Duplicate
**346**	10.1167/tvst.10.1.13	7804520	Duplicate
**347**	10.2147/CEOR.S285434	7814241	Duplicate
**348**	10.1038/s41467-020-20500-w	7801461	Duplicate
**349**	10.21037/tau-20-999	7807359	Duplicate
**350**	10.1007/s13679-020-00422-w	7787121	Duplicate
**351**	10.1007/s11845-020-02450-w	7788179	Duplicate
**352**	10.1080/13543784.2020.1705277	6990416	Duplicate
**353**	10.1111/bph.14920	7707100	Duplicate
**354**	10.1016/S0212-6567(02)79030-6	7684223	Duplicate
**355**	10.2478/jomb-2019-0043	7682853	Duplicate
**356**	10.3390/molecules25225481	7700551	Duplicate
**357**	10.3390/molecules25225318	7696151	Duplicate
**358**	10.1177/2050640617725676	7672678	Duplicate
**359**	10.3390/ijms21218244	7662747	Duplicate
**360**	10.3390/pharmaceutics12090882	7558015	Duplicate
**361**	10.1093/braincomms/fcaa020	7530832	Duplicate
**362**	10.1002/jia2.25616	7533869	Duplicate
**363**	10.1002/ehf2.12772	7524060	Duplicate
**364**	10.1007/s12028-019-00710-x	6759381	Duplicate
**365**	10.1016/j.neures.2018.10.005	6462258	Duplicate
**366**	10.1016/j.ijcha.2020.100632	7502339	Duplicate
**367**	10.1177/1179546820953415	7466888	Duplicate
**368**	10.1038/s41598-020-71946-3	7484782	Duplicate
**369**	10.3390/biom10081162	7464753	Duplicate
**370**	10.3390/jcm9082513	7463896	Duplicate
**371**	10.3390/ijms21155338	7432892	Duplicate
**372**	10.1007/s12551-020-00742-0	7429613	Duplicate
**373**	10.4103/aja.aja_121_19	7406094	Duplicate
**374**	10.4103/aja.aja_106_19	7406095	Duplicate

**375**	10.1155/2020/7078108	7407035	Duplicate
**376**	10.3892/etm.2020.8934	7401651	Duplicate
**377**	10.18632/aging.103571	7377835	Duplicate
**378**	10.1007/s00246-020-02408-w	7393337	Duplicate
**379**	10.1016/j.ijcha.2020.100584	7385446	Duplicate
**380**	10.1155/2020/9175676	7364200	Duplicate
**381**	10.1186/s12874-020-01078-9	7382082	Duplicate
**382**	10.3390/jcm9061995	7355625	Duplicate
**383**	10.4103/ijem.IJEM_225_20	7328526	Duplicate
**384**	10.1002/trc2.12050	7364858	Duplicate
**385**	10.1155/2020/3293065	7345966	Duplicate
**386**	10.5688/ajpe8220	7334344	Duplicate
**387**	10.1152/japplphysiol.00947.2018	6692740	Duplicate
**388**	10.3390/ijms21114020	7312670	Duplicate
**389**	10.4103/aja.aja_15_19	7275806	Duplicate
**390**	10.4103/ijmr.IJMR_502_20	7288773	Duplicate
**391**	10.3390/biom10050752	7277861	Duplicate
**392**	10.1038/s41366-019-0517-7	7260126	Duplicate
**393**	10.1016/B978-3-437-21203-1.10002-0	7271212	Duplicate
**394**	10.1016/B978-343721332-8.50004-1	7271219	Duplicate
**395**	10.1016/B978-3-437-23246-6.10004-3	7271201	Duplicate
**396**	10.1002/ehf2.12694	7261527	Duplicate
**397**	10.1016/j.esxm.2020.02.006	7261691	Duplicate
**398**	10.1016/j.esxm.2020.01.010	7261672	Duplicate
**399**	10.1016/j.esxm.2020.01.006	7261708	Duplicate
**400**	10.1002/14651858.CD012625.pub2	6535156	Duplicate
**401**	10.3390/ijms21082703	7216146	Duplicate
**402**	10.21037/tau.2020.02.07	7214985	Duplicate
**403**	10.1097/HJH.0000000000001988	7223638	Duplicate
**404**	10.15420/ecr.2019.14	7199190	Duplicate
**405**	10.1210/jc.2019-00683	6735730	Duplicate
**406**	10.1038/s41387-020-0116-7	7186220	Duplicate
**407**	10.1007/978-3-642-33108-4_1	7176304	Duplicate
**408**	10.1111/hiv.12513	7165664	Duplicate
**409**	10.1111/jns.12225	7166380	Duplicate
**410**	10.1002/ppul.23576	7159391	Duplicate
**411**	10.1016/B978-0-323-52993-8.00048-5	7161392	Duplicate
**412**	10.1016/B978-0-12-803206-0.00001-8	7149322	Duplicate
**413**	10.1007/978-1-4419-6505-9_2	7139436	Duplicate
**414**	10.1007/978-3-319-11821-5_2	7123375	Duplicate
**415**	10.1007/978-1-60761-685-6_5	7123449	Duplicate
**416**	10.1007/s12055-018-0649-8	7102261	Duplicate
**417**	10.1007/s11096-008-9226-3	7102062	Duplicate
**418**	10.1002/agm2.12097	7099759	Duplicate
**419**	10.1038/s41409-019-0559-4	7091813	Duplicate
**420**	10.1177/1753466620910092	7074506	Duplicate
**421**	10.1155/2020/5214751	7064866	Duplicate
**422**	10.7717/peerj.8653	7050549	Duplicate
**423**	10.3389/fnins.2020.00140	7046549	Duplicate
**424**	10.1177/0300060519859134	7045648	Duplicate
**425**	10.1016/j.esxm.2019.08.012	7042165	Duplicate
**426**	10.1186/s13613-020-0623-7	7013036	Duplicate

**427**	10.2169/internalmedicine.3372-19	7028403	Duplicate
**428**	10.1093/gerona/glz056	6909887	Duplicate
**429**	10.2169/internalmedicine.3625-19	7008044	Duplicate
**430**	10.3390/ijms21010116	6982327	Duplicate
**431**	10.1177/2042018819897527	6977225	Duplicate
**432**	10.4103/aja.aja_108_19	6958978	Duplicate
**433**	10.4103/aja.aja_92_19	6958986	Duplicate
**434**	10.1111/jdi.13089	6944840	Duplicate
**435**	10.1016/j.jacbts.2019.08.008	6939015	Duplicate
**436**	10.1038/s41572-019-0087-y	6944317	Duplicate
**437**	10.14744/nci.2018.23230	6936940	Duplicate
**438**	10.5534/wjmh.180052M	6920065	Duplicate
**439**	10.1016/j.heliyon.2019.e03035	6928307	Duplicate
**440**	10.3390/s19235311	6928990	Duplicate
**441**	10.1136/bmjopen-2019-029098	6887087	Duplicate
**442**	10.1002/14651858.CD010060.pub2	6517182	Duplicate
**443**	10.1186/s12882-019-1584-7	6820937	Duplicate
**444**	10.1007/s11892-017-0917-9	6826336	Duplicate
**445**	10.1177/2050640618792819	6796246	Duplicate
**446**	10.17179/excli2019-1447	6785772	Duplicate
**447**	10.5489/cuaj.6197	6752998	Duplicate
**448**	10.2337/dc18-0255	6150428	Duplicate
**449**	10.1152/physrev.00033.2017	6170978	Duplicate
**450**	10.1371/journal.pone.0221992	6738611	Duplicate
**451**	10.1002/14651858.CD002187.pub3	6718223	Duplicate
**452**	10.1016/j.esxm.2019.04.001	6728771	Duplicate
**453**	10.1186/s13104-019-4576-6	6720088	Duplicate
**454**	10.1177/2040622319868376	6709440	Duplicate
**455**	10.2337/dc17-2285	6105327	Duplicate
**456**	10.1002/clc.21993	6652630	Duplicate
**457**	10.1002/clc.4960271305	6654274	Duplicate
**458**	10.3390/jcm8071017	6678562	Duplicate
**459**	10.3390/ijms20133299	6651183	Duplicate
**460**	10.1177/1751143719835452	6661815	Duplicate
**461**	10.15420/ecr.2019.13.1	6659039	Duplicate
**462**	10.5114/ada.2019.83656	6640017	Duplicate
**463**		6614614	Duplicate
**464**	10.1002/edm2.64	6613223	Duplicate
**465**	10.4111/icu.2019.60.4.275	6607068	Duplicate
**466**	10.1186/s13063-019-3474-5	6588901	Duplicate
**467**	10.1007/s11302-018-9637-0	6339618	Duplicate
**468**	10.2337/dc17-2510	6014549	Duplicate
**469**	10.1016/j.ihj.2018.05.003	6097178	Duplicate
**470**	10.1016/j.ihj.2018.01.002	6097164	Duplicate
**471**	10.1002/cpt.979	6590078	Duplicate
**472**	10.1371/journal.pone.0217690	6563988	Duplicate
**473**	10.1002/14651858.CD009183.pub2	6540387	Duplicate
**474**	10.1007/s40618-018-0977-y	6531405	Duplicate
**475**	10.1136/ejhpharm-2016-001008	6451457	Duplicate
**476**	10.1172/jci.insight.123618	6538324	Duplicate
**477**	10.1016/j.esxm.2019.01.004	6522934	Duplicate
**478**	10.2196/12459	6483060	Duplicate

**479**	10.5534/wjmh.180057	6479089	Duplicate
**480**	10.1172/jci.insight.123611	6485674	Duplicate
**481**	10.4103/aam.aam_3_18	6330780	Duplicate
**482**	10.1002/14651858.CD008226.pub3	6464917	Duplicate
**483**	10.1161/CIRCRESAHA.118.311912	5901903	Duplicate
**484**	10.12688/f1000research.16561.1	6436191	Duplicate
**485**	10.1186/s12933-019-0847-8	6432760	Duplicate
**486**	10.1111/jvim.15372	6430903	Duplicate
**487**	10.7555/JBR.31.20160164	6352876	Duplicate
**488**	10.3399/bjgp18X695261	5819978	Duplicate
**489**	10.12688/f1000research.17118.1	6381801	Duplicate
**490**	10.1177/1559827617695219	6378503	Duplicate
**491**	10.7861/clinmedicine.13-2-136	4952627	Duplicate
**492**	10.7861/clinmedicine.13-1-63	5873712	Duplicate
**493**	10.7861/clinmedicine.1-6-505	4953880	Duplicate
**494**		6306027	Duplicate
**495**		6306026	Duplicate
**496**	10.3390/ijms19123942	6320923	Duplicate
**497**	10.1038/aps.2017.77	5758669	Duplicate
**498**	10.1111/bjh.15011	5847561	Duplicate
**499**	10.5534/wjmh.180027	6305869	Duplicate
**500**	10.2337/db16-1182	5697943	Duplicate
**501**	10.21037/jtd.2018.09.74	6236195	Duplicate
**502**	10.1097/MD.0000000000012559	6200524	Duplicate
**503**	10.4111/icu.2018.59.6.399	6215782	Duplicate
**504**	10.1111/bph.13743	5660004	Duplicate
**505**	10.1016/j.jbi.2017.08.009	5705492	Duplicate
**506**	10.1073/pnas.1809872115	6205494	Duplicate
**507**	10.2147/DMSO.S172057	6181110	Duplicate
**508**	10.1242/bio.036830	6176942	Duplicate
**509**	10.1002/ehf2.12306	6165933	Duplicate
**510**	10.1016/j.jsxm.2017.07.012	5624836	Duplicate
**511**	10.1016/j.visres.2017.03.002	5660664	Duplicate
**512**	10.1016/j.imr.2018.04.002	6160498	Duplicate
**513**	10.1371/journal.pone.0202725	6108484	Duplicate
**514**	10.5534/wjmh.180005	6119841	Duplicate
**515**	10.1186/s12933-018-0763-3	6117983	Duplicate
**516**	10.3390/nu10081126	6115838	Duplicate
**517**	10.1152/ajprenal.00489.2016	6109798	Duplicate
**518**	10.1002/jia2.25148	6062436	Duplicate
**519**	10.1002/rth2.12125	6032109	Duplicate
**520**	10.1007/s13300-018-0454-9	6028327	Duplicate
**521**	10.1371/journal.pone.0199299	6023114	Duplicate
**522**	10.1371/journal.pone.0199194	6014638	Duplicate
**523**	10.1186/s12958-018-0378-2	6015465	Duplicate
**524**	10.1111/apt.14674	6001629	Duplicate
**525**	10.1007/s13300-018-0415-3	5984918	Duplicate
**526**	10.1177/1557988315592026	5987948	Duplicate
**527**	10.1038/s41598-018-24147-y	5940896	Duplicate
**528**	10.1186/s12933-018-0704-1	5907287	Duplicate
**529**	10.1038/s41598-018-24347-6	5897450	Duplicate
**530**	10.1371/journal.pone.0194494	5882124	Duplicate

**531**	10.1155/2018/9389784	5831709	Duplicate
**532**	10.17925/EE.2015.11.02.81	5819072	Duplicate
**533**	10.1007/s11606-017-4028-8	5391321	Duplicate
**534**	10.1186/s12933-018-0688-x	5866526	Duplicate
**535**	10.1093/cvr/cvx008	5852638	Duplicate
**536**	10.1186/s12610-018-0068-0	5838858	Duplicate
**537**	10.1007/s13410-018-0604-7	5838201	Duplicate
**538**	10.1177/1557988316639050	5818109	Duplicate
**539**	10.1111/jvim.14858	5787188	Duplicate
**540**	10.3389/fcvm.2018.00001	5780411	Duplicate
**541**	10.1513/AnnalsATS.201608-605OC	5427733	Duplicate
**542**	10.1590/1414-431X20176601	5769756	Duplicate
**543**	10.4239/wjd.v9.i1.1	5763036	Duplicate
**544**	10.18632/oncotarget.22389	5739671	Duplicate
**545**	10.1177/2045893217743966	5731727	Duplicate
**546**	10.1177/2045893217743616	5731720	Duplicate
**547**	10.1136/bmjopen-2016-015599	5729999	Duplicate
**548**	10.21037/tau.2017.07.04	5715186	Duplicate
**549**	10.1021/acs.jmedchem.6b00669	5564430	Duplicate
**550**	10.1186/s10194-017-0817-z	5709272	Duplicate
**551**	10.3390/nu9111273	5707745	Duplicate
**552**	10.1259/bjr.20160366	5604913	Duplicate
**553**	10.1111/ijcp.12995	5698762	Duplicate
**554**	10.1016/j.esxm.2017.06.006	5693398	Duplicate
**555**	10.1155/2017/4375253	5646336	Duplicate
**556**	10.1681/ASN.2015050473	5084877	Duplicate
**557**	10.4103/jpp.JPP_42_17	5642133	Duplicate
**558**	10.7759/cureus.1598	5652893	Duplicate
**559**	10.1016/j.jcte.2016.11.003	5644434	Duplicate
**560**	10.1007/s13300-017-0313-0	5630567	Duplicate
**561**	10.1111/1440-1681.12796	5601287	Duplicate
**562**	10.1016/j.bbrep.2017.09.002	5613235	Duplicate
**563**	10.4070/kcj.2017.0009	5614939	Duplicate
**564**	10.1186/s12933-017-0595-6	5598064	Duplicate
**565**	10.21037/tau.2017.07.19	5583047	Duplicate
**566**	10.1155/2017/1253425	5574229	Duplicate
**567**	10.1016/j.atherosclerosis.2016.07.921	5035618	Duplicate
**568**	10.1186/s12933-017-0590-y	5577843	Duplicate
**569**	10.1016/j.jsxm.2016.06.004	5333763	Duplicate
**570**	10.1016/j.cgh.2015.11.021	4912904	Duplicate
**571**	10.1210/jc.2016-1294	4929841	Duplicate
**572**	10.3389/fphar.2017.00363	5468794	Duplicate
**573**	10.22038/IJBMS.2017.8690	5478785	Duplicate
**574**	10.1016/j.jcmgh.2017.04.001	5472192	Duplicate
**575**	10.1900/RDS.2015.12.63	5397984	Duplicate
**576**	10.1186/s12969-017-0141-9	5461530	Duplicate
**577**	10.1186/s12882-017-0553-2	5455080	Duplicate
**578**	10.1016/j.jsxm.2016.04.064	5317031	Duplicate
**579**	10.1007/s00125-017-4245-z	5423985	Duplicate
**580**	10.1111/cts.12047	5350776	Duplicate
**581**	10.3904/kjim.2016.208	5432803	Duplicate
**582**	10.1016/j.jsxm.2016.02.168	4886867	Duplicate

**583**	10.1186/s12890-017-0407-5	5405506	Duplicate
**584**	10.3390/healthcare5010015	5371921	Duplicate
**585**	10.7717/peerj.3020	5346286	Duplicate
**586**	10.1371/journal.pone.0172751	5330475	Duplicate
**587**	10.7860/JCDR/2016/19971.8996	5296448	Duplicate
**588**	10.3109/01658107.2013.817593	5291059	Duplicate
**589**	10.1038/nrdp.2016.3	5027992	Duplicate
**590**	10.1503/cmaj.151208	4674398	Duplicate
**591**	10.1093/eurheartj/suv054	4700909	Duplicate
**592**	10.1210/jc.2015-3415	4667163	Duplicate
**593**	10.6515/ACS20160611A	5126440	Duplicate
**594**	10.1016/j.aju.2016.07.002	5122751	Duplicate
**595**	10.1371/journal.pone.0165982	5089726	Duplicate
**596**	10.1136/heartjnl-2015-309223	5099221	Duplicate
**597**	10.1371/journal.pone.0157915	5051725	Duplicate
**598**	10.1093/eurheartj/ehv720	5074060	Duplicate
**599**	10.1177/1535370214547155	4935182	Duplicate
**600**	10.1186/s40635-016-0099-9	5042923	Duplicate
**601**		5010262	Duplicate
**602**	10.1152/japplphysiol.00316.2015	4687862	Duplicate
**603**	10.1016/j.esxm.2016.03.027	5005302	Duplicate
**604**	10.1155/2016/9364861	4993942	Duplicate
**605**	10.3390/ijms17081273	5000671	Duplicate
**606**	10.5534/wjmh.2016.34.2.89	4999494	Duplicate
**607**	10.1136/bcr-2014-205278	4154006	Duplicate
**608**	10.1186/s13098-016-0159-z	4964290	Duplicate
**609**	10.1007/s40256-016-0165-4	4947116	Duplicate
**610**	10.1111/j.1743-6109.2008.01209.x	4951185	Duplicate
**611**	10.1007/s10753-016-0359-6	4883282	Duplicate
**612**	10.1186/s13020-016-0096-7	4864906	Duplicate
**613**	10.7603/s40602-016-0003-6	4833805	Duplicate
**614**	10.1177/1479164115621667	4834510	Duplicate
**615**	10.1002/psp4.12049	4809625	Duplicate
**616**	10.1161/CIRCULATIONAHA.114.013215	4390480	Duplicate
**617**	10.1007/s11606-015-3271-0	4405523	Duplicate
**618**	10.1111/jsm.12848	4390459	Duplicate
**619**	10.1177/1756287215617648	4772354	Duplicate
**620**	10.3389/fphar.2016.00050	4782109	Duplicate
**621**	10.1177/2054270415622602	4776250	Duplicate
**622**		4768436	Duplicate
**623**		4755073	Duplicate
**624**	10.1016/j.pharmthera.2014.10.003	4494657	Duplicate
**625**	10.1002/14651858.CD006127.pub2	4439213	Duplicate
**626**	10.2174/1573403X09666131117174414	4347210	Duplicate
**627**	10.3978/j.issn.2223-4683.2012.07.03	4708248	Duplicate
**628**	10.1002/sm2.91	4721034	Duplicate
**629**	10.5114/aoms.2015.56342	4697050	Duplicate
**630**	10.1111/odi.12275	4275405	Duplicate
**631**	10.1177/2050640615601623	4669991	Duplicate
**632**	10.3109/13685538.2015.1004049	4648196	Duplicate
**633**	10.4103/1008-682X.140966	4650459	Duplicate
**634**	10.1186/s40200-015-0217-3	4665823	Duplicate

**635**		4634342	Duplicate
**636**	10.1155/2015/918069	4609427	Duplicate
**637**	10.1093/eurheartj/ehu204	4204003	Duplicate
**638**	10.1007/s00439-015-1572-3	4607040	Duplicate
**639**	10.1210/me.2014-1120	4179632	Duplicate
**640**	10.14814/phy2.12508	4562591	Duplicate
**641**	10.5152/tud.2013.97752	4548387	Duplicate
**642**		4525223	Duplicate
**643**	10.1186/s13098-015-0060-1	4546105	Duplicate
**644**	10.2147/DDDT.S85676	4535549	Duplicate
**645**	10.17795/ijpbs911	4525453	Duplicate
**646**	10.4137/CMED.S27700	4509465	Duplicate
**647**	10.1016/j.ophtha.2014.03.005	4122609	Duplicate
**648**	10.1371/journal.pone.0133121	4505907	Duplicate
**649**	10.3109/14767058.2012.717126	4511475	Duplicate
**650**	10.1177/2051415813491862	4467226	Duplicate
**651**	10.1111/j.1743-6109.2009.01458.x	4461030	Duplicate
**652**	10.1007/s40620-014-0140-6	4439441	Duplicate
**653**		4430881	Duplicate
**654**	10.1161/CIRCULATIONAHA.113.001805	4053195	Duplicate
**655**	10.1086/679705	4405710	Duplicate
**656**	10.1007/s11606-014-2834-9	4429500	Duplicate
**657**	10.1155/2015/548951	4415735	Duplicate
**658**	10.1038/ijir.2014.14	4216643	Duplicate
**659**	10.1186/s13063-015-0631-3	4411711	Duplicate
**660**	10.1111/j.1582-4934.2008.00639.x	3822508	Duplicate
**661**		4395914	Duplicate
**662**	10.4111/kju.2015.56.4.310	4392031	Duplicate
**663**	10.1155/2015/259592	4385644	Duplicate
**664**	10.1007/s40268-015-0085-9	4359185	Duplicate
**665**		4350889	Duplicate
**666**	10.5489/cuaj.2731	4336025	Duplicate
**667**	10.5489/cuaj.2699	4336024	Duplicate
**668**	10.2147/DMSO.S71376	4334308	Duplicate
**669**	10.1136/bcr-2012-007819	3604290	Duplicate
**670**	10.1001/jamaophthalmol.2013.6326	4050640	Duplicate
**671**	10.4239/wjd.v5.i6.905	4265879	Duplicate
**672**	10.1136/bmjdrc-2013-000004	4212558	Duplicate
**673**	10.1093/ehjci/jeu142	4240406	Duplicate
**674**	10.1371/journal.pone.0112394	4234367	Duplicate
**675**	10.1177/2050640614548980	4212306	Duplicate
**676**	10.1093/ajh/hpt098	3773573	Duplicate
**677**	10.2337/dc13-0315	3781524	Duplicate
**678**	10.2337/dc13-0294	3781490	Duplicate
**679**	10.2147/TCRM.S57610	4155803	Duplicate
**680**	10.1111/bjh.12245	4129543	Duplicate
**681**	10.1177/2042098611428486	4110829	Duplicate
**682**	10.1177/2050640613502899	4070608	Duplicate
**683**	10.1177/2050640613502900	4070603	Duplicate
**684**	10.1093/eurheartj/eht497	3992428	Duplicate
**685**	10.5489/cuaj.1962	4039601	Duplicate
**686**	10.3892/etm.2014.1582	3991500	Duplicate

**687**	10.4103/2230-8210.123552	4046605	Duplicate
**688**		4021780	Duplicate
**689**	10.1155/2014/653587	4009334	Duplicate
**690**	10.1155/2014/143763	4000629	Duplicate
**691**	10.1016/j.pharmthera.2008.05.005	4007052	Duplicate
**692**	10.1111/bph.12143	3651669	Duplicate
**693**	10.1155/2014/878670	3976909	Duplicate
**694**	10.3390/cells2020224	3972685	Duplicate
**695**	10.5489/cuaj.1608	3956835	Duplicate
**696**	10.2147/DMSO.S36455	3949699	Duplicate
**697**	10.2147/PPA.S48357	3920925	Duplicate
**698**	10.1097/MBP.0b013e328344c713	3915515	Duplicate
**699**	10.1016/j.jash.2011.02.007	3915530	Duplicate
**700**	10.4103/0975-7406.124317	3895294	Duplicate
**701**	10.1155/2013/504915	3884863	Duplicate
**702**	10.3205/cto000094	3884537	Duplicate
**703**	10.1371/journal.pone.0083951	3877124	Duplicate
**704**	10.1371/journal.pone.0085071	3873469	Duplicate
**705**	10.4111/kju.2013.54.12.858	3866290	Duplicate
**706**	10.1089/wound.2012.0422	3817001	Duplicate
**707**	10.4239/wjd.v4.i5.177	3797883	Duplicate
**708**		3776489	Duplicate
**709**	10.5534/wjmh.2013.31.2.83	3770856	Duplicate
**710**	10.5534/wjmh.2013.31.2.103	3770846	Duplicate
**711**	10.1038/aja.2010.123	3739608	Duplicate
**712**	10.1186/1471-244X-13-214	3766216	Duplicate
**713**	10.1016/j.juro.2012.04.001	3764461	Duplicate
**714**	10.1155/2013/323574	3728554	Duplicate
**715**	10.1007/s11606-011-1966-4	3378740	Duplicate
**716**	10.1097/HJH.0b013e32834000a7	3682653	Duplicate
**717**	10.1155/2013/763125	3665238	Duplicate
**718**	10.1164/rccm.201111-2082CI	3373067	Duplicate
**719**		3641729	Duplicate
**720**	10.5534/wjmh.2012.30.2.114	3623519	Duplicate
**721**	10.1007/s00125-011-2402-3	3329963	Duplicate
**722**	10.1186/1758-5996-4-43	3568051	Duplicate
**723**	10.1089/scd.2011.0303	3272247	Duplicate
**724**		3565859	Duplicate
**725**	10.1016/j.mayocp.2012.06.015	3498391	Duplicate
**726**	10.1155/2012/836893	3521631	Duplicate
**727**	10.1186/1471-2377-12-108	3517486	Duplicate
**728**		3410114	Duplicate
**729**	10.2147/VHRM.S26712	3433322	Duplicate
**730**	10.1210/jc.2010-2724	3167667	Duplicate
**731**		3413650	Duplicate
**732**	10.2337/dc10-2339	3120209	Duplicate
**733**		3351876	Duplicate
**734**	10.1136/amiajnl-2011-000113	3078666	Duplicate
**735**	10.1155/2012/569654	3303762	Duplicate
**736**	10.1038/nutd.2011.6	3302138	Duplicate
**737**	10.1186/1472-6904-12-5	3296596	Duplicate
**738**	10.4111/kju.2011.52.11.725	3242984	Duplicate

**739**	10.1016/j.juro.2010.12.098	3220602	Duplicate
**740**		3206546	Duplicate
**741**	10.1530/EJE-11-0221	3188848	Duplicate
**742**	10.2119/molmed.2011.00100	3188861	Duplicate
**743**	10.1186/1471-2261-11-36	3157429	Duplicate
**744**	10.2147/JPR.S21751	3141833	Duplicate
**745**		3048017	Duplicate
**746**	10.1124/pr.109.002014	2835398	Duplicate
**747**	10.2174/1874192401004010240	3026340	Duplicate
**748**		3020279	Duplicate
**749**	10.4065/mcp.2010.0164	2894722	Duplicate
**750**		2094729	Duplicate
**751**	10.1186/1475-2840-9-55	2954908	Duplicate
**752**	10.1152/ajpendo.90996.2008	2763792	Duplicate
**753**		2941787	Duplicate
**754**	10.1007/s00125-010-1819-4	2931646	Duplicate
**755**		2854169	Duplicate
**756**		2911822	Duplicate
**757**	10.1186/1472-6963-10-128	2893175	Duplicate
**758**	10.1186/1477-7827-8-50	2887879	Duplicate
**759**		1913720	Duplicate
**760**		2849981	Duplicate
**761**	10.2337/dc07-2375	2660483	Duplicate
**762**	10.1007/s11606-009-0963-3	2670986	Duplicate
**763**		2809989	Duplicate
**764**		2801586	Duplicate
**765**	10.1007/s00431-006-0349-z	2799065	Duplicate
**766**	10.2174/157340308785160589	2780822	Duplicate
**767**		2730071	Duplicate
**768**	10.4103/0970-1591.52907	2710058	Duplicate
**769**		2664585	Duplicate
**770**		2699638	Duplicate
**771**	10.3748/wjg.14.1564	2693753	Duplicate
**772**		2686330	Duplicate
**773**	10.4103/0970-1591.42612	2684375	Duplicate
**774**		2544367	Duplicate
**775**		1860735	Duplicate
**776**	10.1186/1475-2840-8-19	2667490	Duplicate
**777**	10.1186/1476-4598-7-82	2615789	Duplicate
**778**		1853342	Duplicate
**779**	10.1007/s00592-008-0030-2	2335289	Duplicate
**780**		1994005	Duplicate
**781**	10.1016/j.blre.2006.07.001	2048670	Duplicate
**782**	10.1111/j.1525-1497.2006.00469.x	1484878	Duplicate
**783**		1716227	Duplicate
**784**		1502384	Duplicate
**785**	10.1111/j.1525-1497.2005.020S1.x	1490295	Duplicate
**786**		1495721	Duplicate
**787**	10.1172/JCI27758	1359065	Duplicate
**788**		1120492	Duplicate
**789**		1117946	Duplicate
**790**	10.1186/1475-2840-2-8	194431	Duplicate

k)

#	DOI	PMID	STATUS
**1**	10.1186/s13098-024-01363-3	38915115	Duplicate
**2**	10.1016/j.eclinm.2023.101985	37256099	Duplicate
**3**		36305253	Not a clinical trial
**4**	10.1126/scitranslmed.abl8503	35704597	Duplicate
**5**	10.1186/s13098-022-00825-w	35449082	Duplicate
**6**	10.1111/and.14421	35301742	Duplicate
**7**	10.1152/ajpcell.00595.2020	33788629	Duplicate
**8**	10.1097/MJT.0000000000001303	33369909	Duplicate
**9**	10.1111/andr.12919	33022887	Duplicate
**10**	10.4103/aja.aja_121_19	31696836	Duplicate
**11**	10.1016/j.jsxm.2019.01.003	30773497	Duplicate
**12**	10.1016/j.jcte.2016.11.003	29067241	Duplicate
**13**	10.1080/13685538.2016.1260107	28084147	Duplicate
**14**	10.1002/ejhf.662	27873388	Duplicate
**15**	10.1016/j.jsxm.2016.04.075	27235284	Duplicate
**16**	10.1517/14728222.2015.1066337	26178526	Duplicate
**17**	10.1038/ijir.2010.21	20811390	Duplicate
**18**		20565373	Duplicate
**19**	10.1038/ijir.2009.51	19907424	Not a clinical trial
**20**	10.2298/vsp0706399p	17687944	Duplicate
**21**	10.1016/j.eururo.2007.04.042	17478034	Duplicate
**22**	10.1016/j.diabres.2007.02.006	17374416	Duplicate
**23**	10.1111/j.1743-6109.2006.00295.x	16942532	Duplicate
**24**	10.1111/j.1442-2042.2006.01480.x	16903931	Duplicate
**25**	10.1016/j.fertnstert.2005.10.043	16579999	Duplicate
**26**	10.1007/s00125-004-1549-6	15599697	Duplicate
**27**		15562798	Duplicate
**28**		14971333	Duplicate
**29**	10.2337/diacare.26.3.777	12610037	Duplicate
**30**	10.2337/diacare.25.12.2159	12453954	Duplicate
**31**	10.1016/s0212-6567(02)79030-6	12372210	Duplicate
**32**	10.1007/s001250100656	11692178	Duplicate

Supplementary References

1. Cochrane. Cochrane Handbook for Systematic Reviews of Interventions version 6.4. August 2024 2024. www.training.cochrane.org/handbook (accessed 2024-10-27.
2. Page MJ, McKenzie JE, Bossuyt PM, et al. The PRISMA 2020 statement: an updated guideline for reporting systematic reviews. *BMJ* 2021; **372**: n71.
3. Hoelzel W, Weykamp C, Jeppsson JO, et al. IFCC reference system for measurement of hemoglobin A1c in human blood and the national standardization schemes in the United States, Japan, and Sweden: a method-comparison study. *Clin Chem* 2004; **50**(1): 166-74.
4. Corona G, Cucinotta D, Di Lorenzo G, et al. The Italian Society of Andrology and Sexual Medicine (SIAMS), along with ten other Italian Scientific Societies, guidelines on the diagnosis and management of erectile dysfunction. *J Endocrinol Invest* 2023; **46**(6): 1241-74.
5. Xu Y, Dunn TC, Ajjan RA. A Kinetic Model for Glucose Levels and Hemoglobin A1c Provides a Novel Tool for Individualized Diabetes Management. *J Diabetes Sci Technol* 2021; **15**(2): 294-302.
6. Carlisle JB. Data fabrication and other reasons for non-random sampling in 5087 randomised, controlled trials in anaesthetic and general medical journals. *Anaesthesia* 2017; **72**(8): 944-52.
7. Hegazy SK, Amaar WA, Hegab WSM. Tadalafil versus pentoxifylline in the management of diabetic kidney disease: a randomized clinical trial. *Diabetol Metab Syndr* 2024; **16**(1): 138.
8. Fryk E, Rodrigues Silva VR, Bauza-Thorbrugge M, et al. Feasibility of high-dose tadalafil and effects on insulin resistance in well-controlled patients with type 2 diabetes (MAKROTAD): a single-centre, double-blind, randomised, placebo-controlled, cross-over phase 2 trial. *EClinicalMedicine* 2023; **59**: 101985.
9. Pofi R, Giannetta E, Feola T, et al. Sex-specific effects of daily tadalafil on diabetic heart kinetics in RECOGITO, a randomized, double-blind, placebo-controlled trial. *Sci Transl Med* 2022; **14**(649): eabl8503.
10. Lee MK, Lee JH, Sohn SY, Lee SY, Jeong TY, Kim SC. Effect of low-dose tadalafil once daily on glycemic control in patients with type 2 diabetes and erectile dysfunction: a randomized, double-blind, placebo-controlled pilot study. *Diabetol Metab Syndr* 2022; **14**(1): 56.
11. Derosa G, D'Angelo A, Preti PS, Maffioli P. Evaluation of the Effect on Sexual Performance of a Nutraceutical Combination Containing Alpha Lipoic Acid, Vitis vinifera L. and Ginkgo biloba, Compared to Placebo, Avanafil or a Combination of Nutraceutical Plus Avanafil in Males With Type 2 Diabetes Mellitus With Erectile Dysfunction. *Front Endocrinol (Lausanne)* 2022; **13**: 847240.
12. Derosa G, Tinelli C, D'Angelo A, et al. Glyco-metabolic profile among type 2 diabetic patients with erectile dysfunction. *Endocr J* 2012; **59**(7): 611-9.
13. Derosa G, D'Angelo A, Maffioli P. Change of some oxidative stress parameters after supplementation with whey protein isolate in patients with type 2 diabetes. *Nutrition* 2020; **73**: 110700.
14. Derosa G, D'Angelo A, Maffioli P. Ilex paraguariensis, white mulberry and chromium picolinate in patients with pre-diabetes. *Phytother Res* 2020; **34**(6): 1377-84.
15. Liu LC, Hummel YM, van der Meer P, et al. Effects of sildenafil on cardiac structure and function, cardiopulmonary exercise testing and health-related quality of life measures in heart failure patients with preserved ejection fraction and pulmonary hypertension. *Eur J Heart Fail* 2017; **19**(1): 116-25.
16. Hoendermis ES, Liu LC, Hummel YM, et al. Effects of sildenafil on invasive haemodynamics and exercise capacity in heart failure patients with preserved ejection fraction and pulmonary hypertension: a randomized controlled trial. *Eur Heart J* 2015; **36**(38): 2565-73.
17. Hoendermis ES. Sildenafil in HFpEF (Heart Failure With Preserved Ejection Fraction) and PH. 2016-03-21 ed. ClinicalTrials.gov; 2016.
18. Scheele W, Diamond S, Gale J, et al. Phosphodiesterase Type 5 Inhibition Reduces Albuminuria in Subjects with Overt Diabetic Nephropathy. *J Am Soc Nephrol* 2016; **27**(11): 3459-68.
19. Kirilmaz U, Guzel O, Aslan Y, Balci M, Tuncel A, Atan A. The effect of lifestyle modification and glycemic control on the efficiency of sildenafil citrate in patients with erectile dysfunction due to type-2 diabetes mellitus. *Aging Male* 2015; **18**(4): 244-8.
20. Tuncel A, Balci M, Postaci A, Aslan Y, Atan A. Comparison of different postoperative pain managements in patients submitted to transperitoneal laparoscopic renal and adrenal surgery. *Int Braz J Urol* 2015; **41**(4): 669-75.
21. Keten T, Aslan Y, Balci M, et al. Determination of the efficiency of 8 mg doxazosin XL treatment in patients with an inadequate response to 4 mg doxazosin XL treatment for benign prostatic hyperplasia. *Urology* 2015; **85**(1): 189-94.
22. Khazaal FA. Low dose tadalafil effect on anthropometric and metabolic parameters in Iraqi diabetic obese men. *Al-Kindy College Medical Journal* 2014; **10**(2): 94-8.
23. Giannetta E, Isidori AM, Galea N, et al. Chronic Inhibition of cGMP phosphodiesterase 5A improves diabetic cardiomyopathy: a randomized, controlled clinical trial using magnetic resonance imaging with myocardial tagging. *Circulation* 2012; **125**(19): 2323-33.
24. Morano S, Mandosi E, Fallarino M, et al. Antioxidant treatment associated with sildenafil reduces monocyte activation and markers of endothelial damage in patients with diabetic erectile dysfunction: a double-blind, placebo-controlled study. *Eur Urol* 2007; **52**(6): 1768-74.
25. Agency EM. ICH E6 (R2) Good clinical practice - Scientific guideline. 1997. https://www.ema.europa.eu/en/ich-e6-r2-good-clinical-practice-scientific-guideline.
26. Jannini EA, Isidori AM, Gravina GL, et al. The ENDOTRIAL study: a spontaneous, open-label, randomized, multicenter, crossover study on the efficacy of sildenafil, tadalafil, and vardenafil in the treatment of erectile dysfunction. *J Sex Med* 2009; **6**(9): 2547-60.
27. Aversa A, Letizia C, Francomano D, Bruzziches R, Natali M, Lenzi A. A spontaneous, double-blind, double-dummy cross-over study on the effects of daily vardenafil on arterial stiffness in patients with vasculogenic erectile dysfunction. *Int J Cardiol* 2012; **160**(3): 187-91.
28. Grover-Paez F, Villegas Rivera G, Guillen Ortiz R. Sildenafil citrate diminishes microalbuminuria and the
